# Supplementary figures and images for: Effector membrane translocation biosensors reveal G protein and βarrestin coupling profiles of 100 therapeutically relevant GPCRs
Source: eLife. 2022 Mar 18;11:e74101. doi: 10.7554/eLife.74101 (PMC9005190; doi:10.7554/eLife.74101)

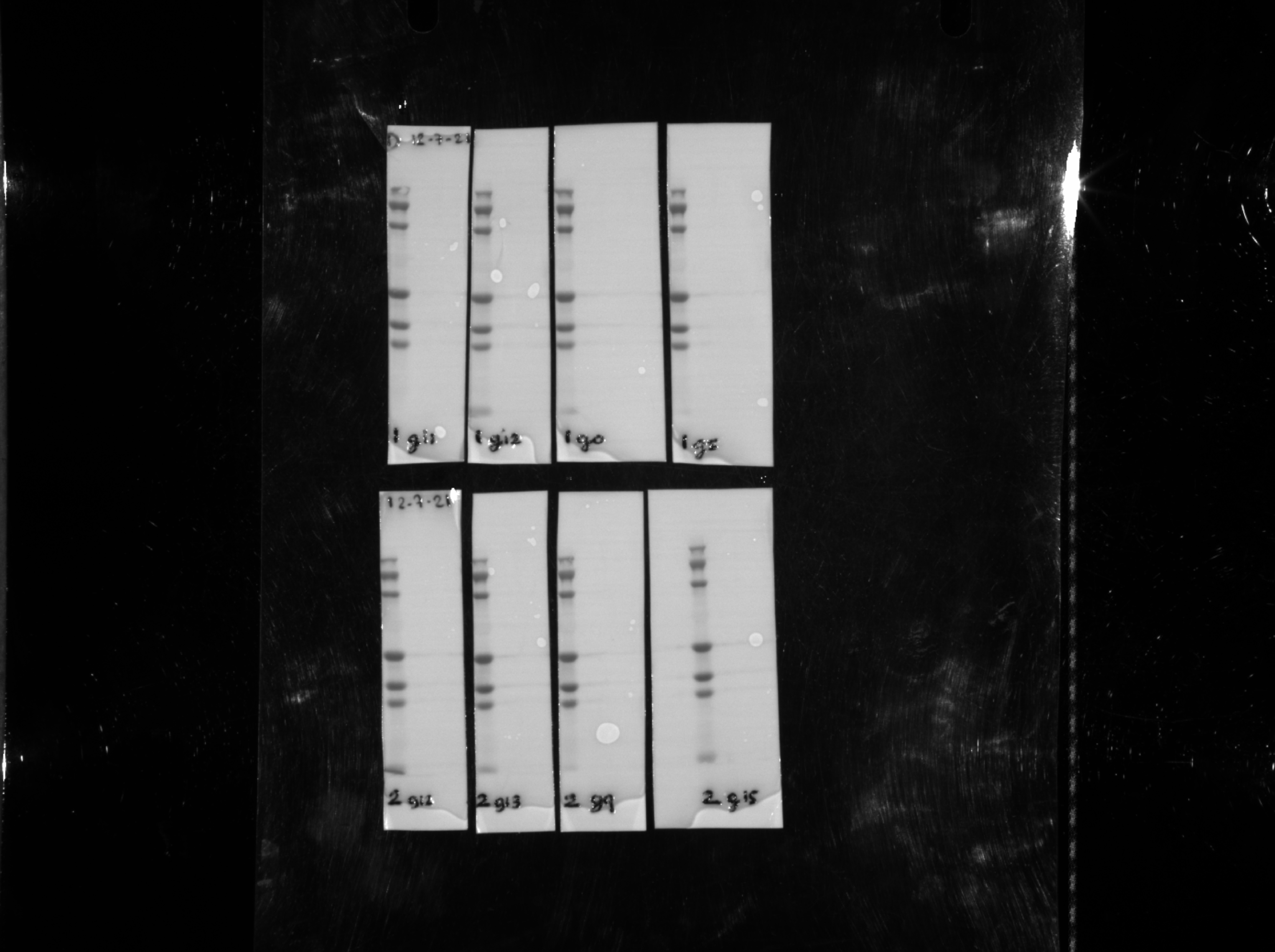

Supplement: Figure 2—figure supplement 6—source data 1. [file elife-74101-fig2-figsupp6-data1.zip › Figure 2- figure supplement 6_Source Data 1/EPIDIG WB bActin Ab Gi1, i2, oA+B,12, 13, q, 15, s.tif]

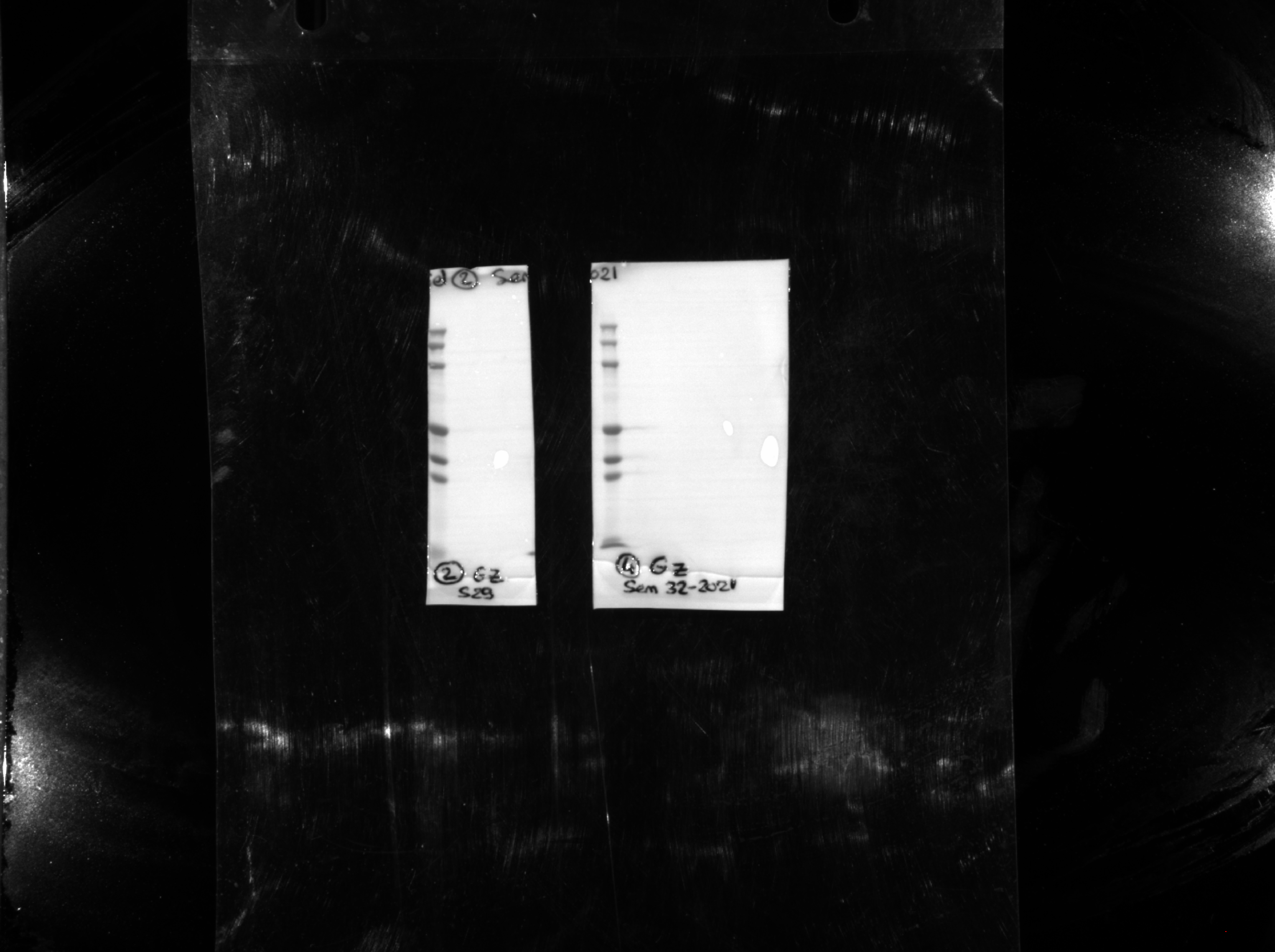

Supplement: Figure 2—figure supplement 6—source data 1. [file elife-74101-fig2-figsupp6-data1.zip › Figure 2- figure supplement 6_Source Data 1/EPIDIG WB bActin Ab Gz.tif]

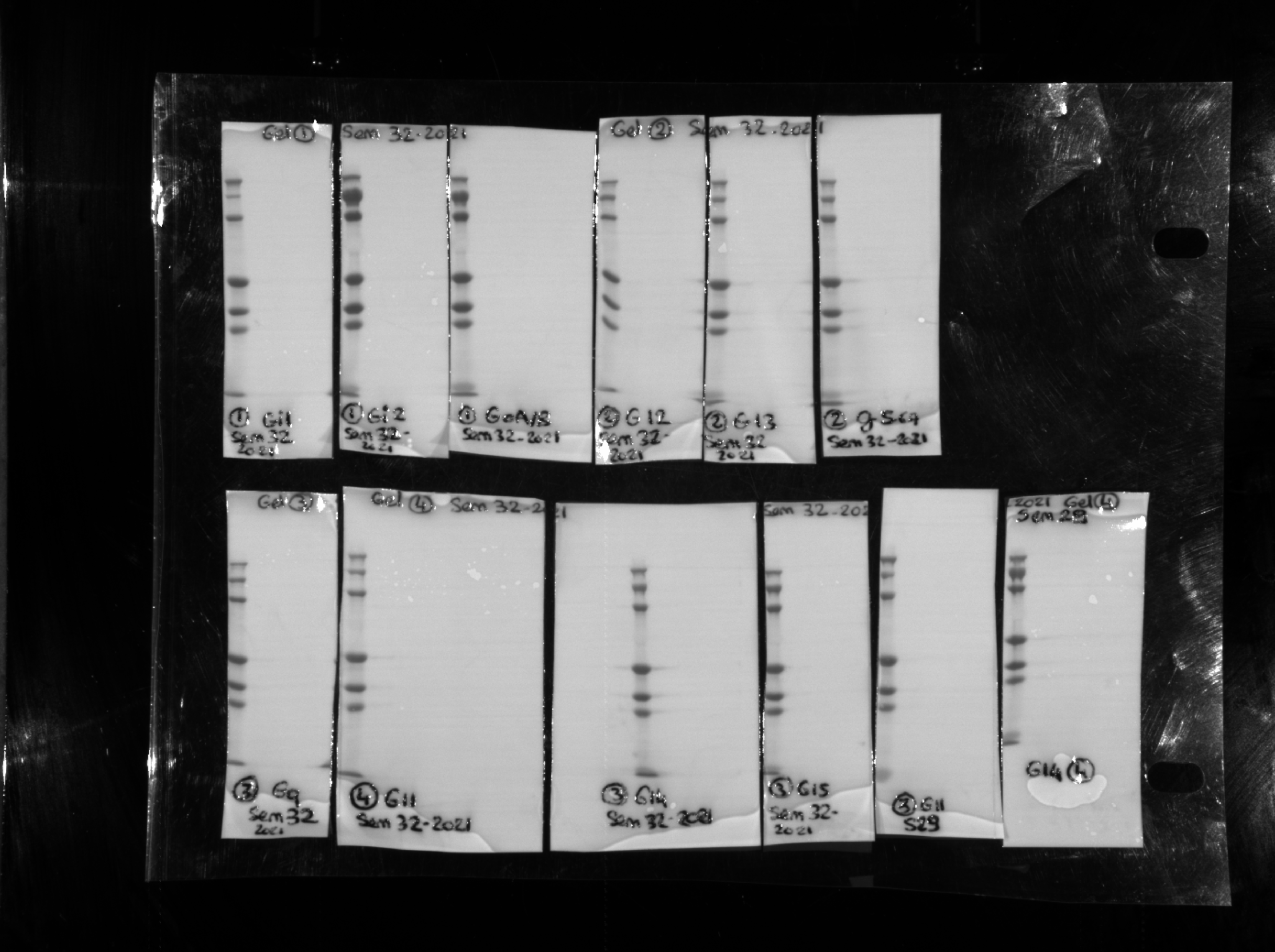

Supplement: Figure 2—figure supplement 6—source data 1. [file elife-74101-fig2-figsupp6-data1.zip › Figure 2- figure supplement 6_Source Data 1/EPIDIG WB bActin prot G11, 14.tif]

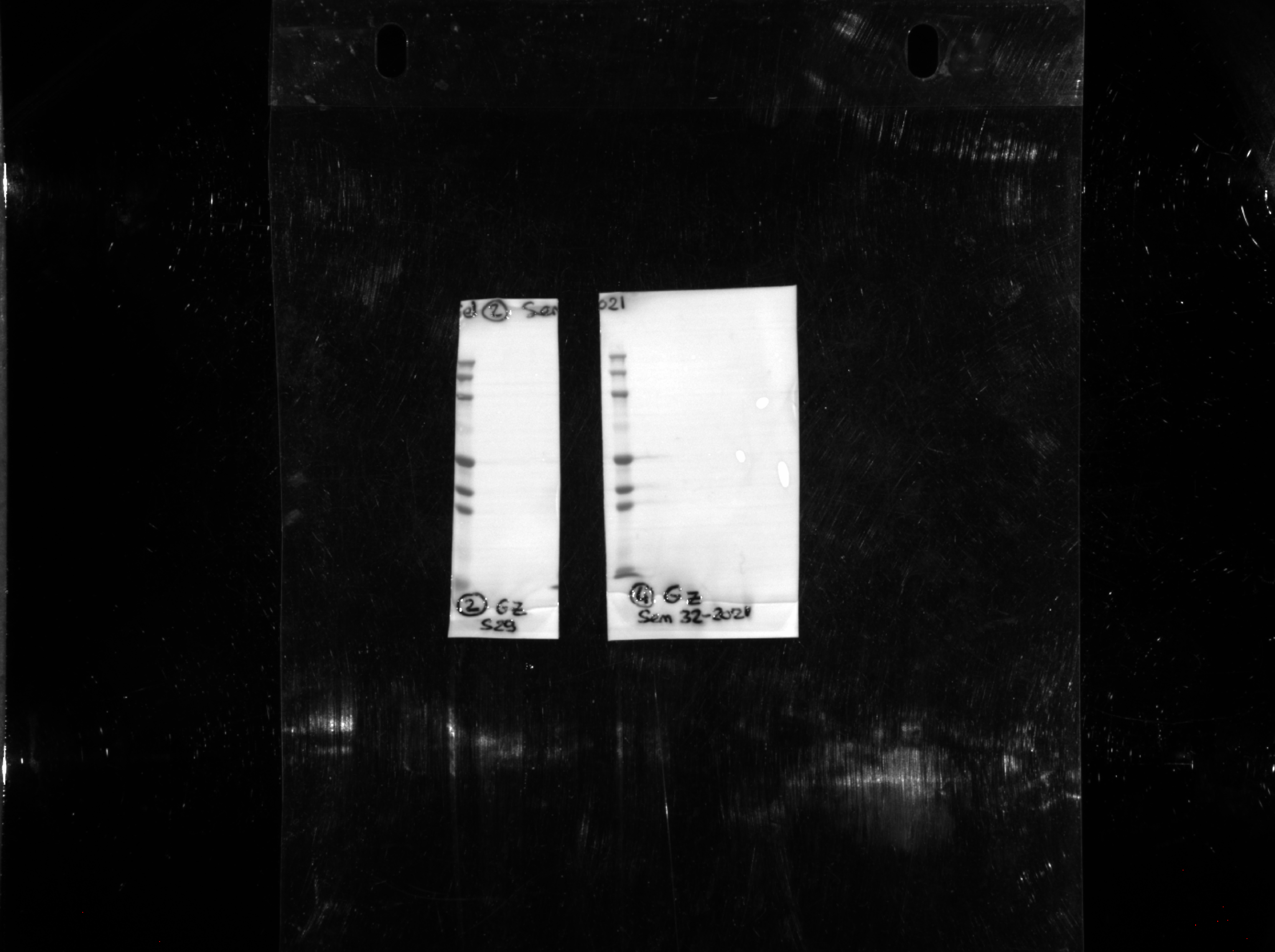

Supplement: Figure 2—figure supplement 6—source data 1. [file elife-74101-fig2-figsupp6-data1.zip › Figure 2- figure supplement 6_Source Data 1/EpiDig WB Gz.tif]

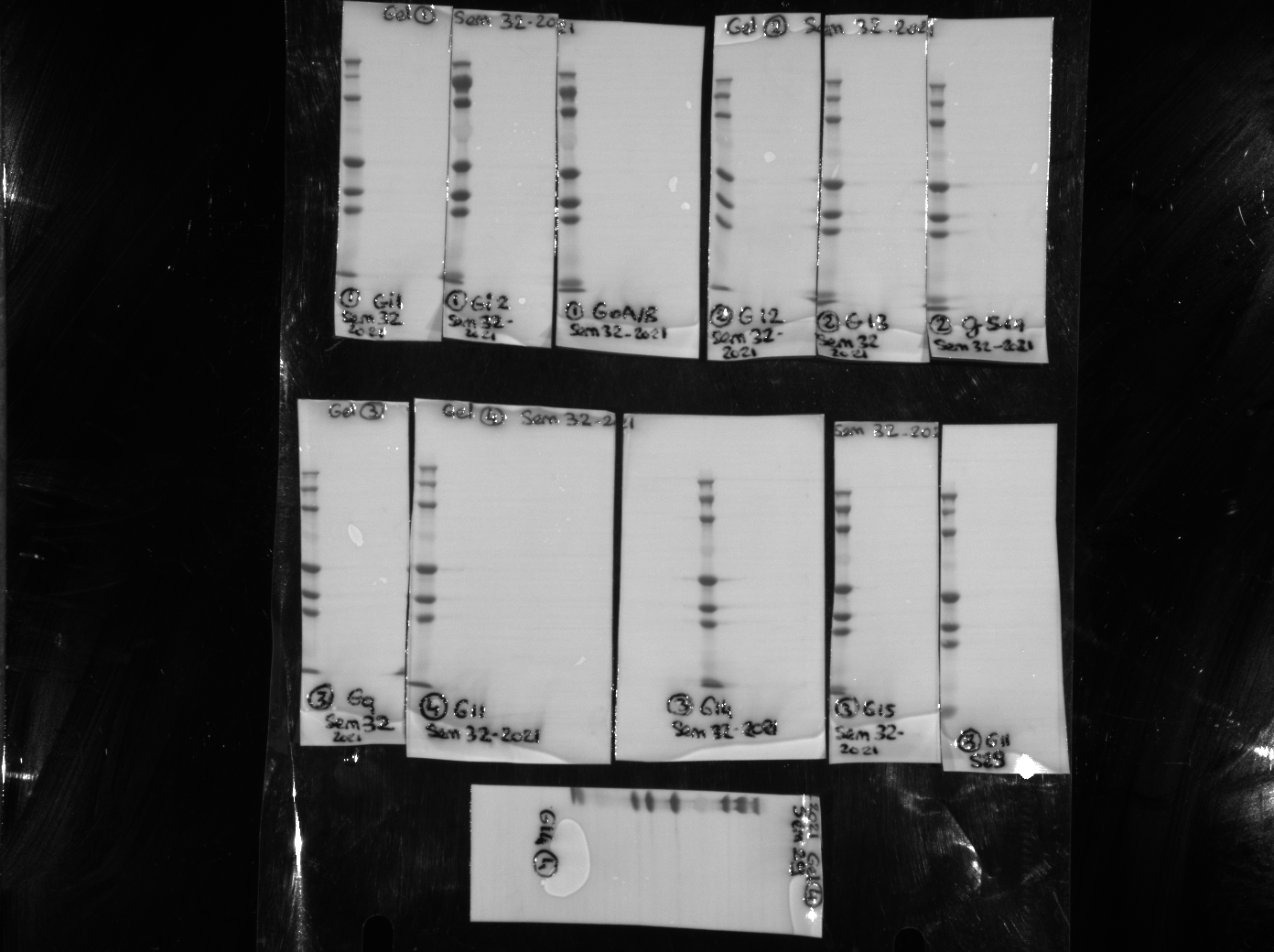

Supplement: Figure 2—figure supplement 6—source data 1. [file elife-74101-fig2-figsupp6-data1.zip › Figure 2- figure supplement 6_Source Data 1/EPIDIG WB Prot G11.tif]

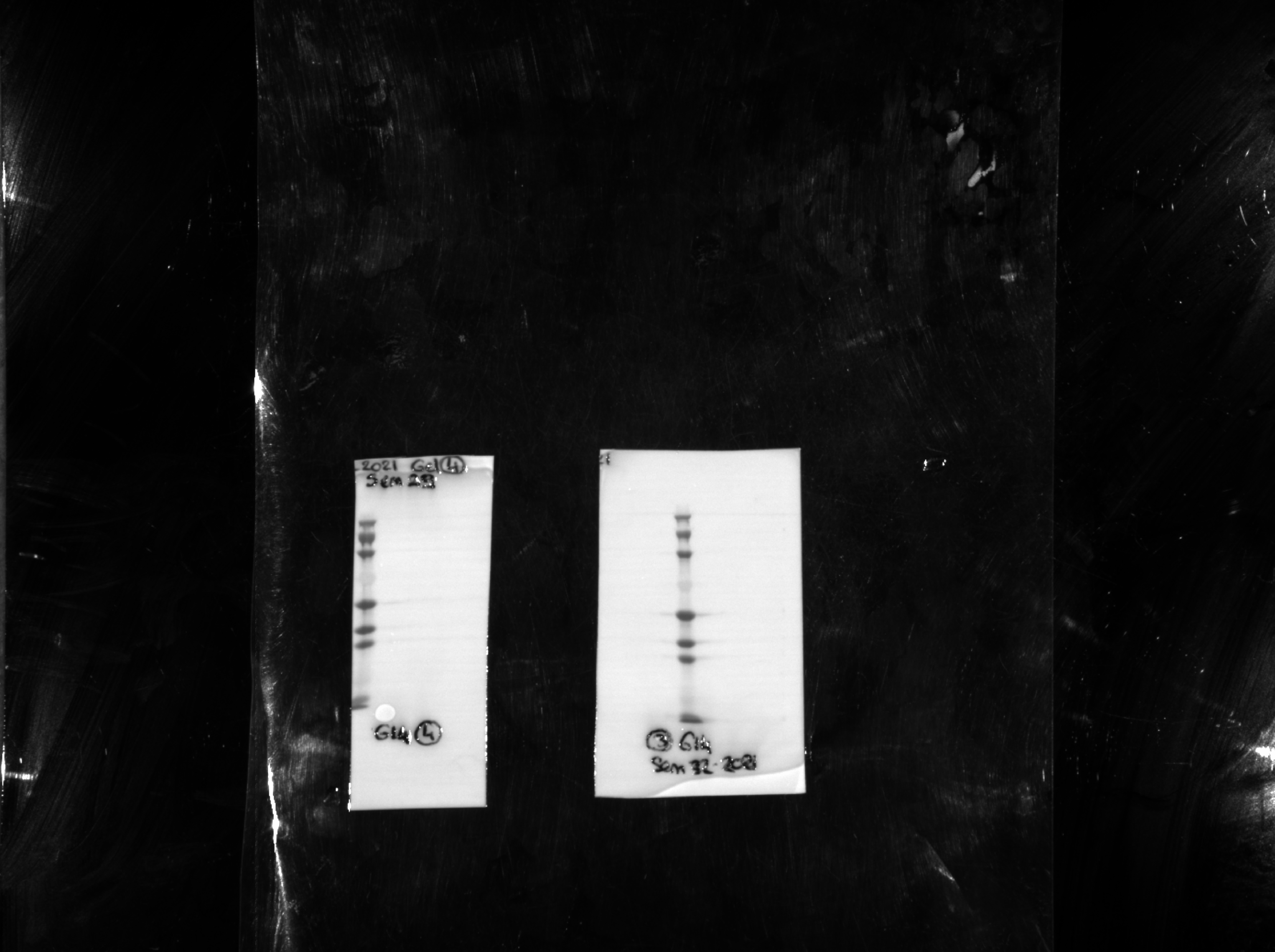

Supplement: Figure 2—figure supplement 6—source data 1. [file elife-74101-fig2-figsupp6-data1.zip › Figure 2- figure supplement 6_Source Data 1/EPIDIG WB prot G14.tif]

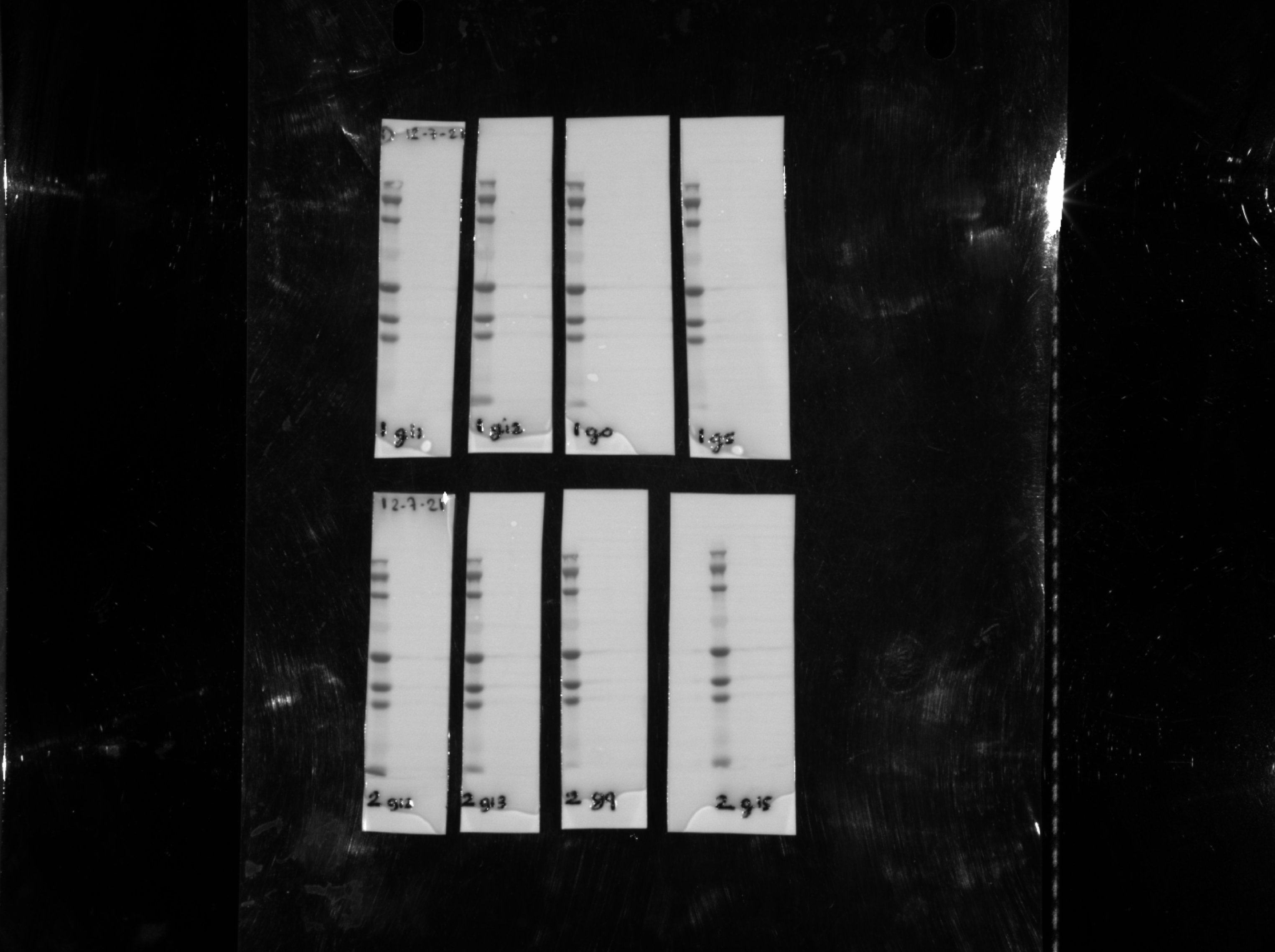

Supplement: Figure 2—figure supplement 6—source data 1. [file elife-74101-fig2-figsupp6-data1.zip › Figure 2- figure supplement 6_Source Data 1/EPIDIG WB prot Gi1, i2, oA+B,12, 13, q, 15, s.tif]

Figure 2- figure supplement 6; Uncropped blots

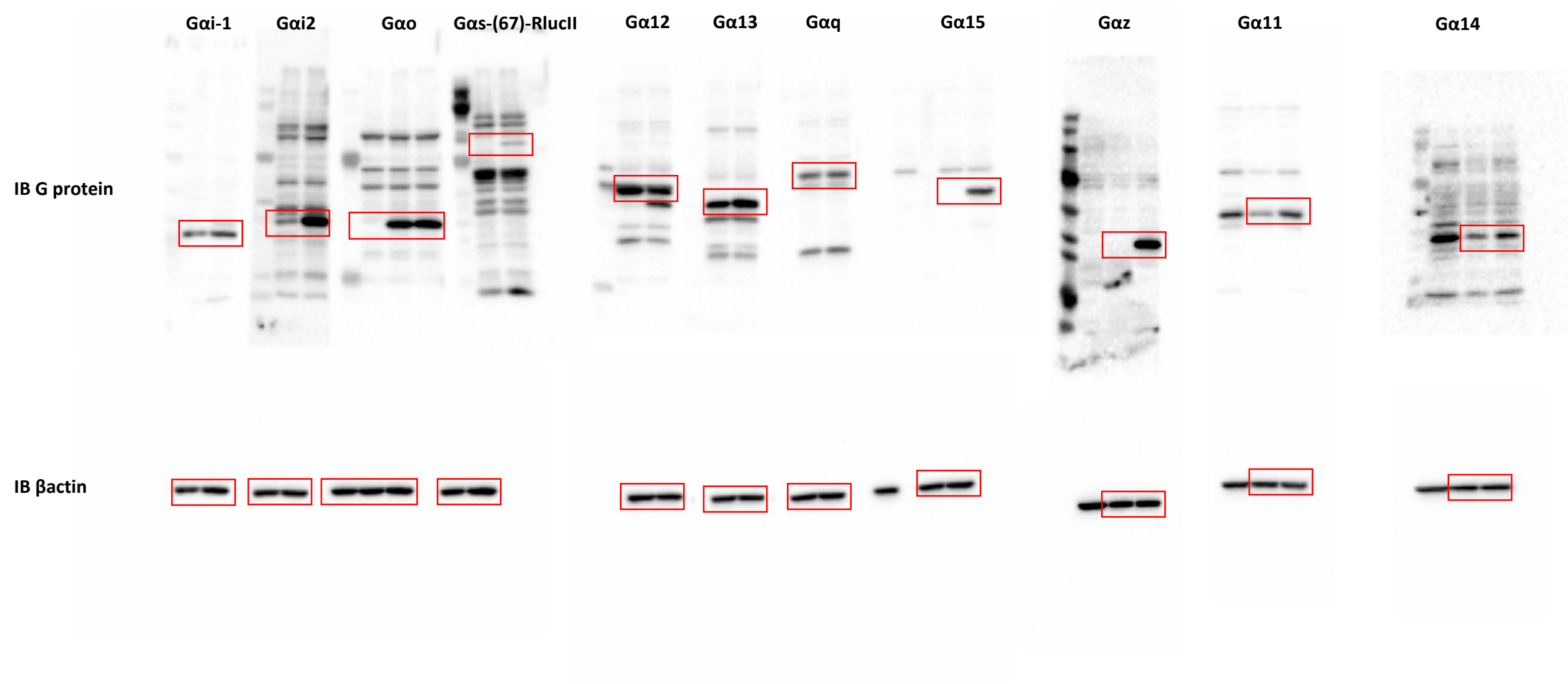

Supplement: Figure 2—figure supplement 6—source data 1. [file elife-74101-fig2-figsupp6-data1.zip › Figure 2- figure supplement 6_Source Data 1/Figure 2- figure supplement 6_Uncropped Blots.pdf]

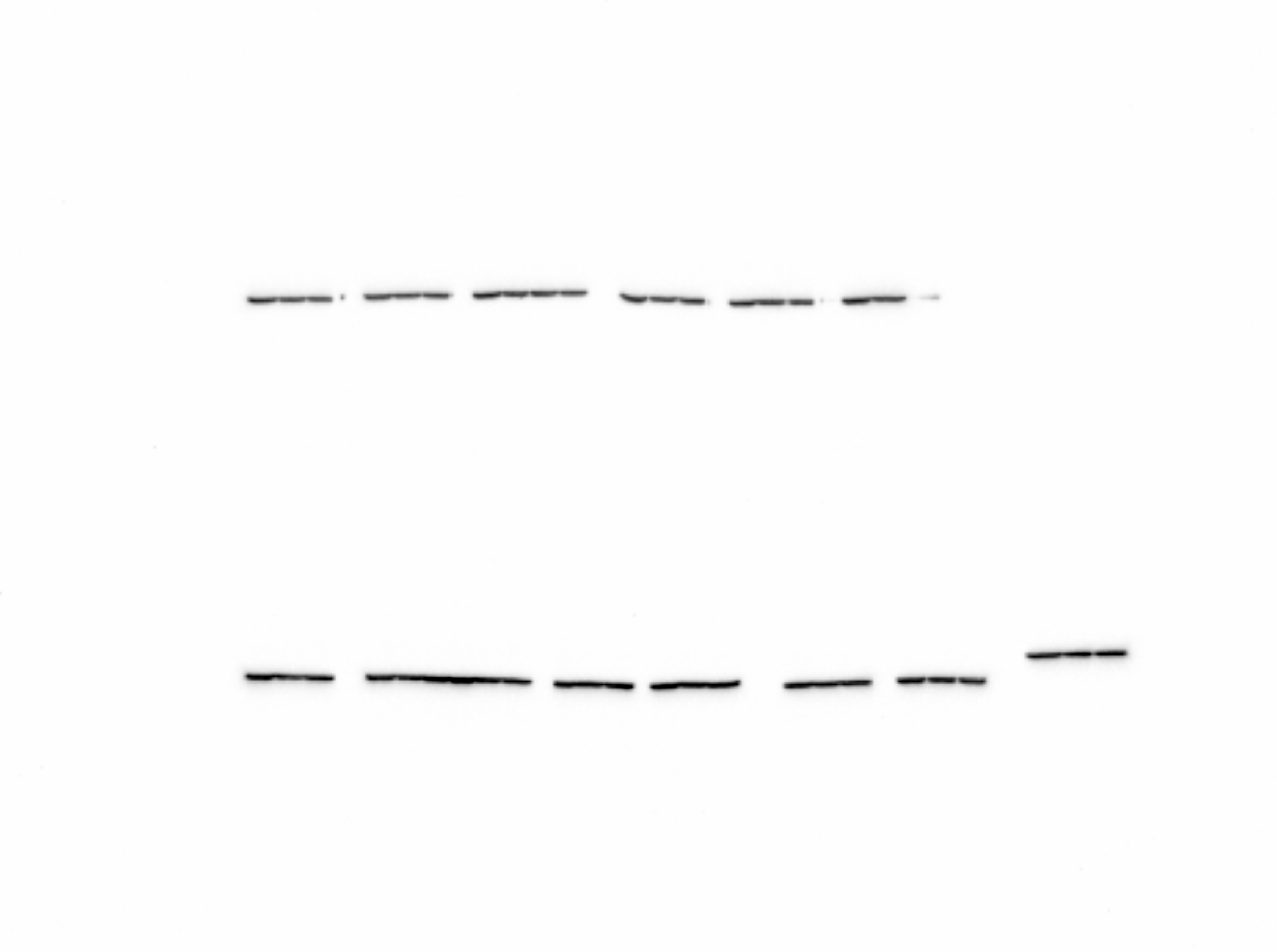

Supplement: Figure 2—figure supplement 6—source data 1. [file elife-74101-fig2-figsupp6-data1.zip › Figure 2- figure supplement 6_Source Data 1/WB bActin prot G11, 14.tif]

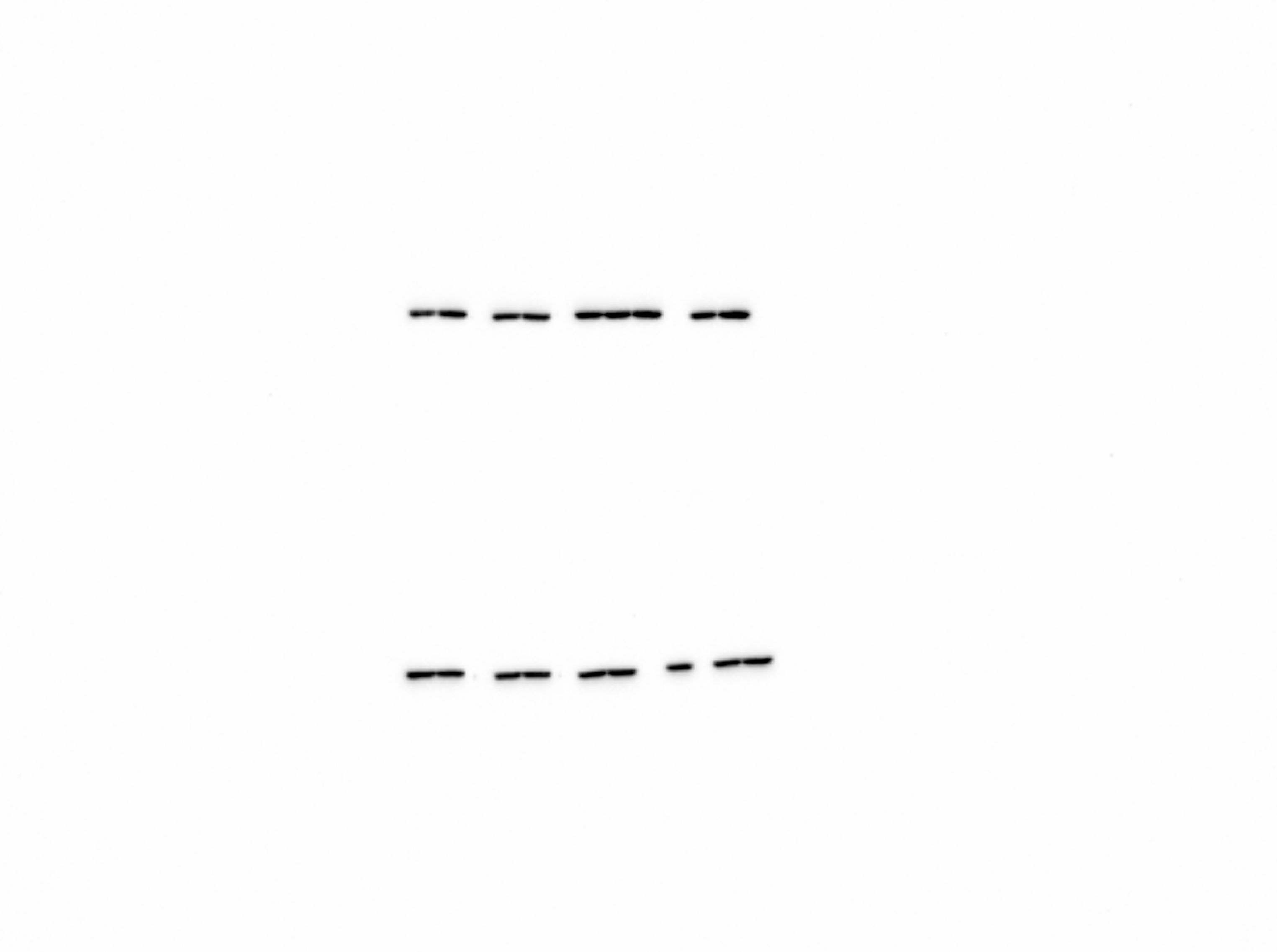

Supplement: Figure 2—figure supplement 6—source data 1. [file elife-74101-fig2-figsupp6-data1.zip › Figure 2- figure supplement 6_Source Data 1/WB bActin prot Gi1, i2, oA+B,12, 13, q, 15, s.tif]

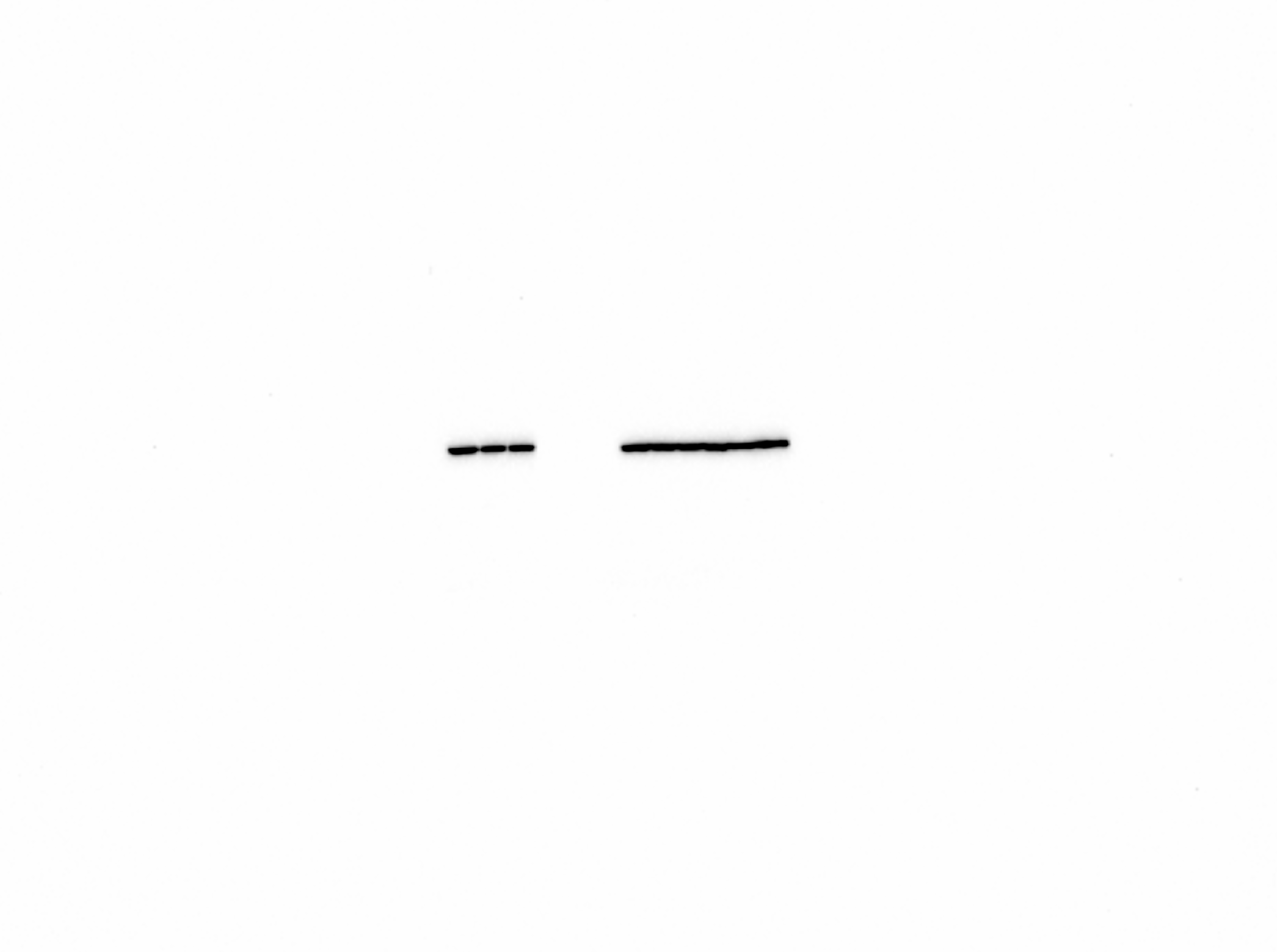

Supplement: Figure 2—figure supplement 6—source data 1. [file elife-74101-fig2-figsupp6-data1.zip › Figure 2- figure supplement 6_Source Data 1/WB bActin prot Gz.tif]

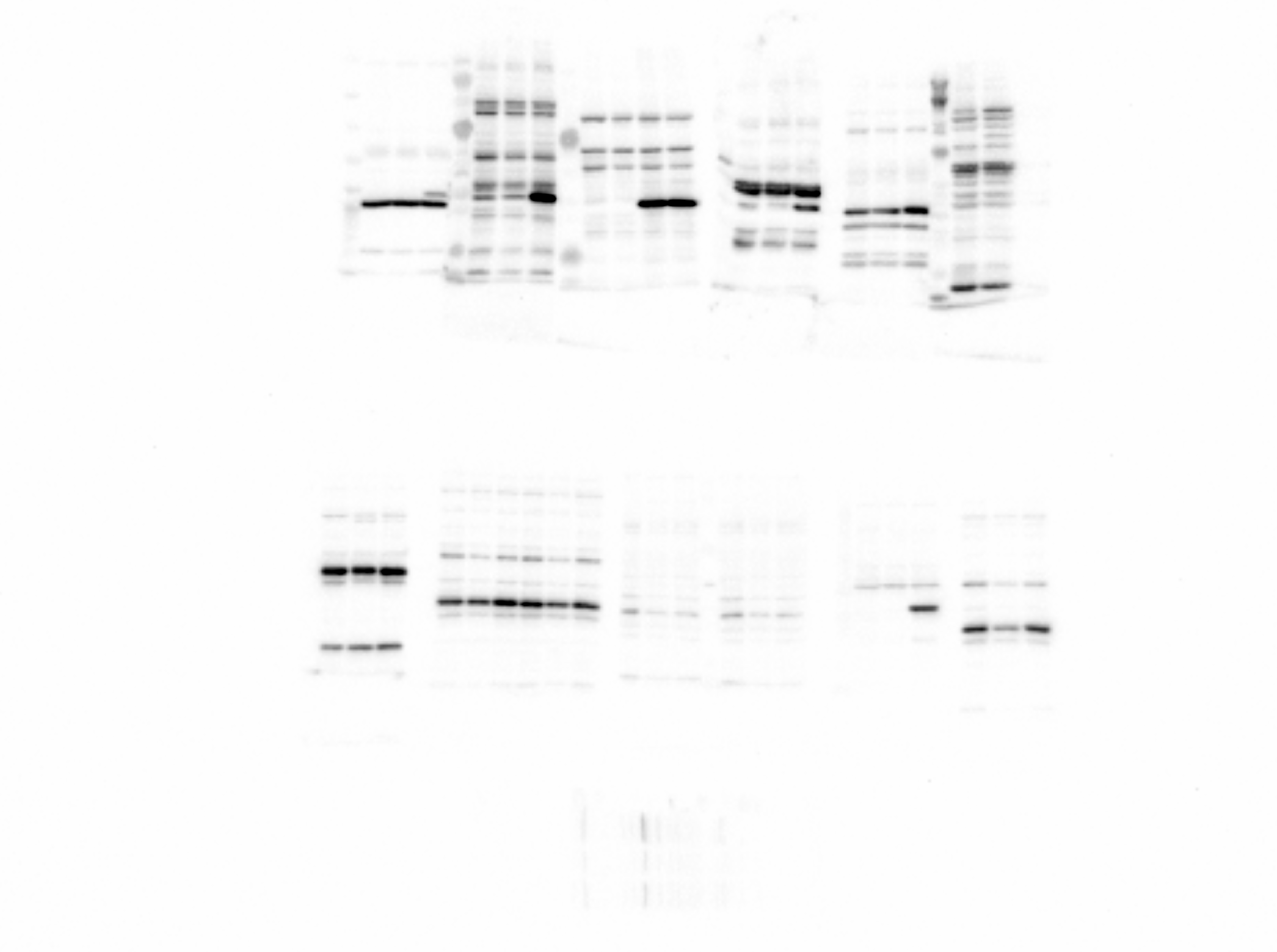

Supplement: Figure 2—figure supplement 6—source data 1. [file elife-74101-fig2-figsupp6-data1.zip › Figure 2- figure supplement 6_Source Data 1/WB Prot G11.tif]

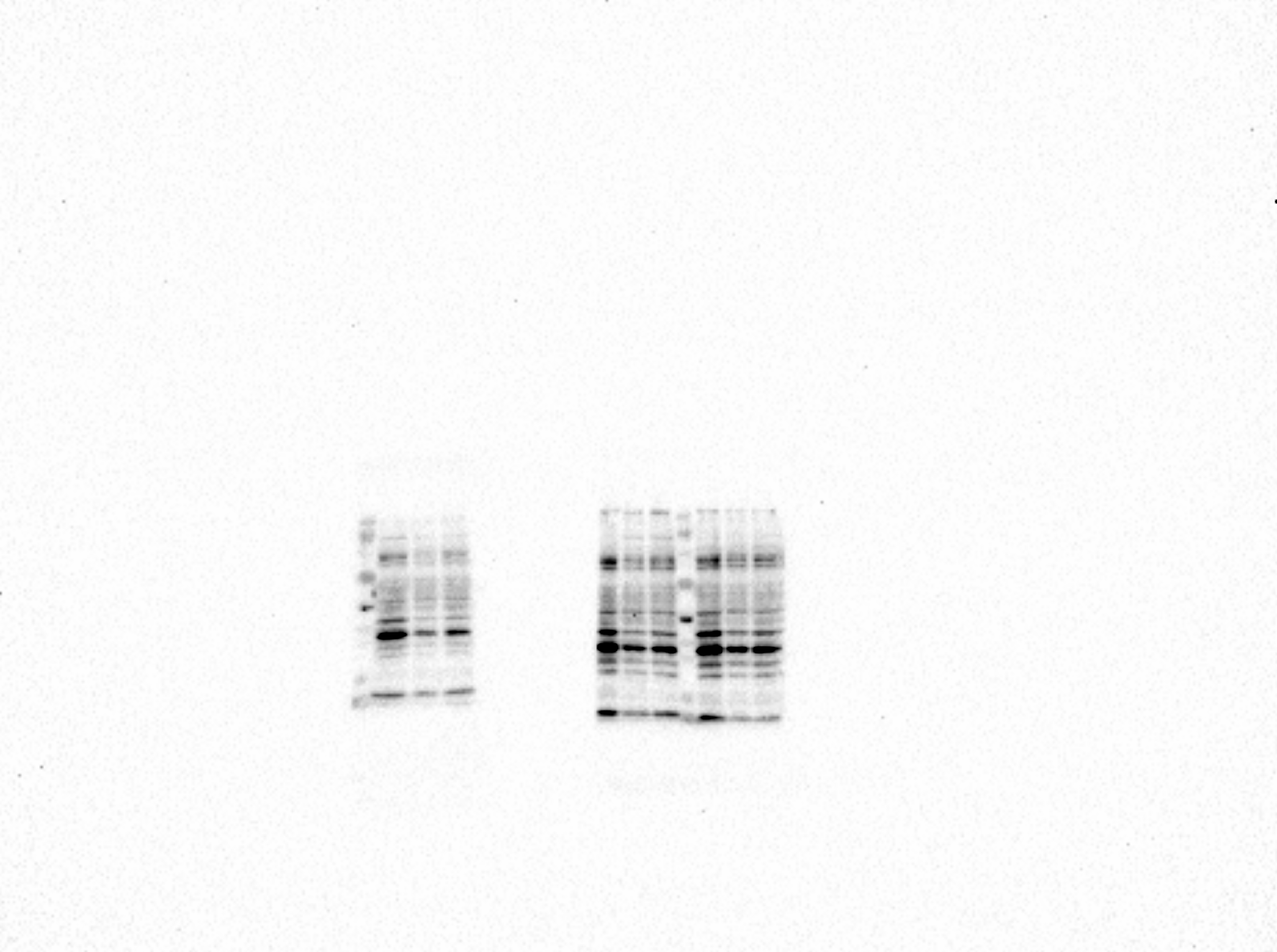

Supplement: Figure 2—figure supplement 6—source data 1. [file elife-74101-fig2-figsupp6-data1.zip › Figure 2- figure supplement 6_Source Data 1/WB prot G14.tif]

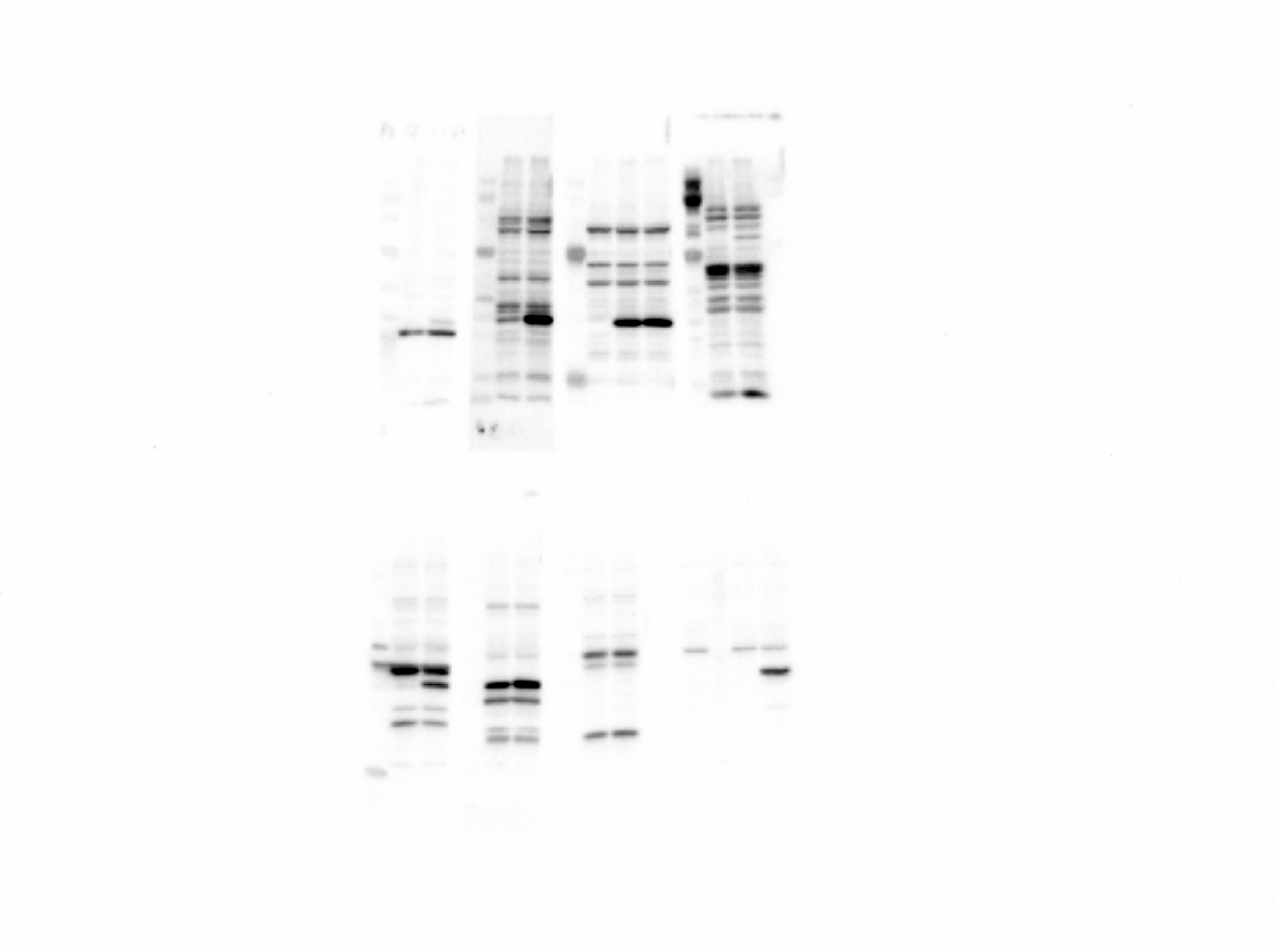

Supplement: Figure 2—figure supplement 6—source data 1. [file elife-74101-fig2-figsupp6-data1.zip › Figure 2- figure supplement 6_Source Data 1/WB prot Gi1, i2, oA+B,12, 13, q, 15, s.tif]

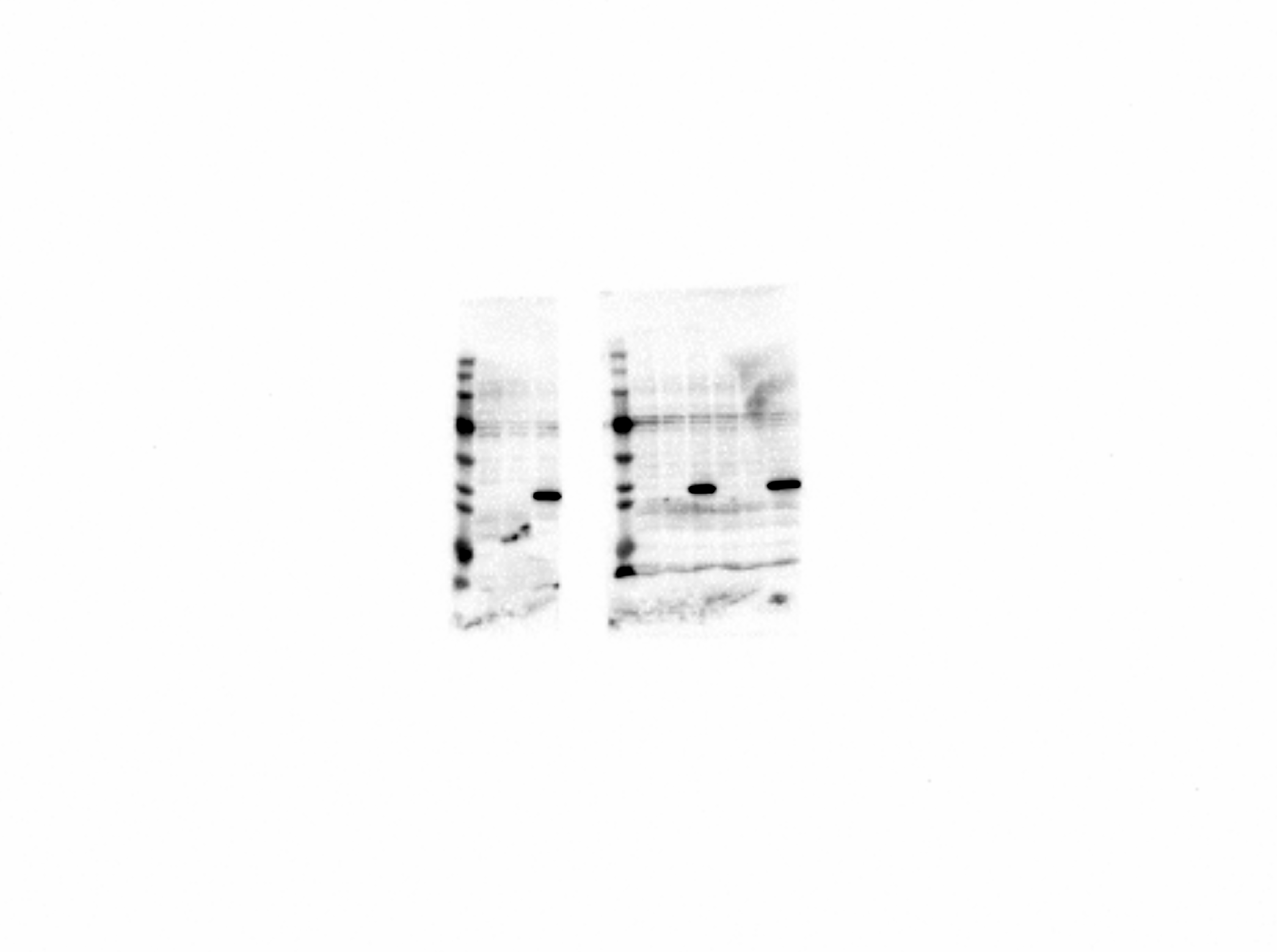

Supplement: Figure 2—figure supplement 6—source data 1. [file elife-74101-fig2-figsupp6-data1.zip › Figure 2- figure supplement 6_Source Data 1/WB prot Gz.tif]
