# Supplementary material for: Effector membrane translocation biosensors reveal G protein and βarrestin coupling profiles of 100 therapeutically relevant GPCRs
Source: eLife. 2022 Mar 18;11:e74101. doi: 10.7554/eLife.74101 (PMC9005190; doi:10.7554/eLife.74101)

GPCR: 5-HT_{1A}
Ligand: Serotonin

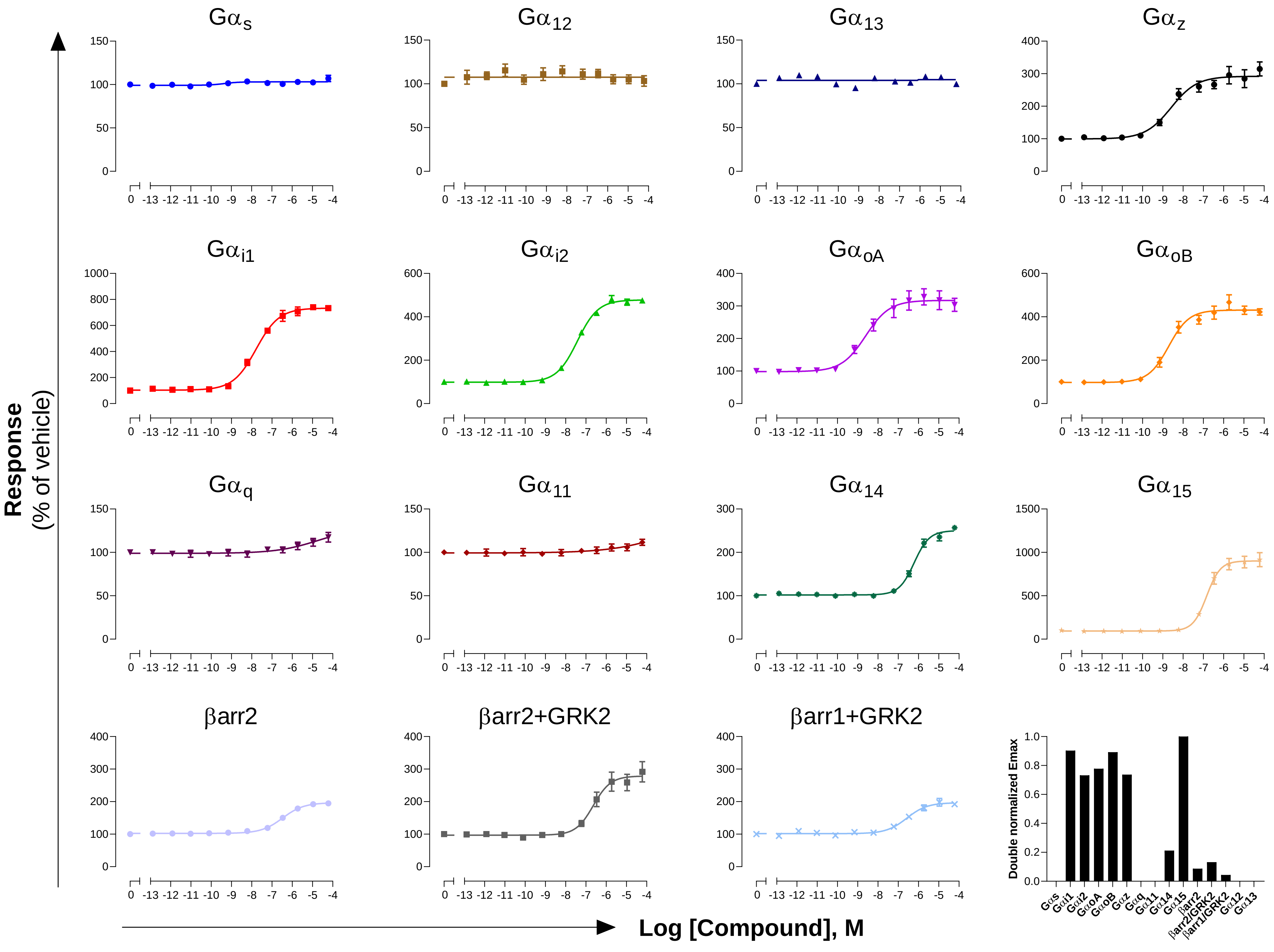

GPCR: 5-HT$_{1B}$
Ligand: Serotonin

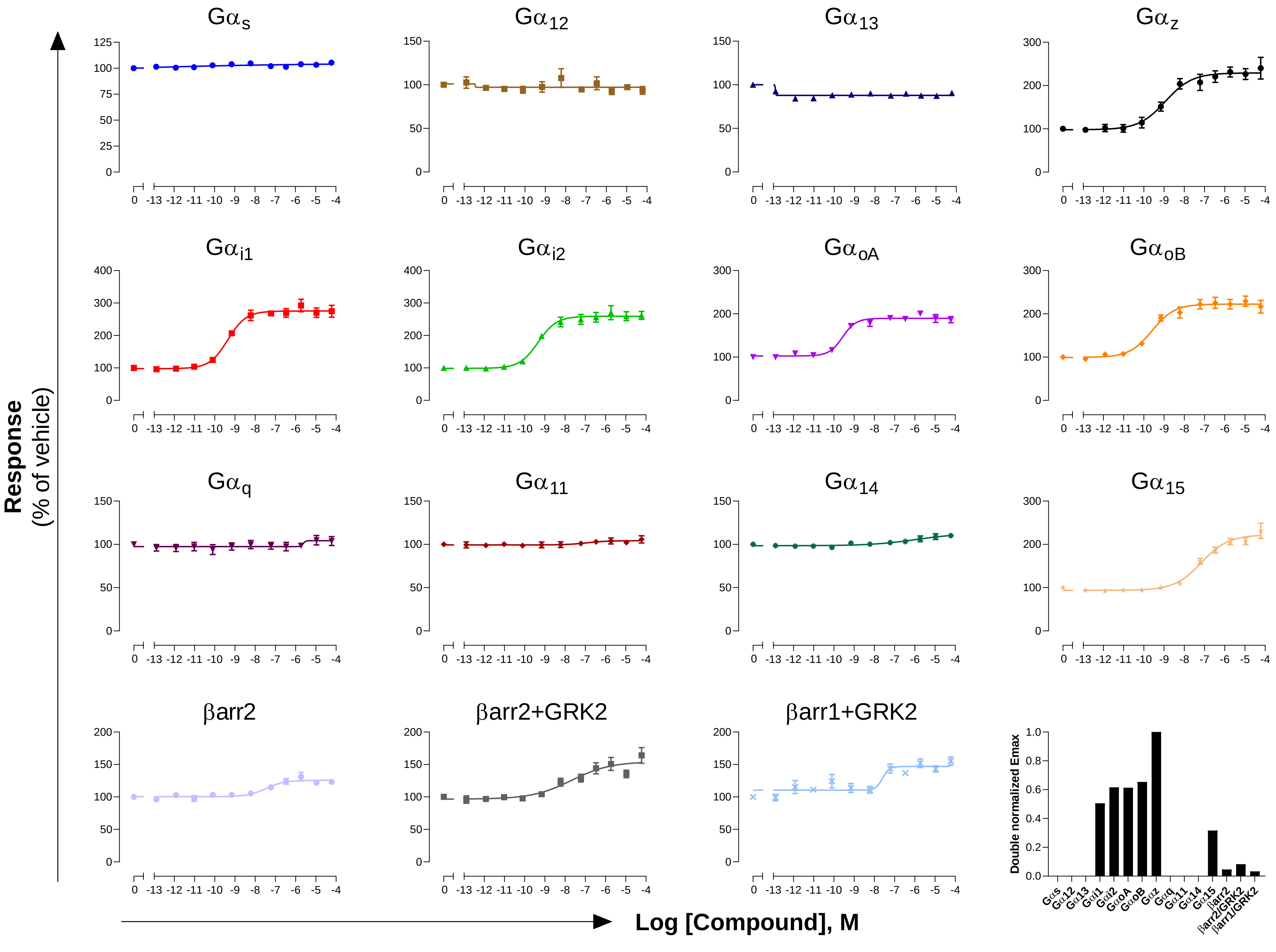

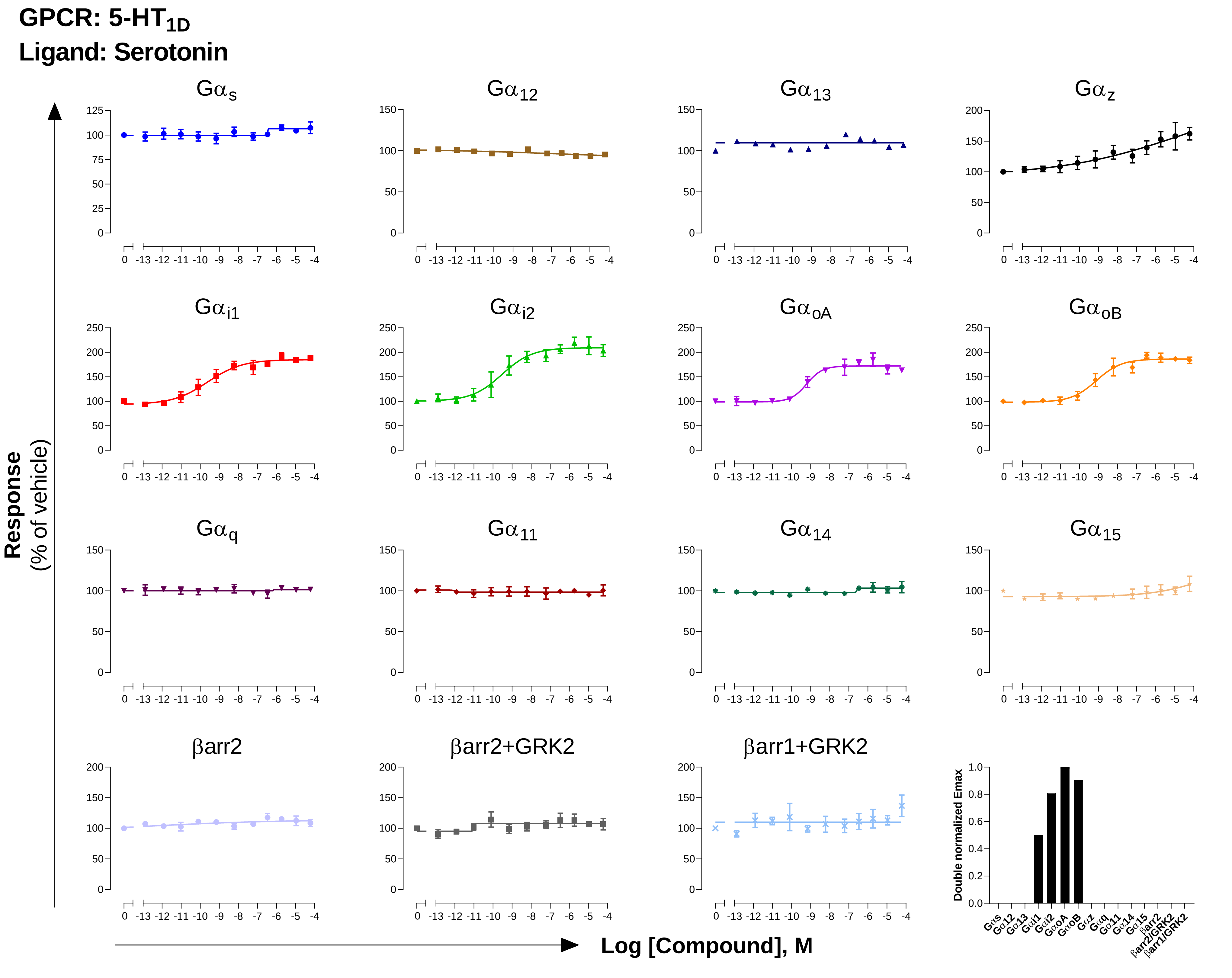

GPCR: 5-HT1D
Ligand: Serotonin
Gαs
Gα12
Gα13
Gαz
Gαi1
Gαi2
GαoA
GαoB
Gαq
Gα11
Gα14
Gα15
βarr2
βarr2+GRK2
βarr1+GRK2
Response
(% of vehicle)
Log [Compound], M
Double normalized Emax

GPCR: 5-HT_{2A}
Ligand: Serotonin

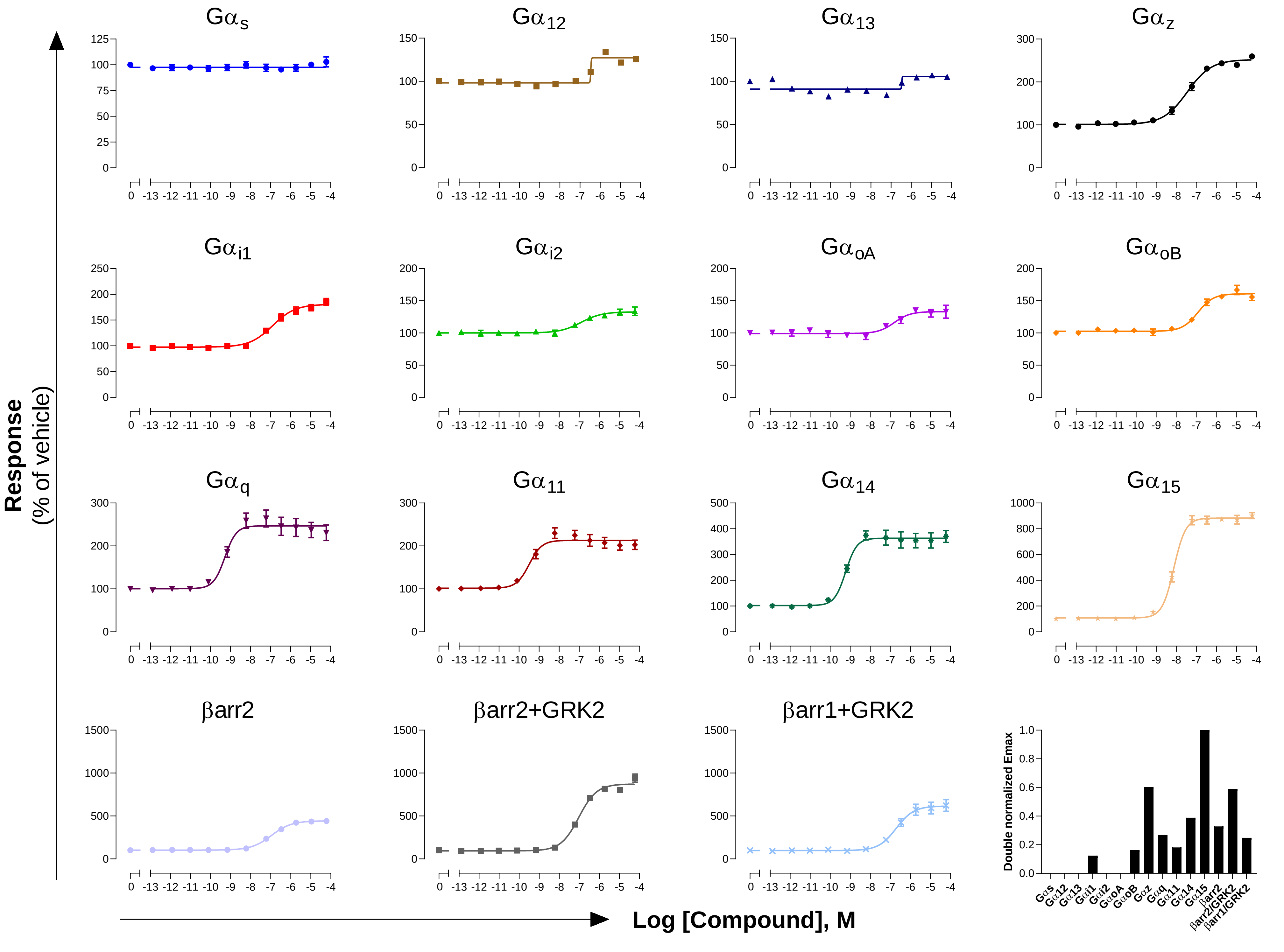

GPCR: 5-HT_{2B}

Ligand: Serotonin

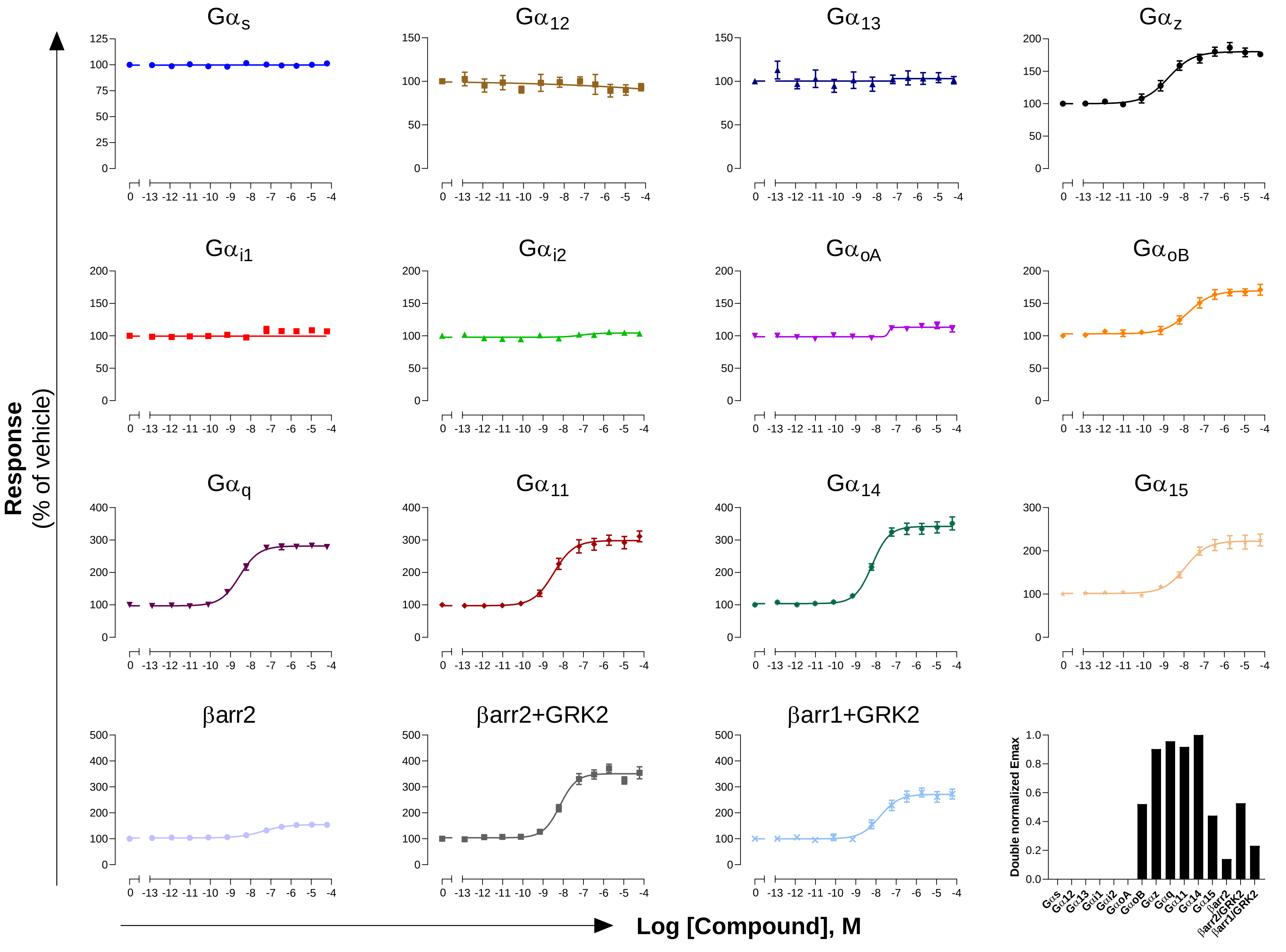

GPCR: 5-HT_{2C}
Ligand: Serotonin

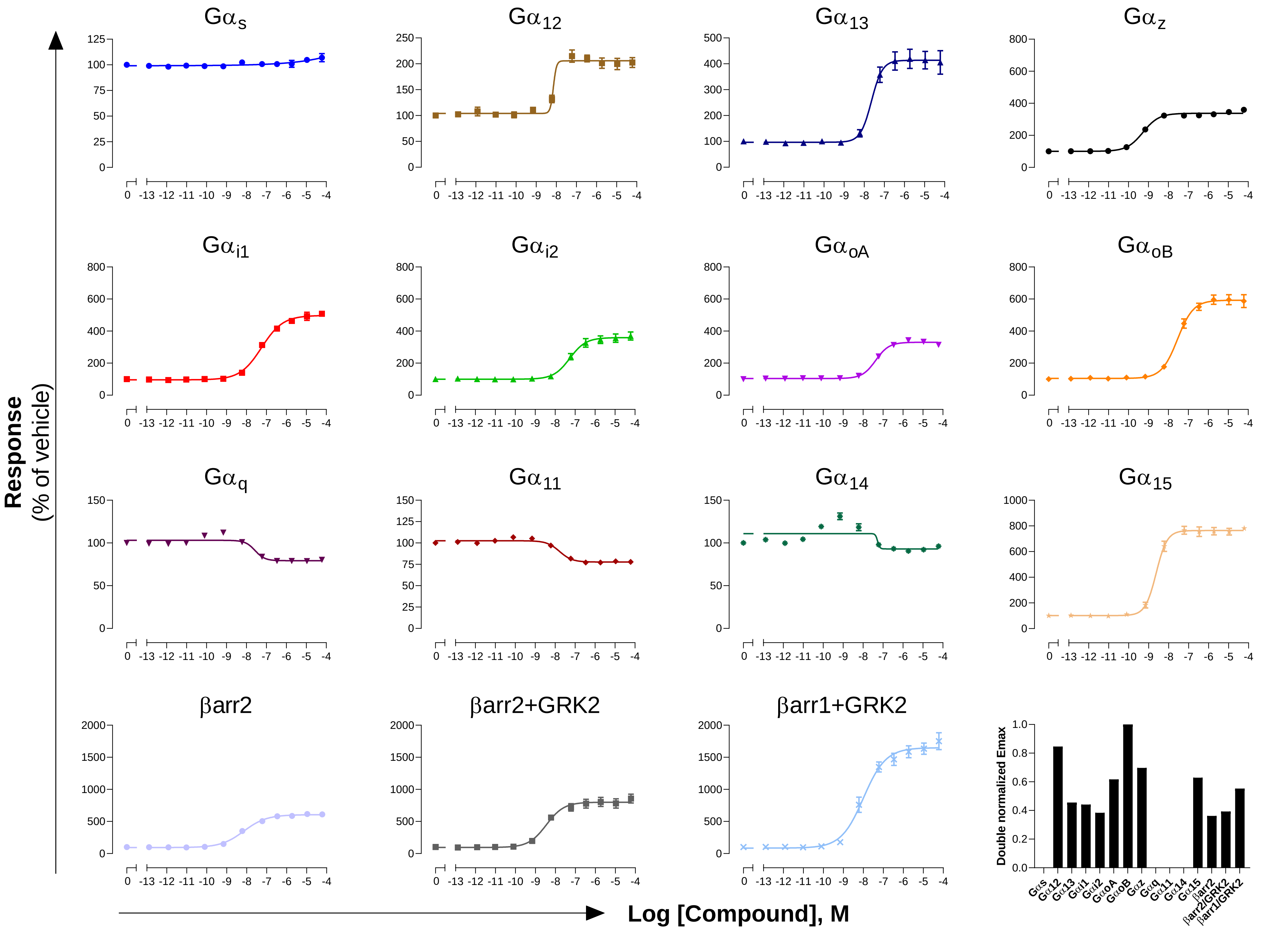

GPCR: α_{1A}AR

Ligand: Noradrenaline

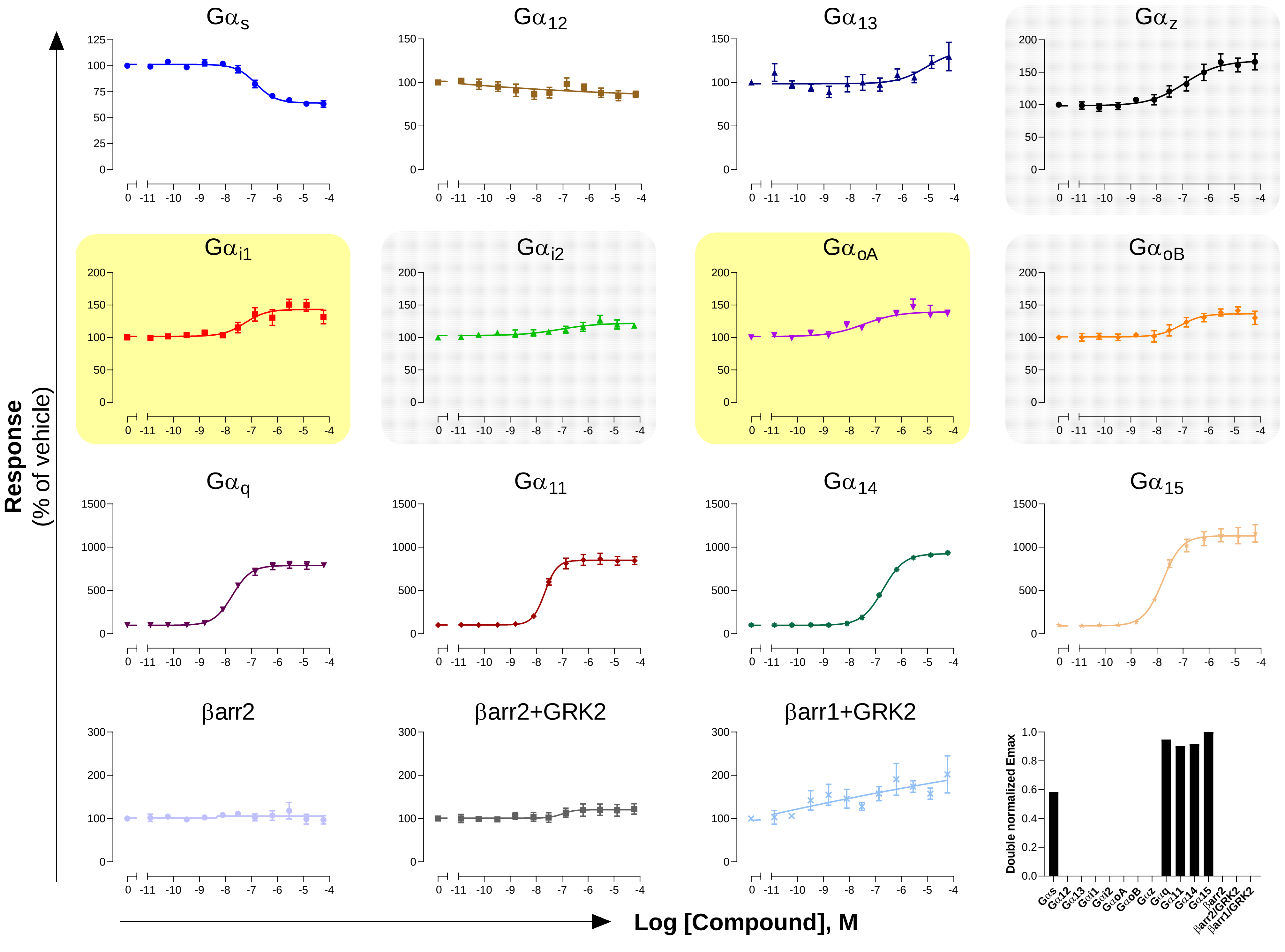

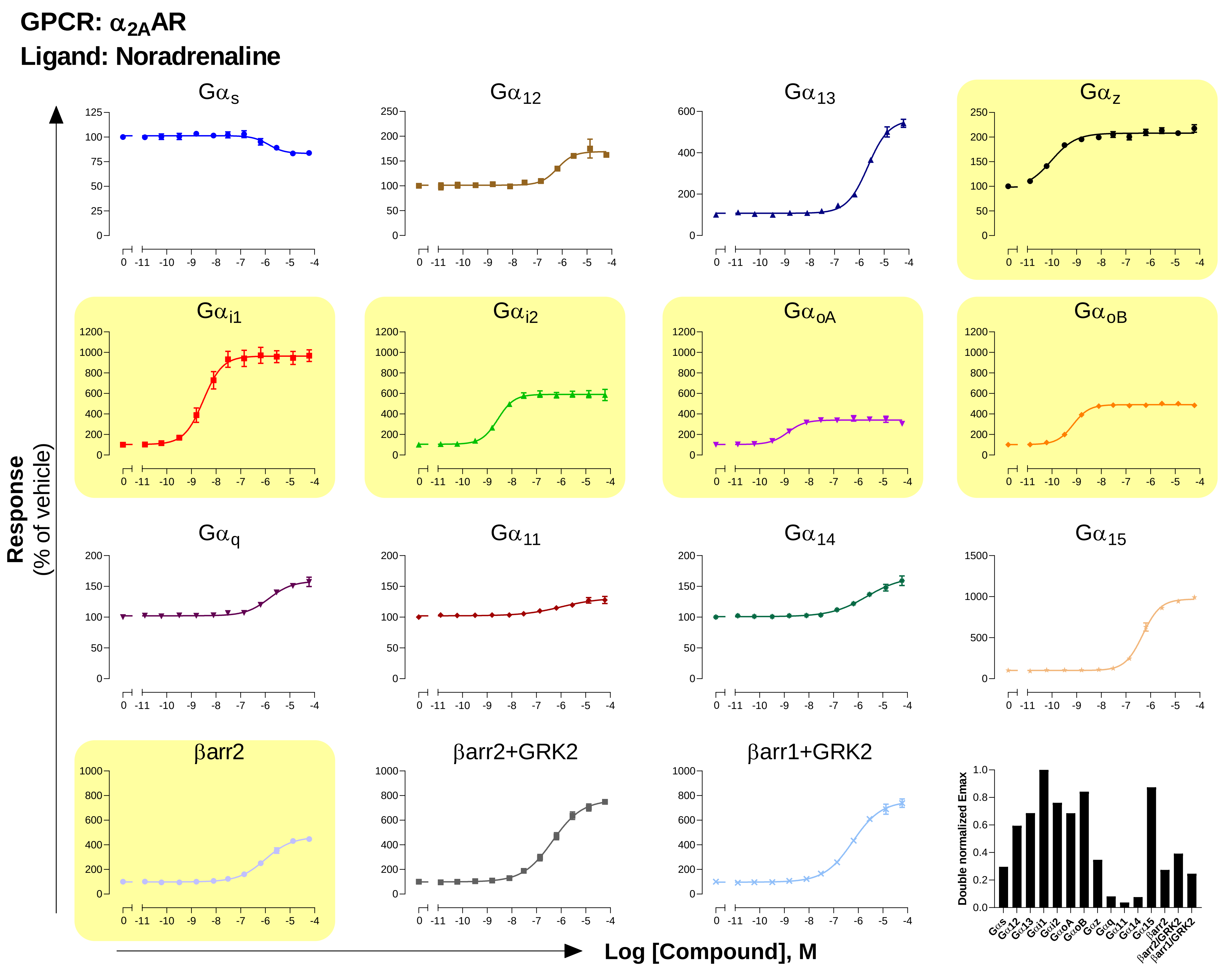

GPCR: α2AAR
Ligand: Noradrenaline
Gαs
Gα12
Gα13
Gαz
Gαi1
Gαi2
GαoA
GαoB
Gαq
Gα11
Gα14
Gα15
βarr2
βarr2+GRK2
βarr1+GRK2
Response
(% of vehicle)
Log [Compound], M
Double normalized Emax

GPCR: α_{2B}AR

Ligand: Noradrenaline

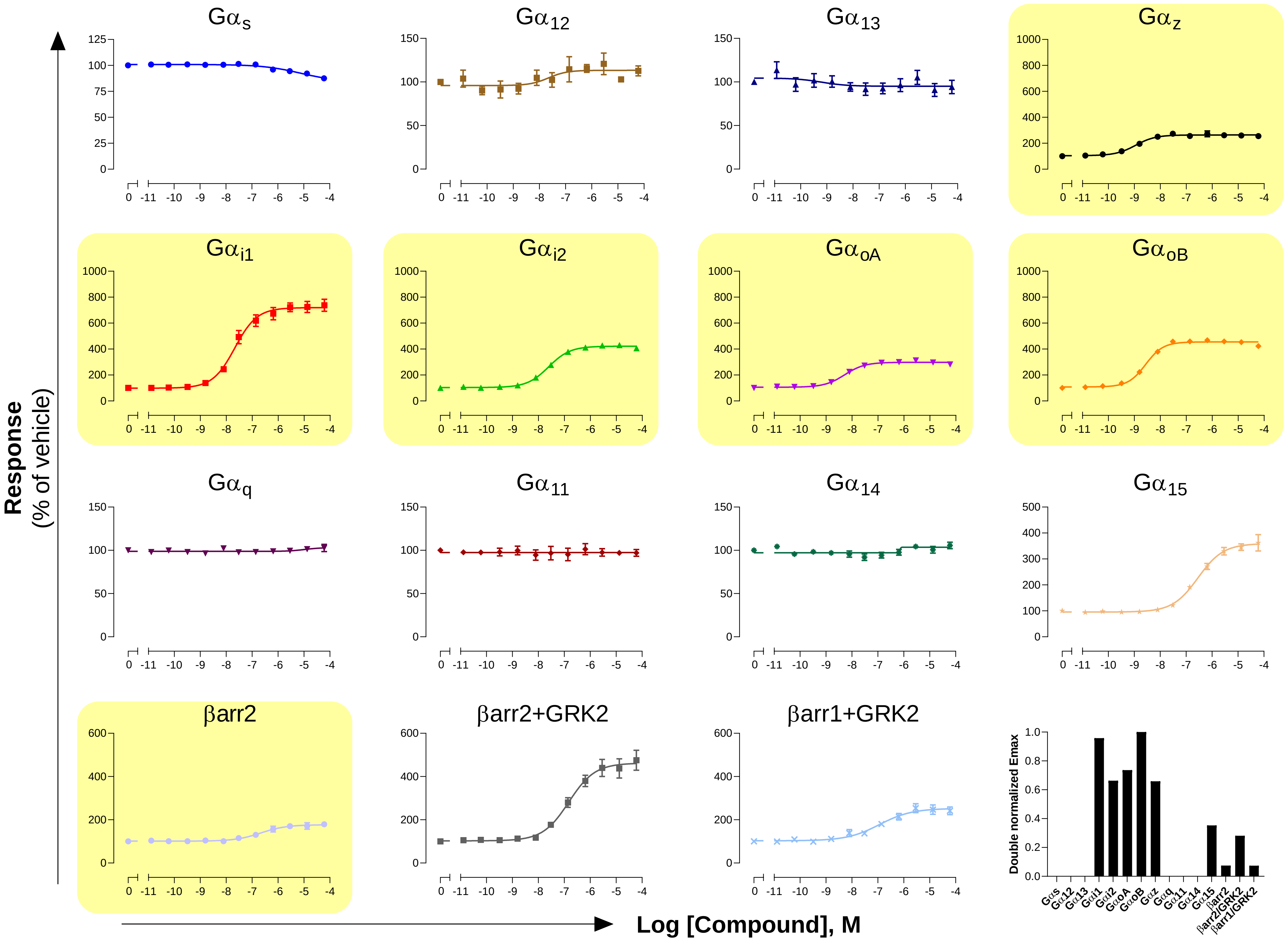

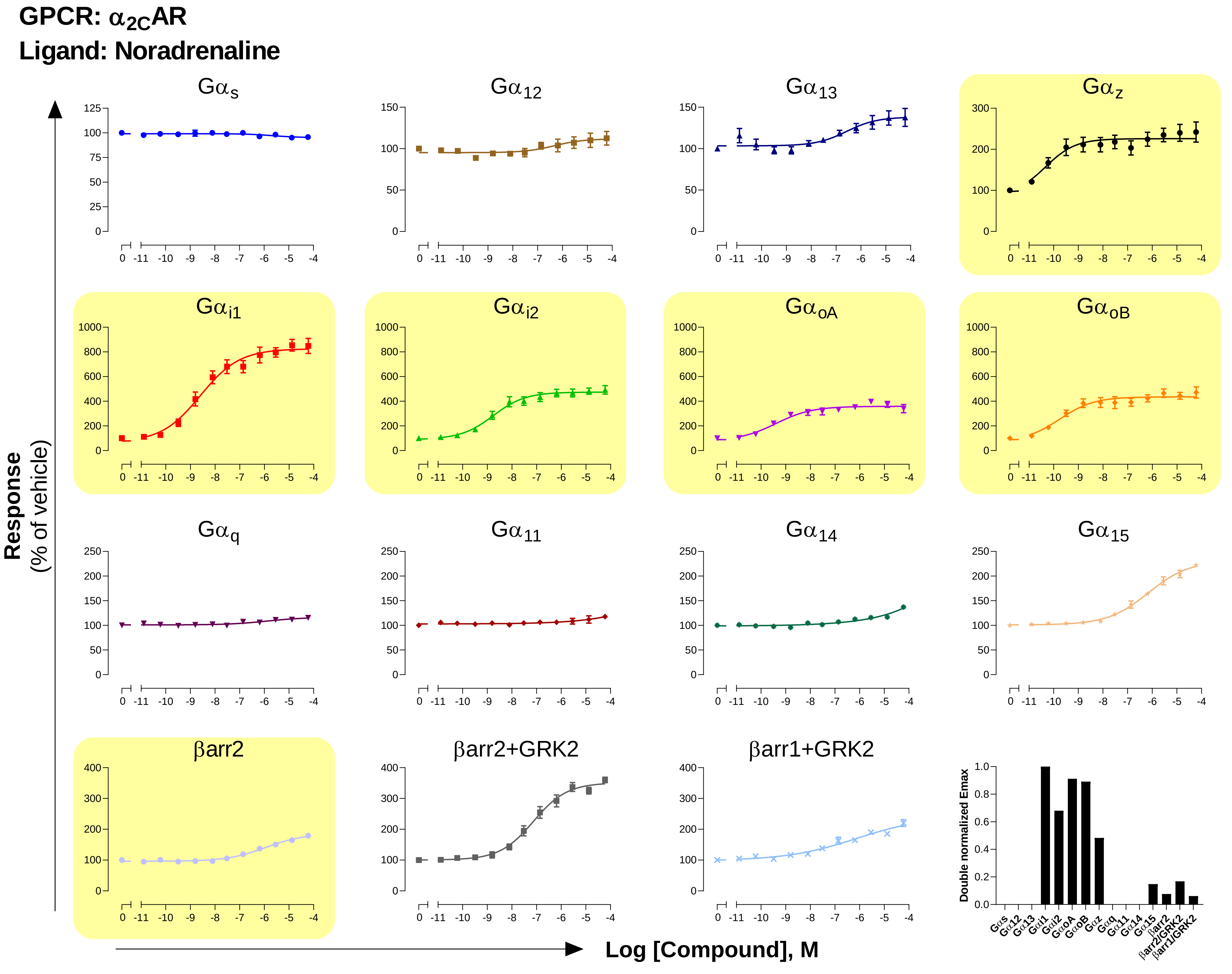

GPCR: α2CAR
Ligand: Noradrenaline
Gαs
Gα12
Gα13
Gαz
Gαi1
Gαi2
GαoA
GαoB
Gαq
Gα11
Gα14
Gα15
βarr2
βarr2+GRK2
βarr1+GRK2
Response
(% of vehicle)
Log [Compound], M
Double normalized Emax
Gαs Gα12 Gα13 Gαi1 Gαi2 GαoA GαoB Gαz Gαq Gα11 Gα14 Gα15 βarr2 βarr2/GRK2 βarr1/GRK2

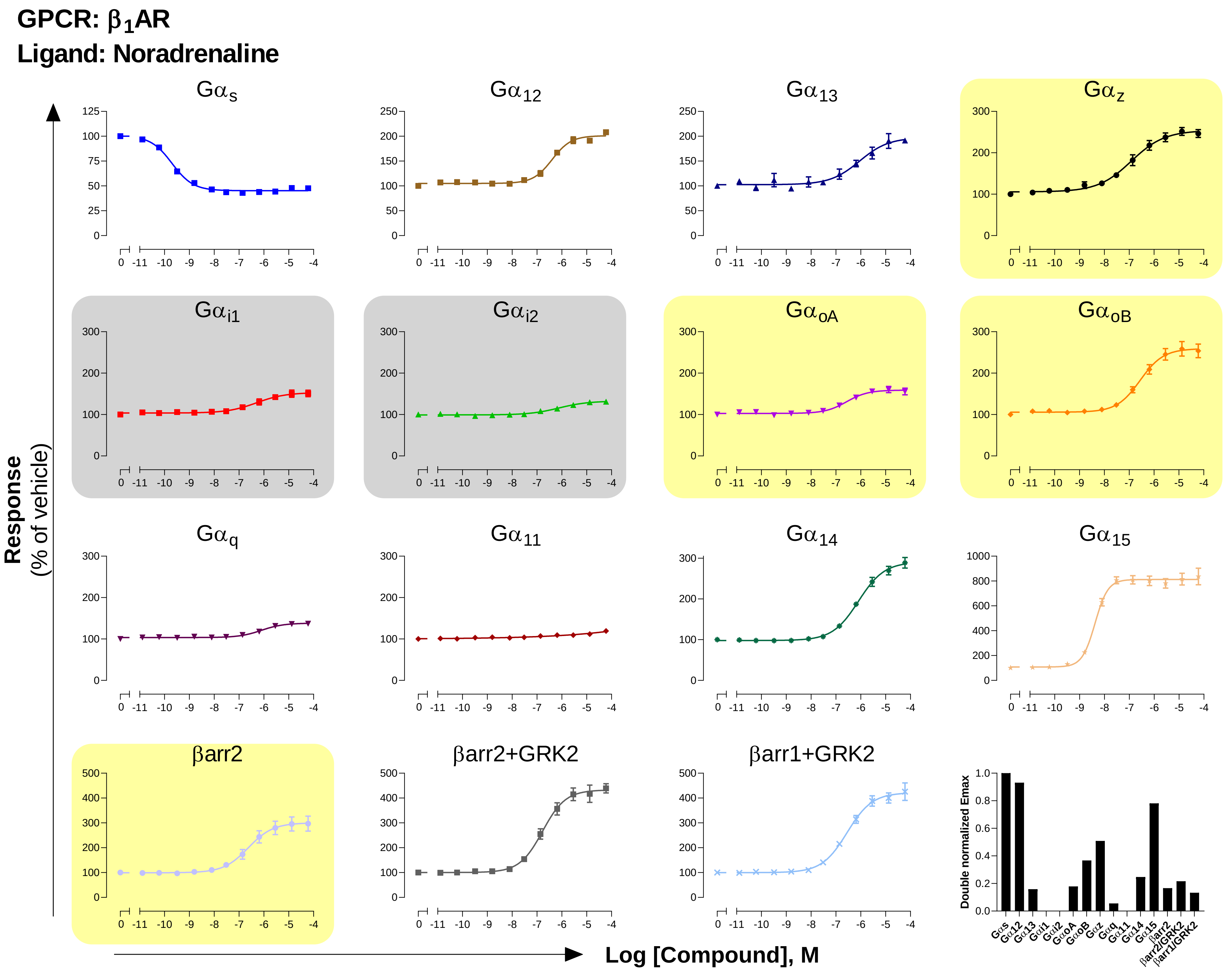
GPCR: β1AR
Ligand: Noradrenaline
Gαs
Gα12
Gα13
Gαz
Gαi1
Gαi2
GαoA
GαoB
Gαq
Gα11
Gα14
Gα15
βarr2
βarr2+GRK2
βarr1+GRK2
Response
(% of vehicle)
Log [Compound], M
Double normalized Emax

GPCR: β$_2$AR

Ligand: Noradrenaline

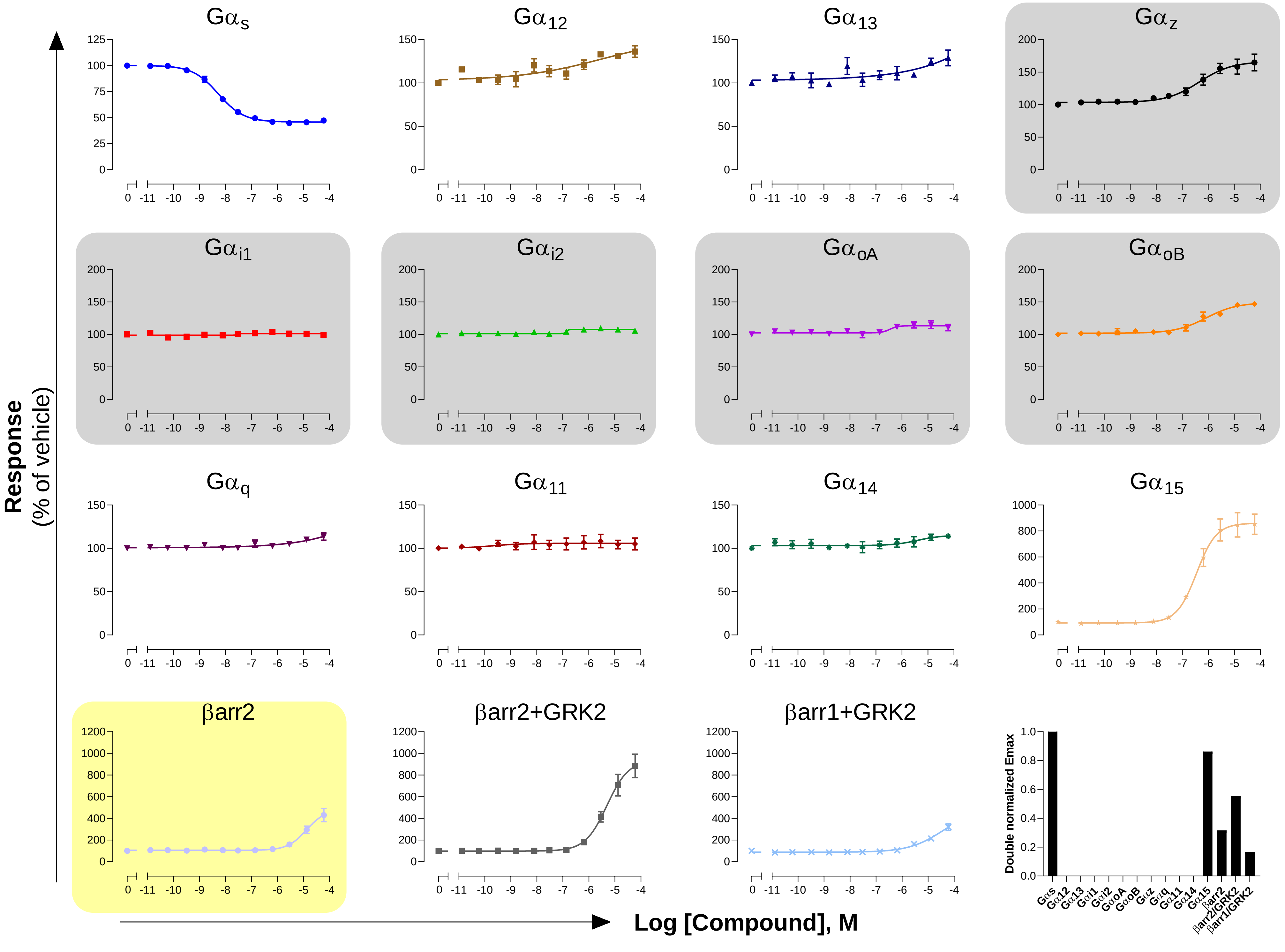

GPCR: A1
Ligand: Adenosine

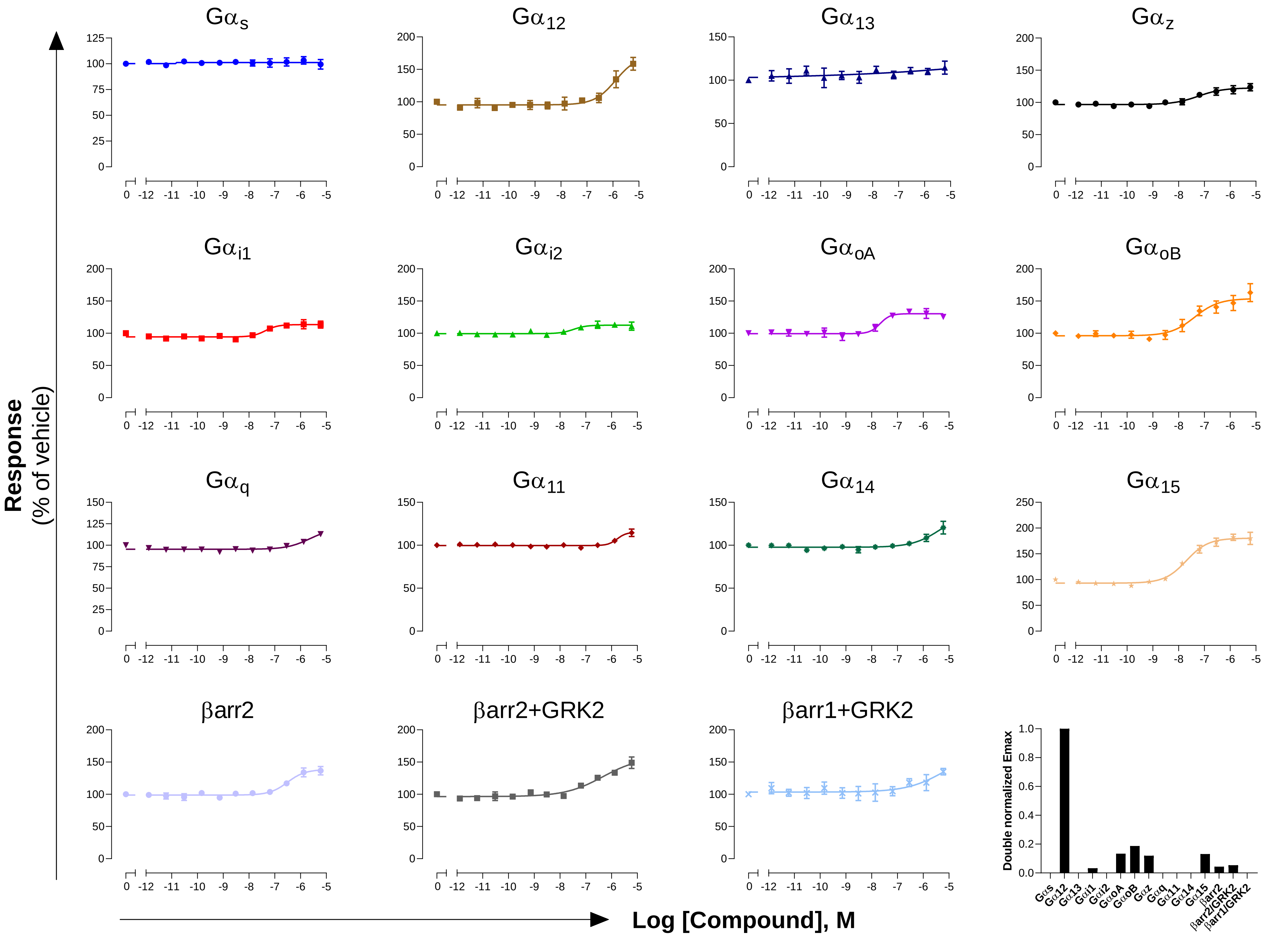

GPCR: A_{2A}

Ligand: Adenosine

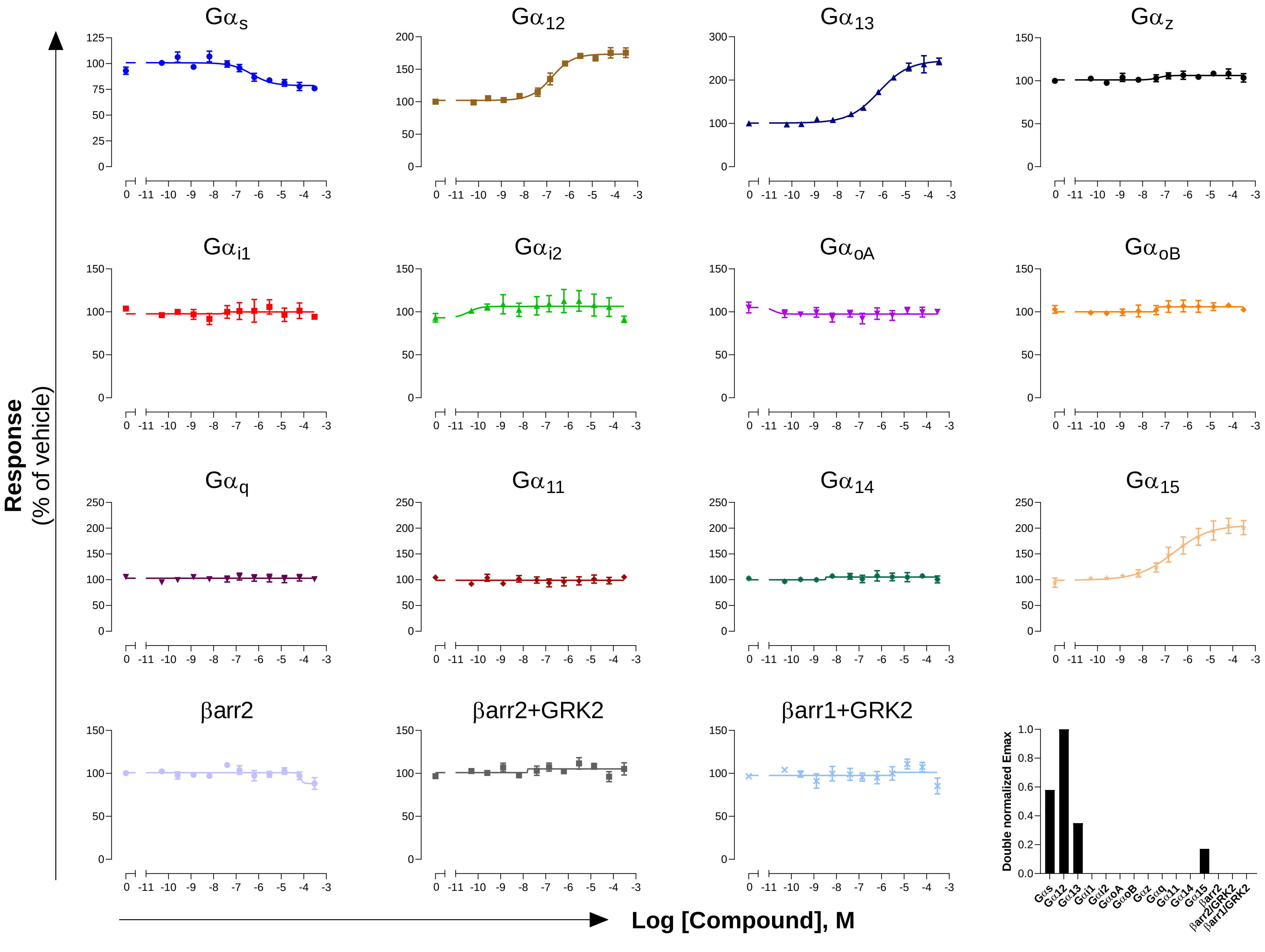

GPCR: A_{2B}
Ligand: Adenosine

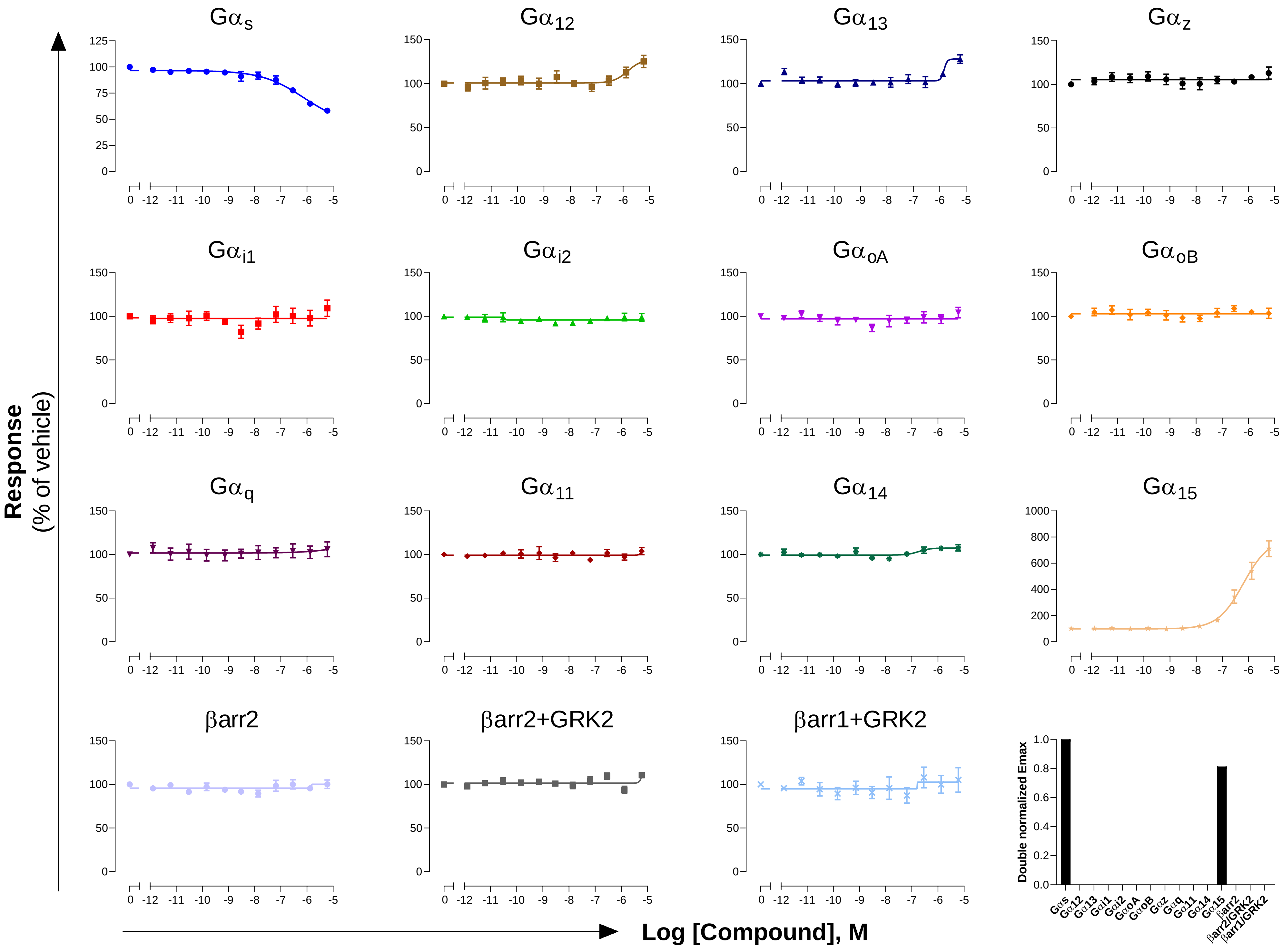

GPCR: A_3

Ligand: Adenosine

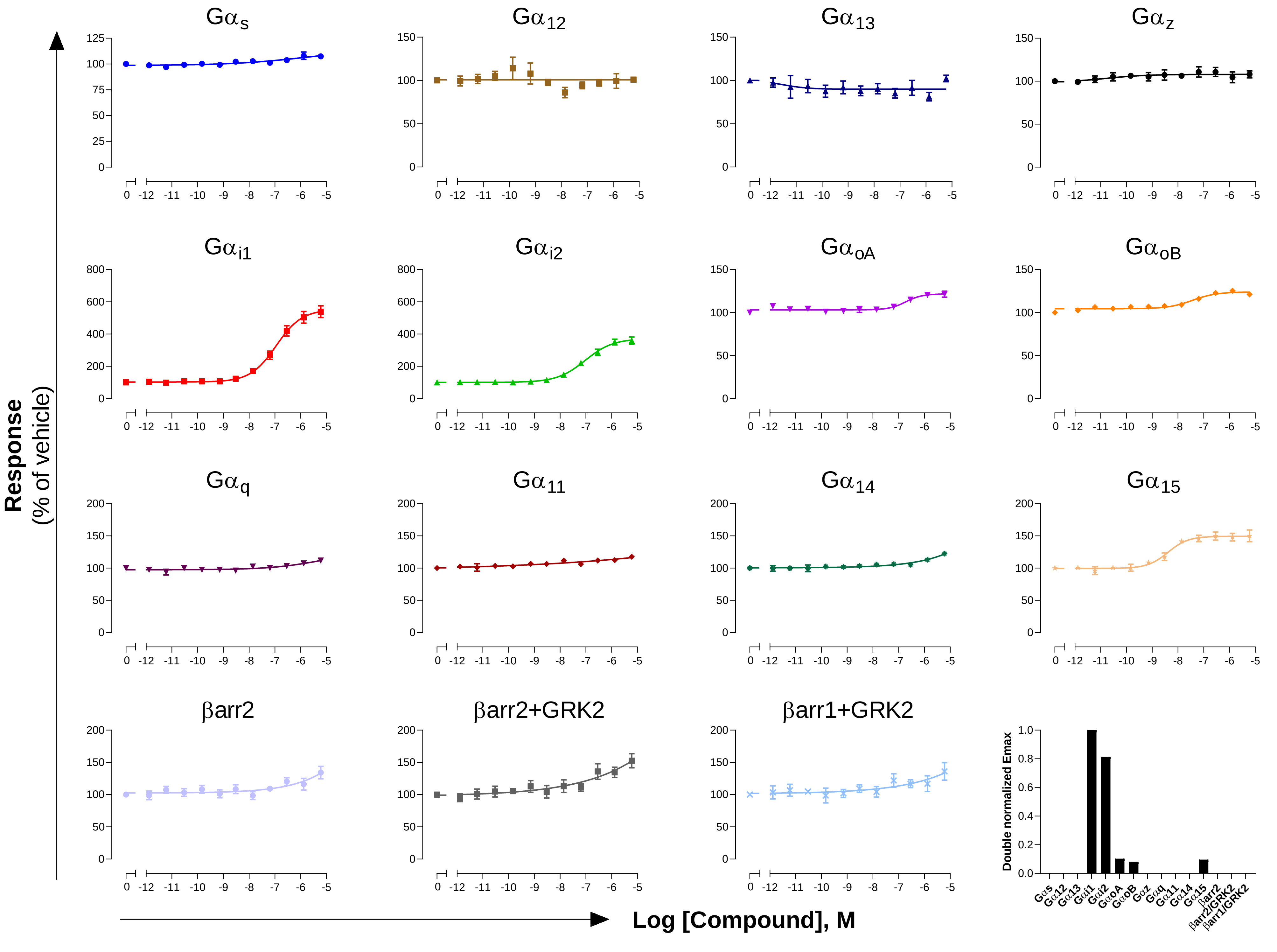

GPCR: AMY$_3$

Ligand: Amylin

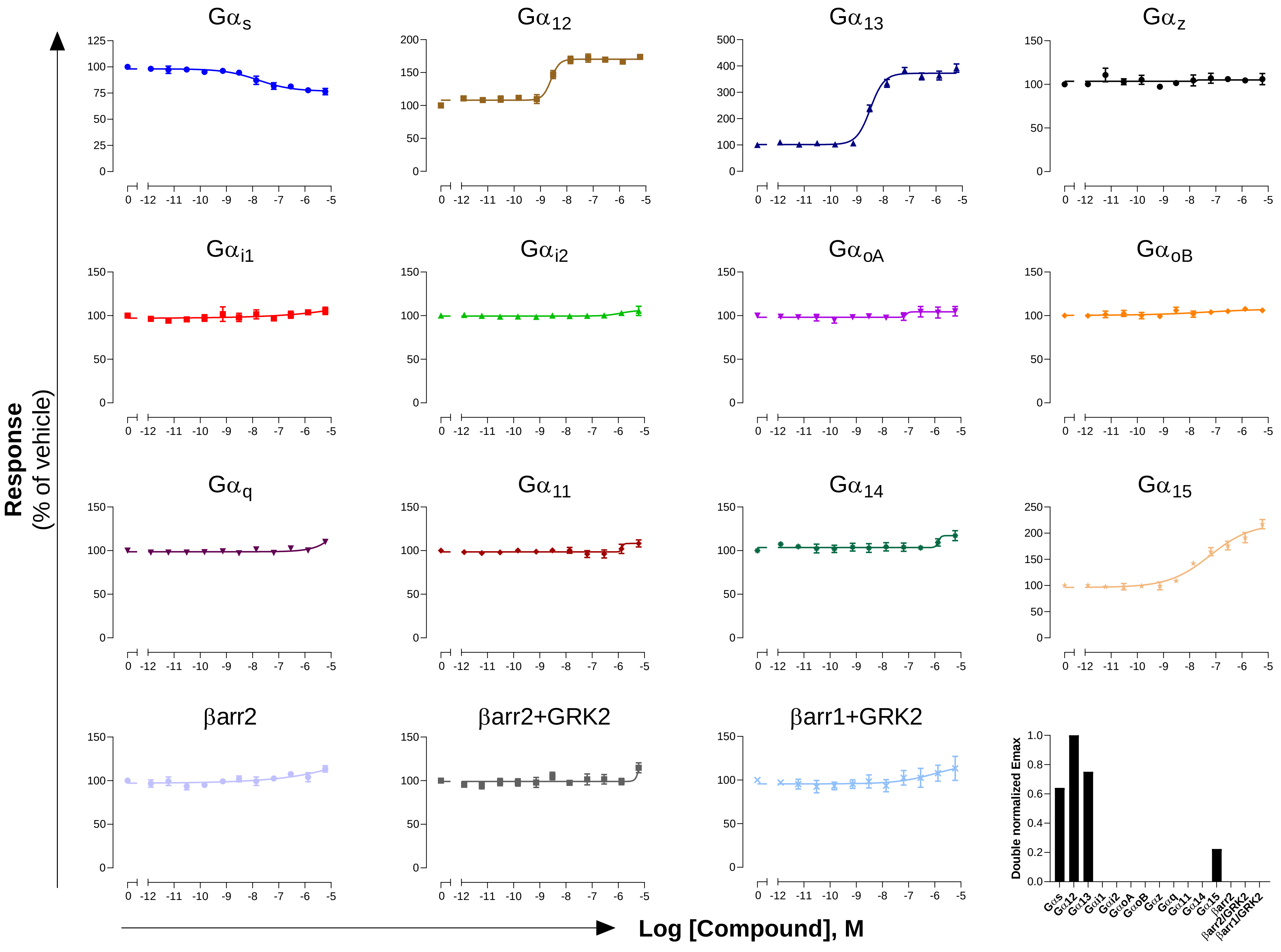

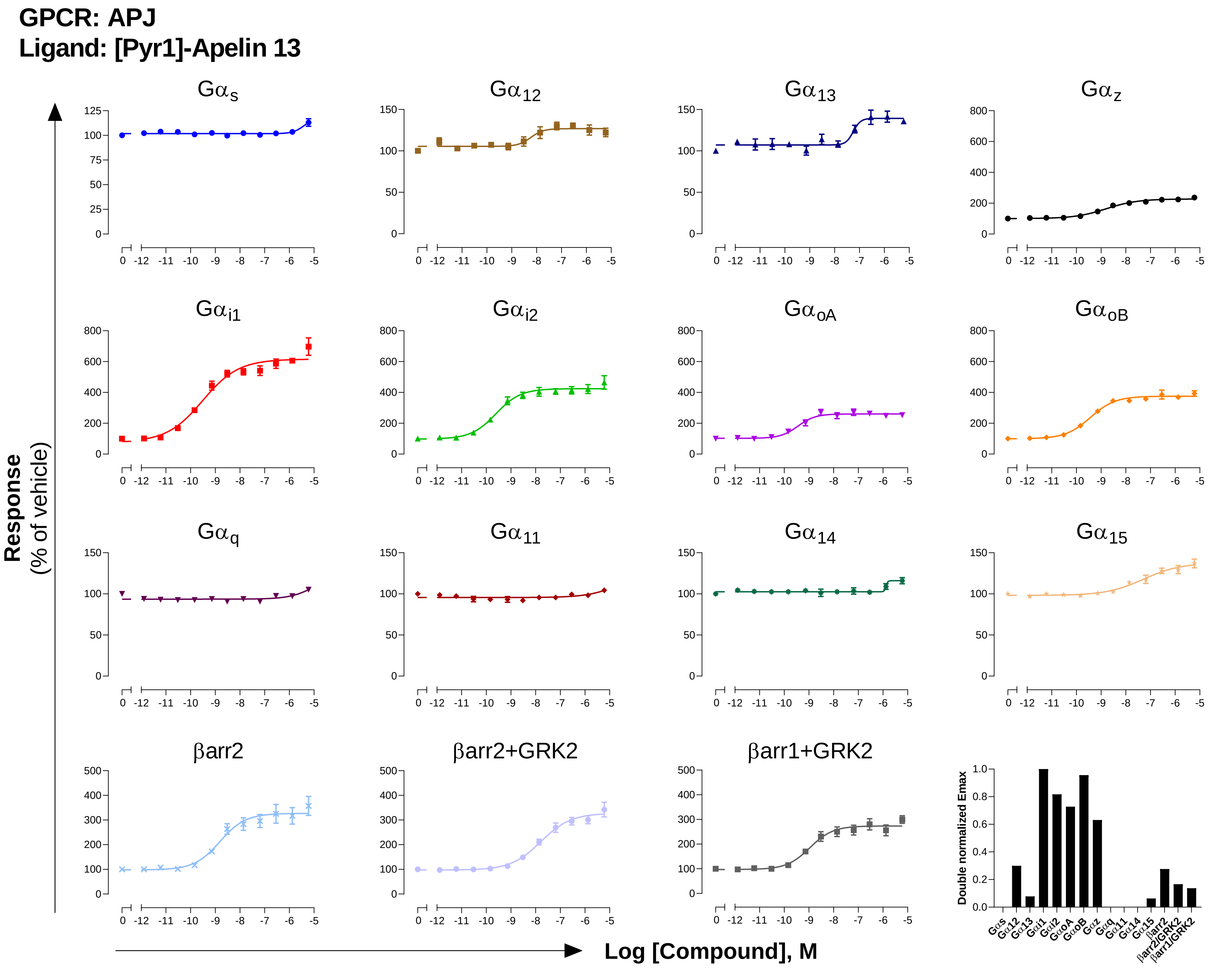
GPCR: APJ
Ligand: [Pyr1]-Apelin 13
Gαs
Gα12
Gα13
Gαz
Gαi1
Gαi2
GαoA
GαoB
Gαq
Gα11
Gα14
Gα15
βarr2
βarr2+GRK2
βarr1+GRK2
Response
(% of vehicle)
Log [Compound], M
Double normalized Emax

GPCR: AT_1

Ligand: Angiotensin II

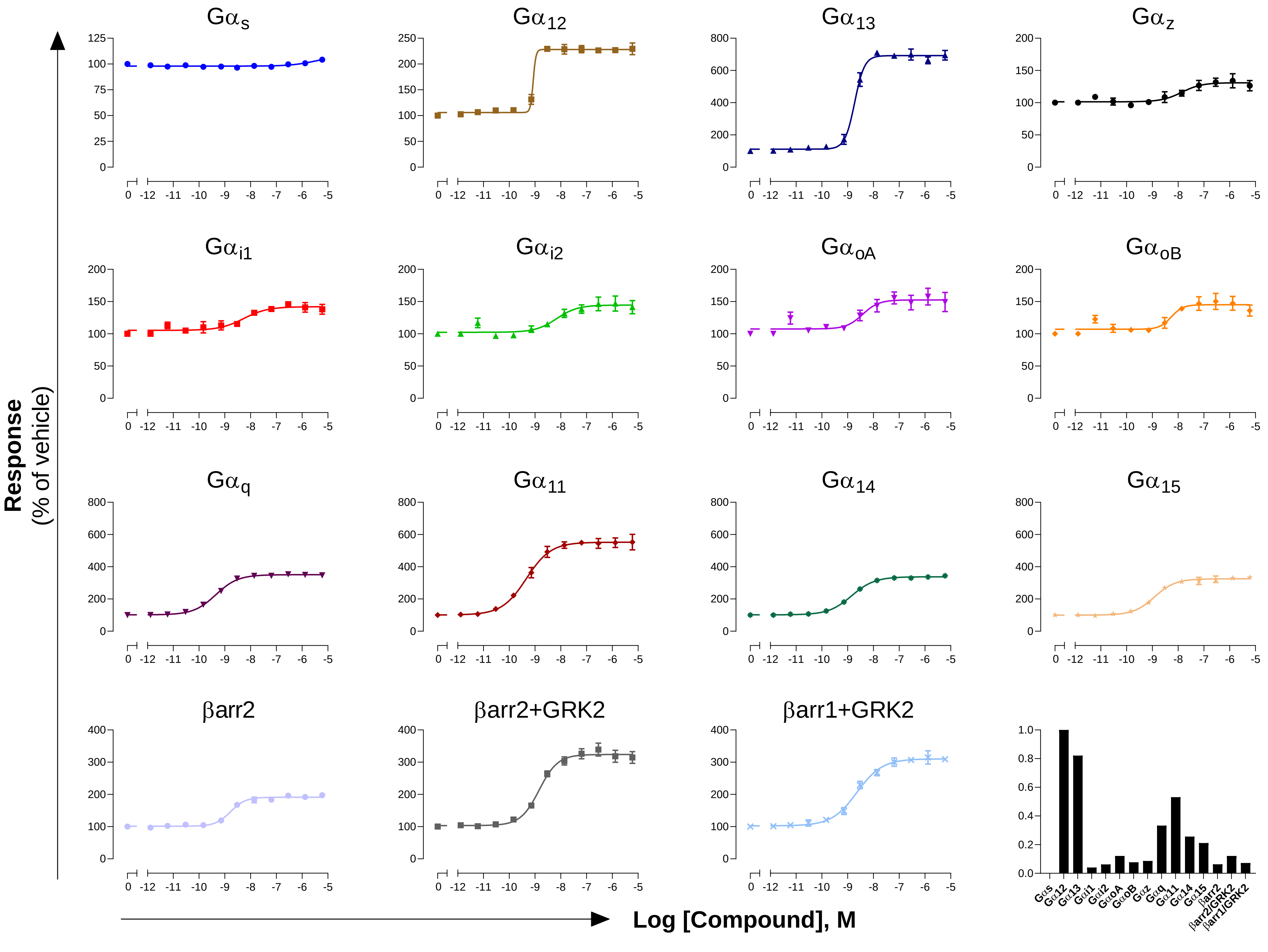

GPCR: B$_1$

Ligand: Kallidin

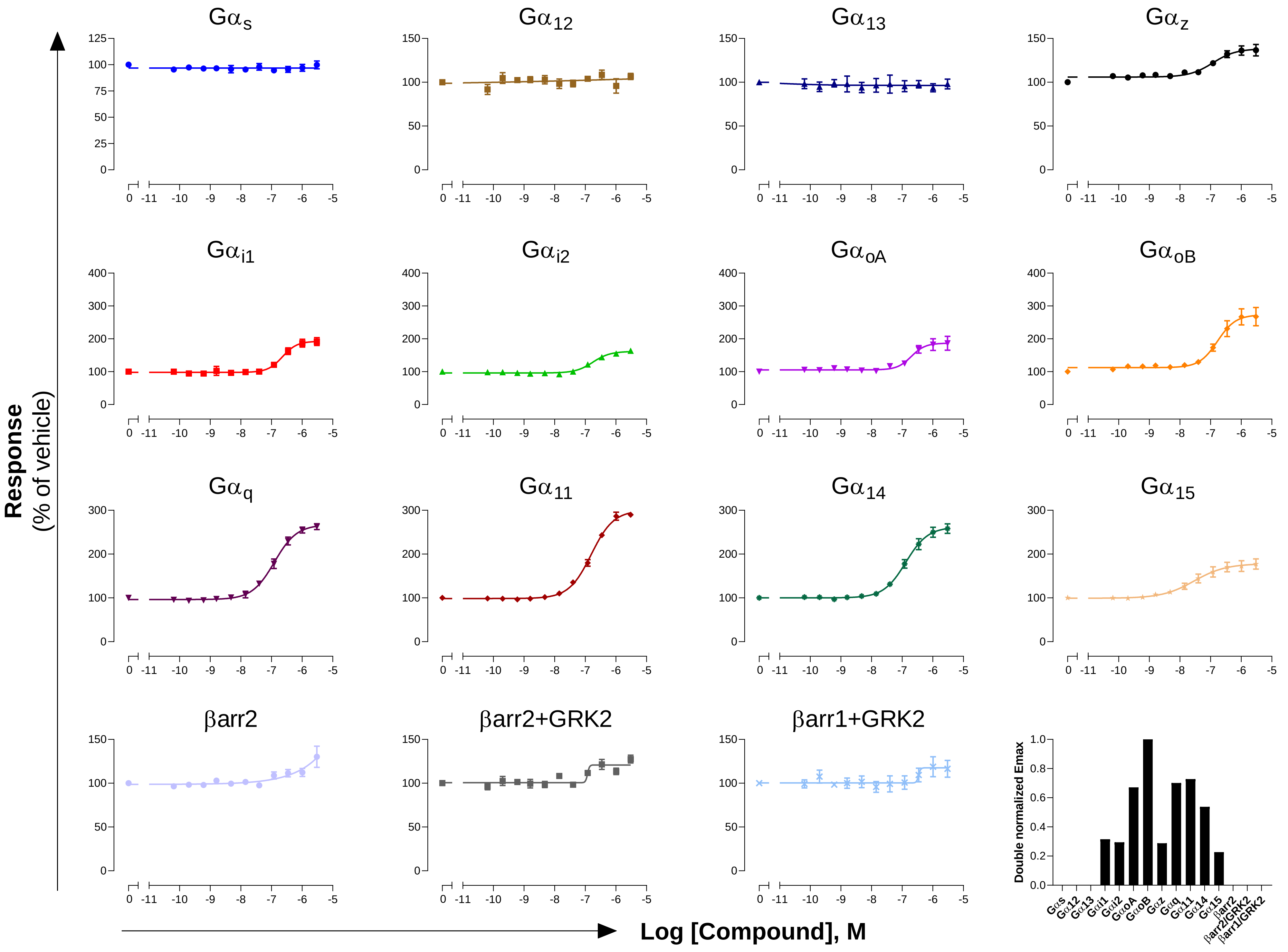

GPCR: B_2
Ligand: Kallidin

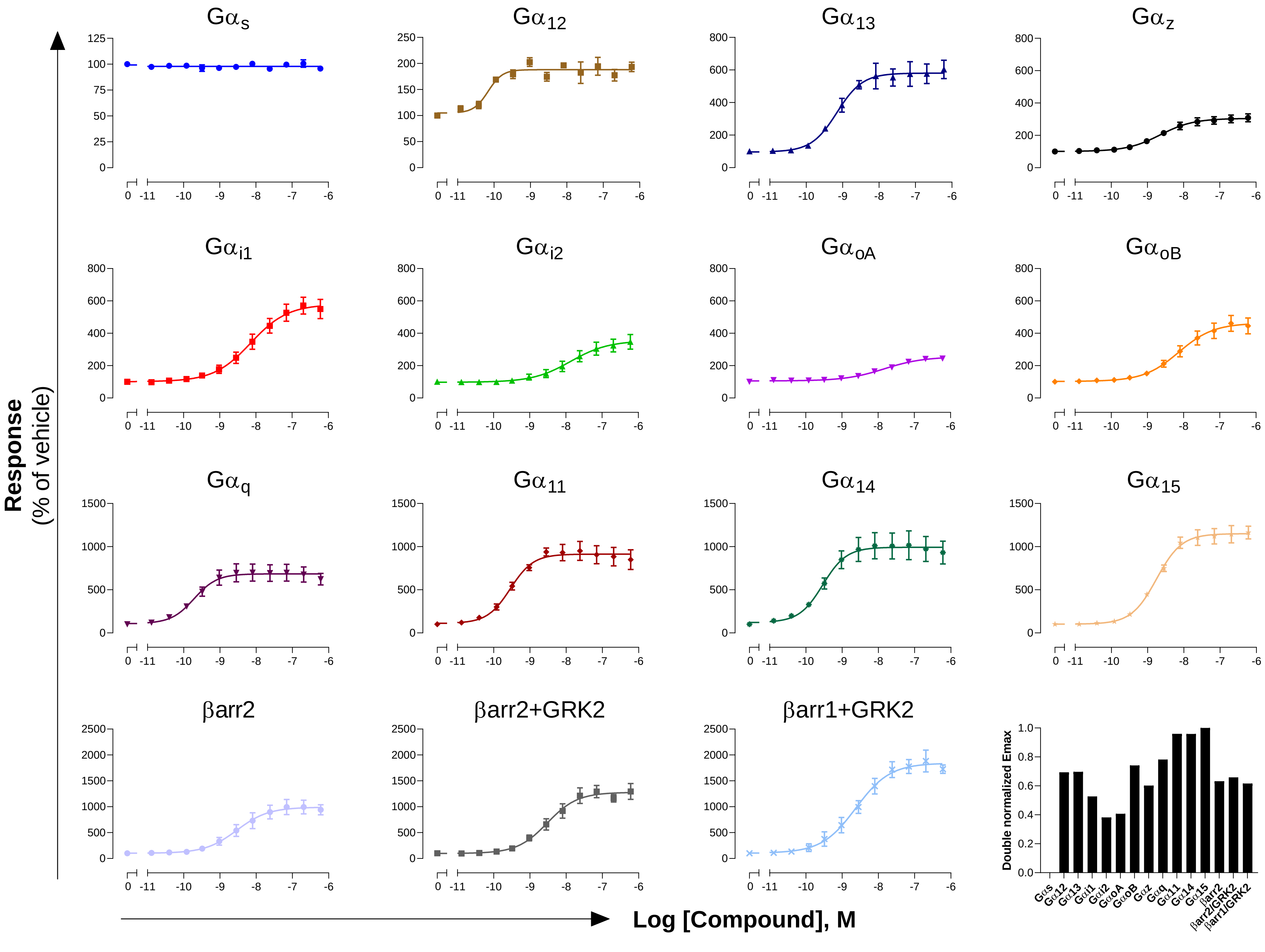

GPCR: BLT$_1$
Ligand: LTB4

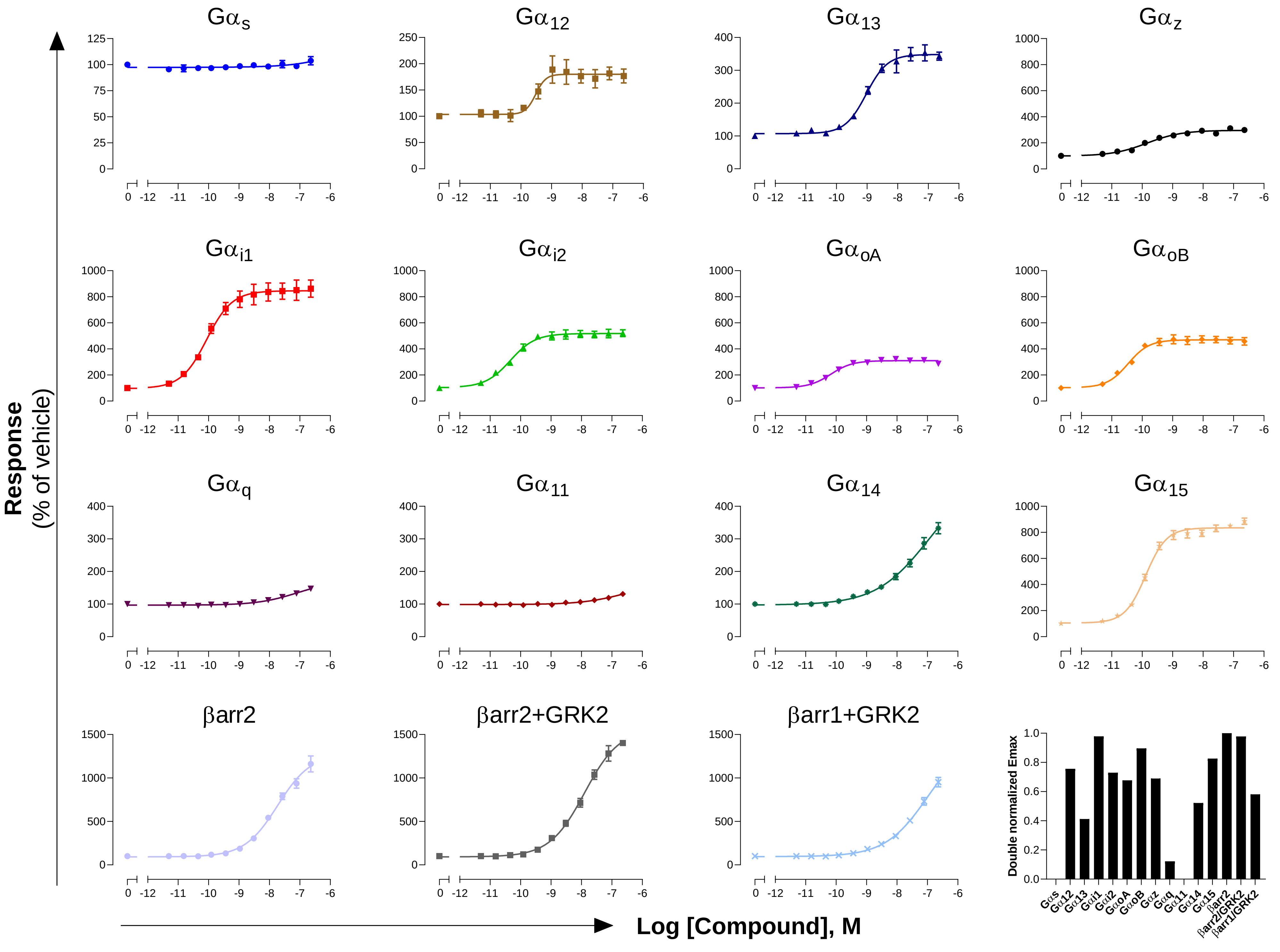

GPCR: BLT_2
Ligand: LTB4

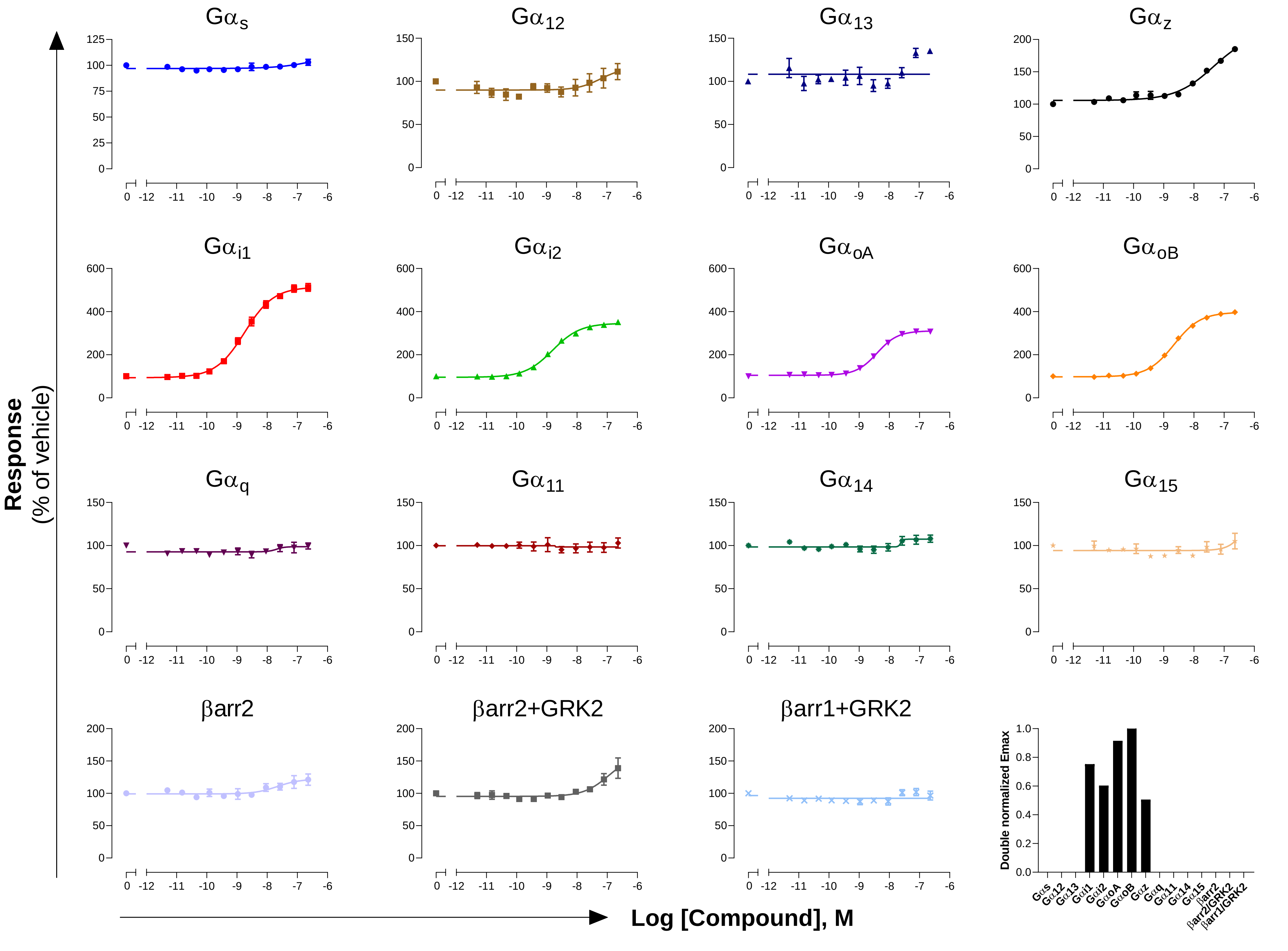

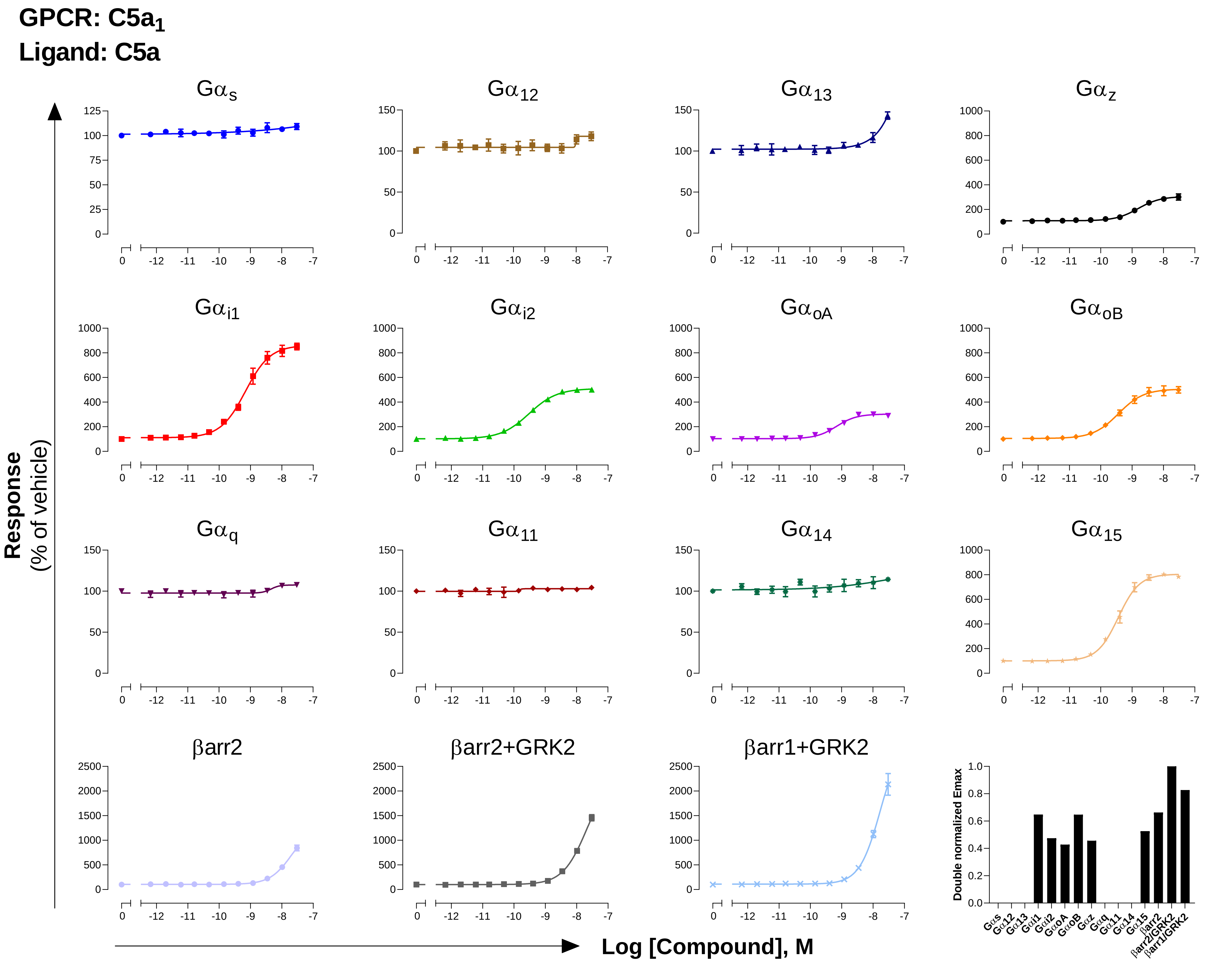
GPCR: C5a1
Ligand: C5a
Gαs
Gα12
Gα13
Gαz
Gαi1
Gαi2
GαoA
GαoB
Gαq
Gα11
Gα14
Gα15
βarr2
βarr2+GRK2
βarr1+GRK2
Response
(% of vehicle)
Log [Compound], M
Double normalized Emax

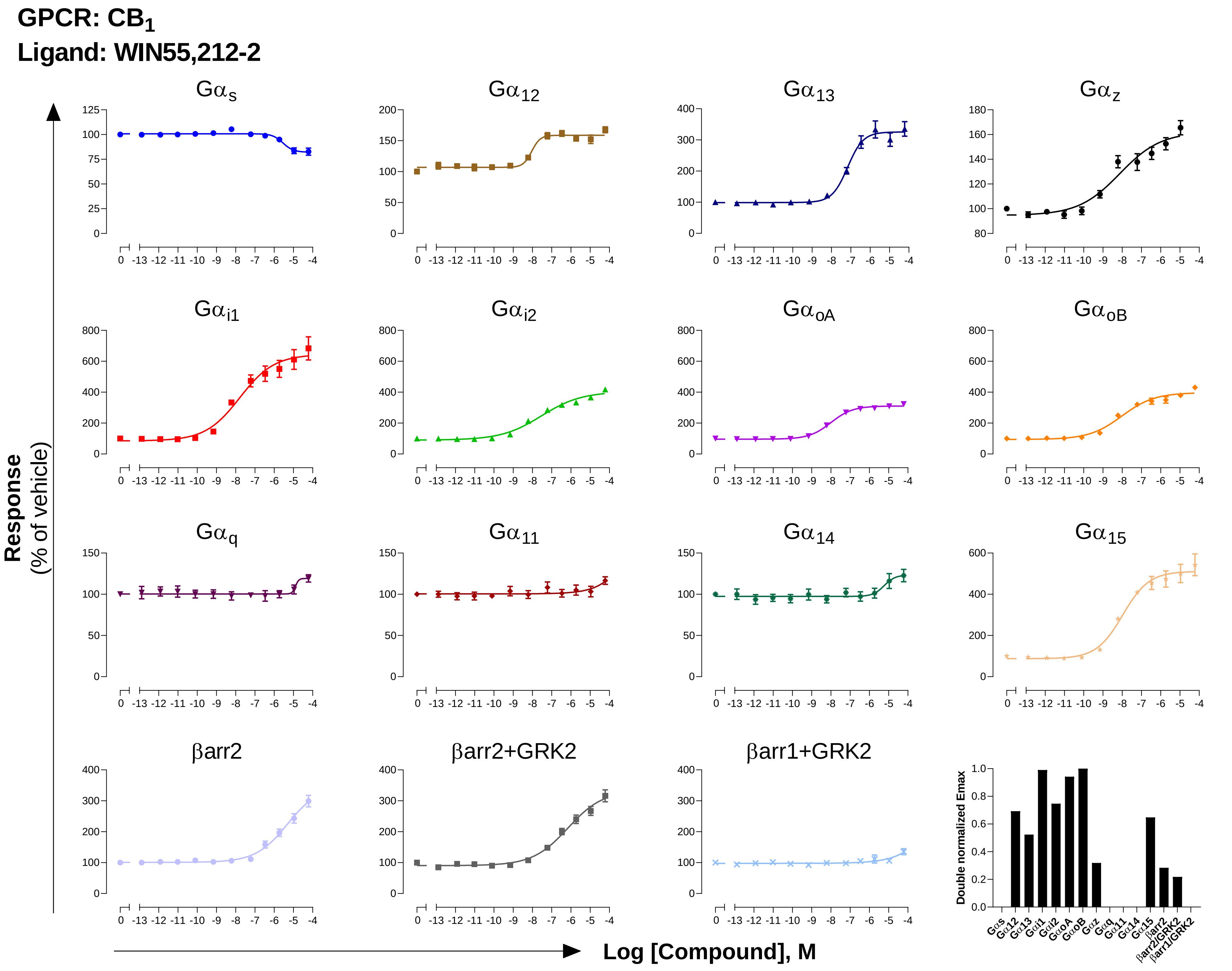
GPCR: CB1
Ligand: WIN55,212-2
Gαs
Gα12
Gα13
Gαz
Gαi1
Gαi2
GαoA
GαoB
Gαq
Gα11
Gα14
Gα15
βarr2
βarr2+GRK2
βarr1+GRK2
Response
(% of vehicle)
Log [Compound], M
Double normalized Emax

GPCR: CB_2

Ligand: WIN55,212-2

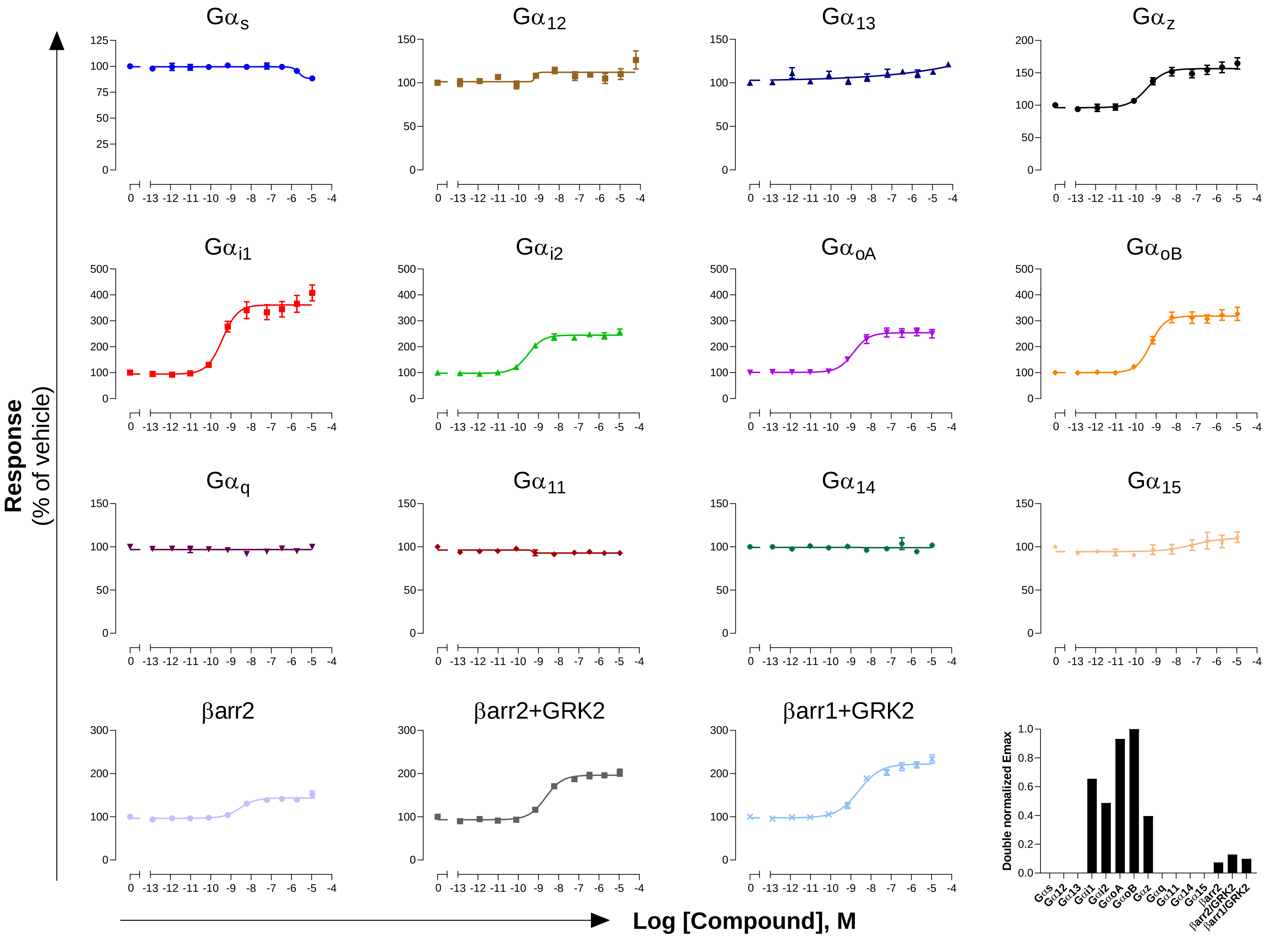

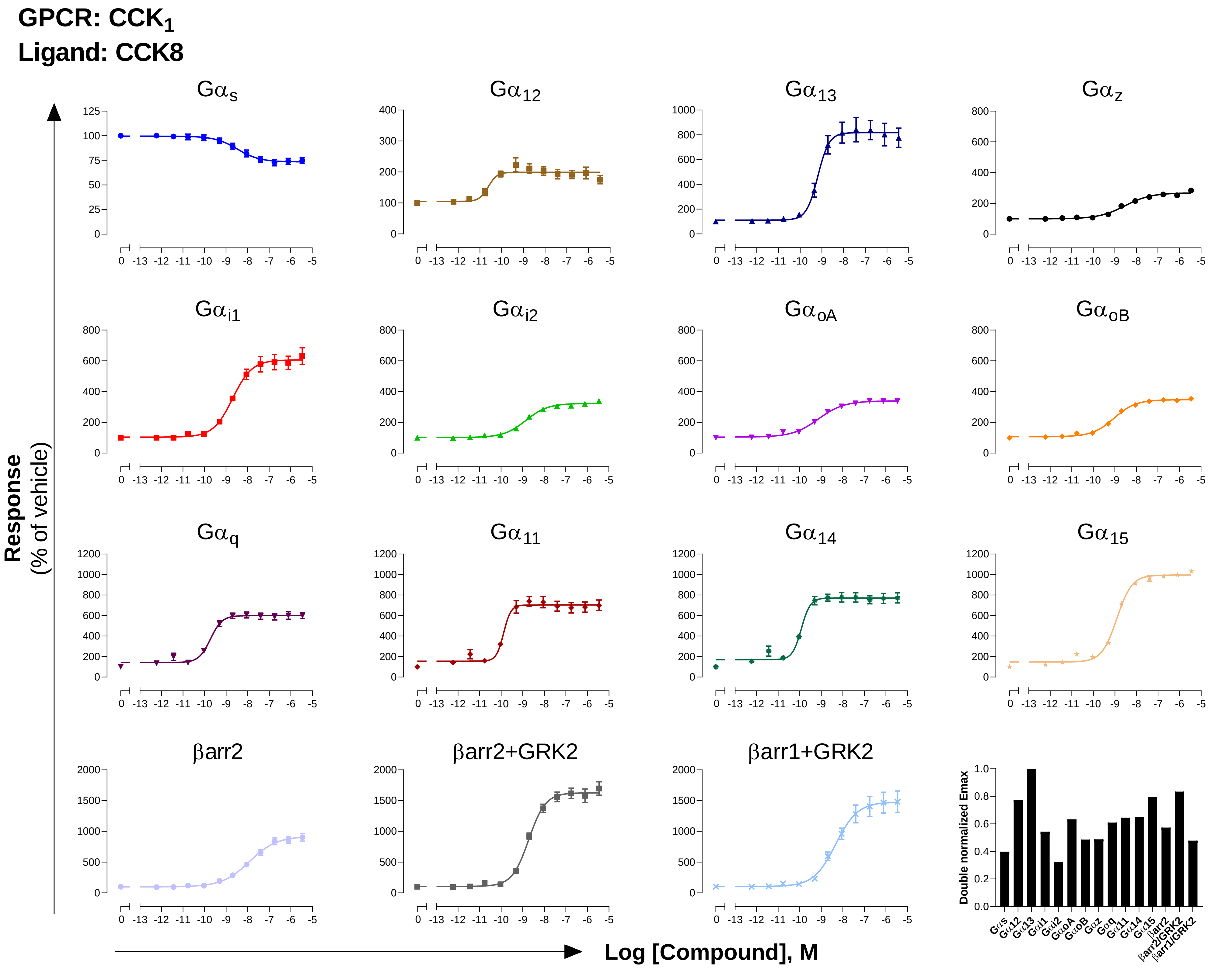

GPCR: CCK_1
Ligand: CCK8
$G\alpha_s$
$G\alpha_{12}$
$G\alpha_{13}$
$G\alpha_z$
$G\alpha_{i1}$
$G\alpha_{i2}$
$G\alpha_{oA}$
$G\alpha_{oB}$
$G\alpha_q$
$G\alpha_{11}$
$G\alpha_{14}$
$G\alpha_{15}$
βarr2
βarr2+GRK2
βarr1+GRK2
Response
(% of vehicle)
Log [Compound], M
Double normalized Emax
Gαs
Gα12
Gα13
Gαi1
Gαi2
GαoA
GαoB
Gαz
Gαq
Gα11
Gα14
Gα15
βarr2
βarr2/GRK2
βarr1/GRK2

GPCR: CCR5
Ligand: CCL3

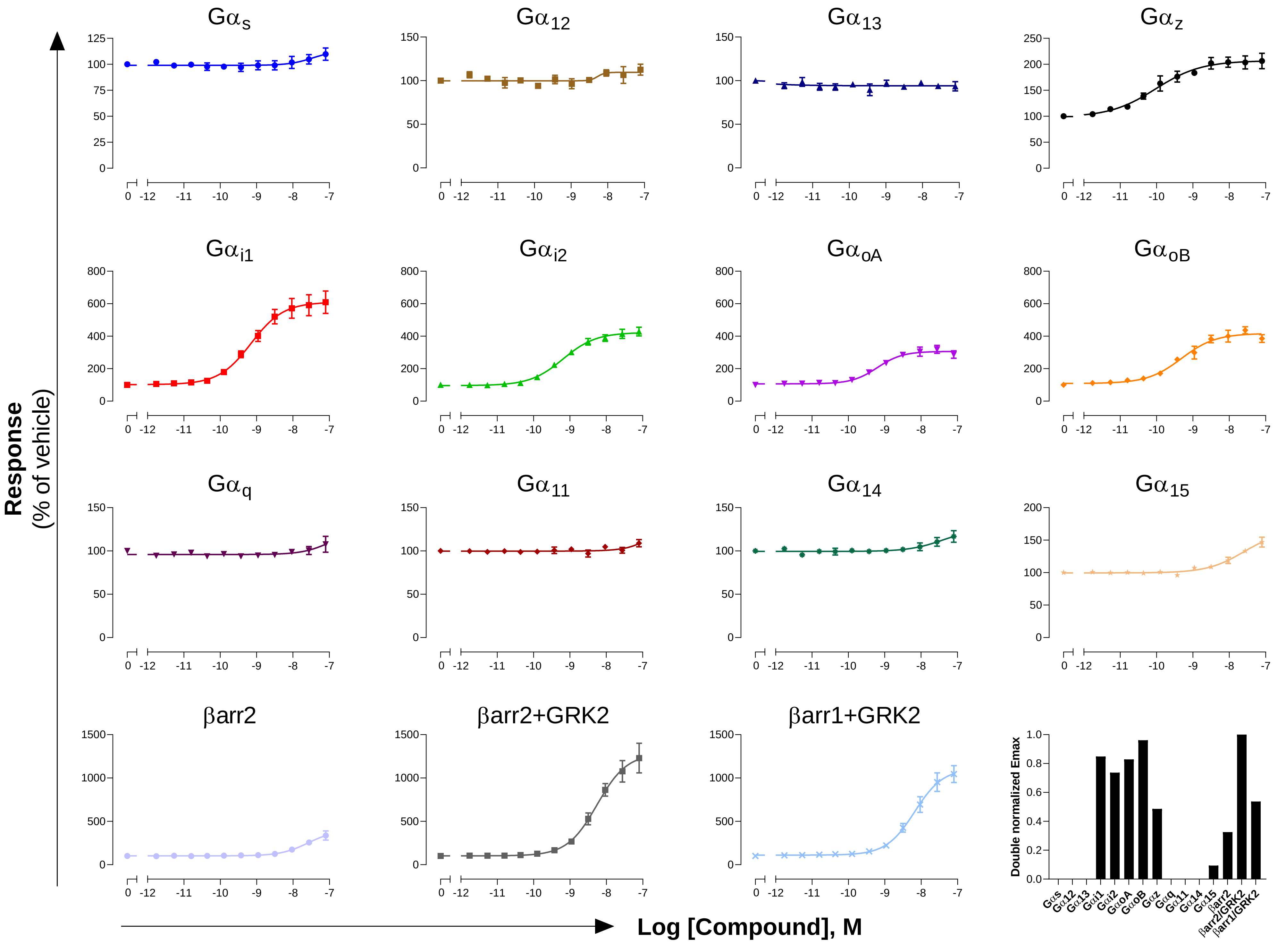

GPCR: CCR6
Ligand: CCL20

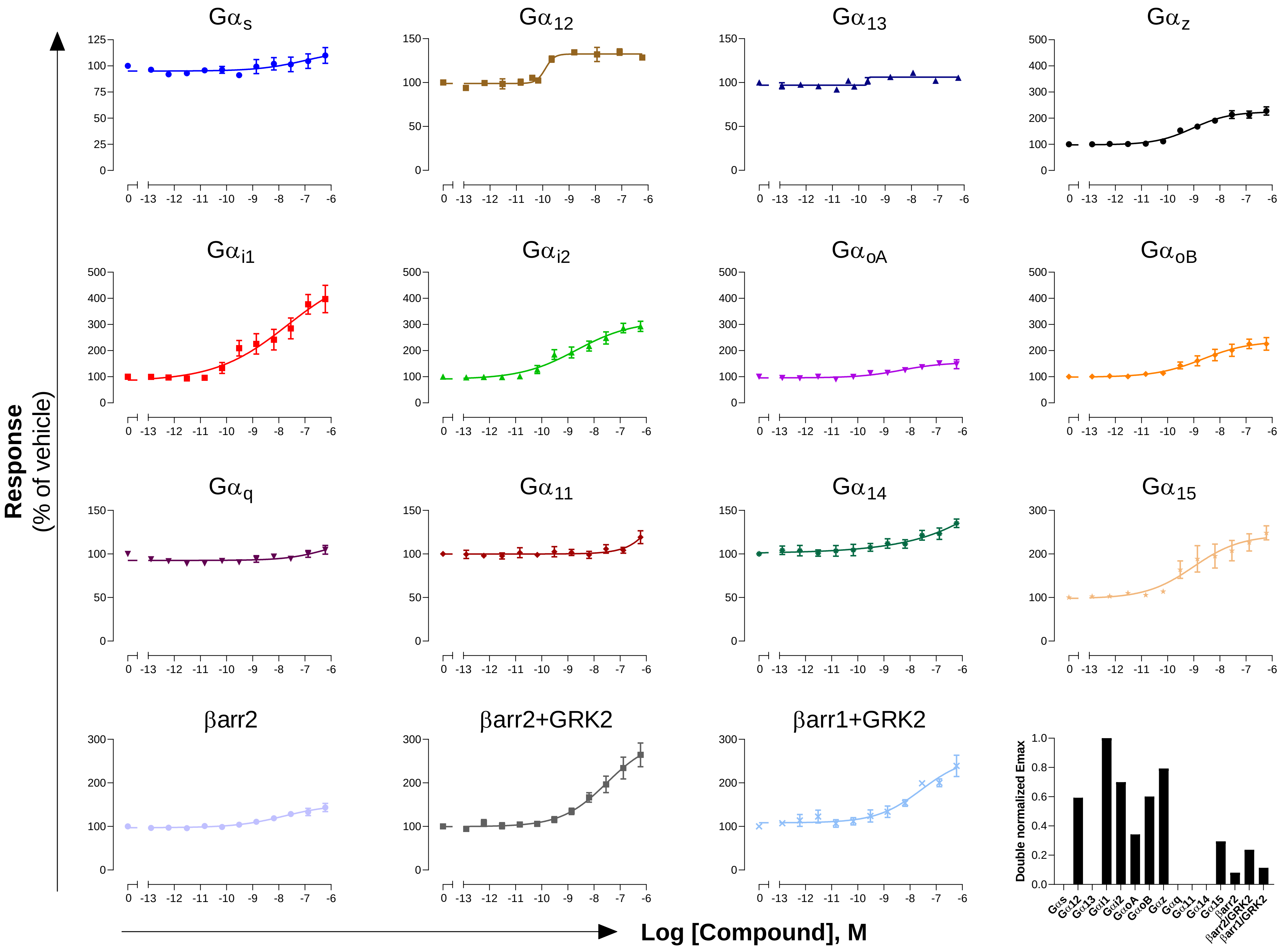

GPCR: CRFR1
Ligand: CRF

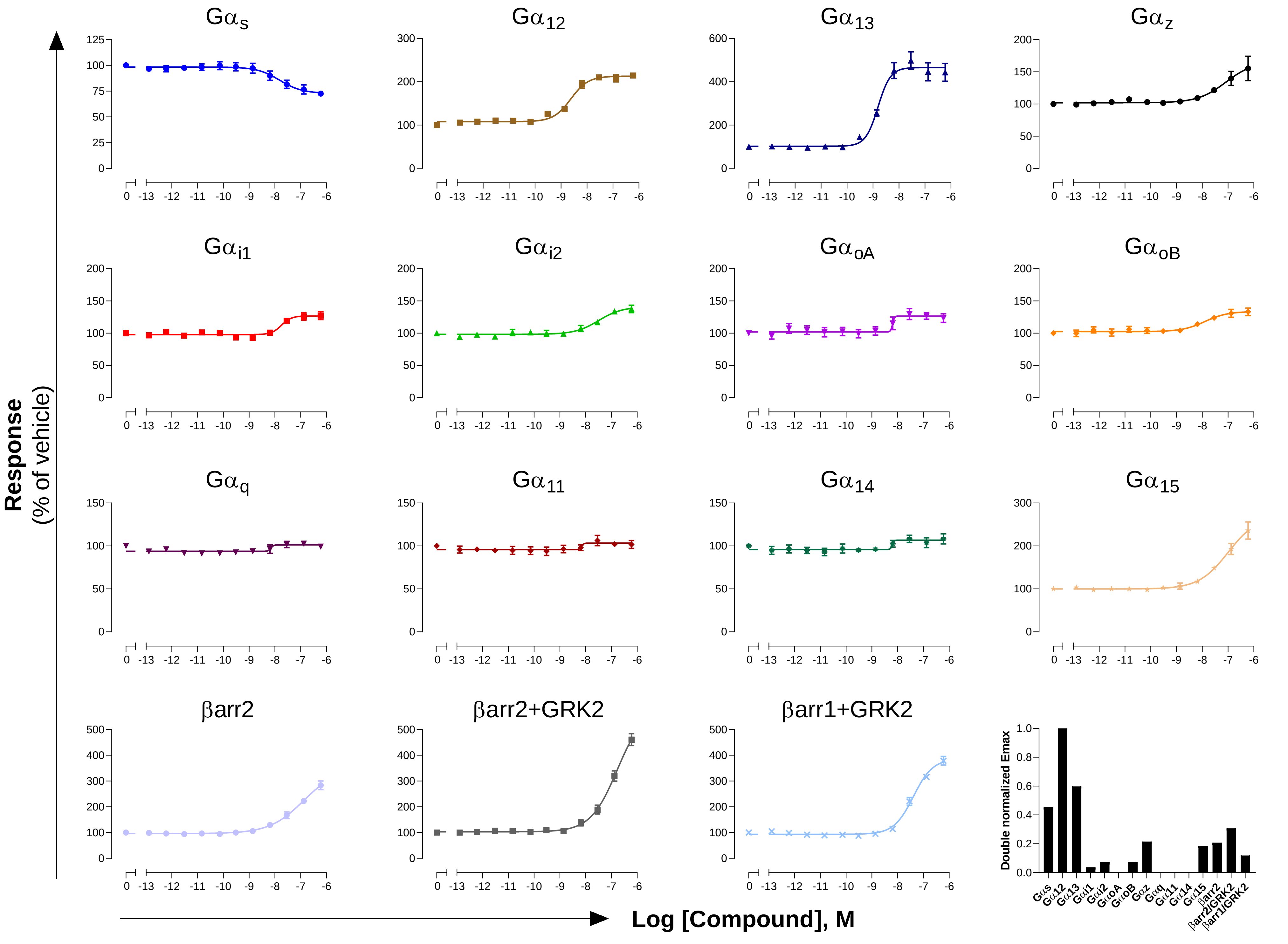

GPCR: CRFR2
Ligand: Urocortin II

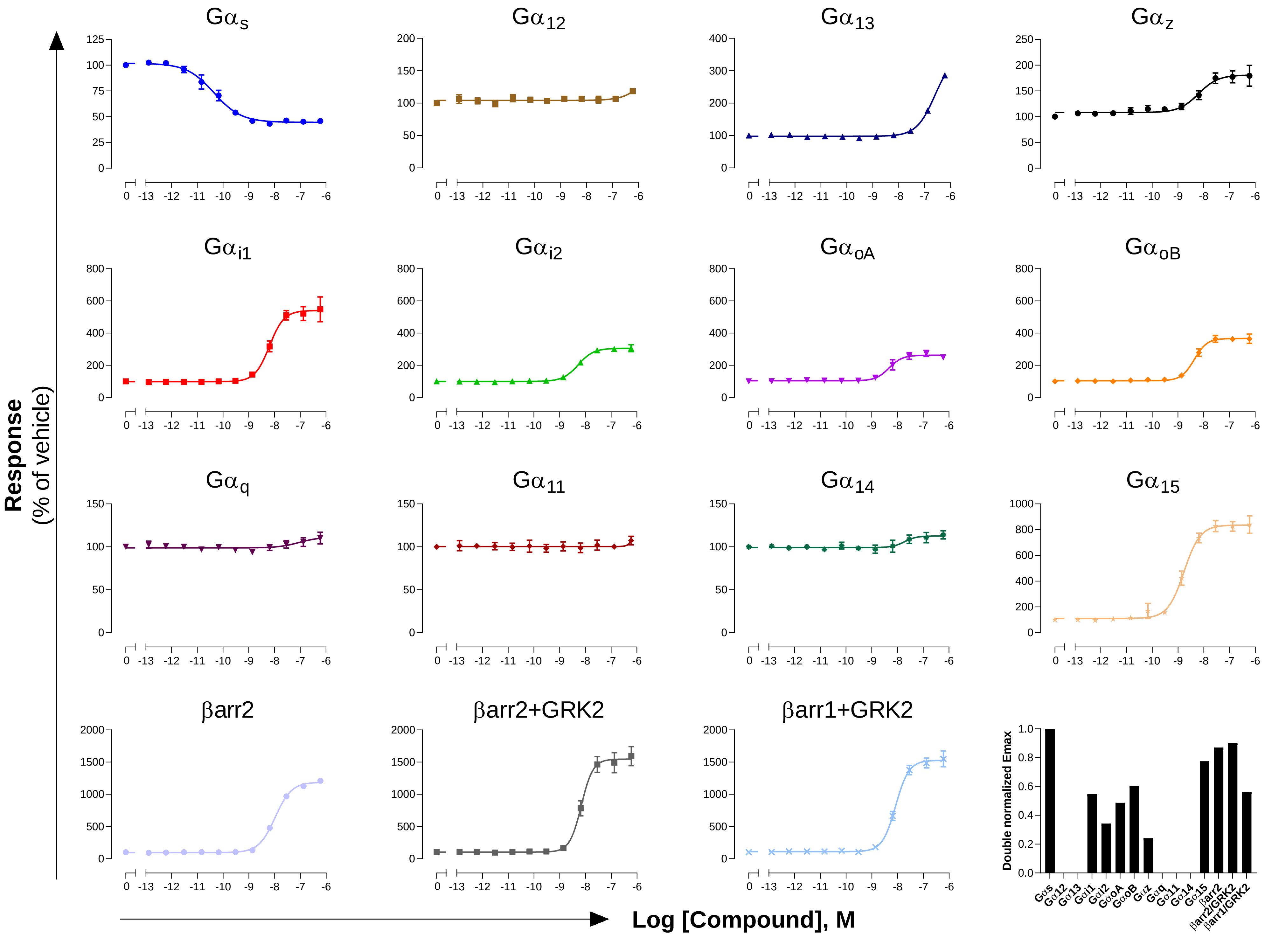

GPCR: CTR
Ligand: Calcitonin

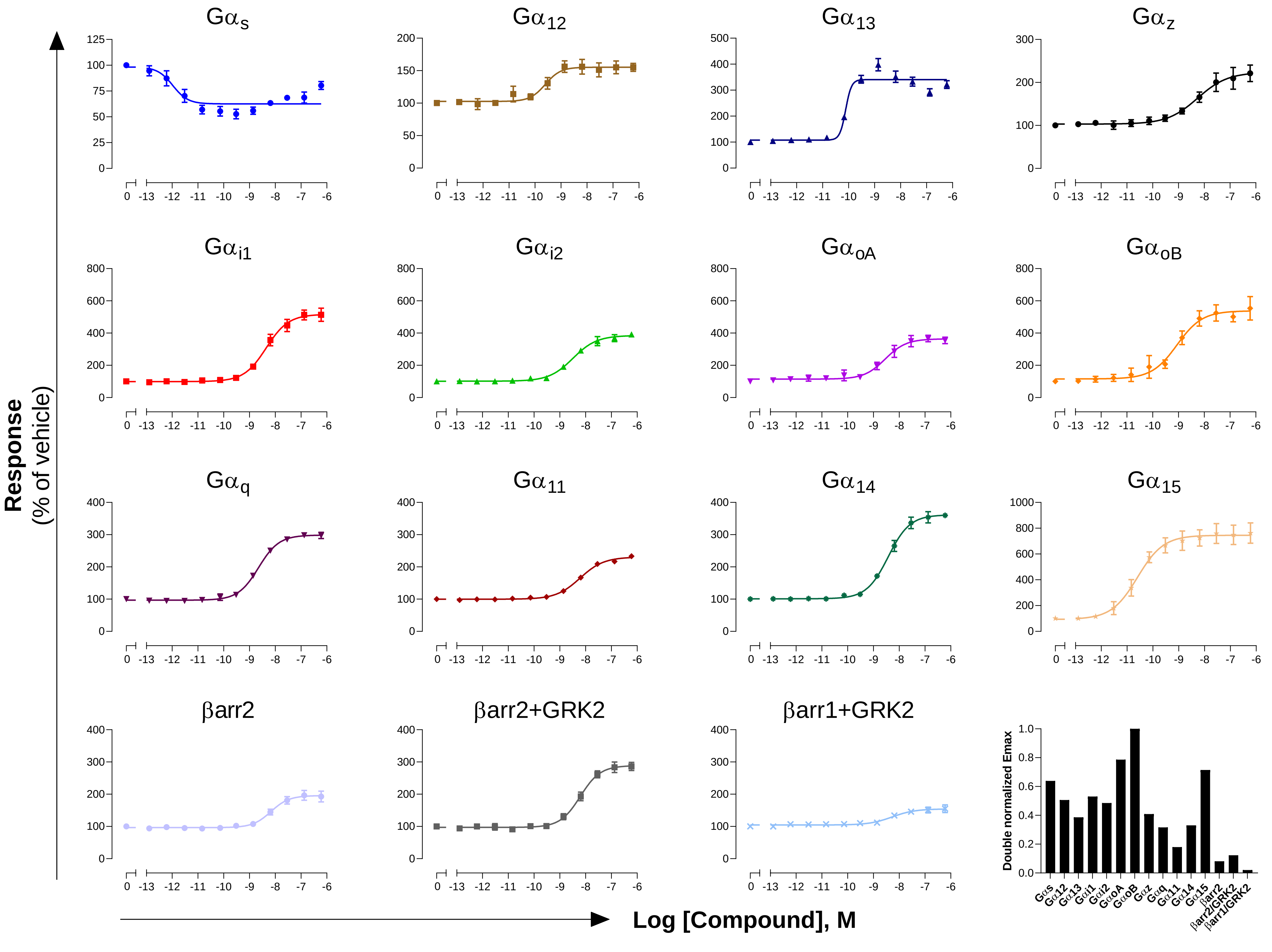

GPCR: CXCR2
Ligand: CXCL8

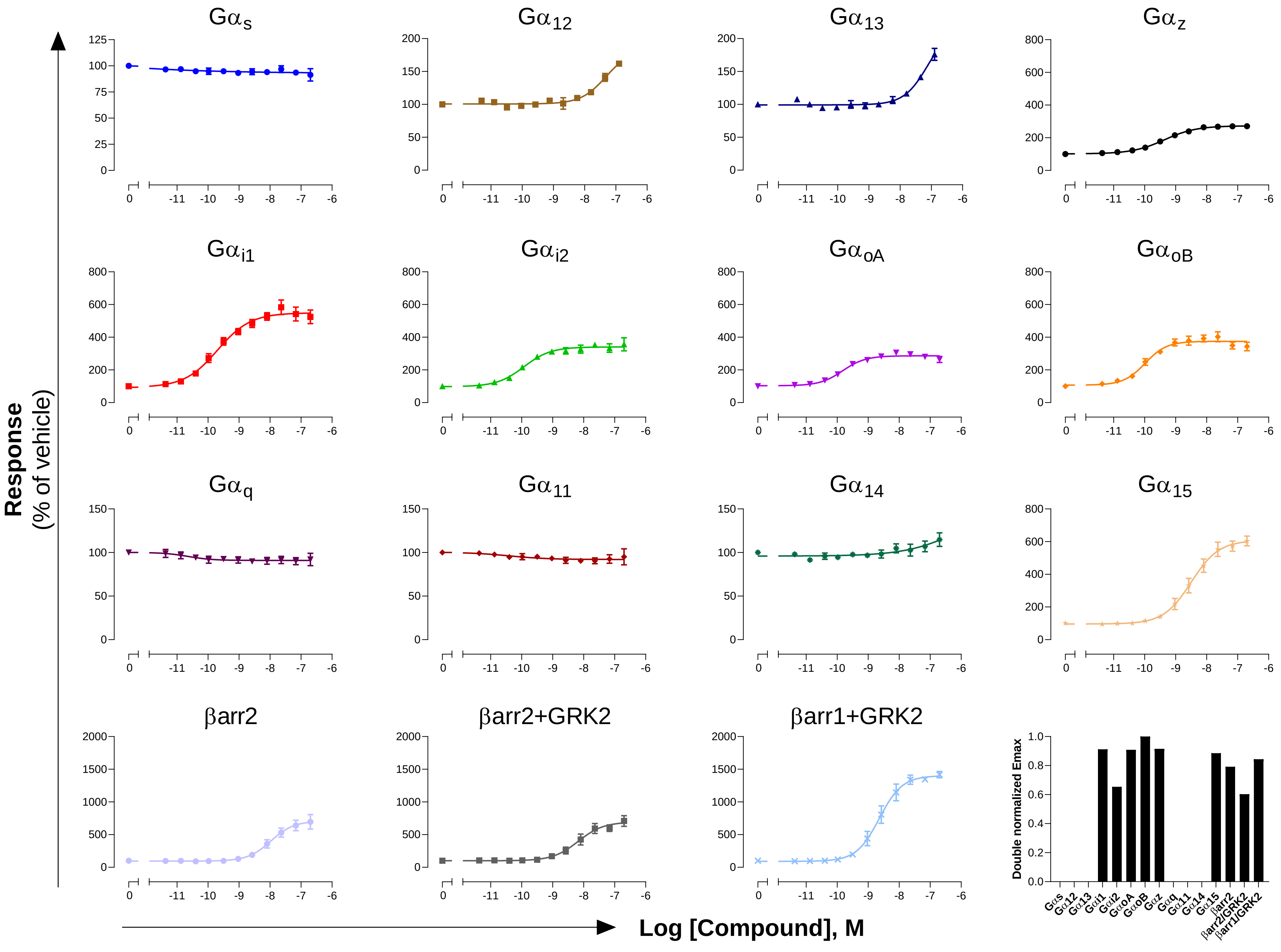

GPCR: CXCR4
Ligand: CXCL12

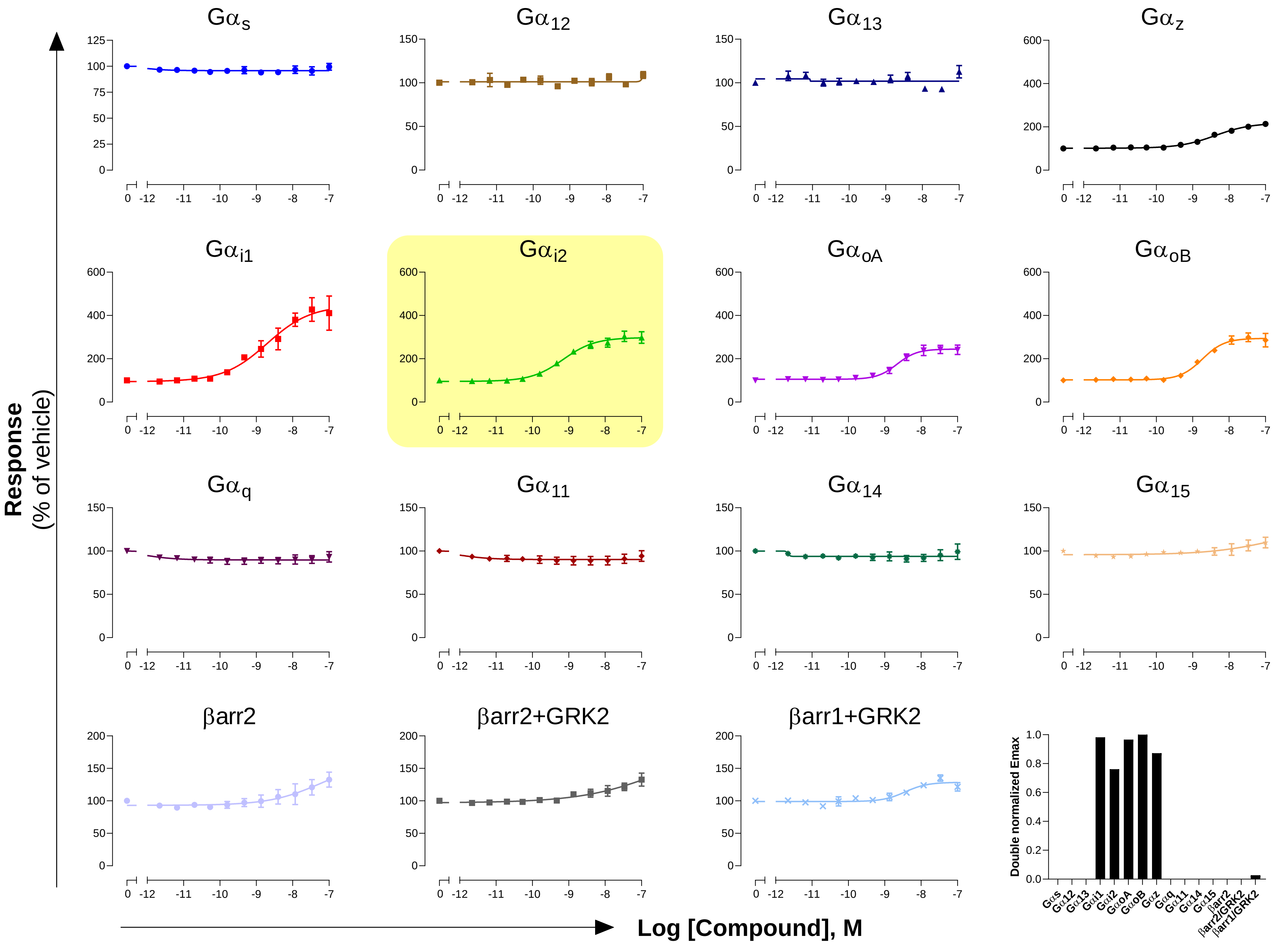

GPCR: CXCR5
Ligand: CXCL13

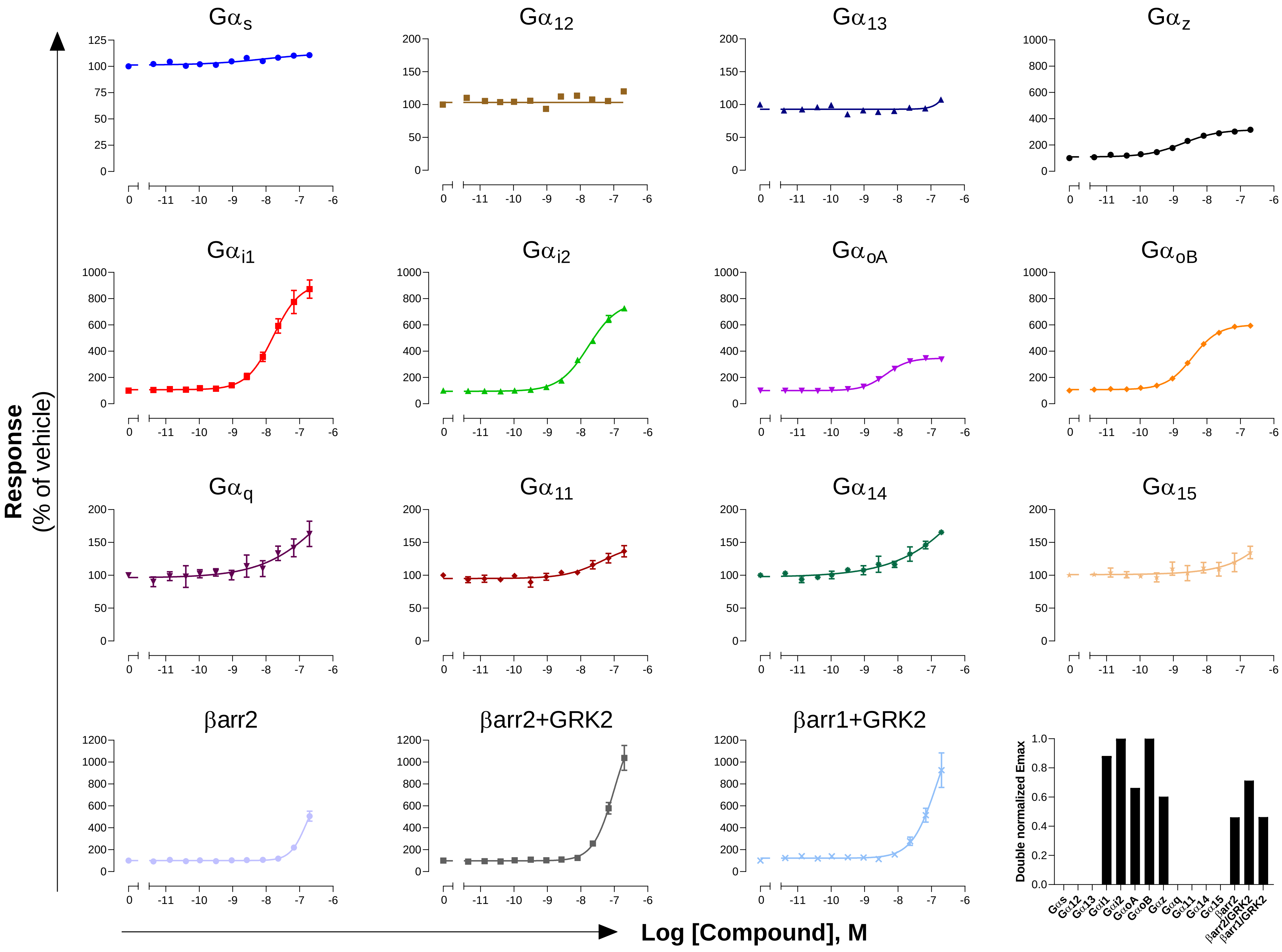

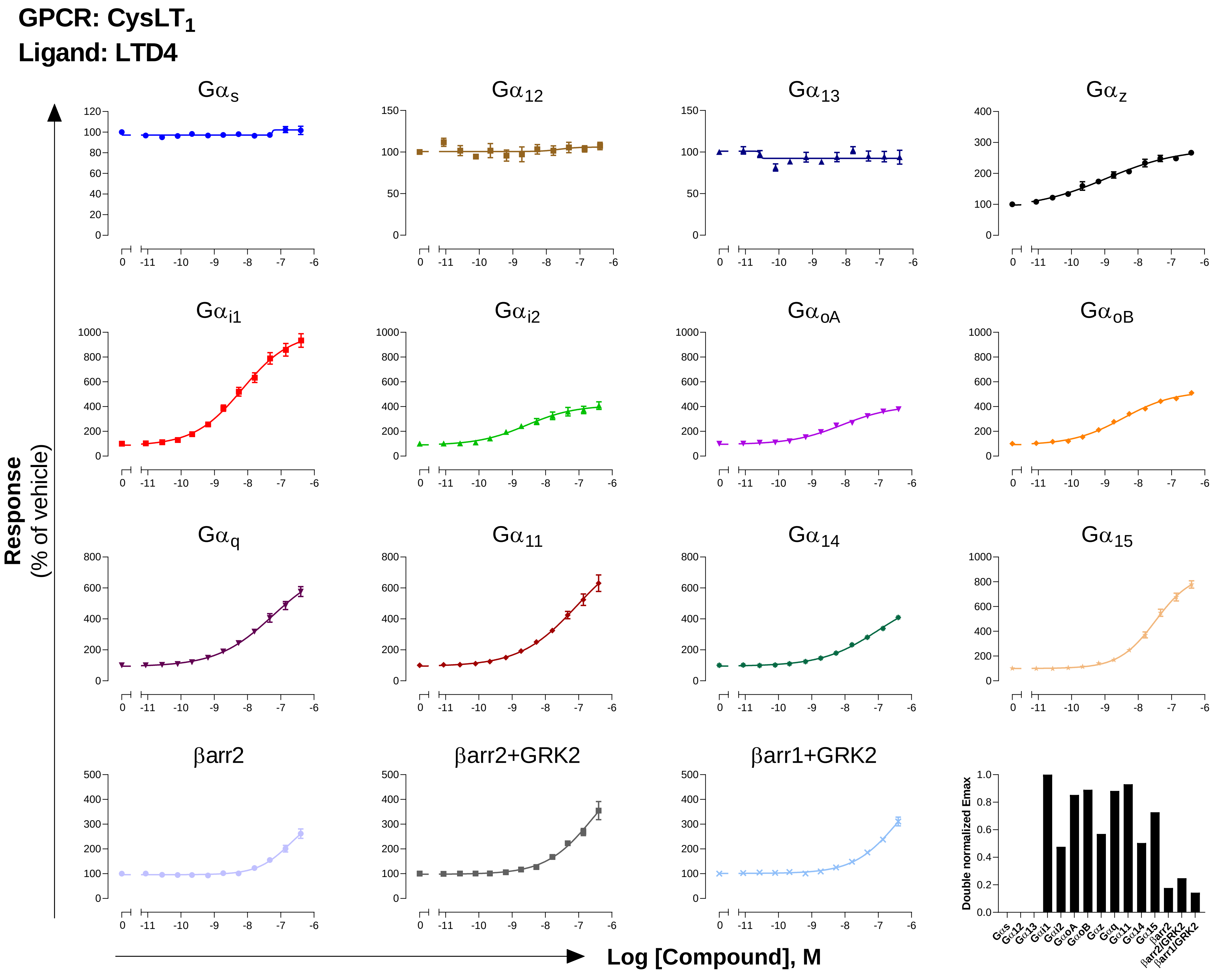

GPCR: CysLT1
Ligand: LTD4
Gαs
Gα12
Gα13
Gαz
Gαi1
Gαi2
GαoA
GαoB
Gαq
Gα11
Gα14
Gα15
βarr2
βarr2+GRK2
βarr1+GRK2
Response
(% of vehicle)
Log [Compound], M
Double normalized Emax

GPCR: CysLT$_2$

Ligand: LTD4

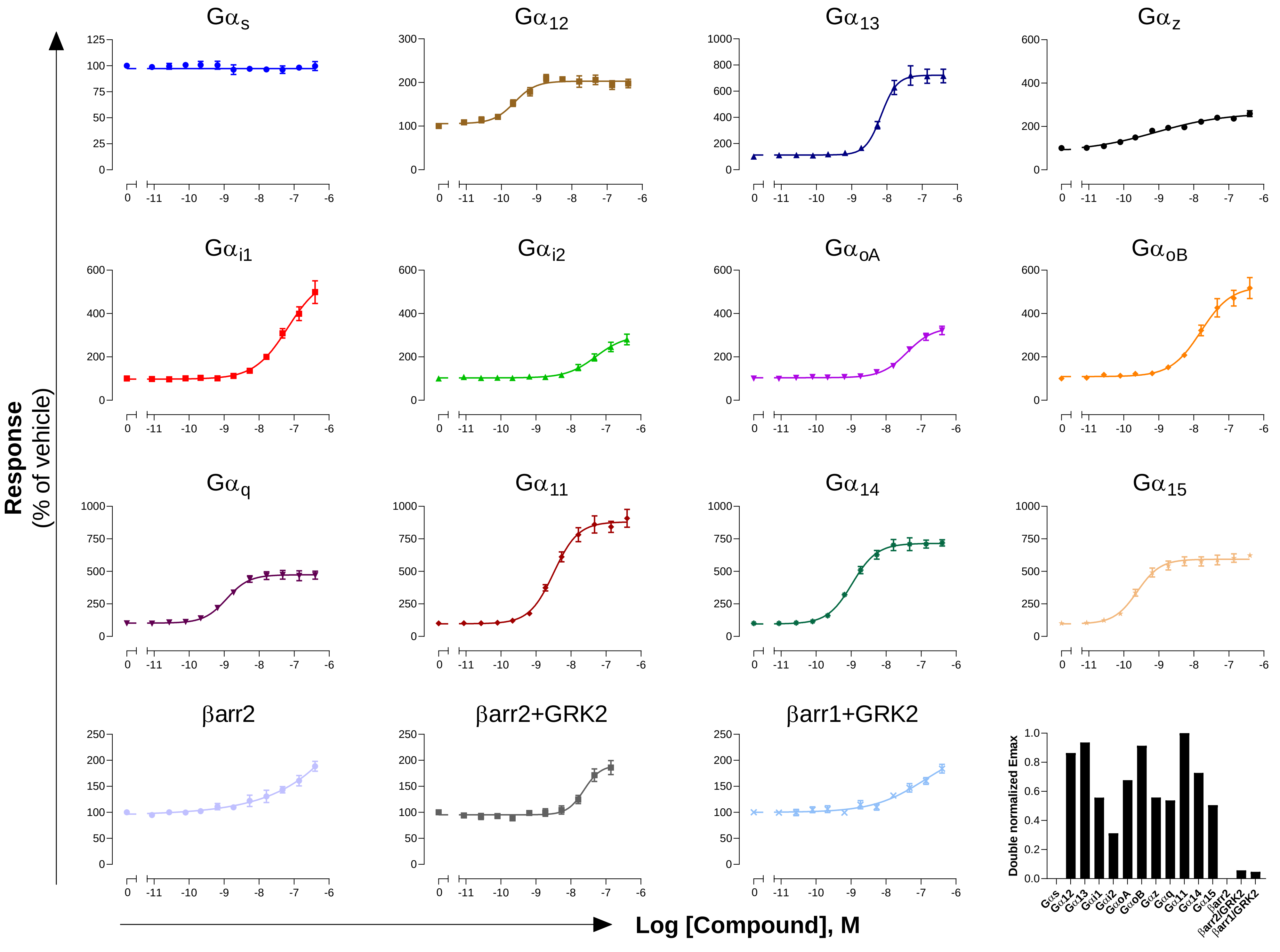

GPCR: D_1
Ligand: Dopamine

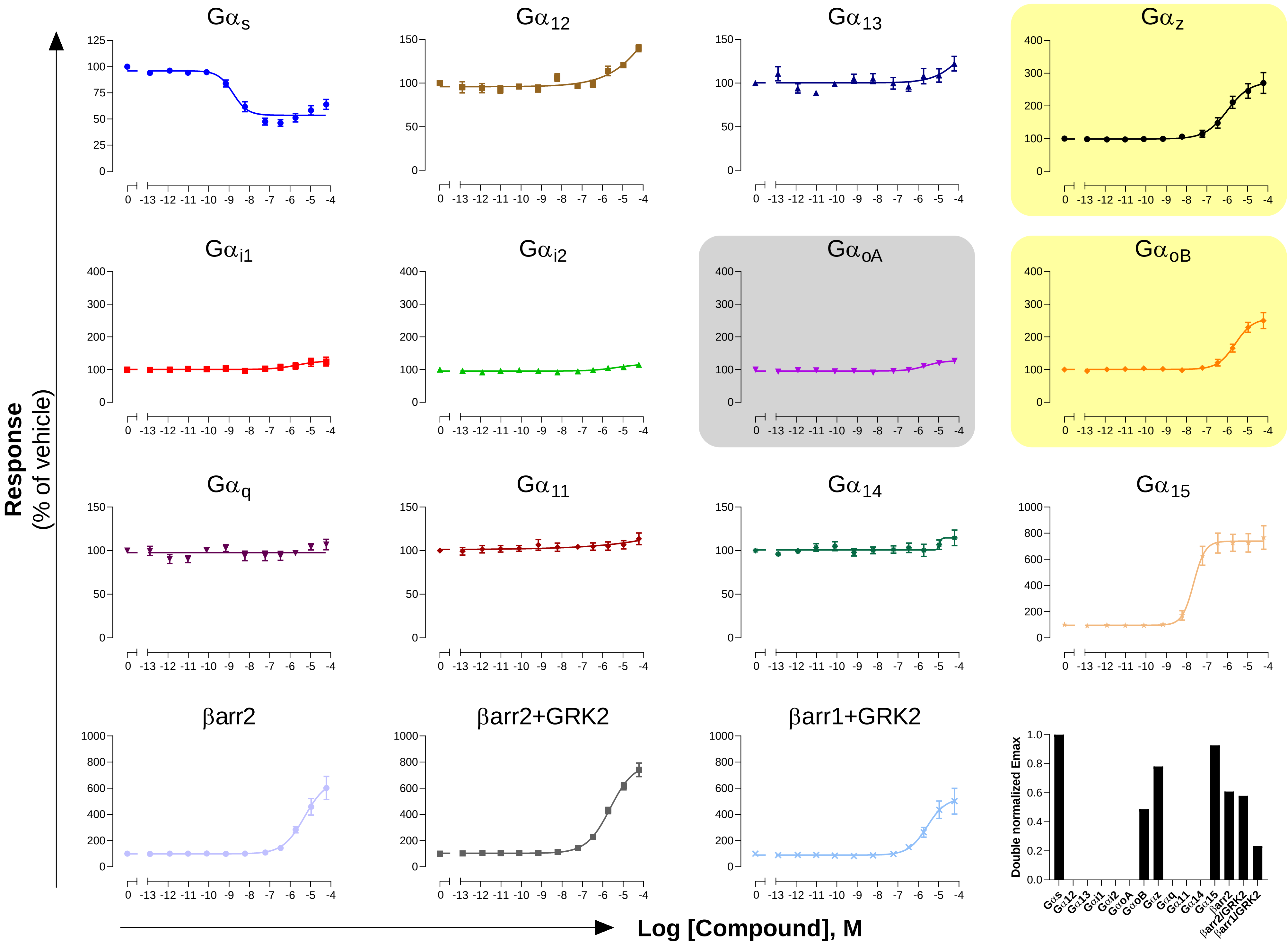

GPCR: D_2

Ligand: Dopamine

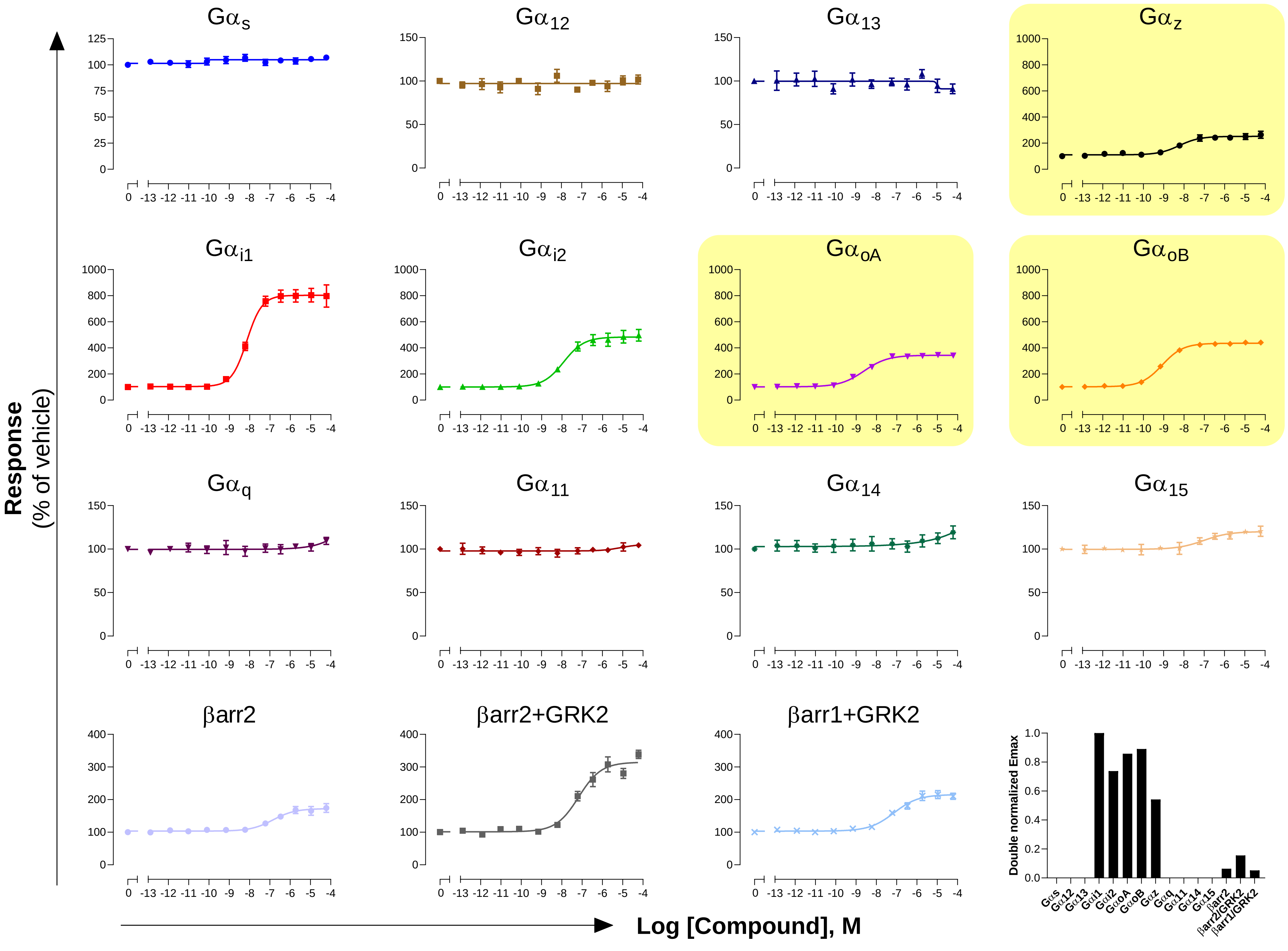

GPCR: D_5

Ligand: Dopamine

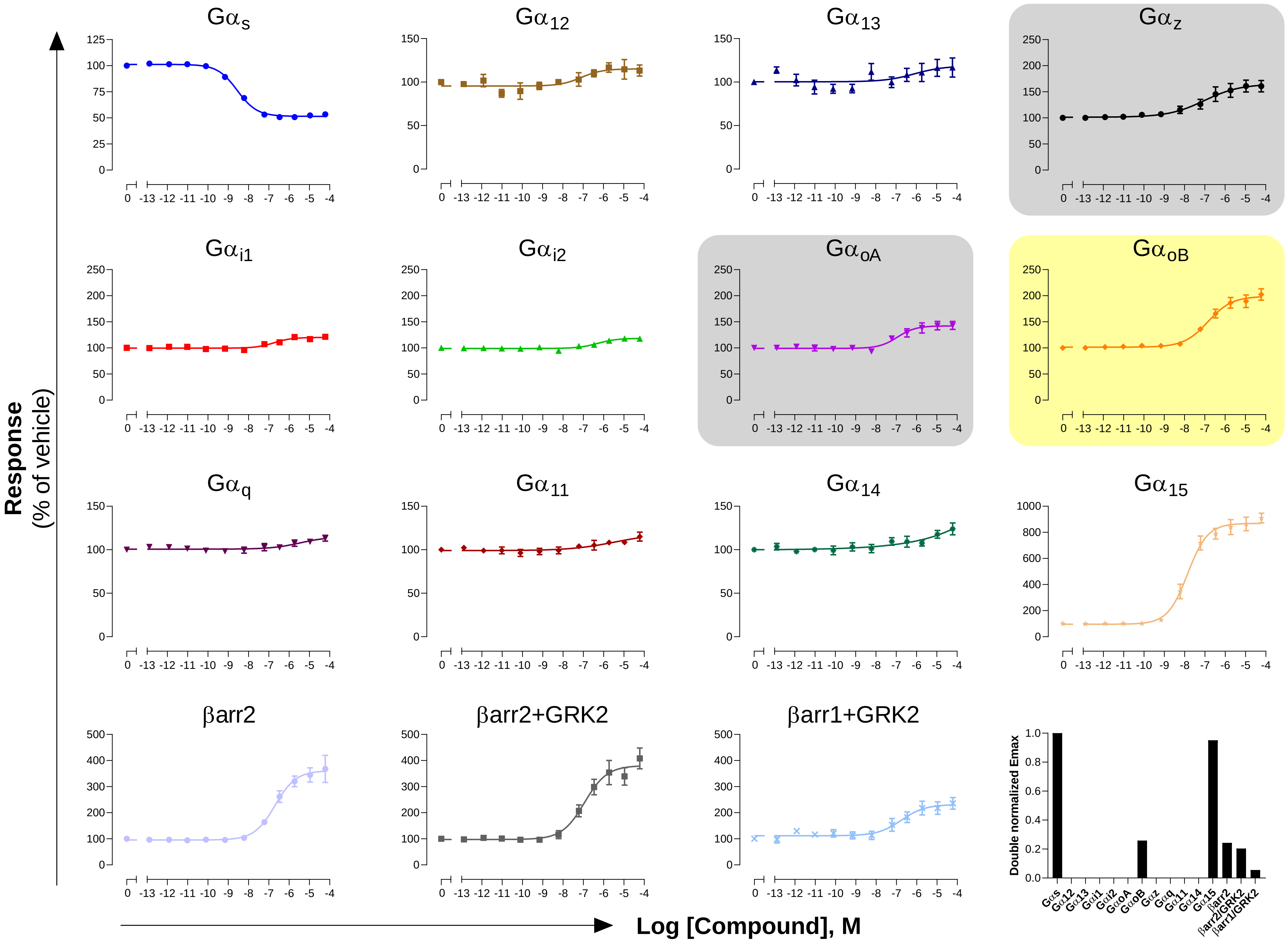

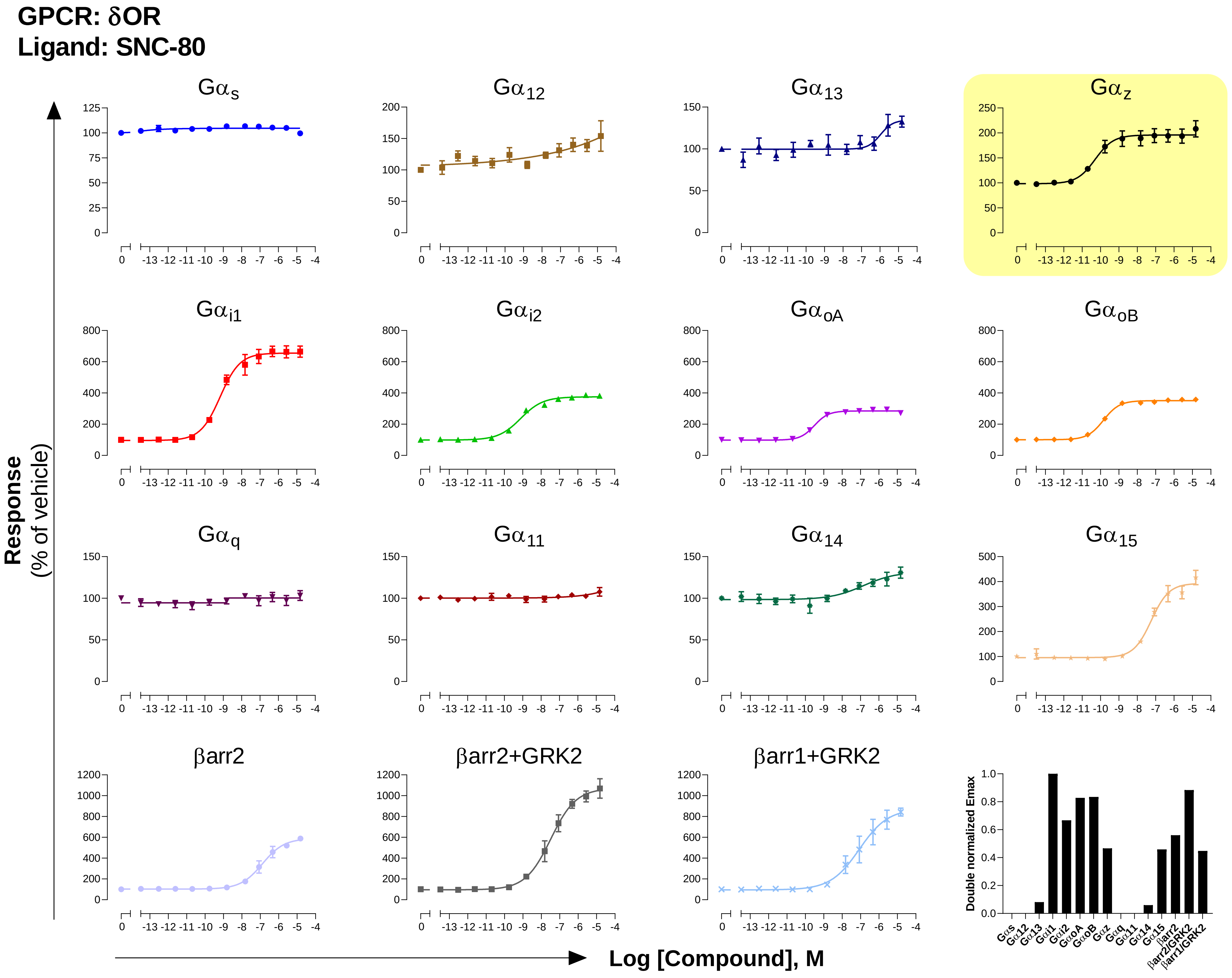

GPCR: δOR
Ligand: SNC-80
Gαs
Gα12
Gα13
Gαz
Gαi1
Gαi2
GαoA
GαoB
Gαq
Gα11
Gα14
Gα15
βarr2
βarr2+GRK2
βarr1+GRK2
Response
(% of vehicle)
Log [Compound], M
Double normalized Emax

GPCR: EP_1

Ligand: PGE2

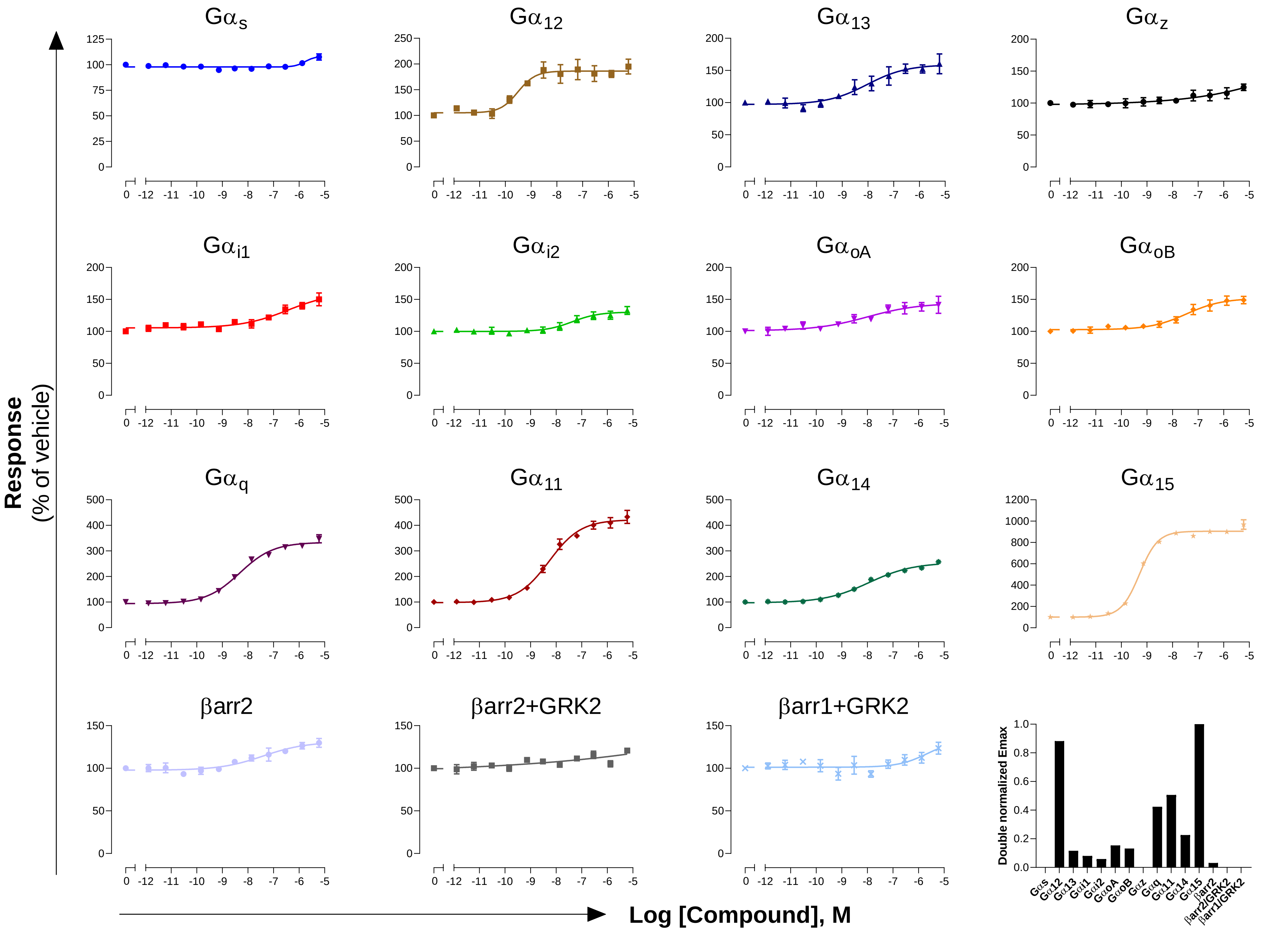

GPCR: EP_2
Ligand: PGE2

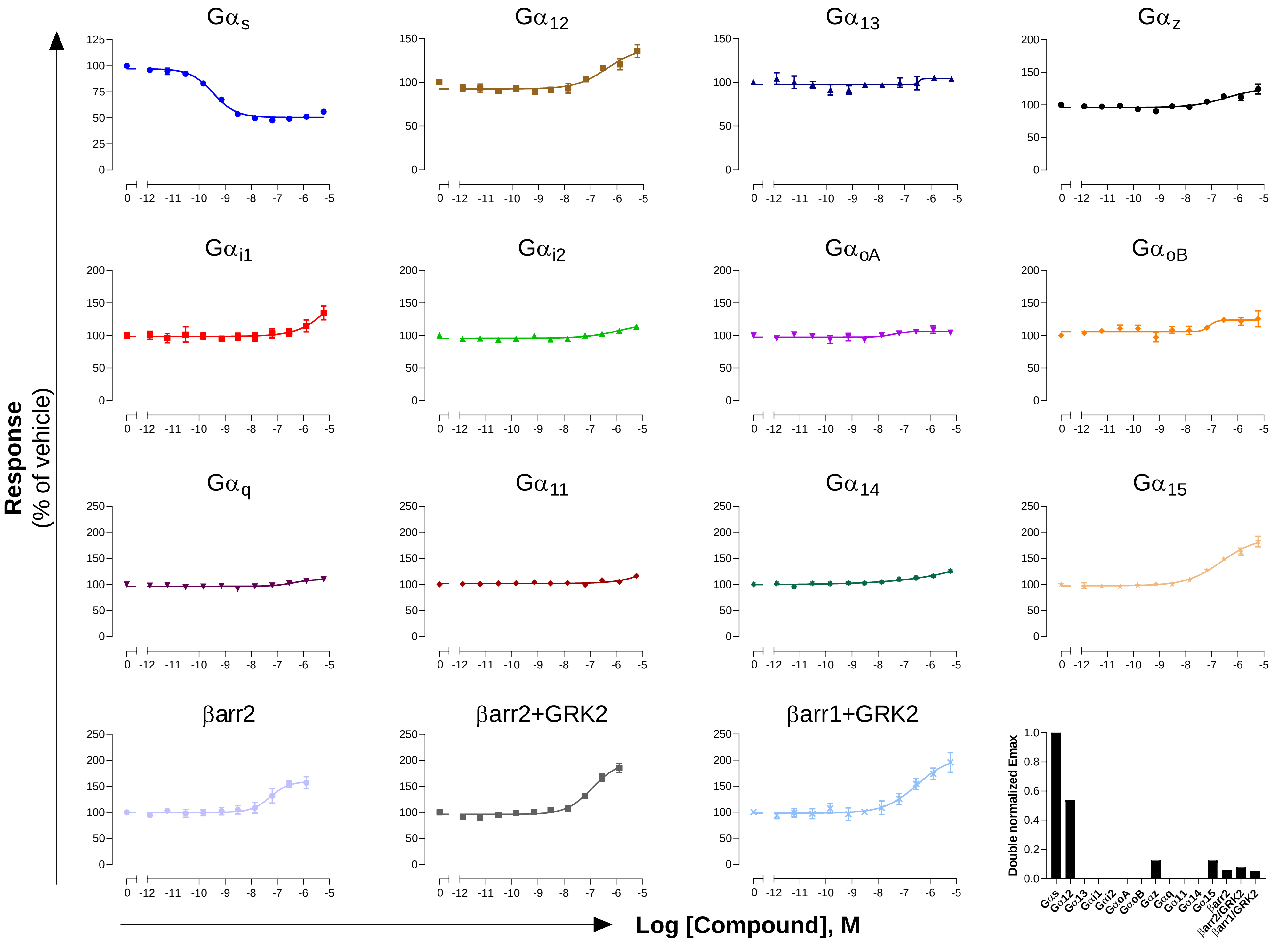

GPCR: EP_3
Ligand: PGE2

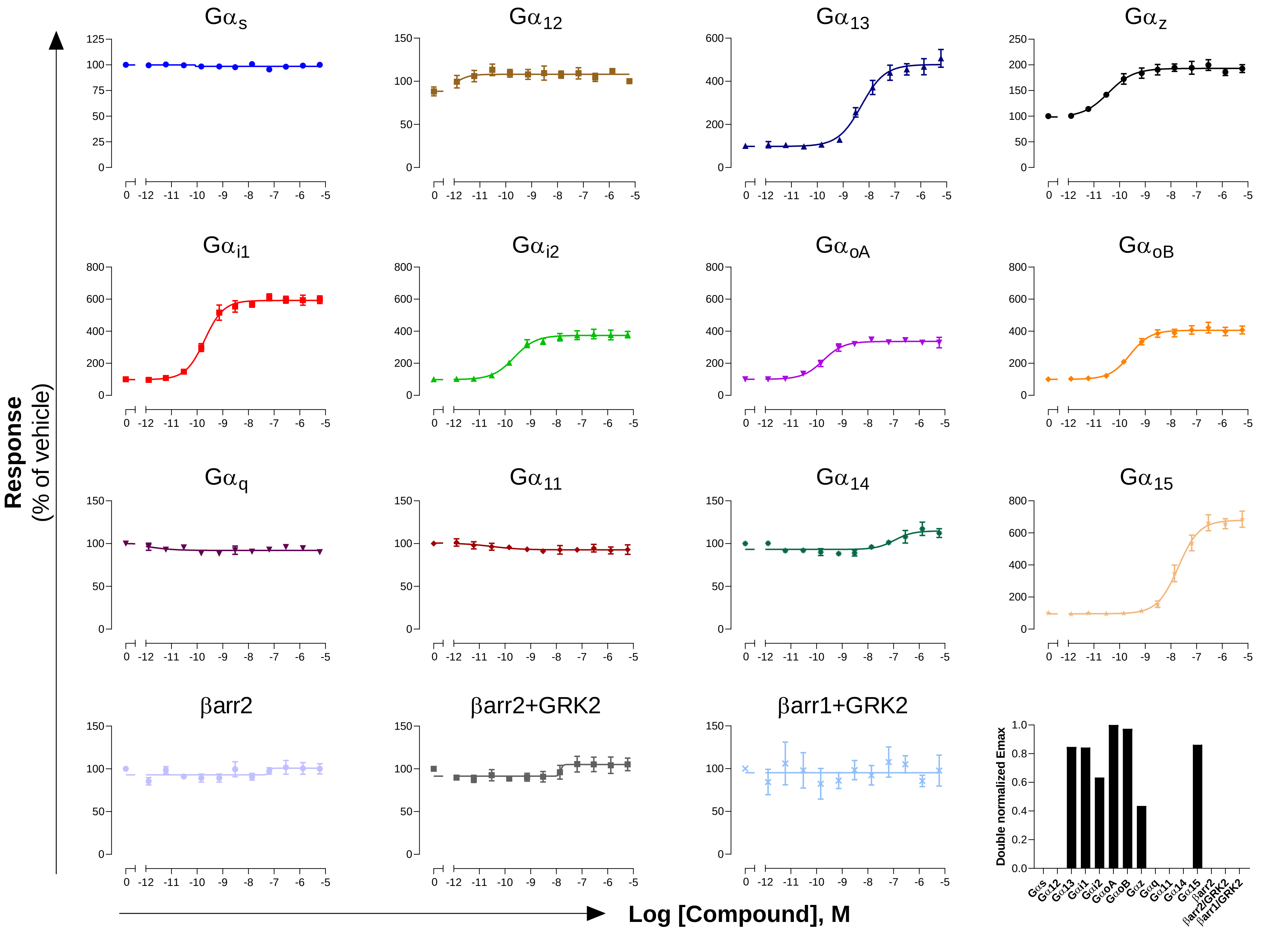

GPCR: EP_4

Ligand: PGE2

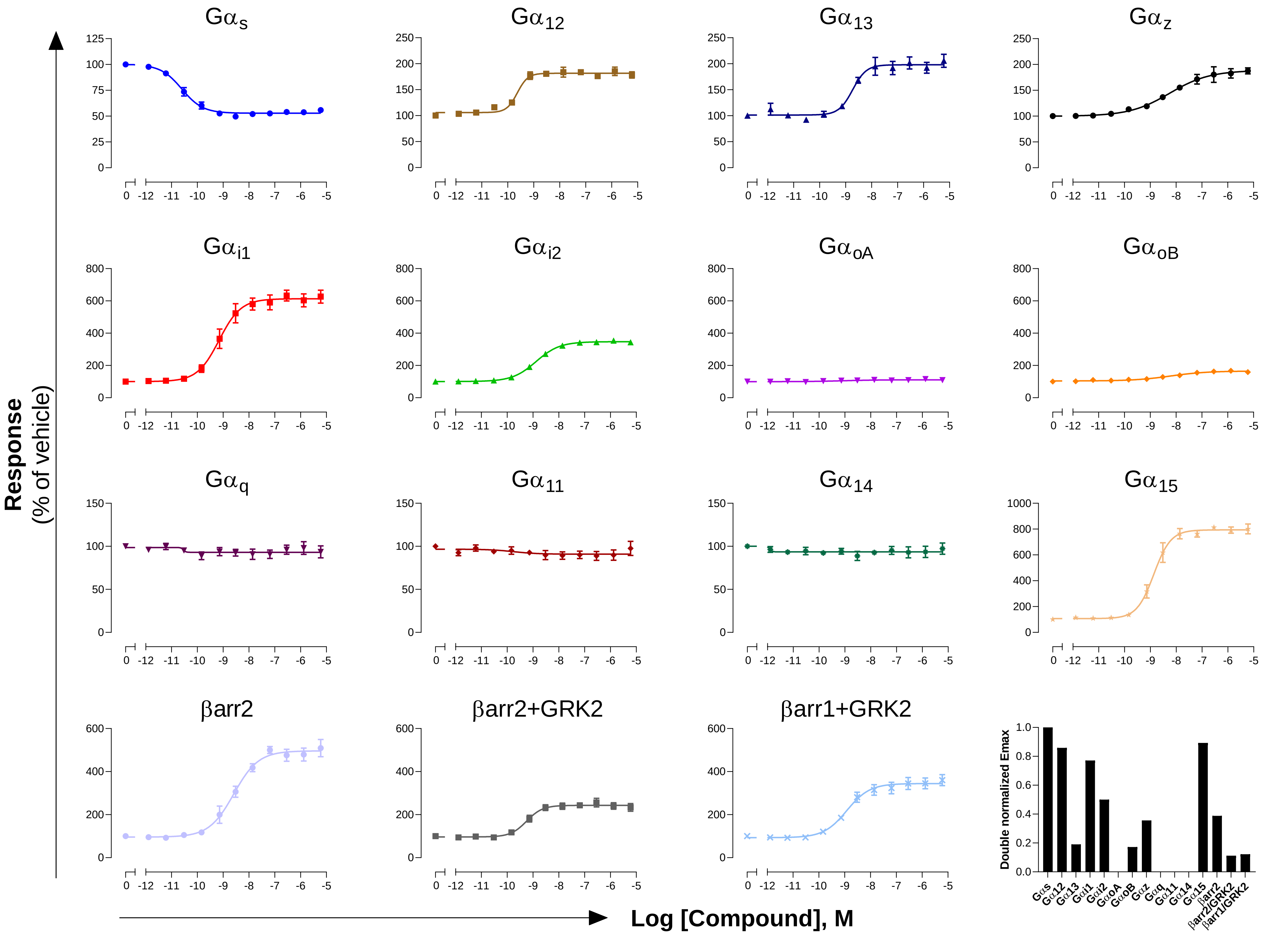

GPCR: ET_A
Ligand: Endothelin-1

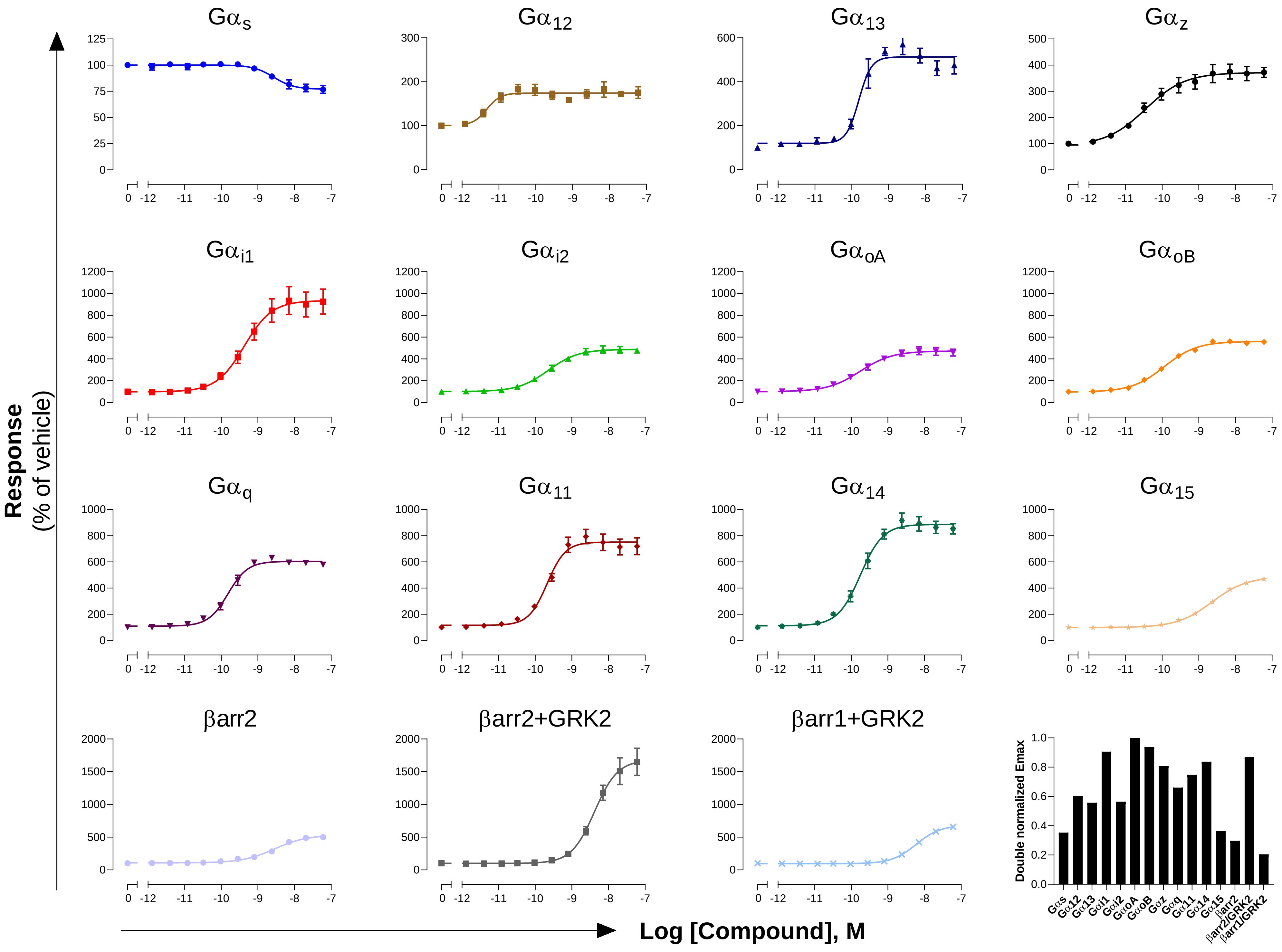

GPCR: FFA2
Ligand: Propionate

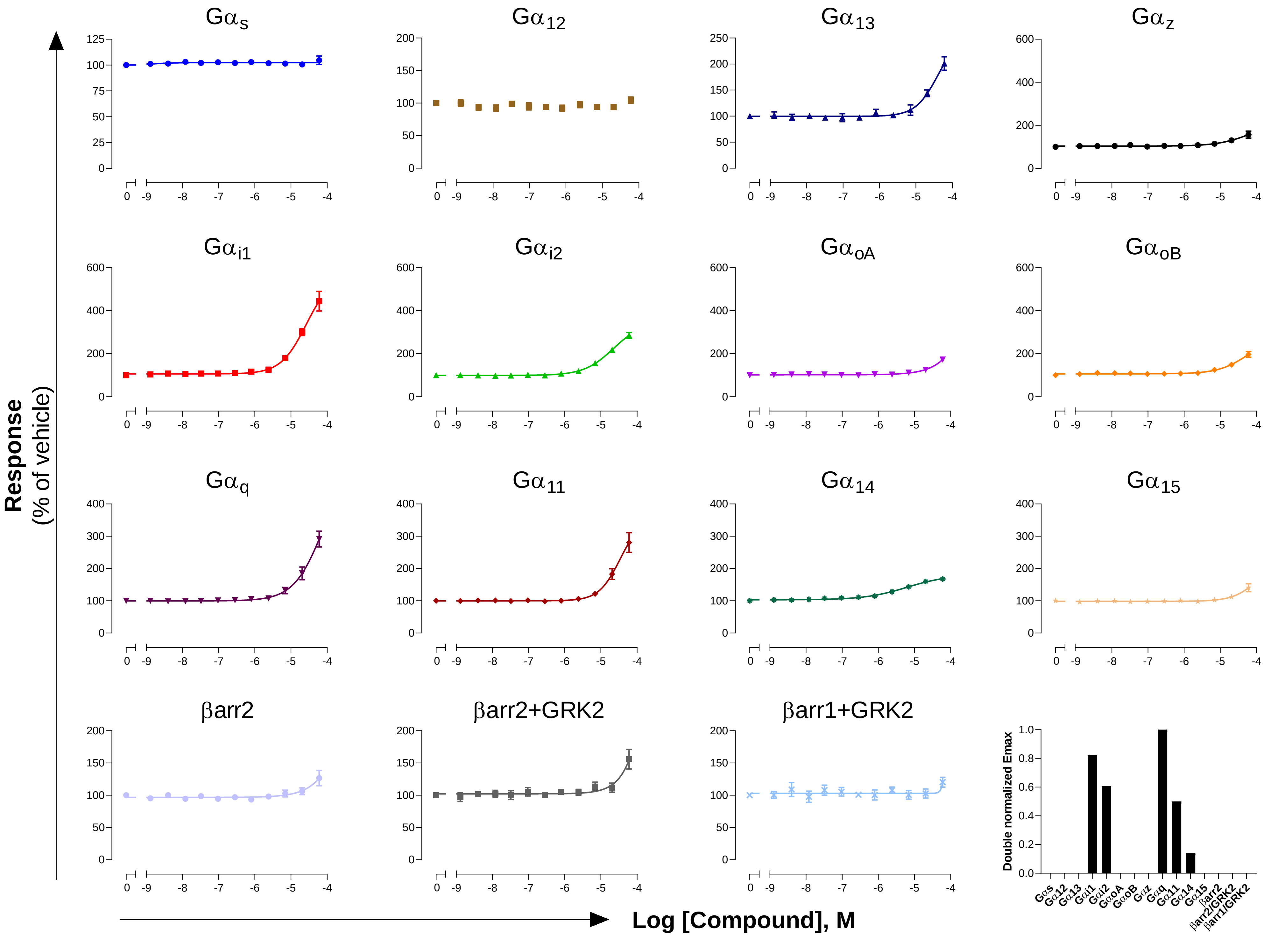

GPCR: FFA3
Ligand: Propionate

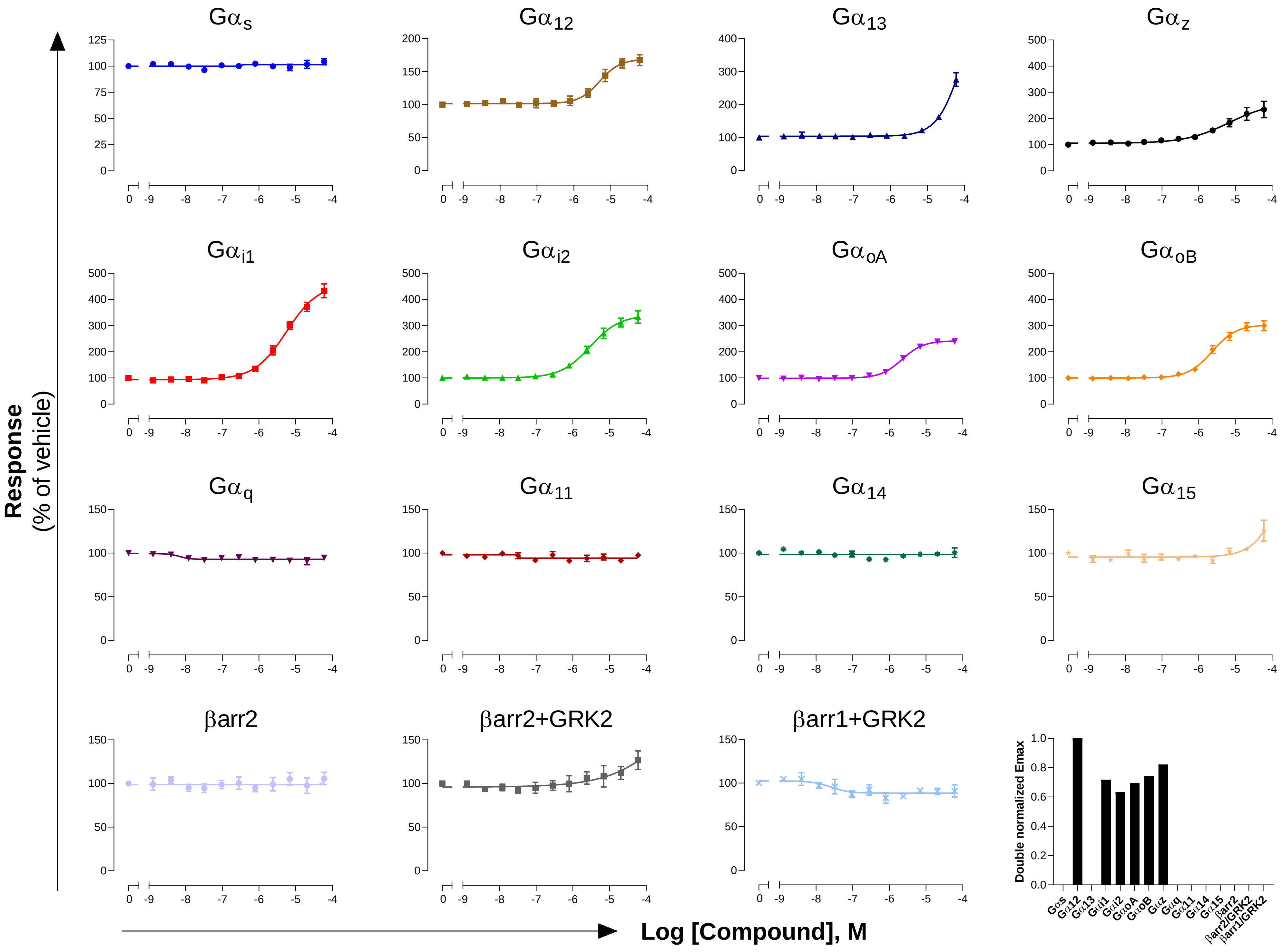

GPCR: FFA4
Ligand: α-linolenic acid

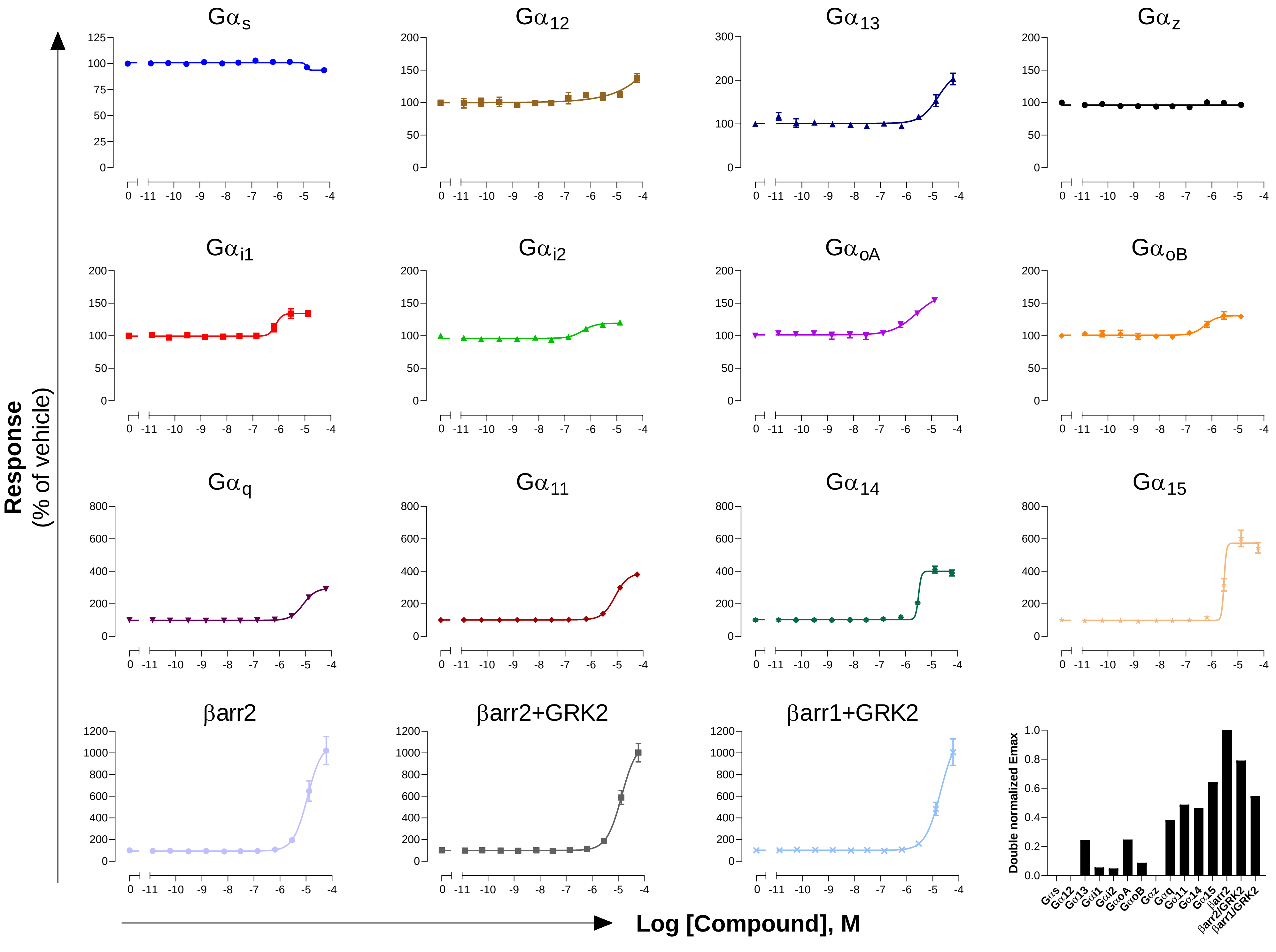

GPCR: FP
Ligand: PGD2

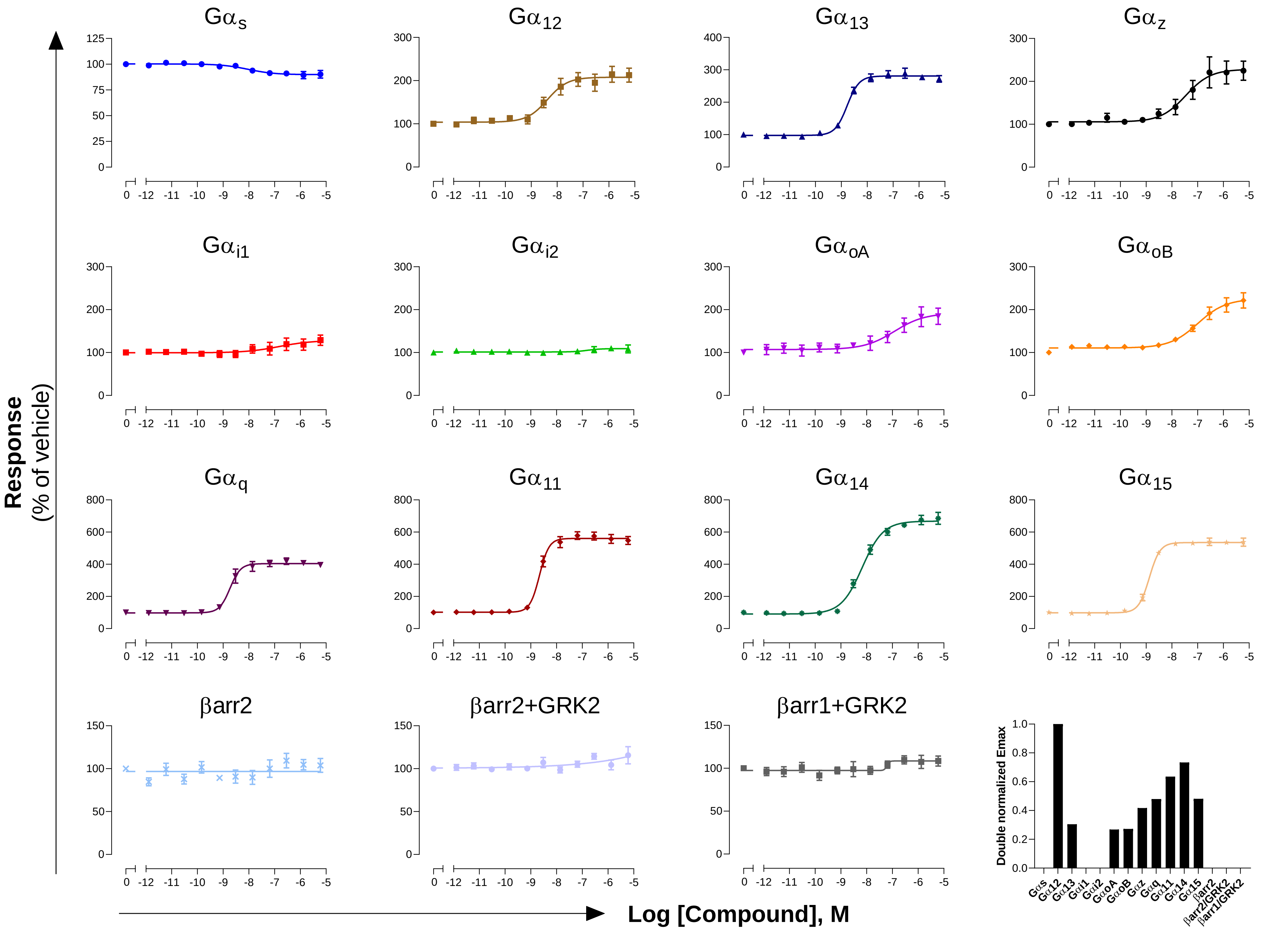

GPCR: GCGR
Ligand: Glucagon

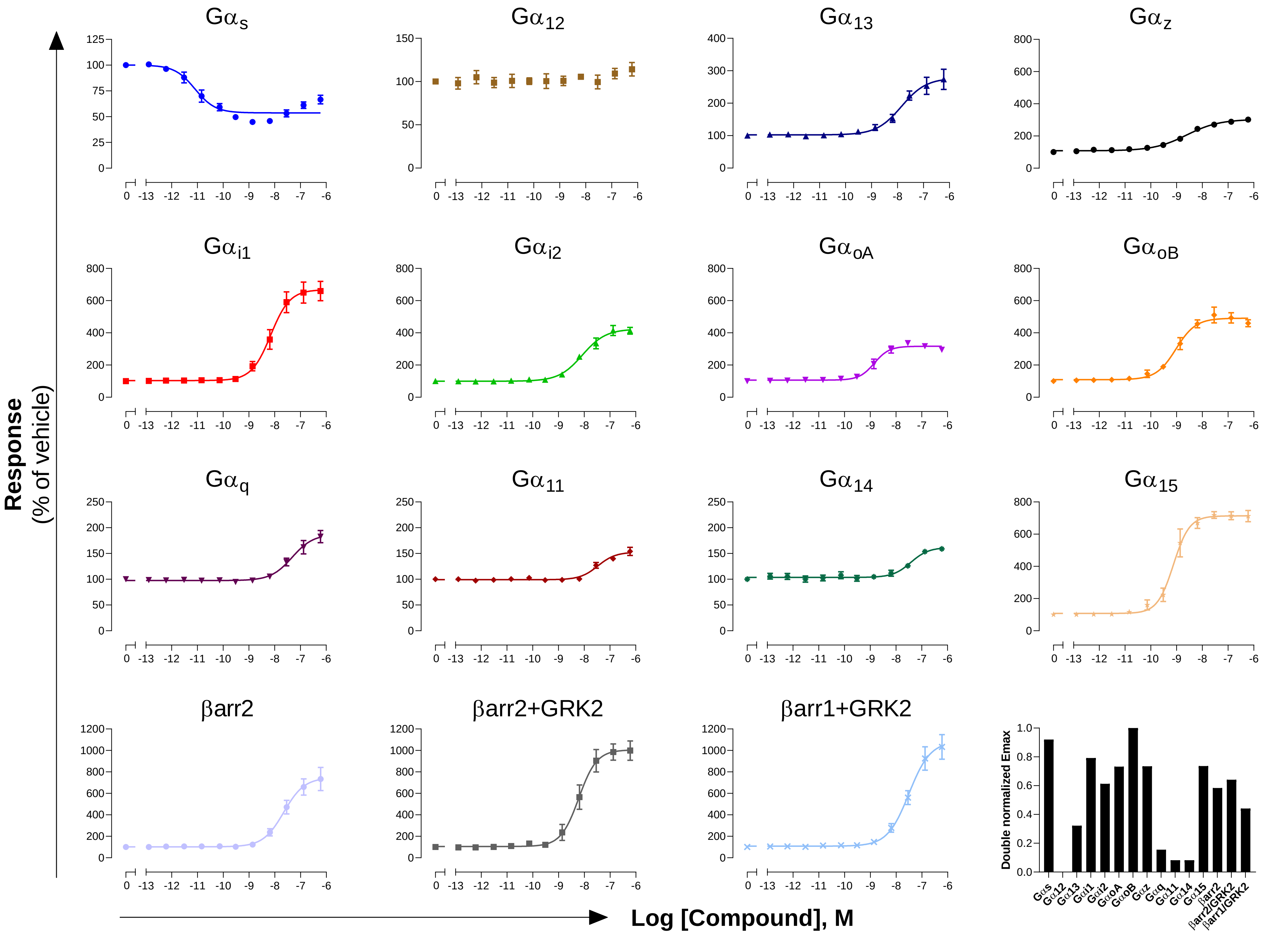

GPCR: GHSR
Ligand: Ghrelin

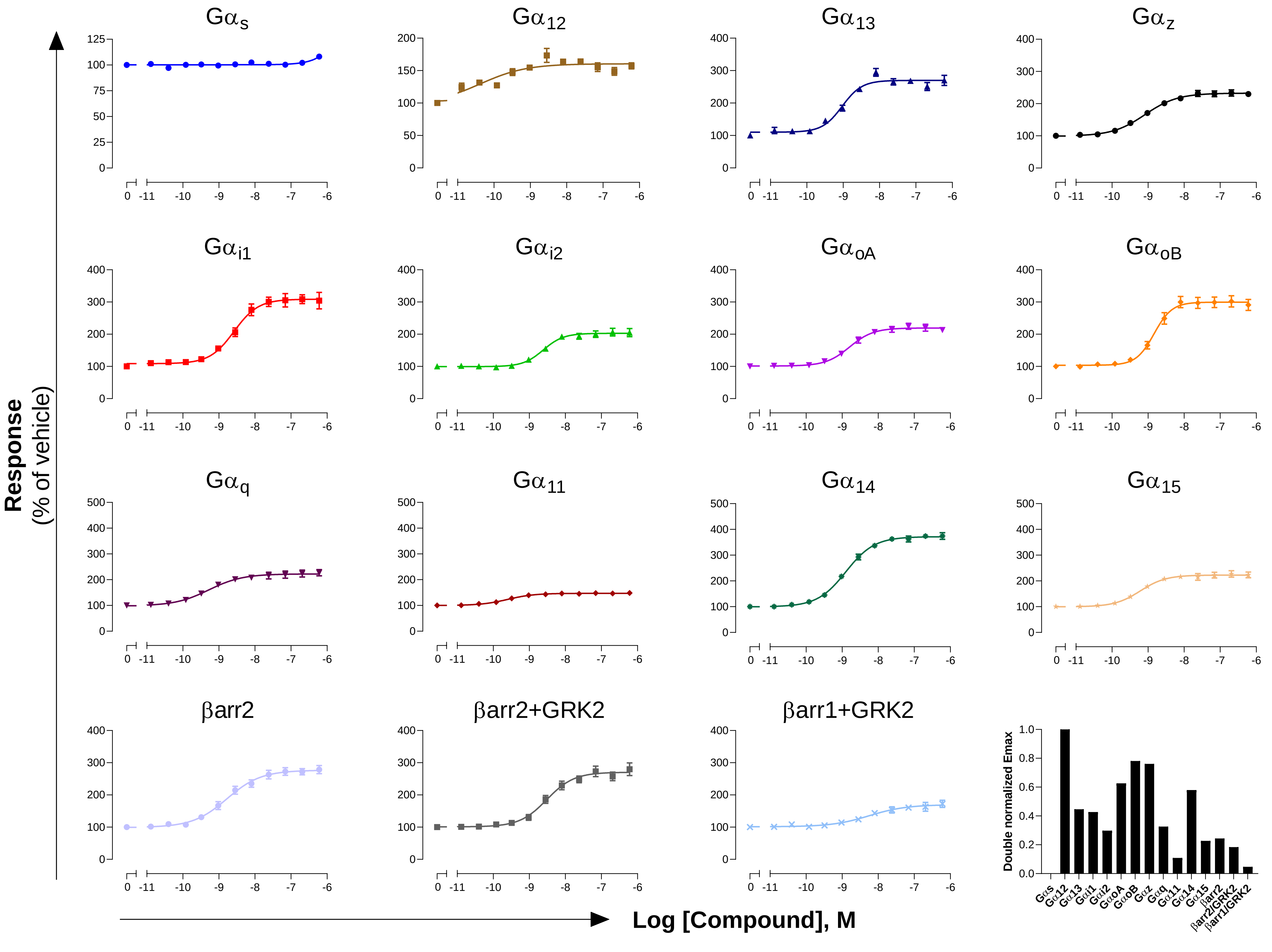

GPCR: GIP
Ligand: GIP

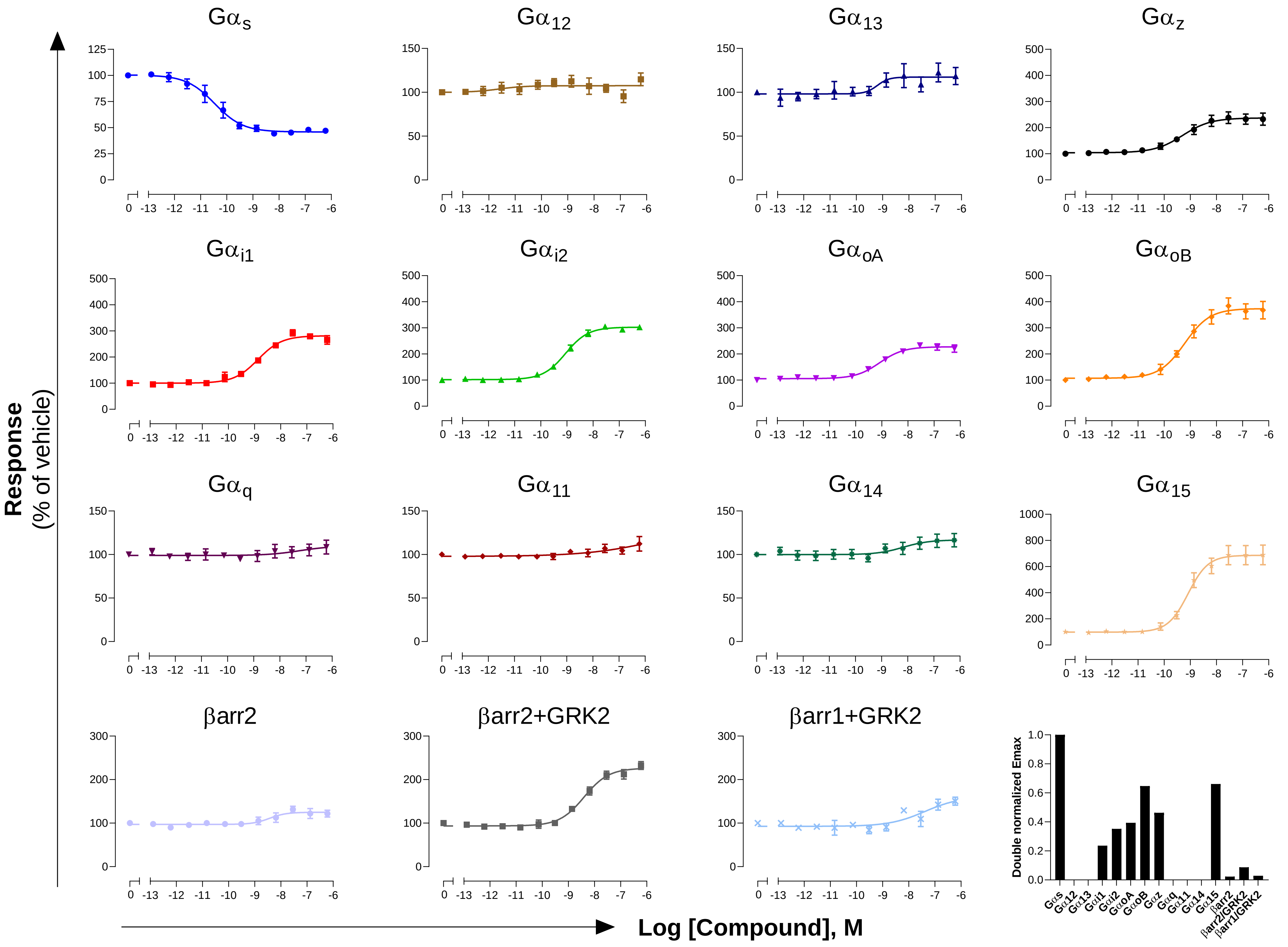

GPCR: GLP-1
Ligand: GLP-1 (7-36)

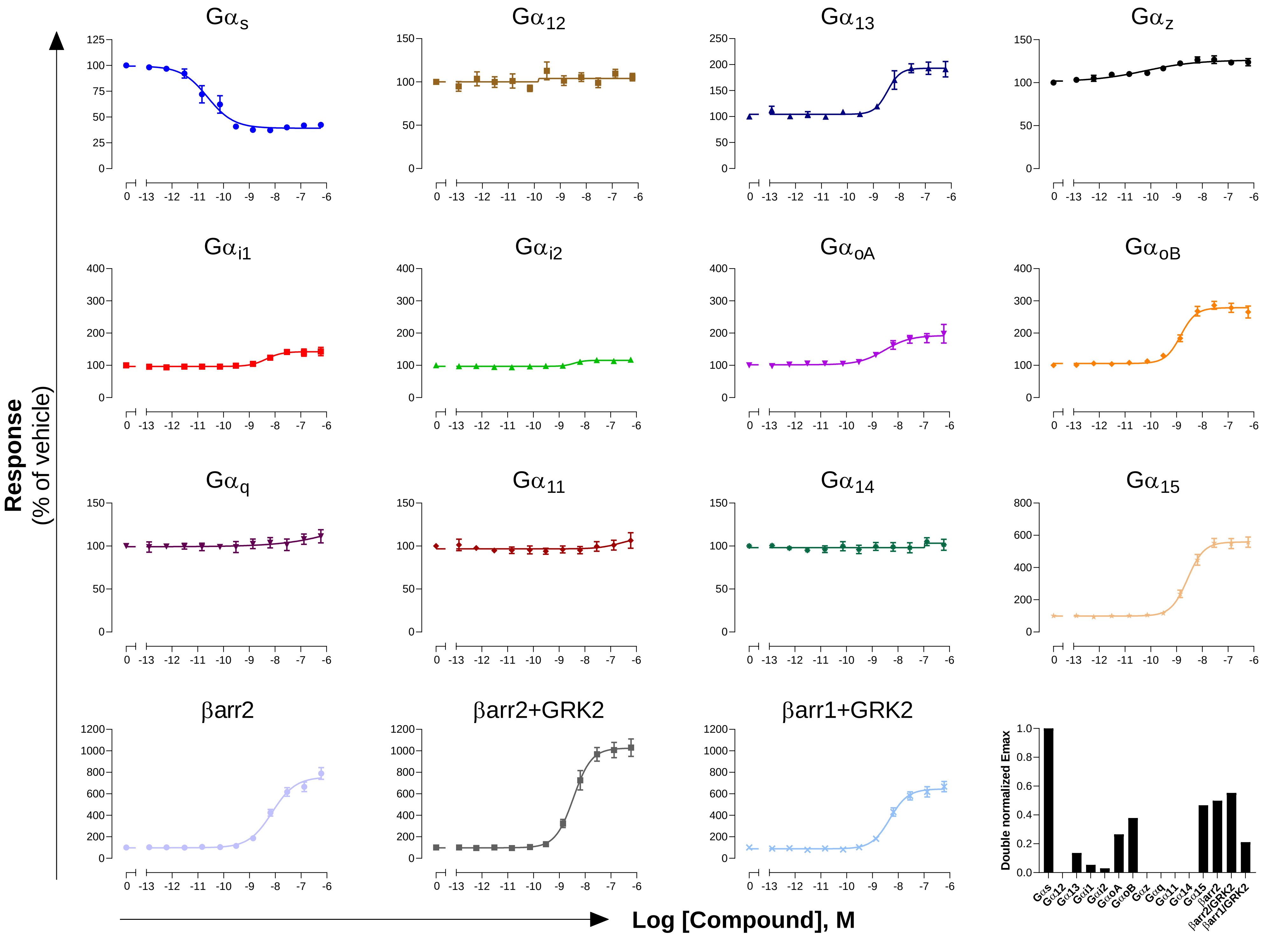

GPCR: GLP-2
Ligand: GLP-2 (1-33)

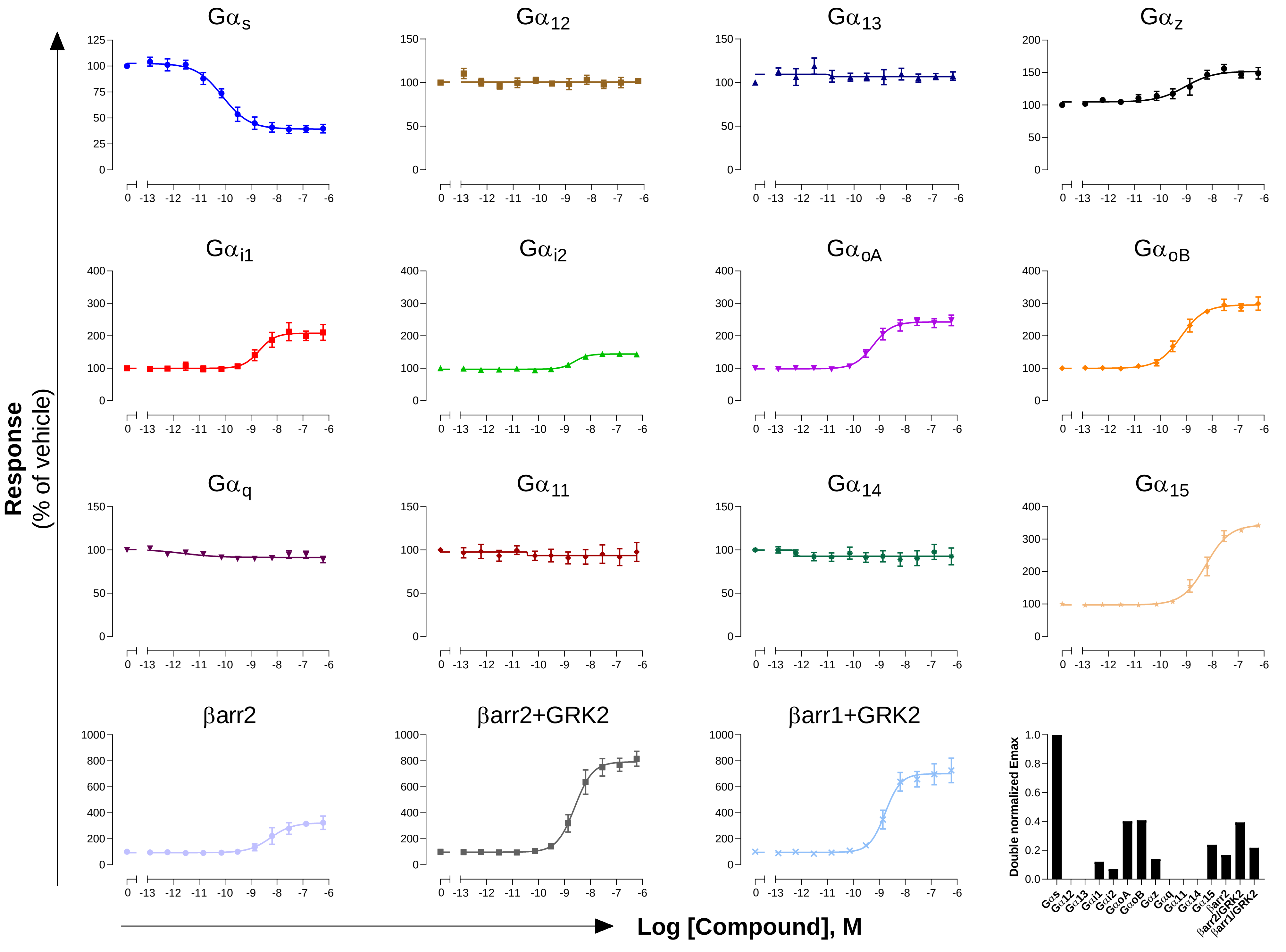

GPCR: GnRHR
Ligand: GnRH

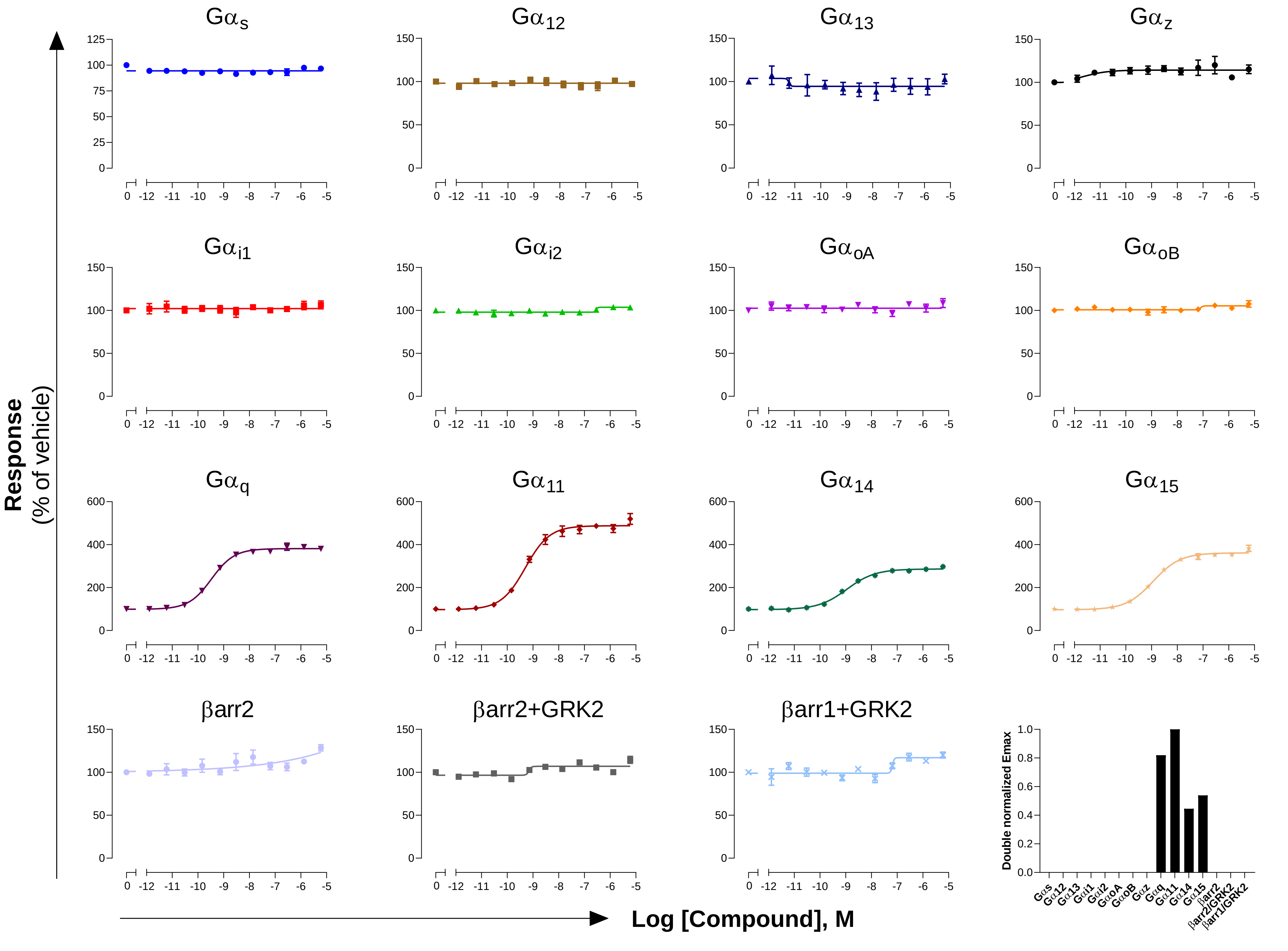

GPCR: GPBA
Ligand: Litocholic acid

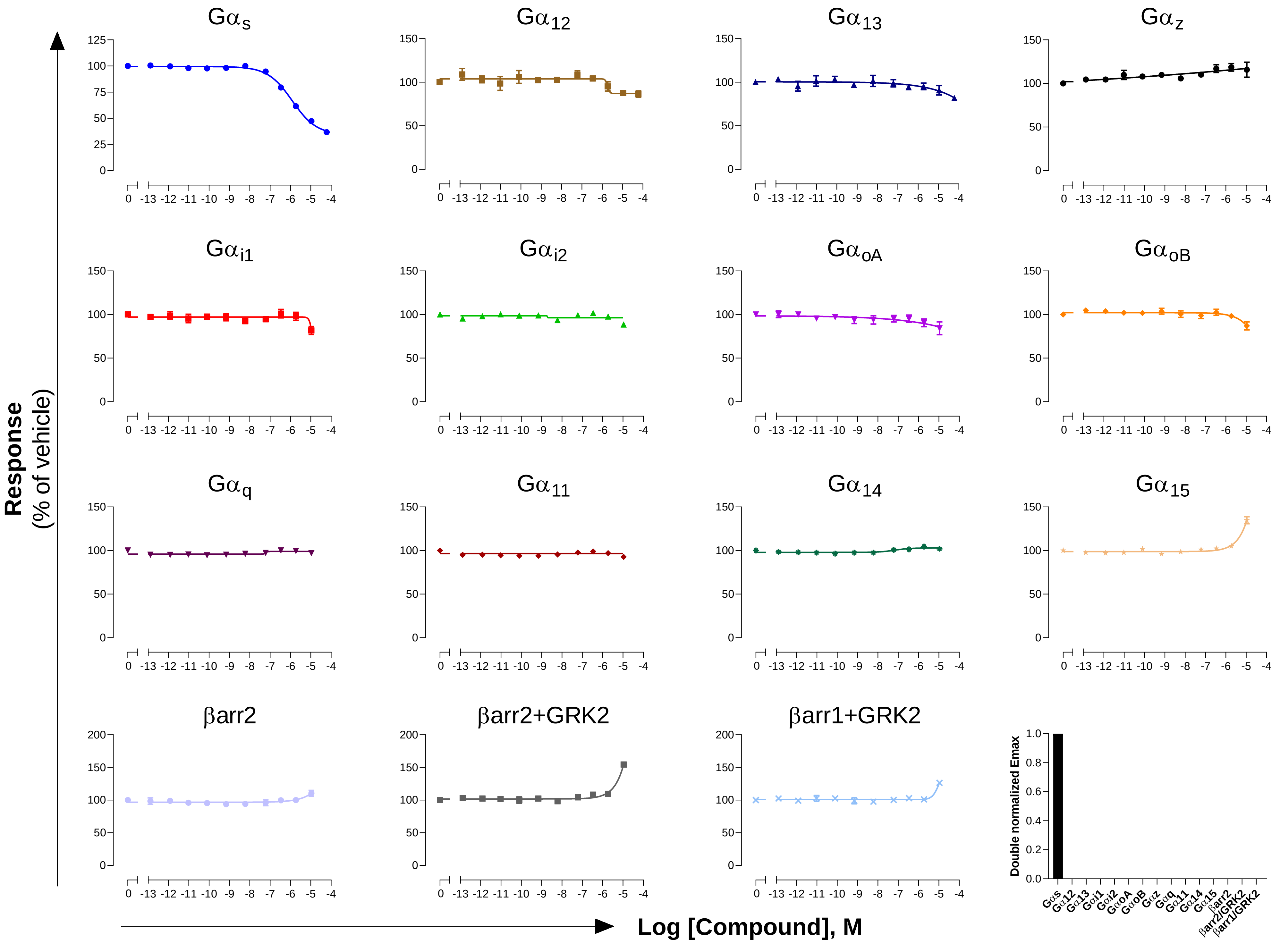

GPCR: GPR39
Ligand: Zn^{2+}

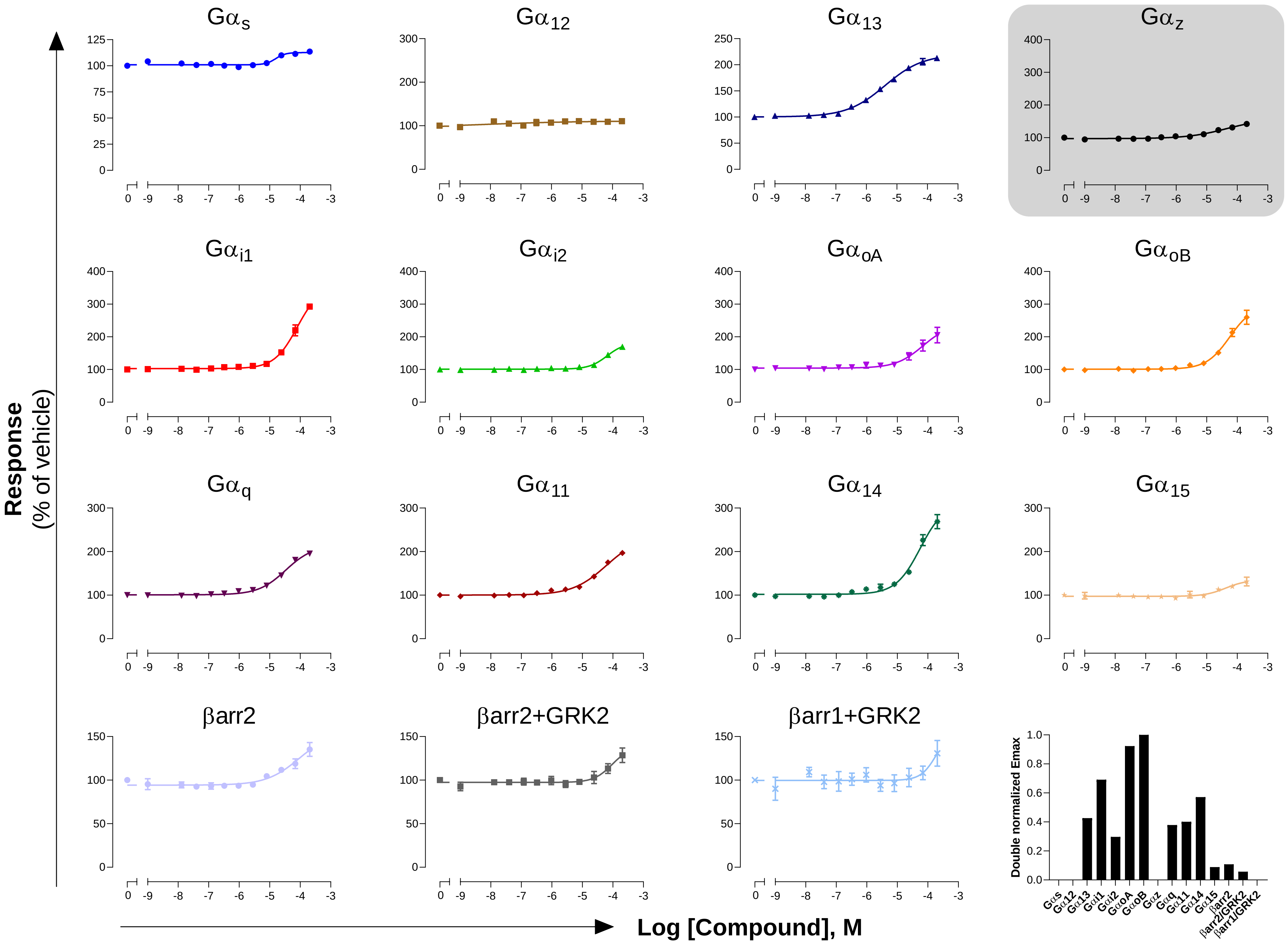

GPCR: GPR4
Ligand: Protons (pH)

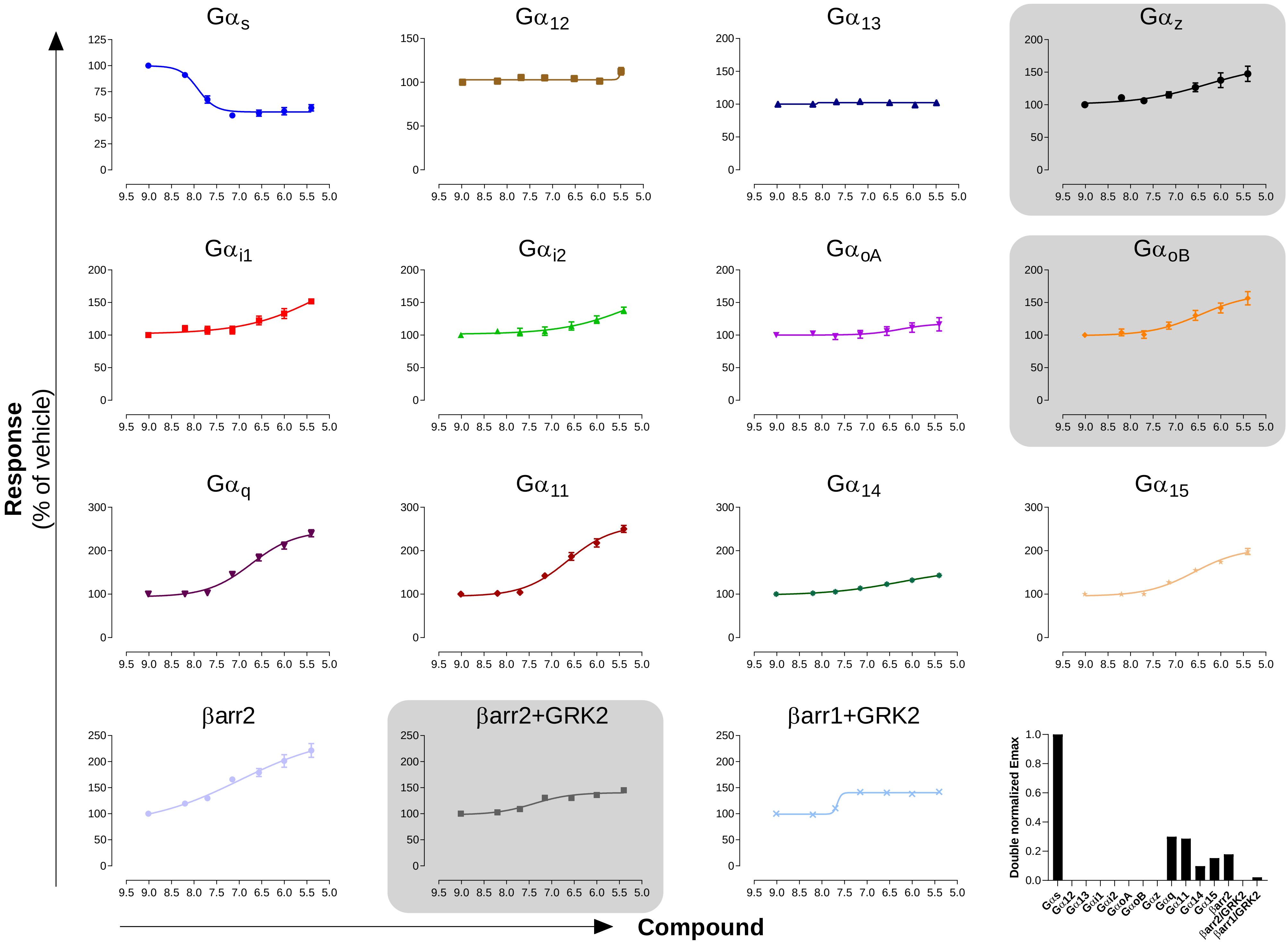

GPCR: GPR65
Ligand: protons (pH)

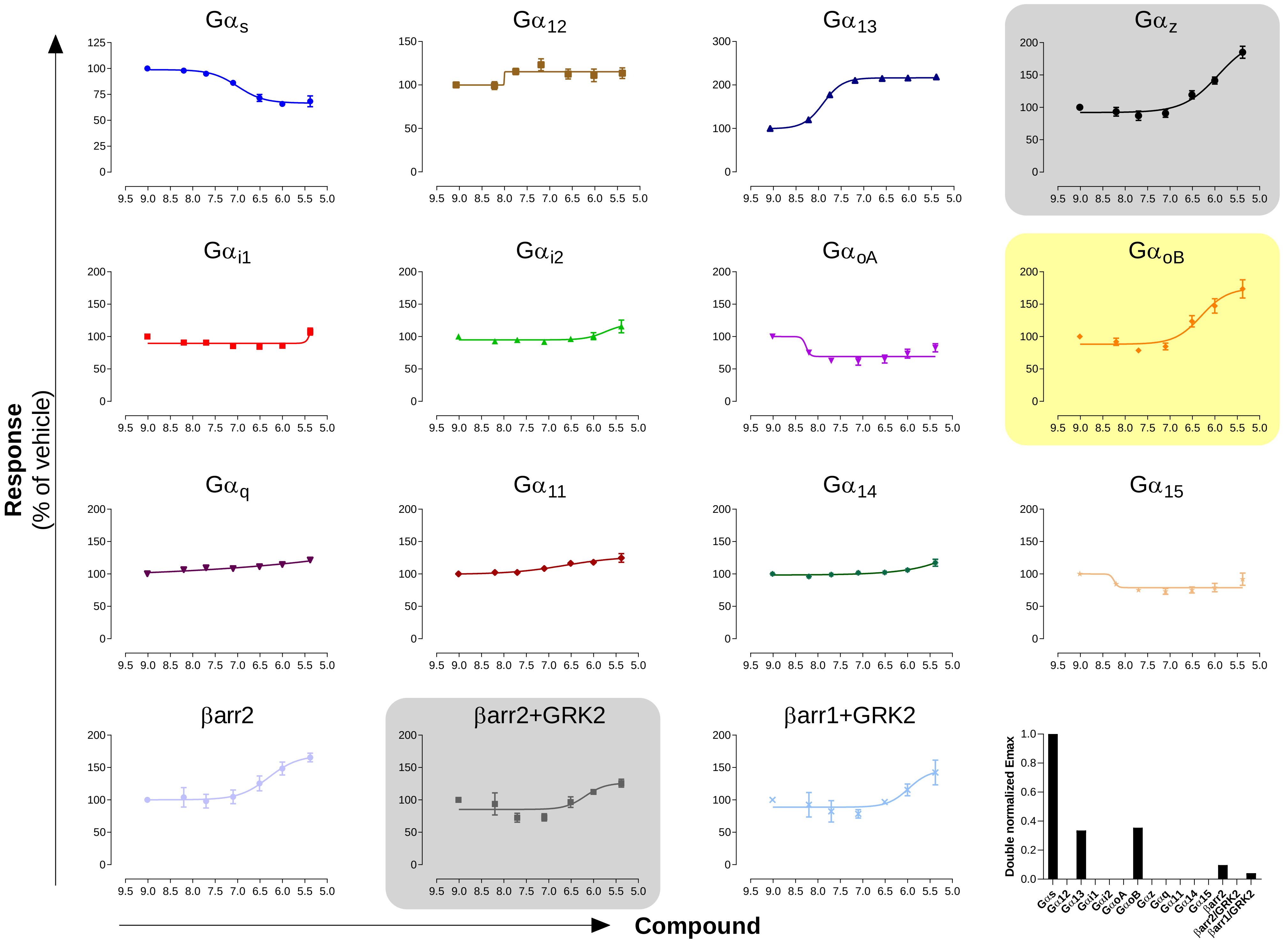

GPCR: GPR68
Ligand: Protons (pH)

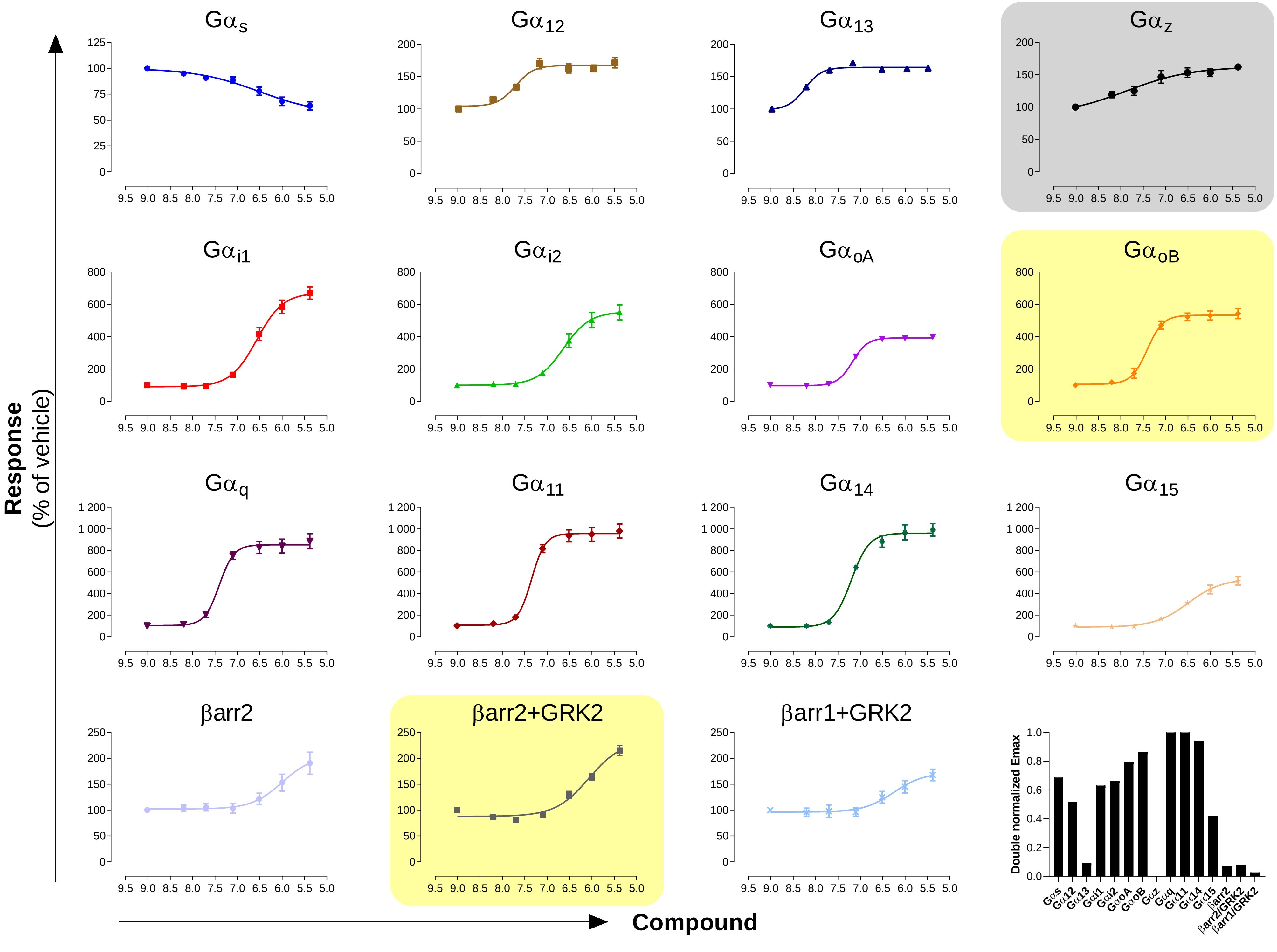

GPCR: GPR84
Ligand: Undecanoic acid

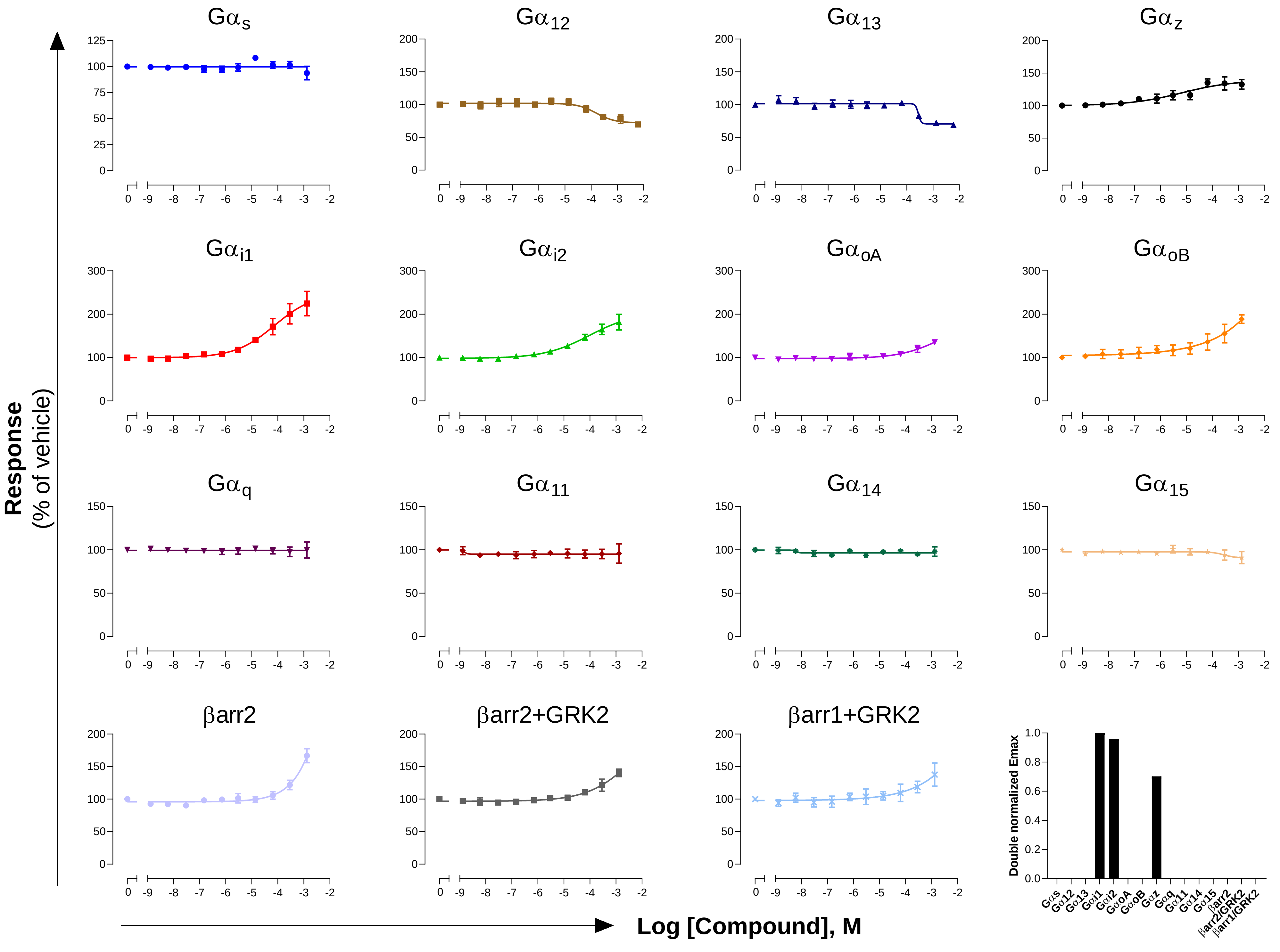

GPCR: GPR183
Ligand: 7α-25 dihydroxycholesterol

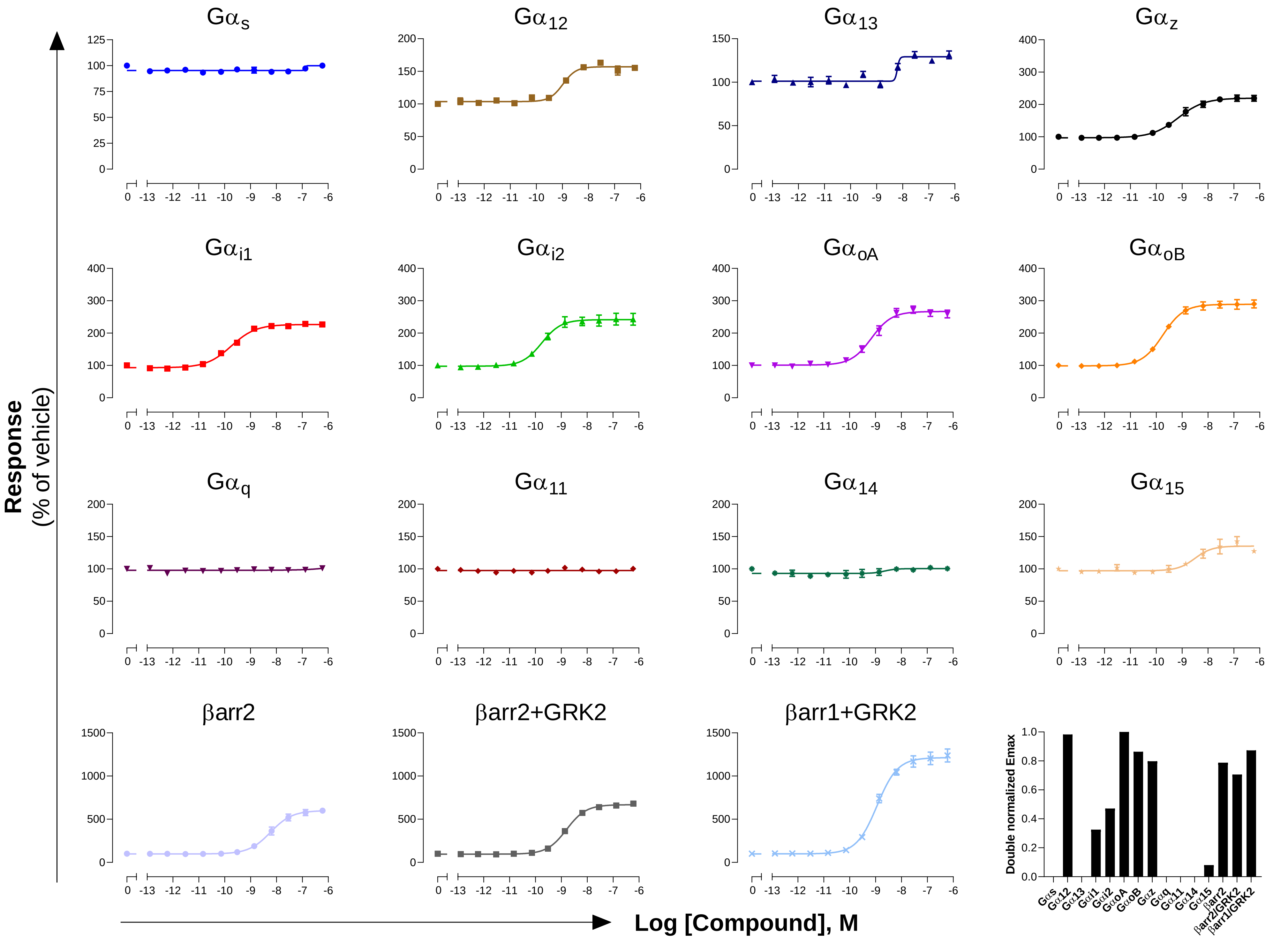

GPCR: H_1

Ligand: Histamine

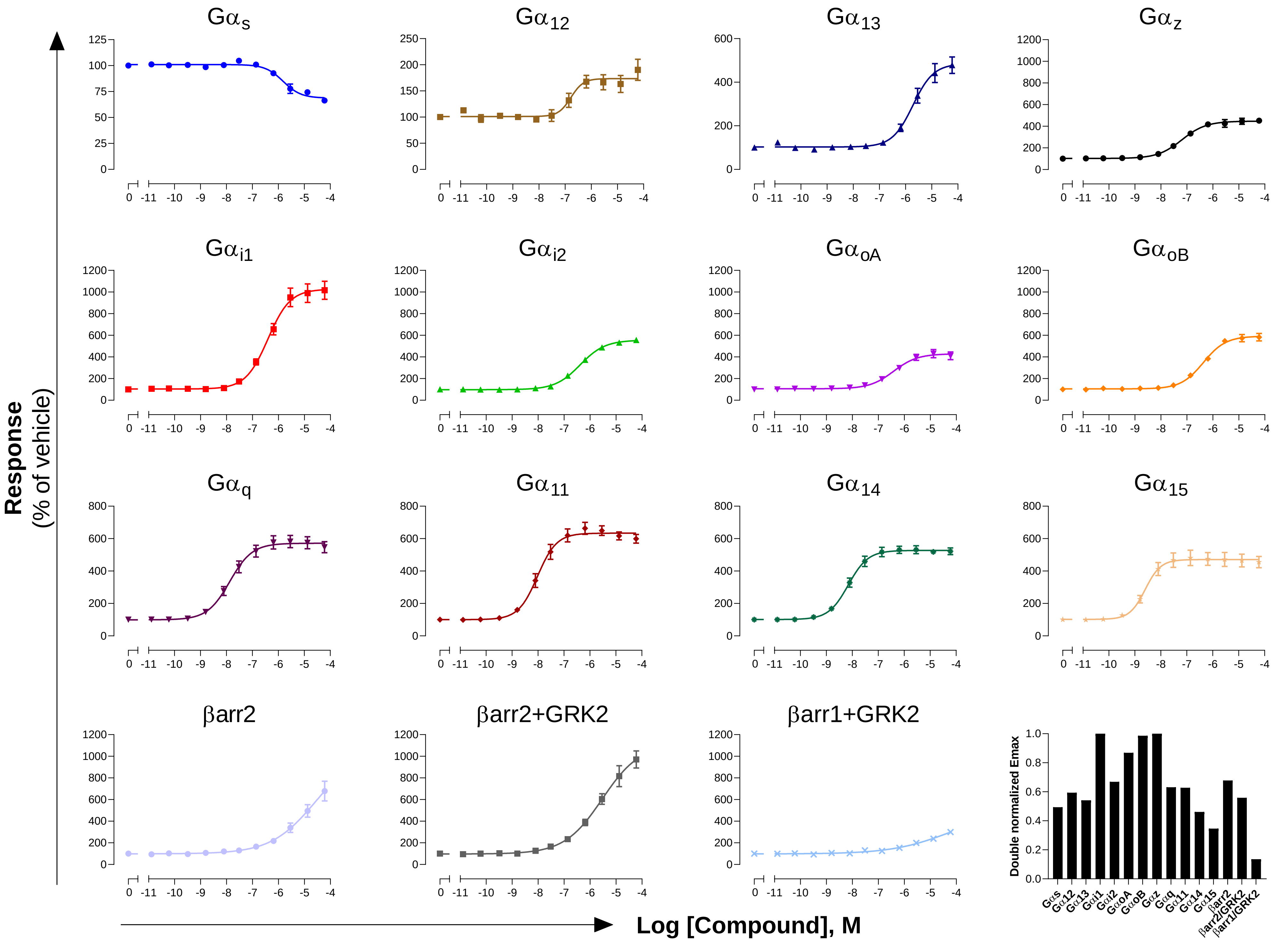

GPCR: H_2

Ligand: Histamine

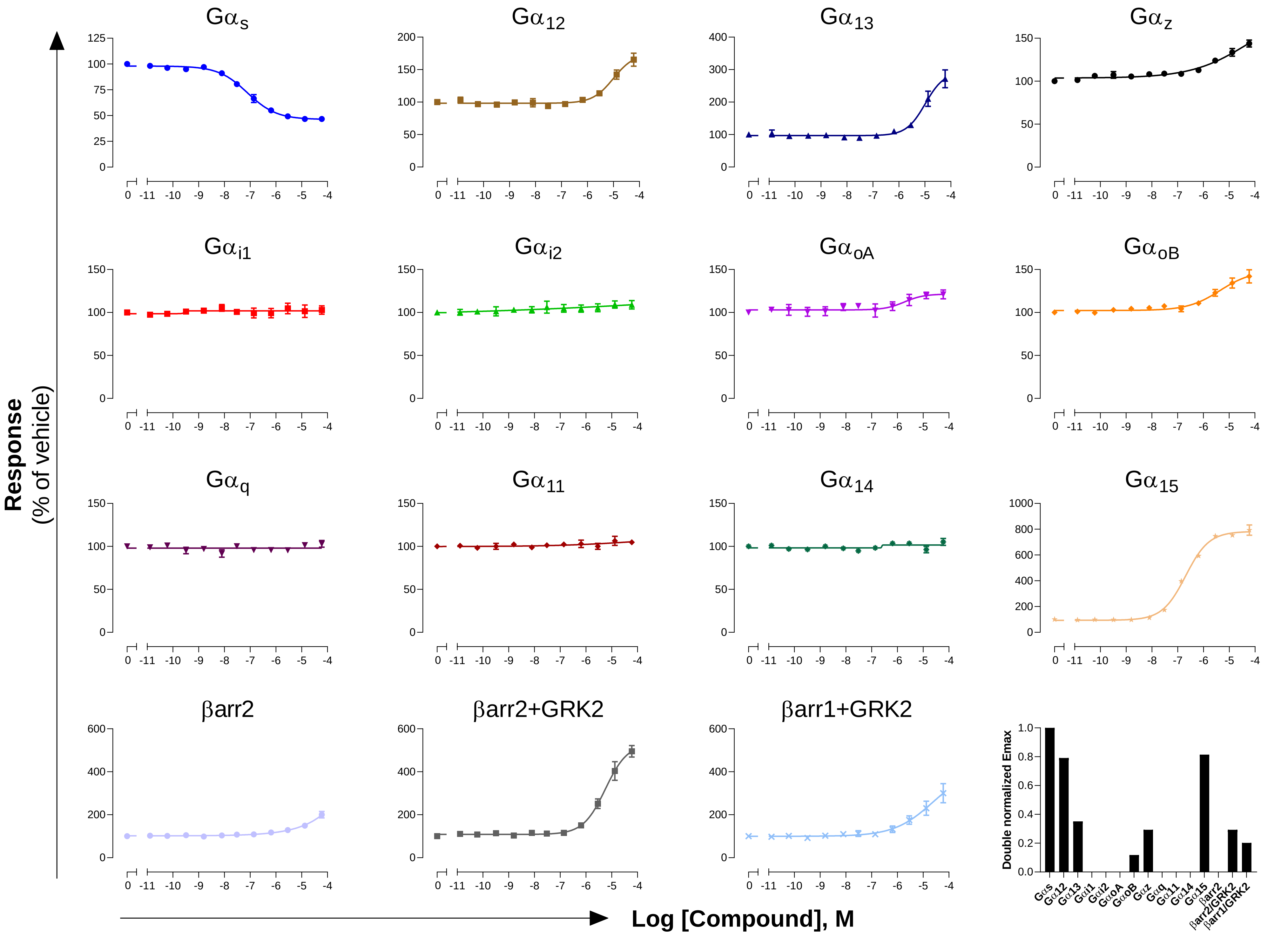

GPCR: HCA_2
Ligand: Nicotinic acid

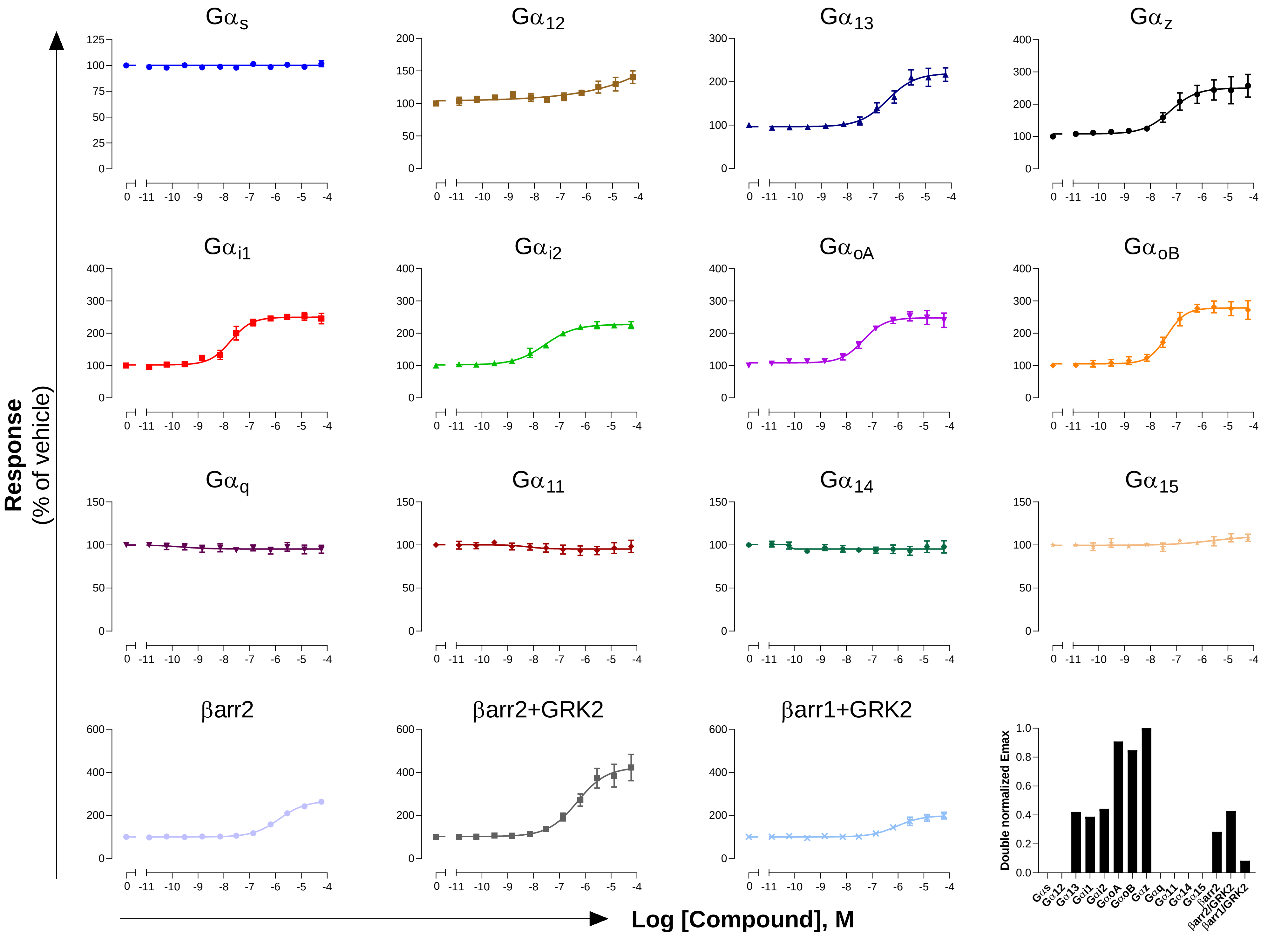

GPCR: HCA_3

Ligand: 3-hydroxyoctanoic acid (3-HOA)

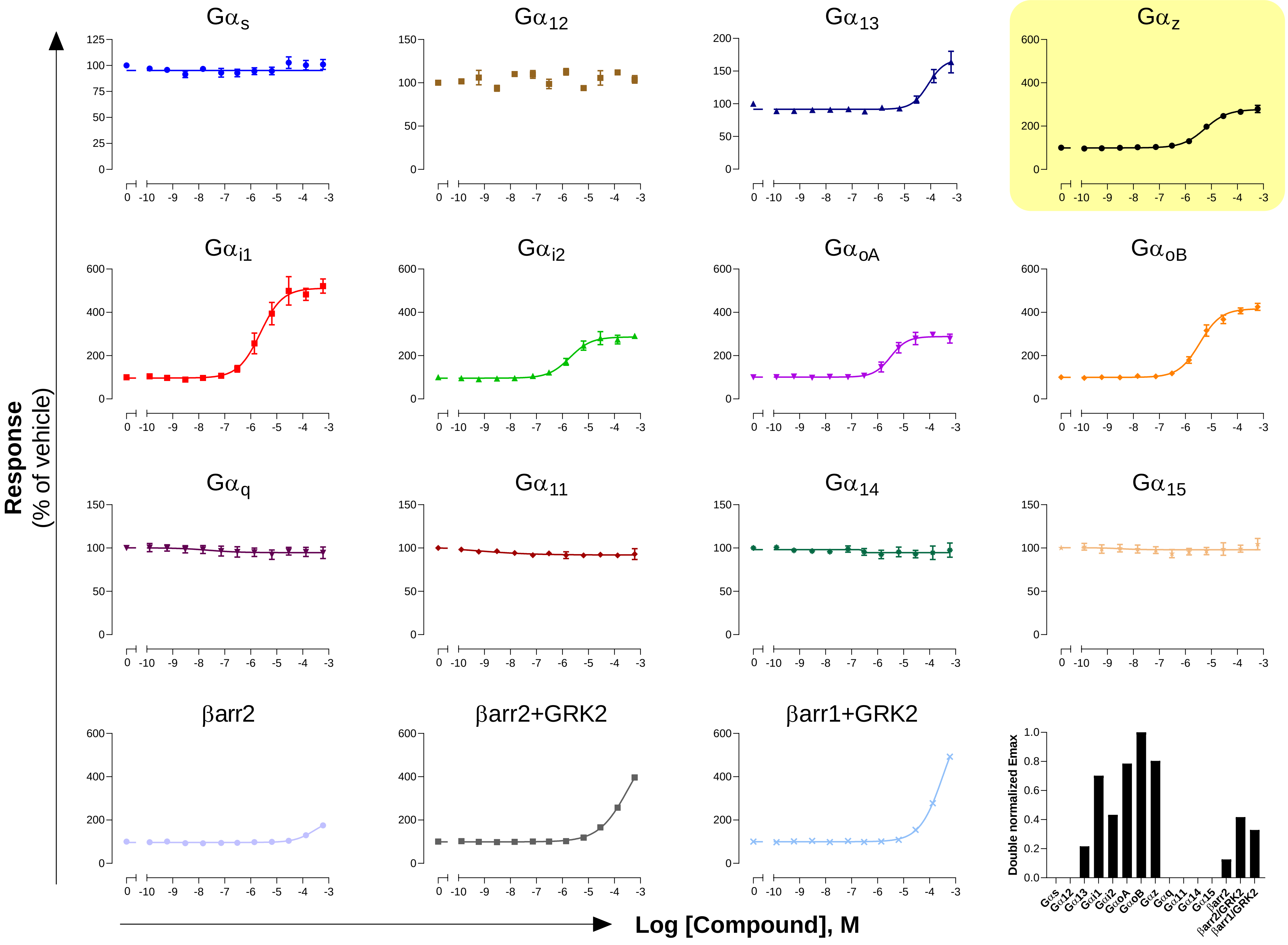

GPCR: κOR
Ligand: Dynorphin A

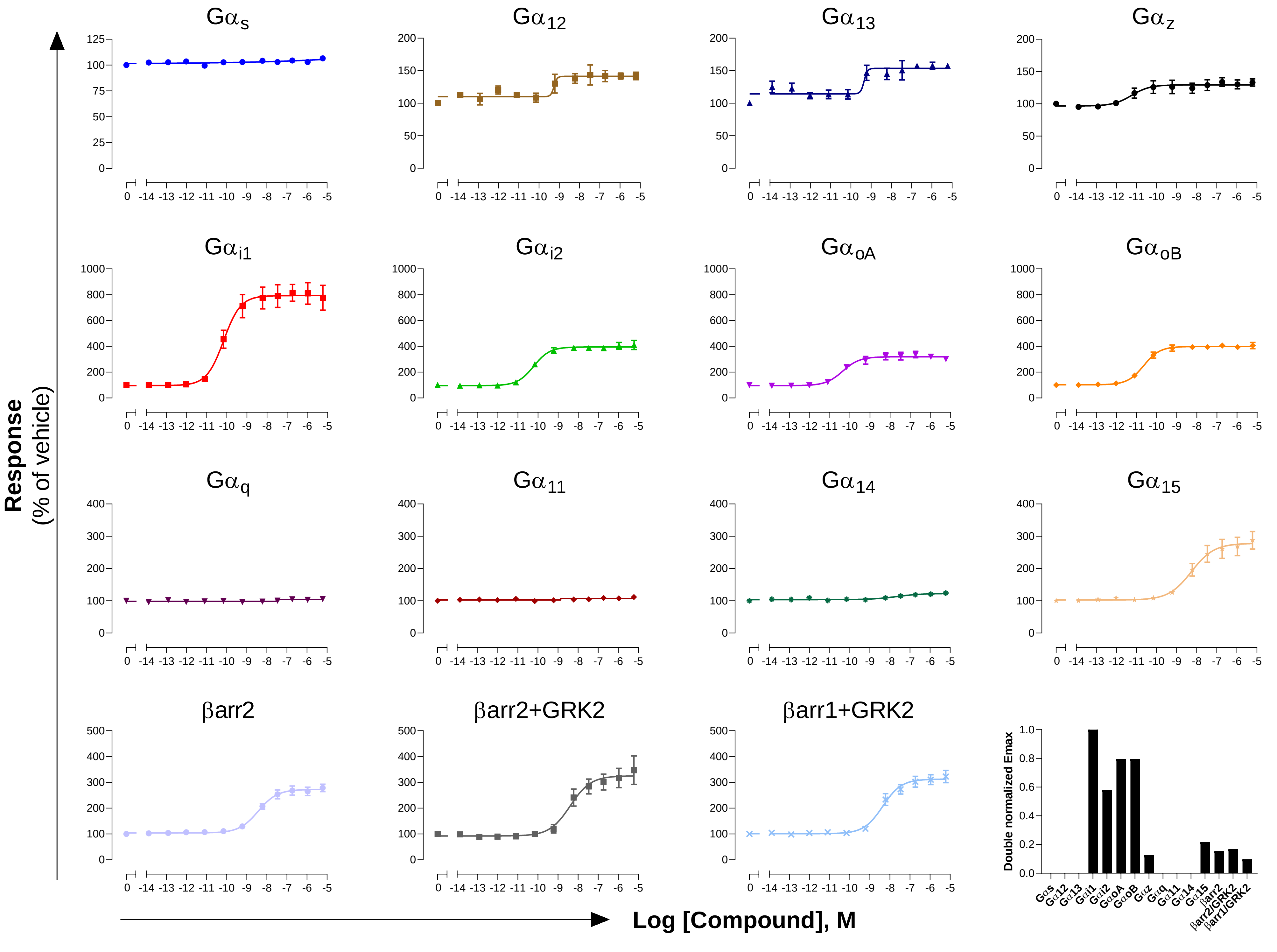

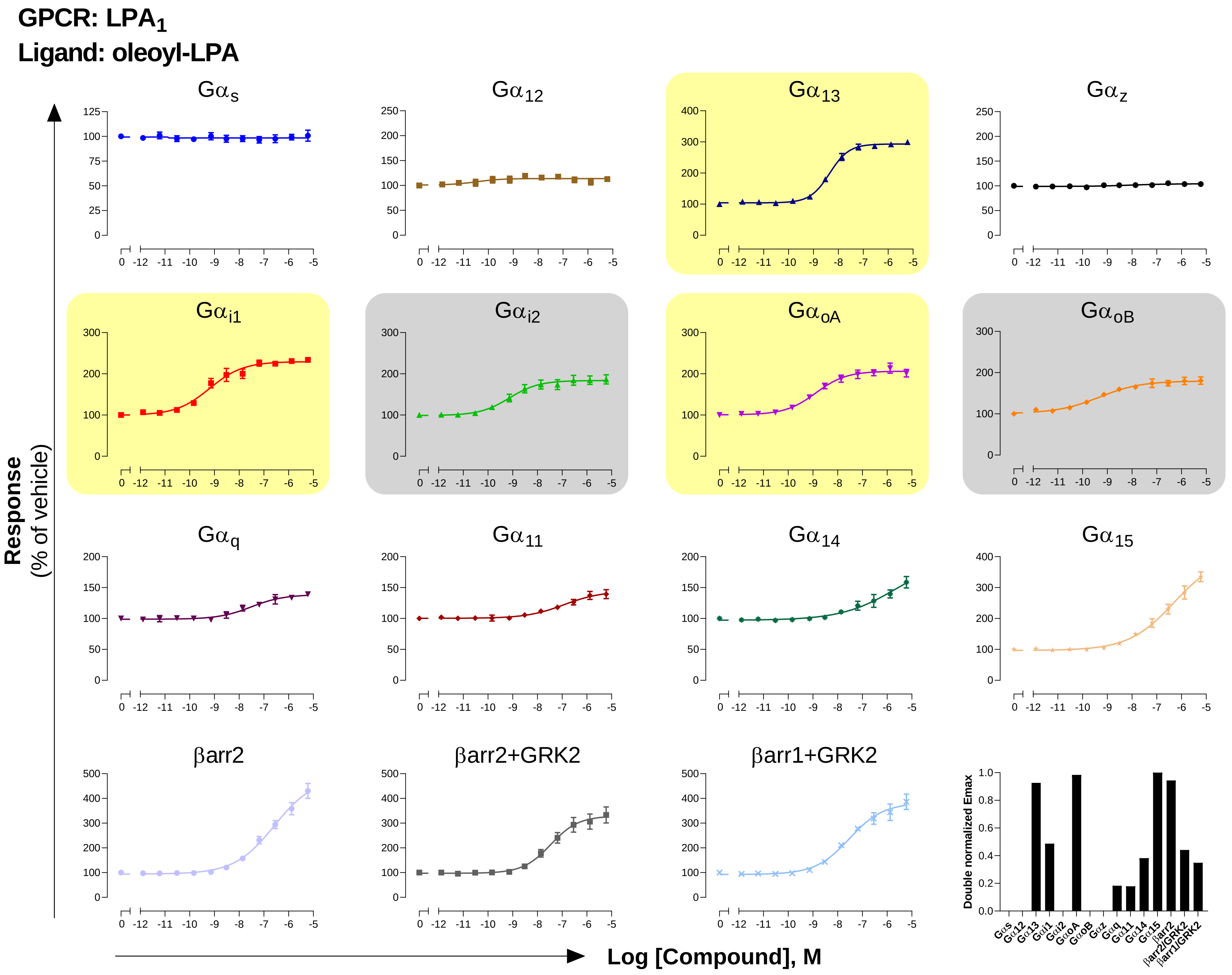
GPCR: LPA1
Ligand: oleoyl-LPA
Gαs
Gα12
Gα13
Gαz
Gαi1
Gαi2
GαoA
GαoB
Gαq
Gα11
Gα14
Gα15
βarr2
βarr2+GRK2
βarr1+GRK2
Response (% of vehicle)
Log [Compound], M
Double normalized Emax
Gαs Gα12 Gα13 Gαi1 Gαi2 GαoA GαoB Gαz Gαq Gα11 Gα14 Gα15 βarr2 βarr2/GRK2 βarr1/GRK2

GPCR: LPA_2
Ligand: oleoyl-LPA

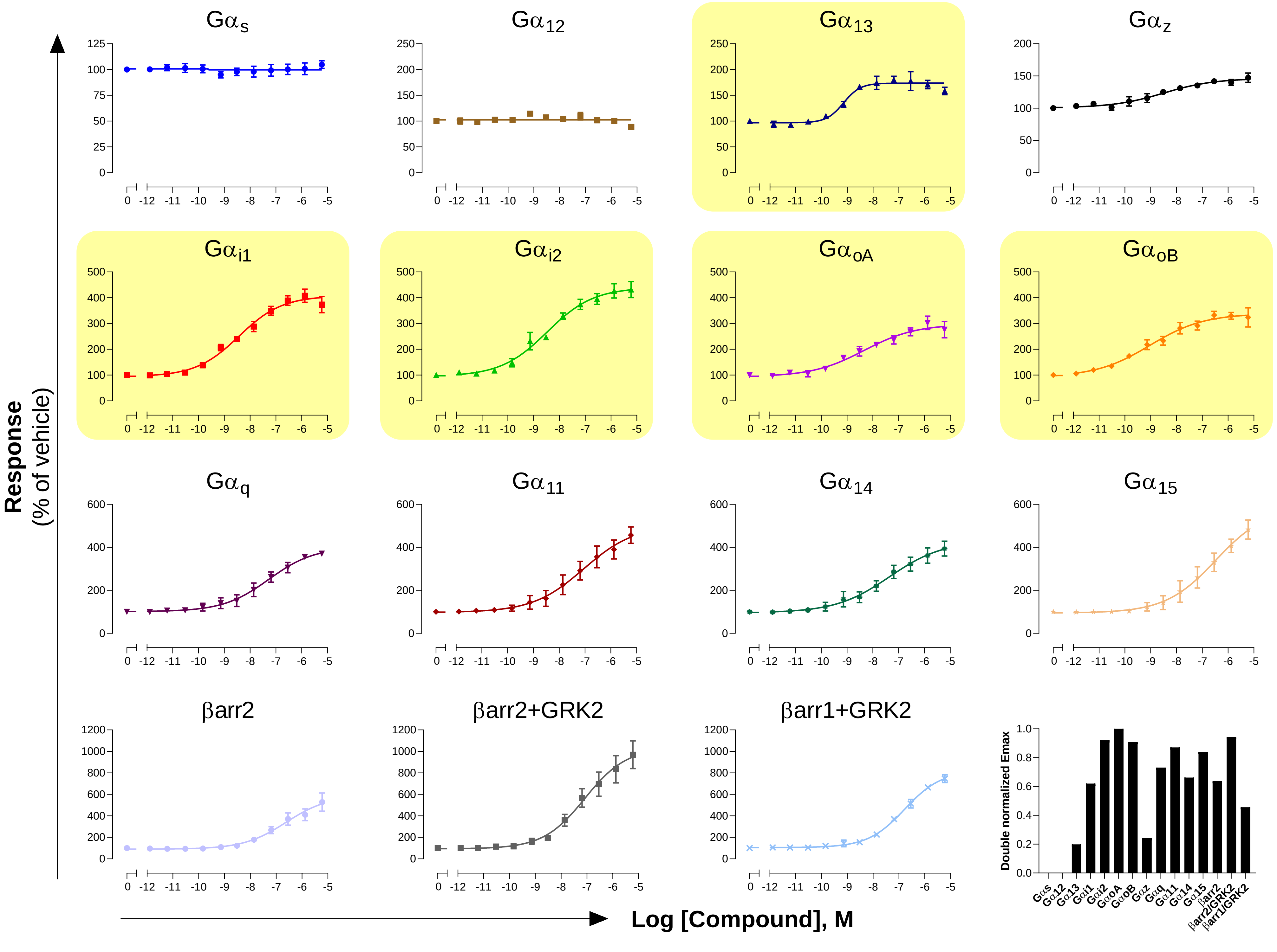

GPCR: M_1

Ligand: Acetylcholine

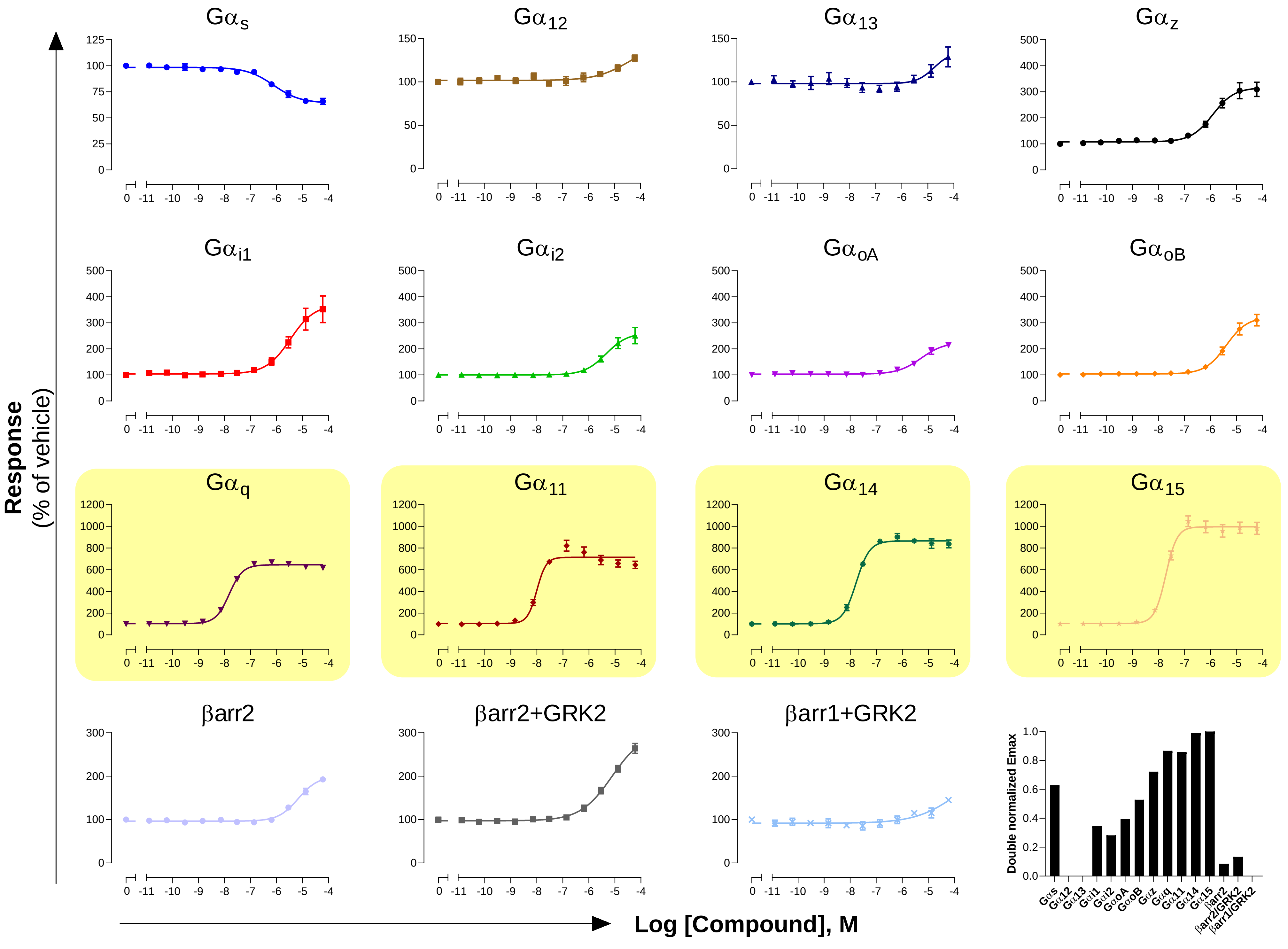

GPCR: M_2
Ligand: Acetylcholine

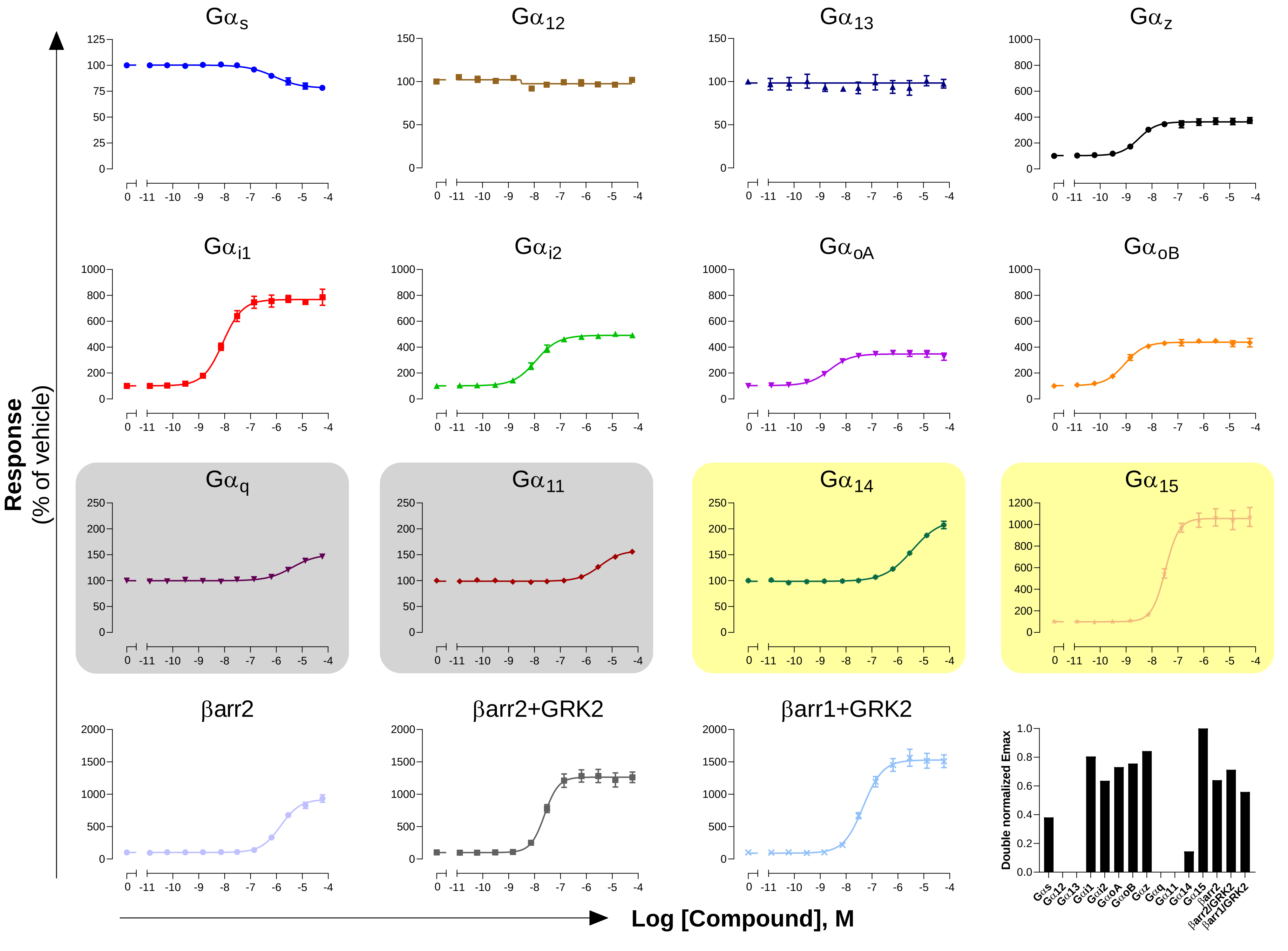

GPCR: M_3
Ligand: Acetylcholine

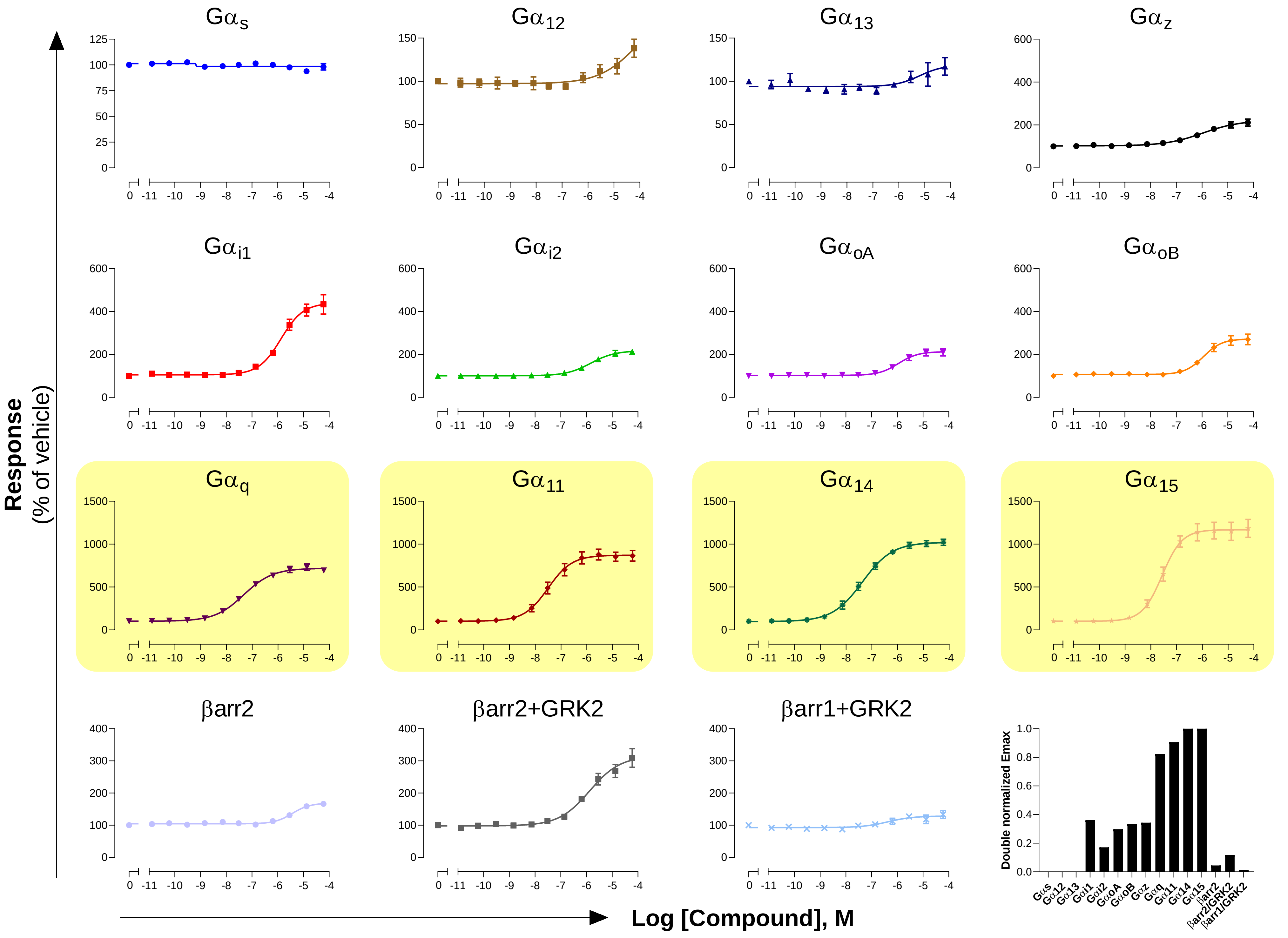

GPCR: M_4
Ligand: Acetylcholine

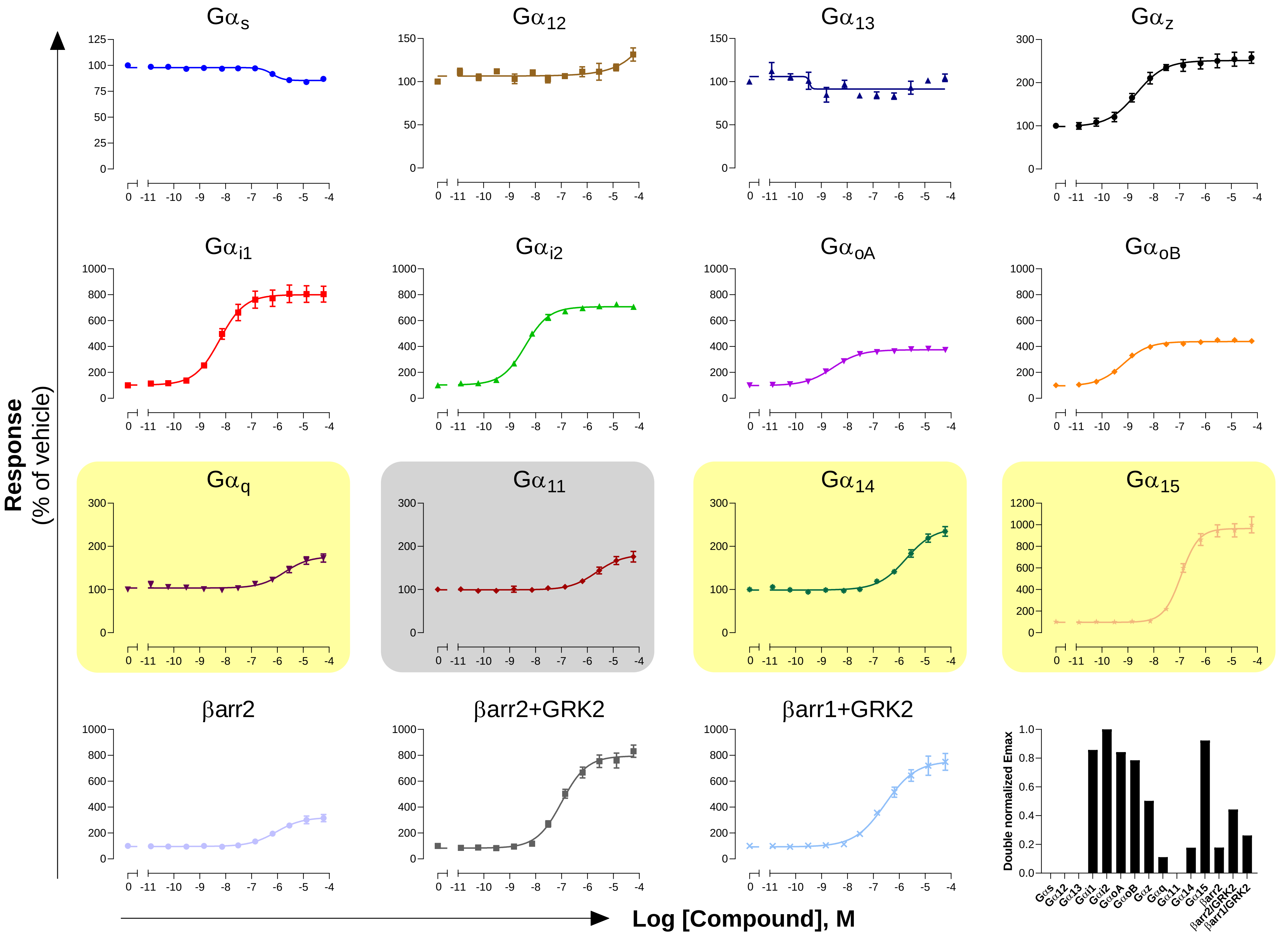

GPCR: MC3R
Ligand: γ-MSH

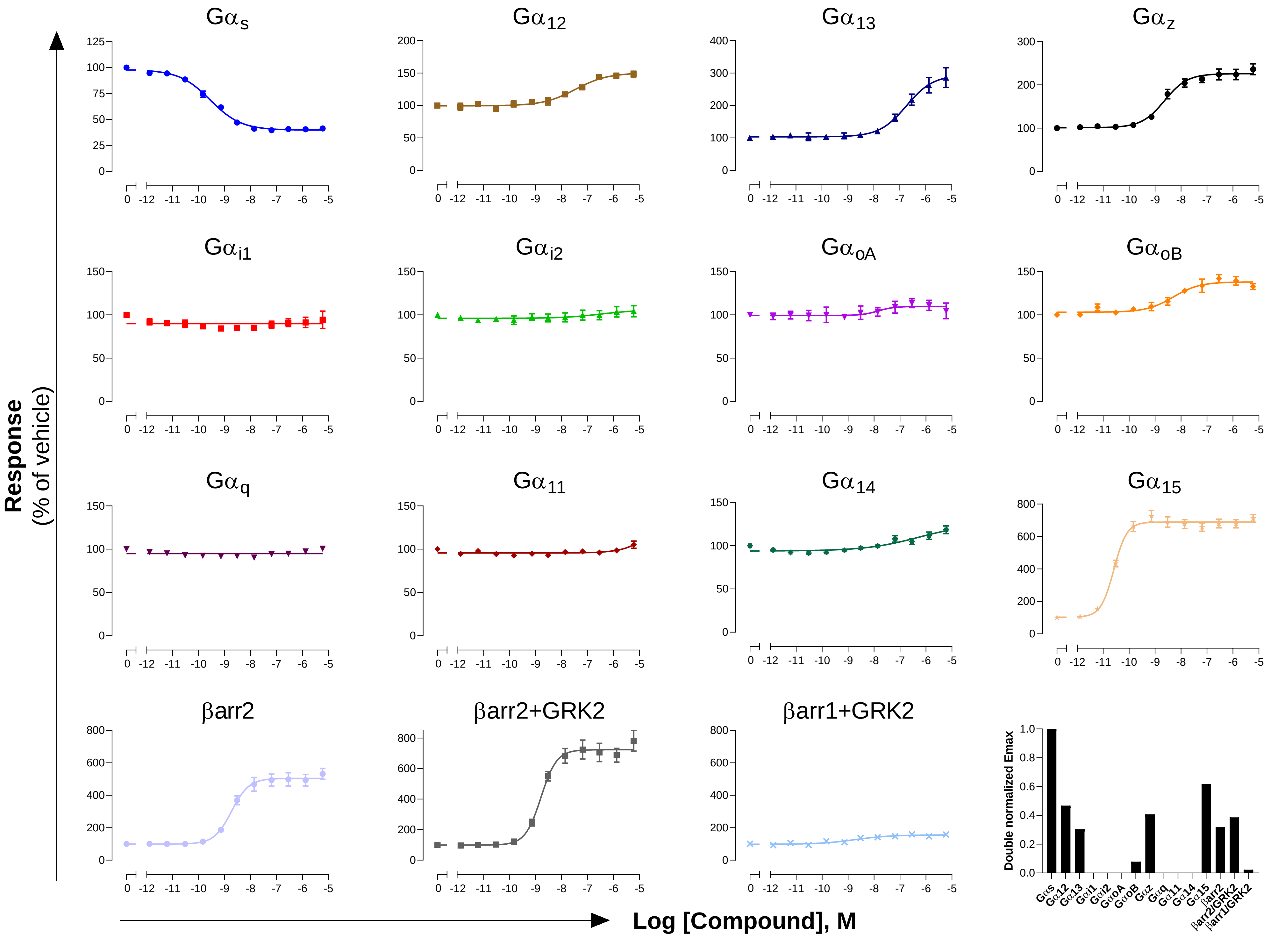

GPCR: MC4R
Ligand: α-MSH

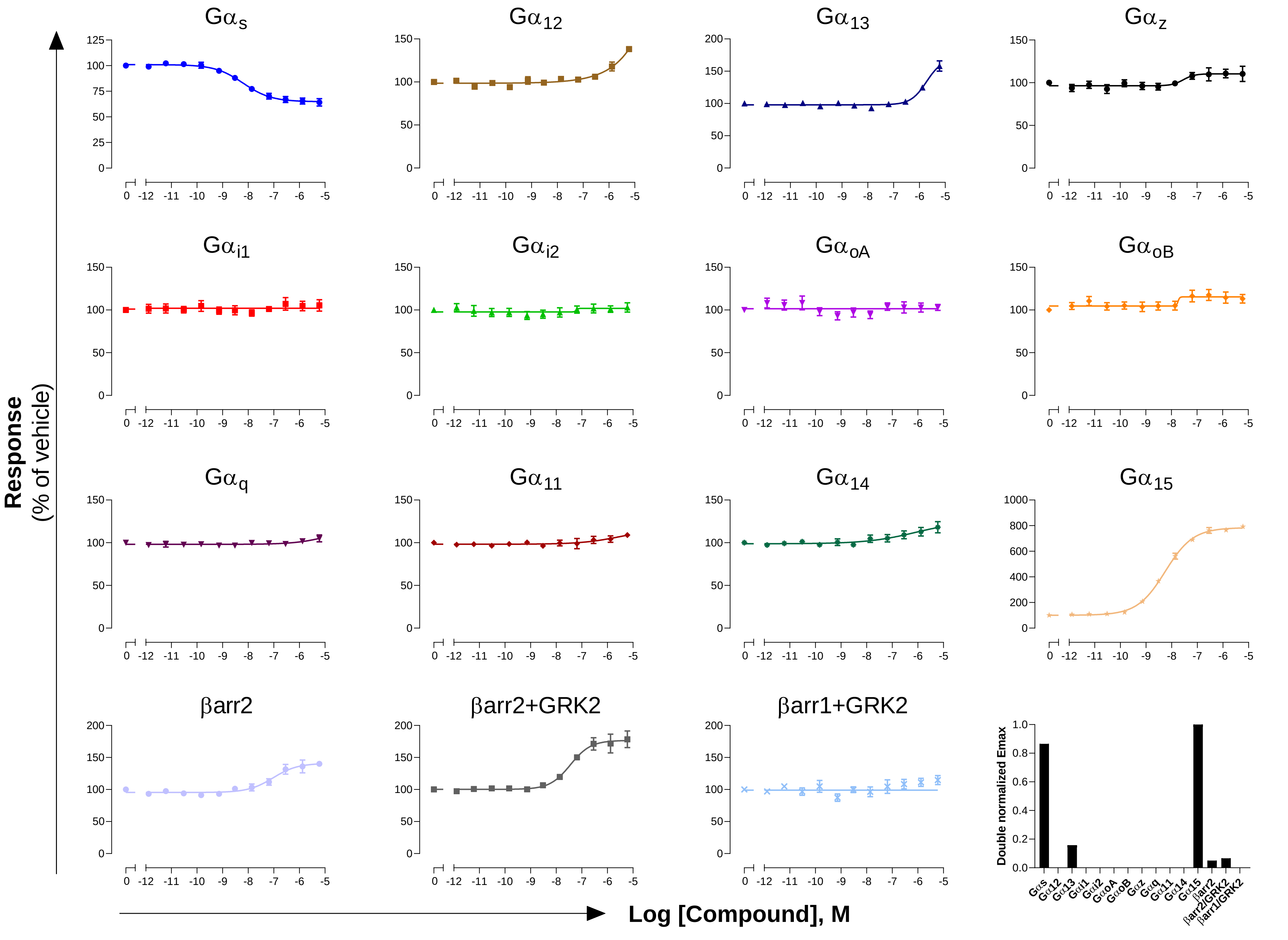

GPCR: mGluR2
Ligand: Glutamate

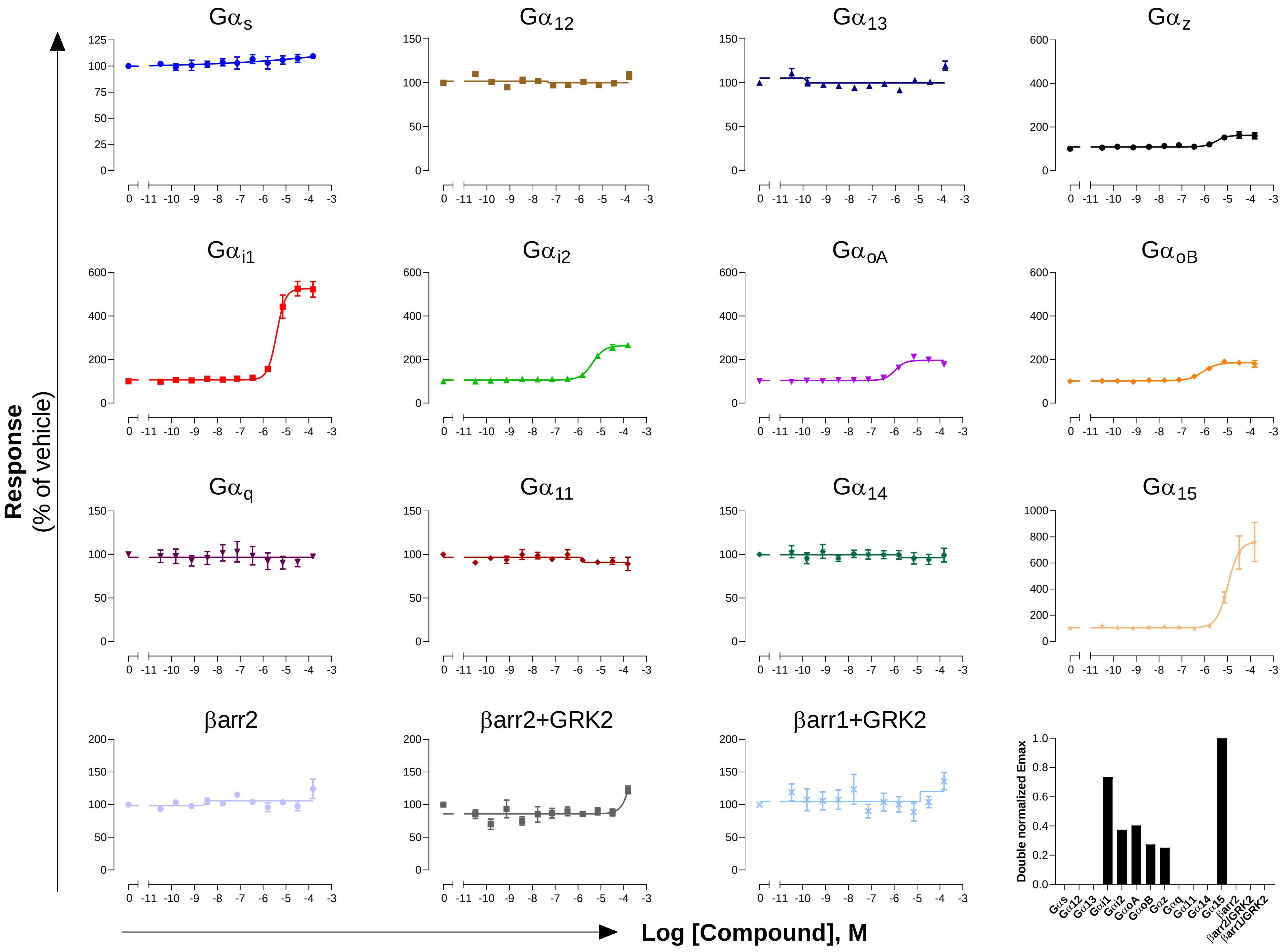

GPCR: mGluR4
Ligand: Glutamate

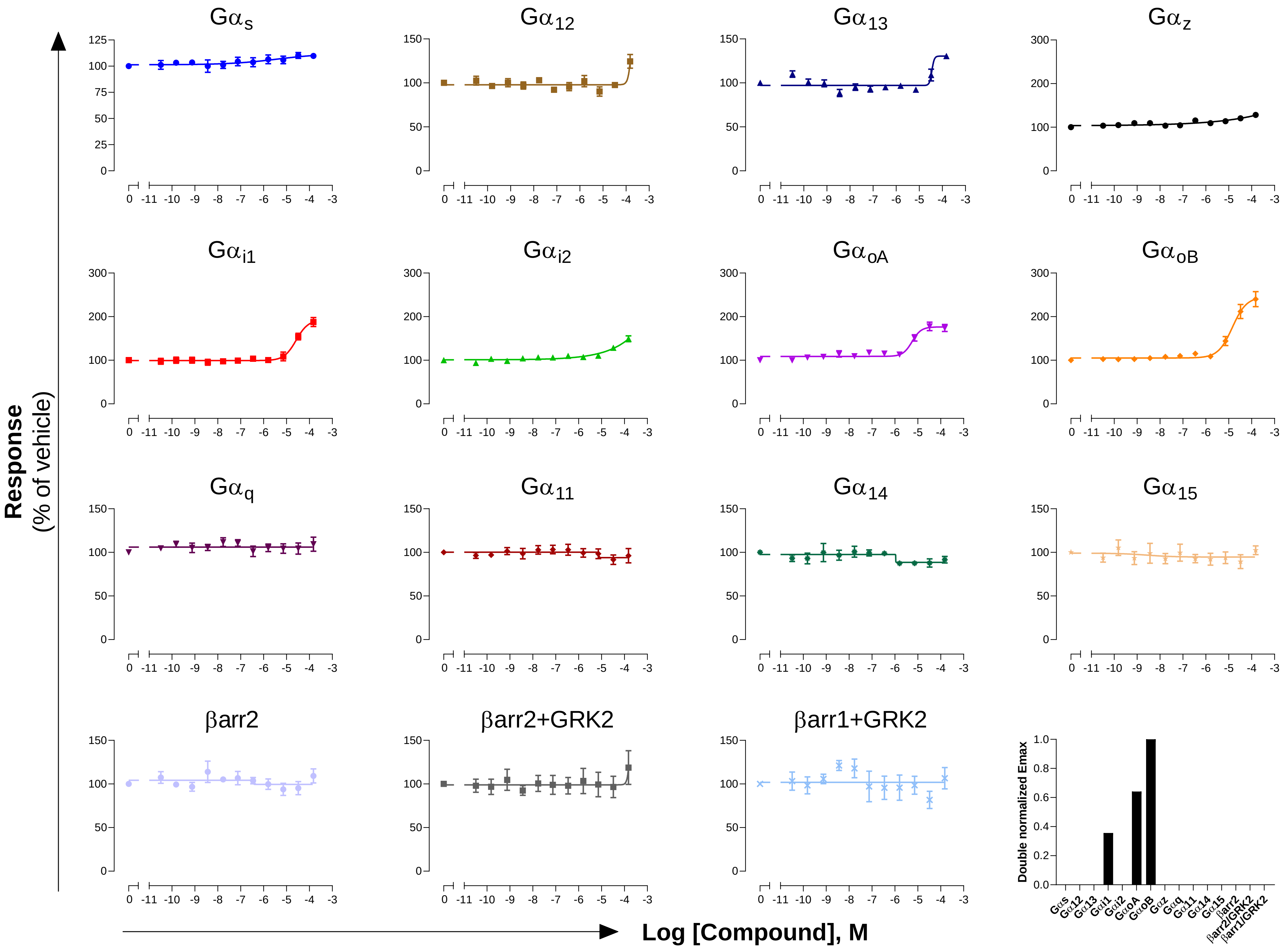

GPCR: mGluR5
Ligand: Glutamate

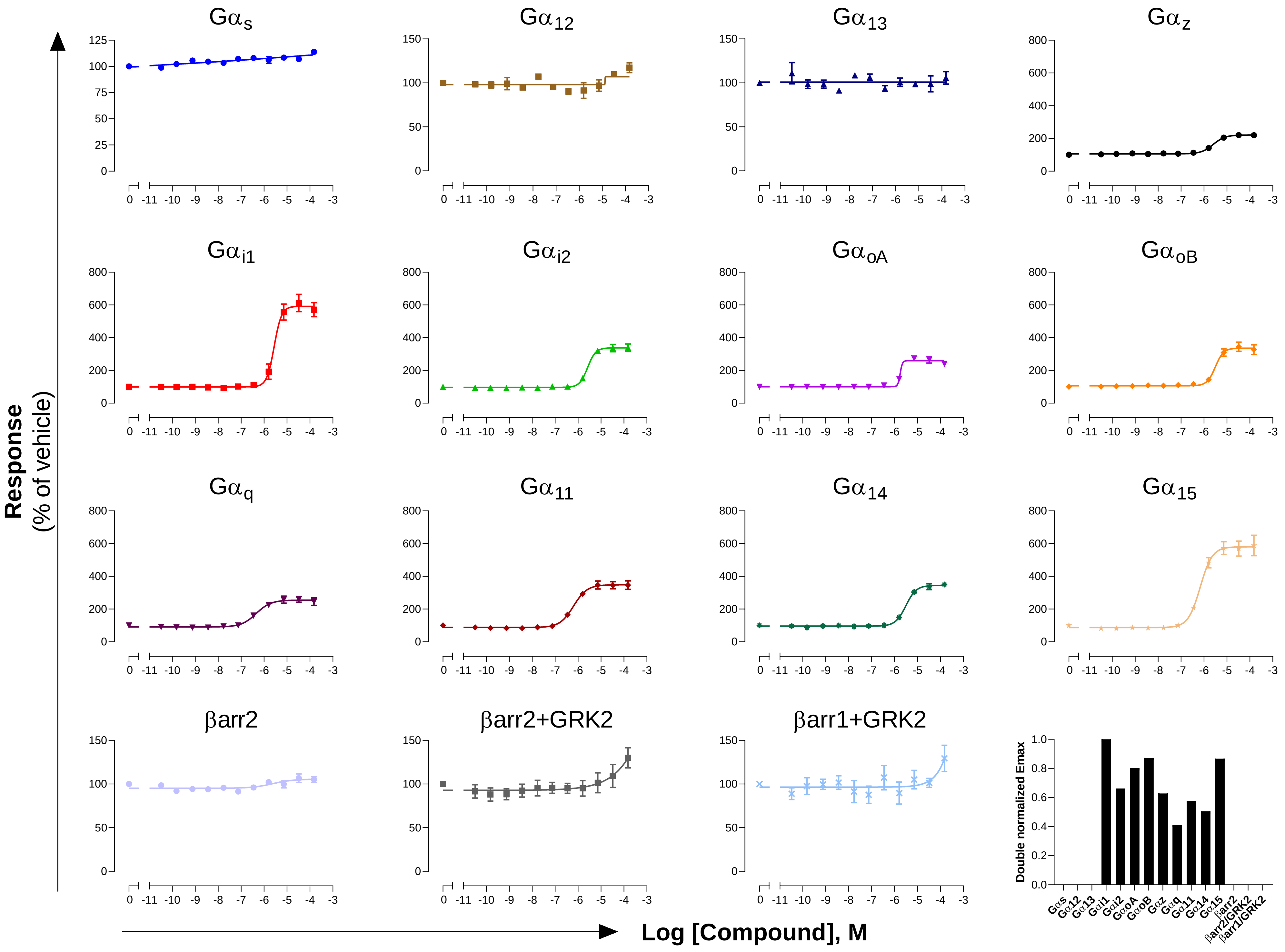

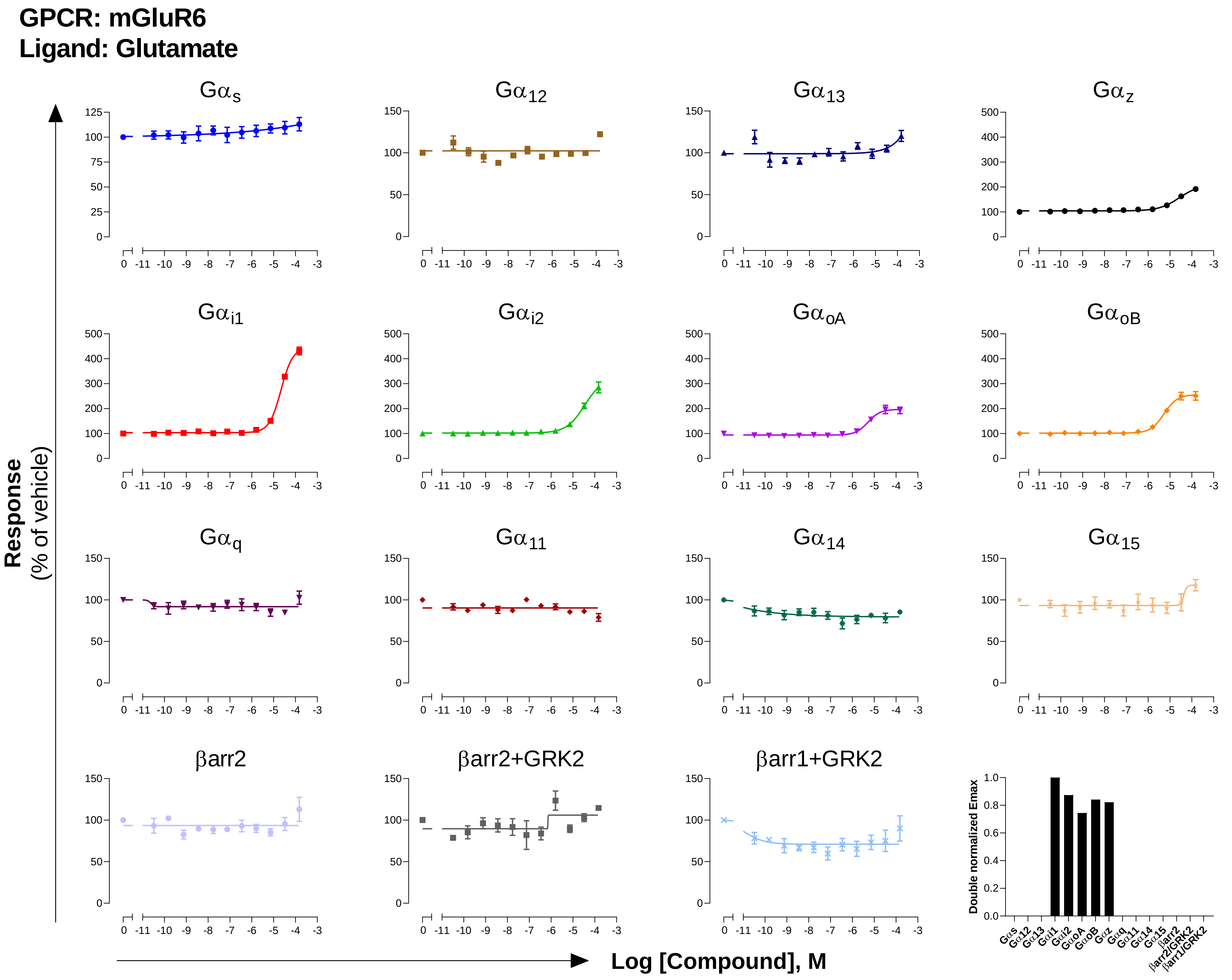
GPCR: mGluR6
Ligand: Glutamate
Gαs
Gα12
Gα13
Gαz
Gαi1
Gαi2
GαoA
GαoB
Gαq
Gα11
Gα14
Gα15
βarr2
βarr2+GRK2
βarr1+GRK2
Response
(% of vehicle)
Log [Compound], M
Double normalized Emax

GPCR: mGluR8
Ligand: Glutamate

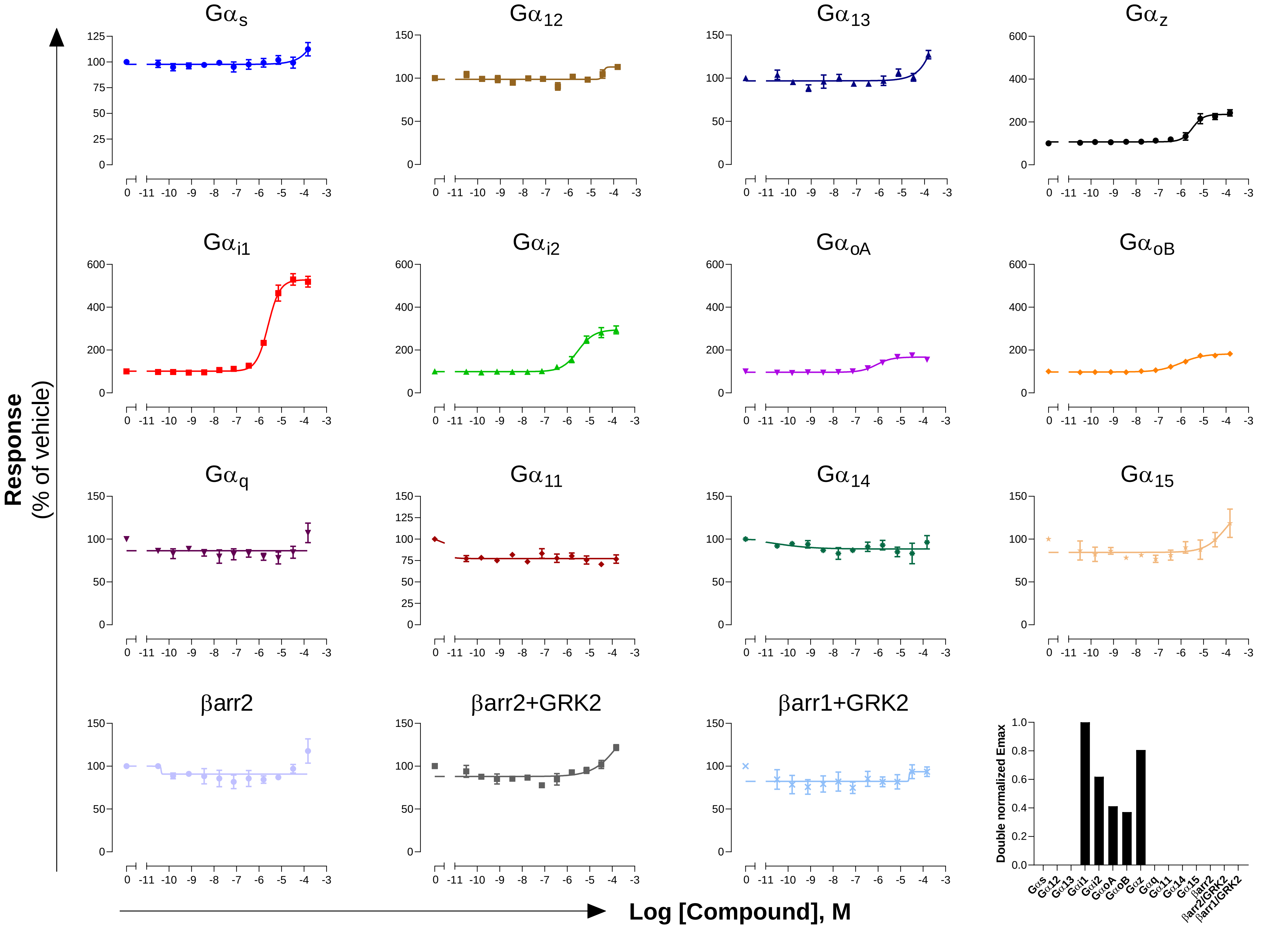

GPCR: μOR
Ligand: DAMGO

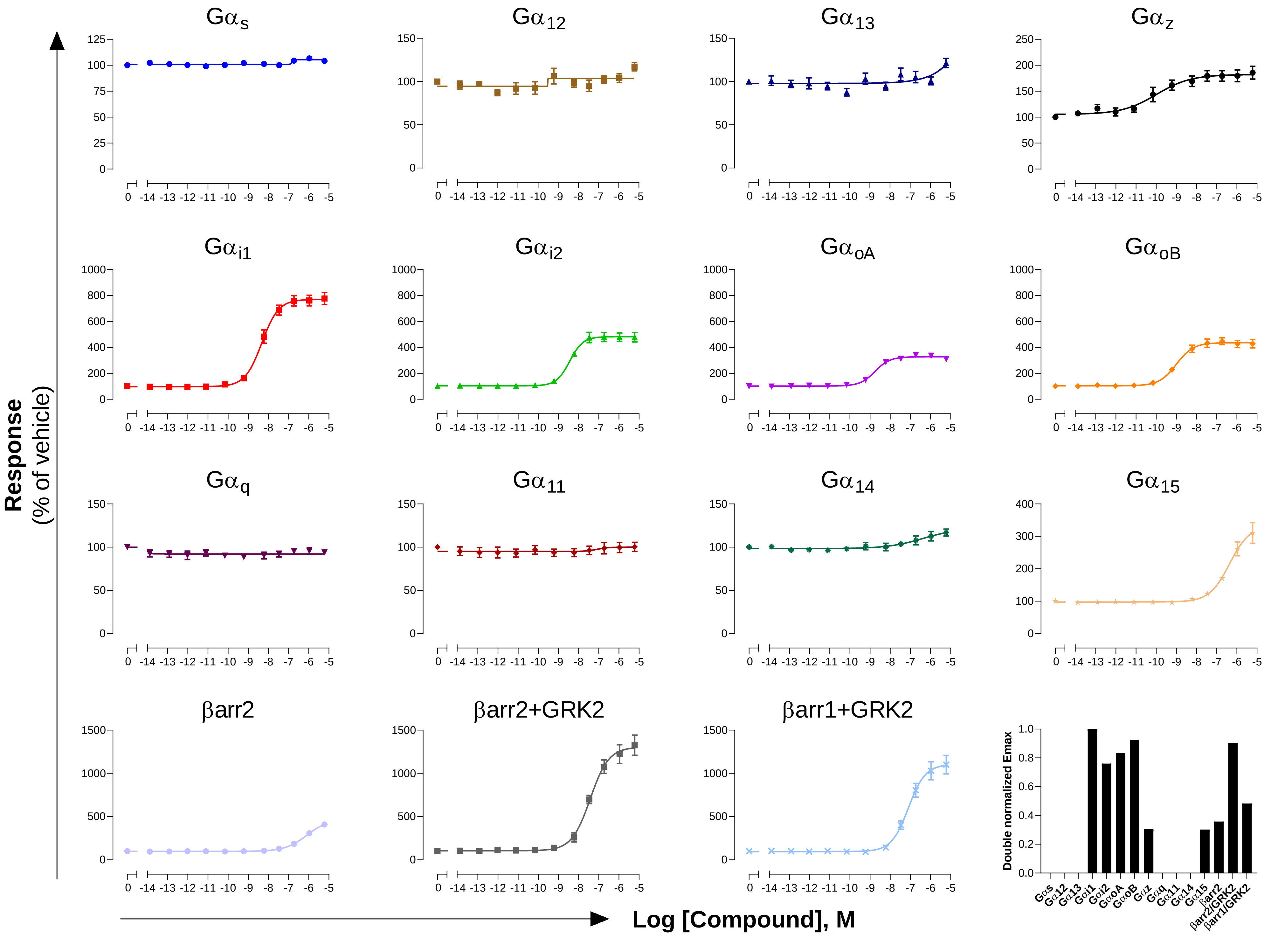

GPCR: MT_1

Ligand: Melatonin

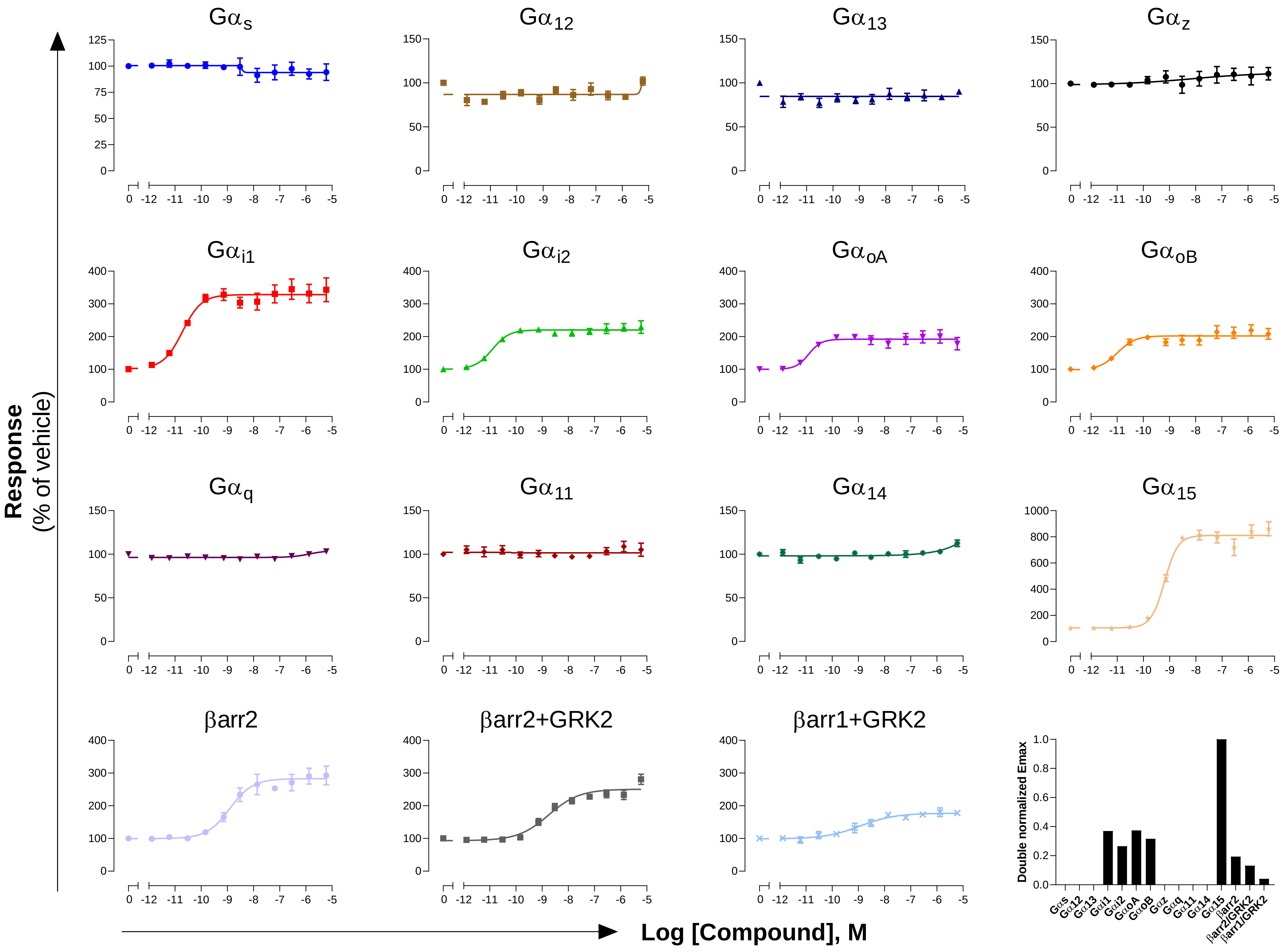

GPCR: MT_2

Ligand: Melatonin

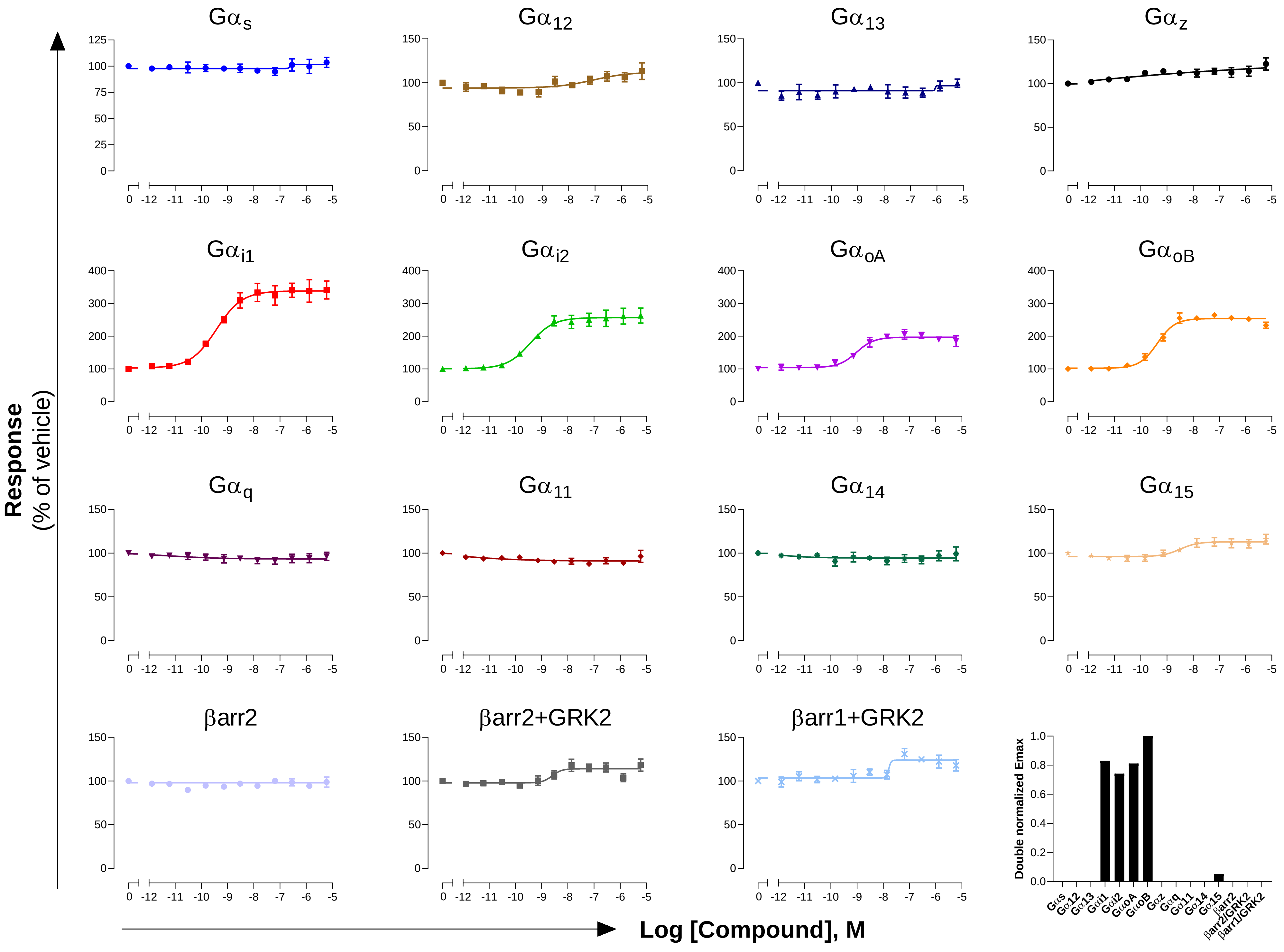

GPCR: NOP
Ligand: Nociceptin

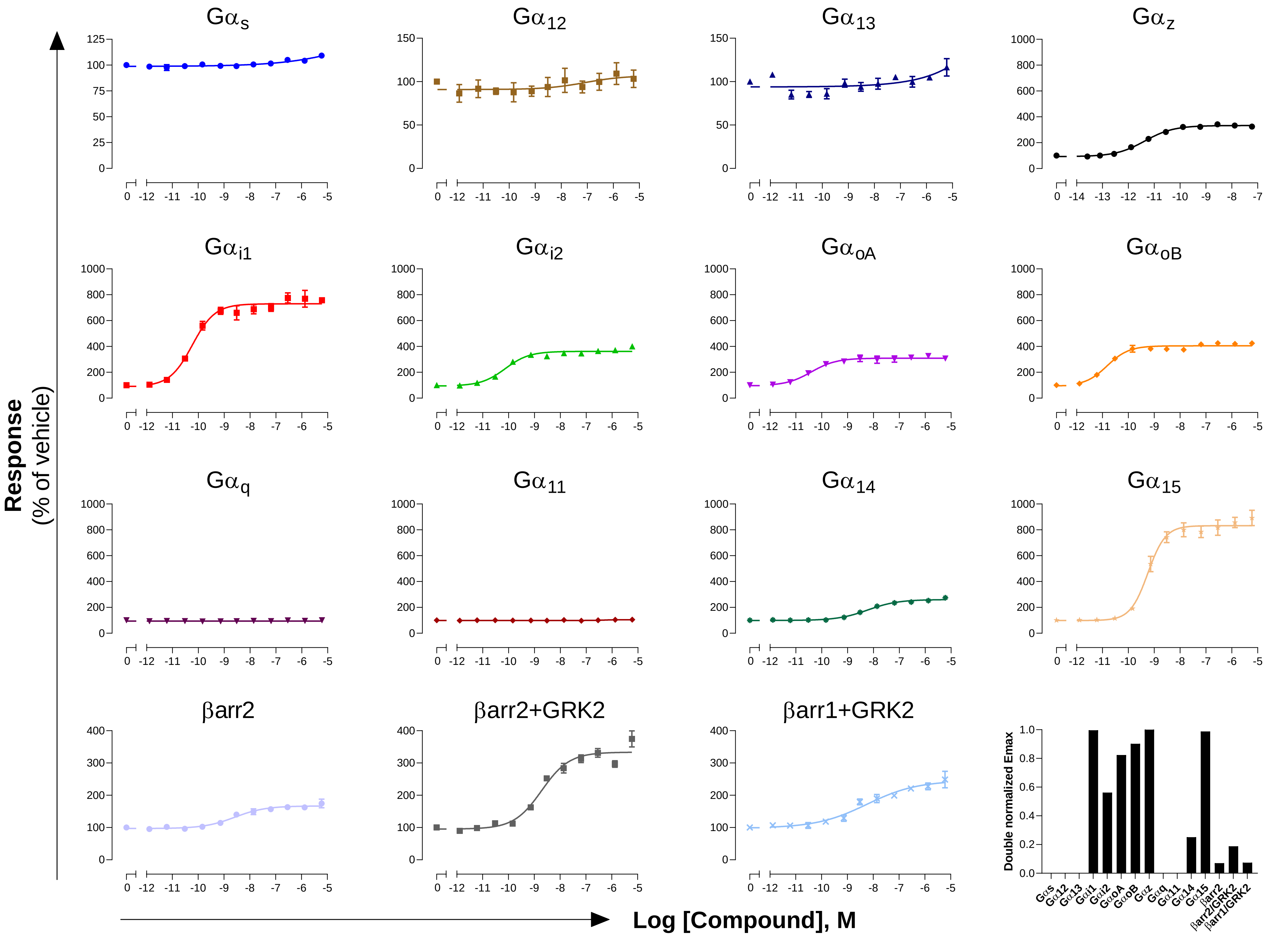

GPCR: NPFF1
Ligand: RFRP3

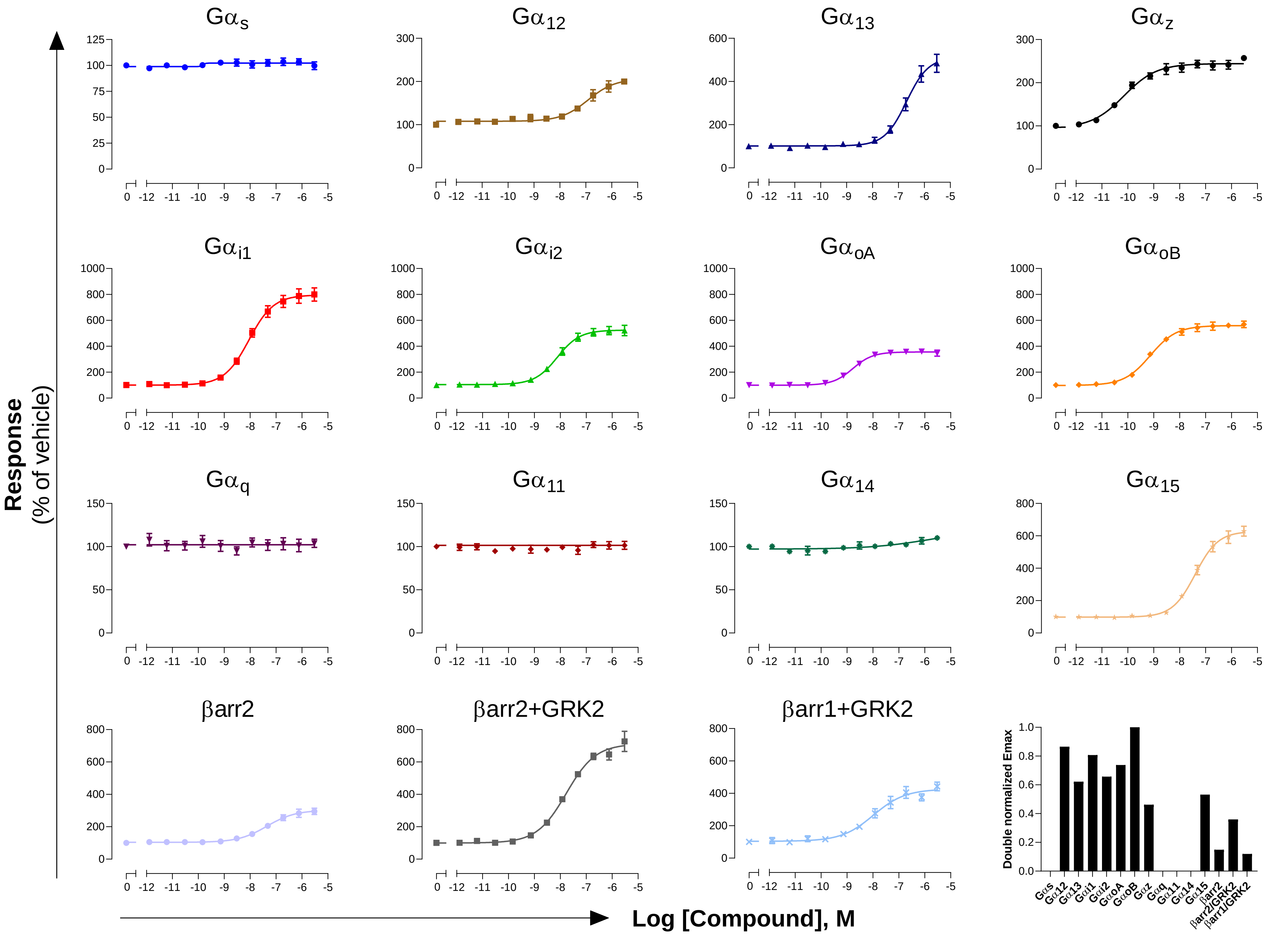

GPCR: NPFF2
Ligand: NPFF

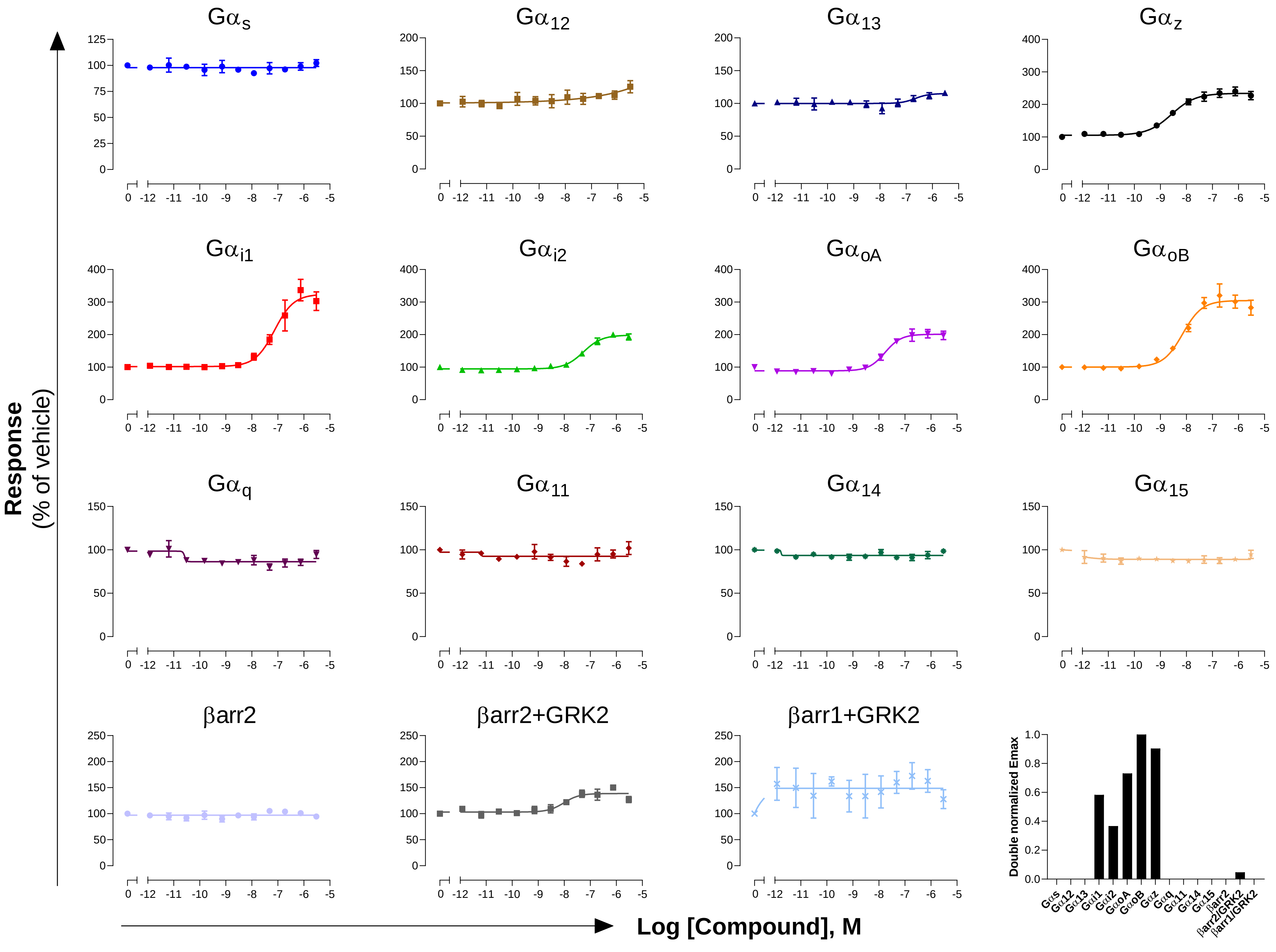

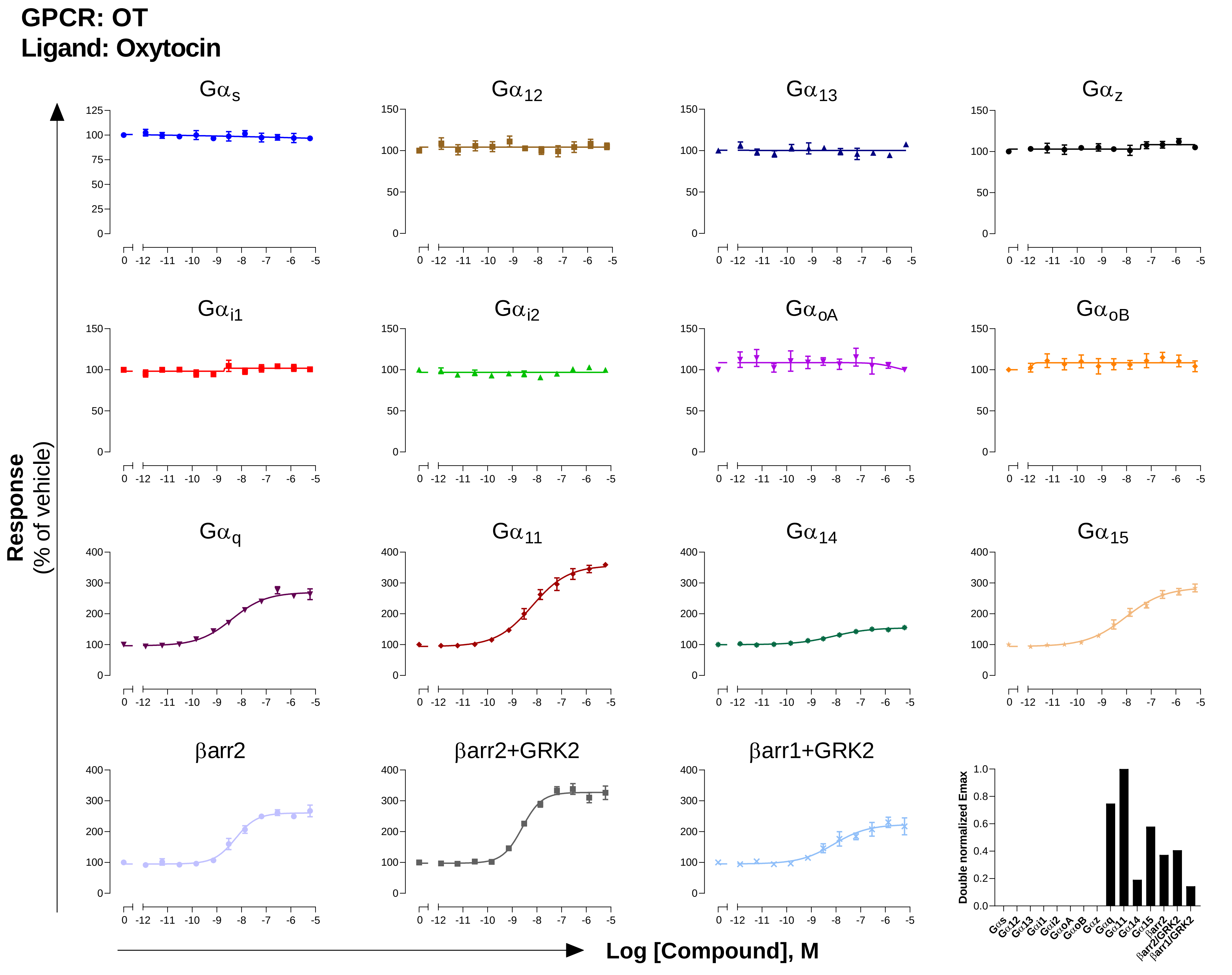

GPCR: OT
Ligand: Oxytocin
Gαs
Gα12
Gα13
Gαz
Gαi1
Gαi2
GαoA
GαoB
Gαq
Gα11
Gα14
Gα15
βarr2
βarr2+GRK2
βarr1+GRK2
Response
(% of vehicle)
Log [Compound], M
Double normalized Emax
βarr2/GRK2
βarr1/GRK2

GPCR: OX_2

Ligand: Orexin-A

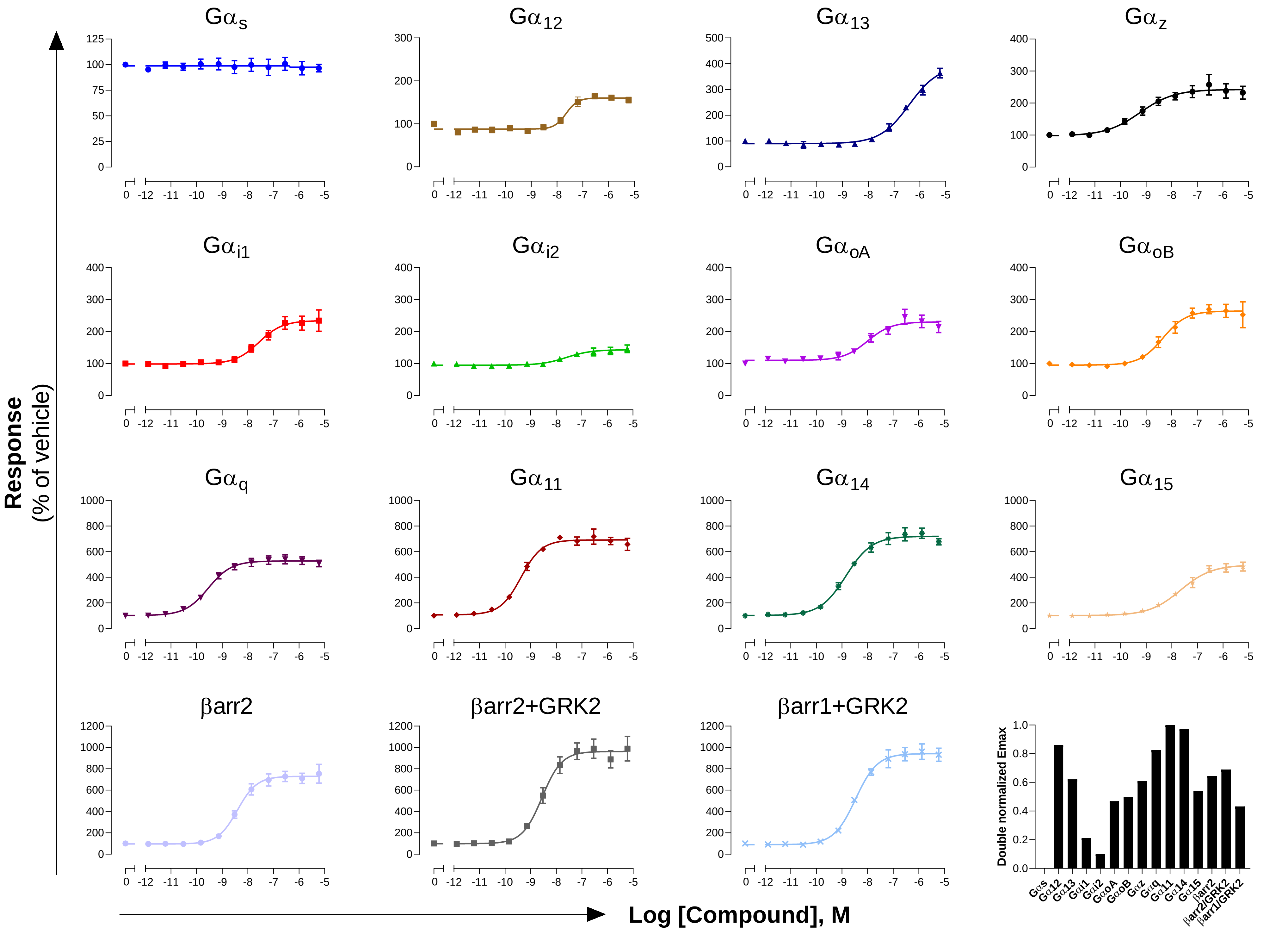

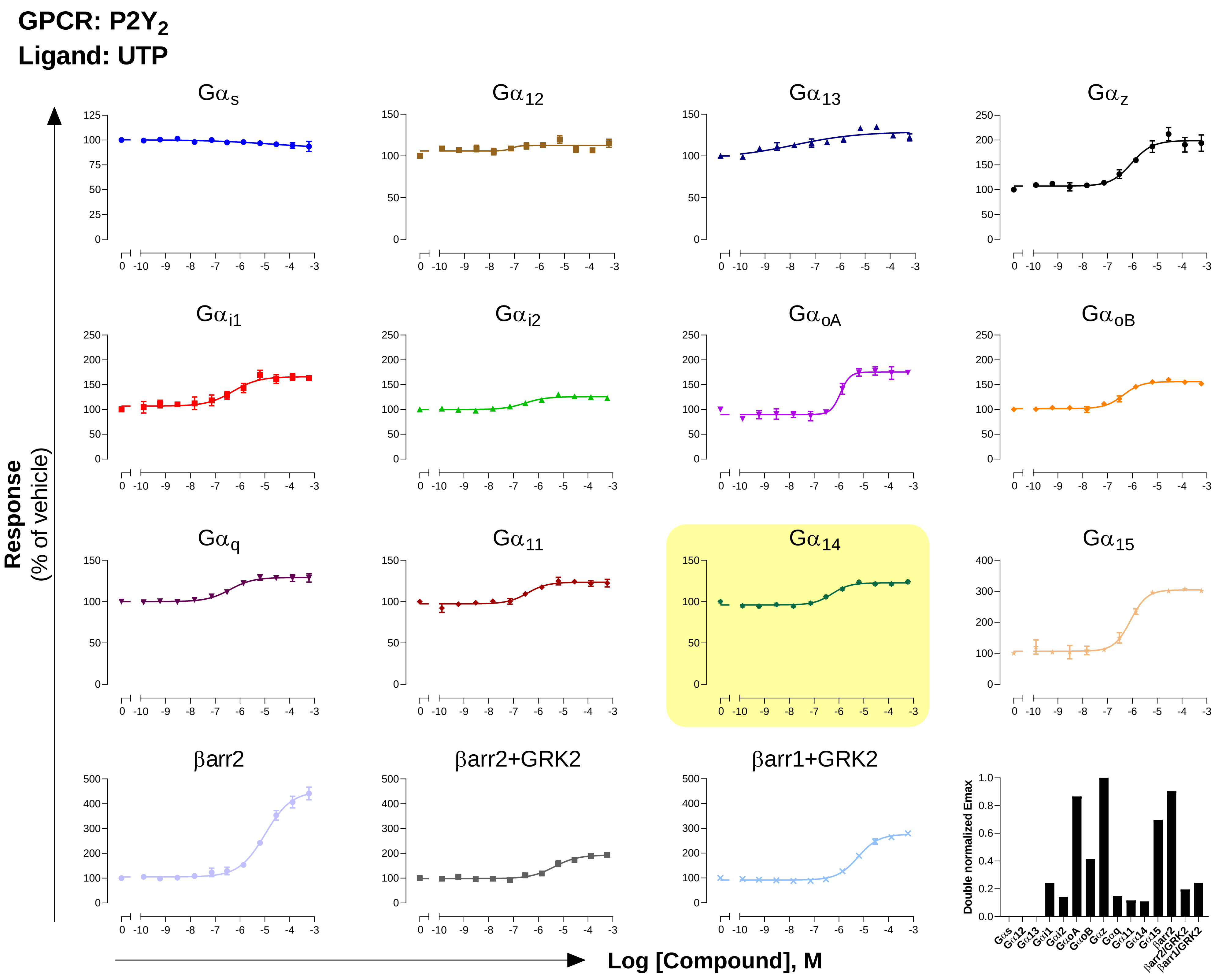

GPCR: P2Y2
Ligand: UTP
Gαs
Gα12
Gα13
Gαz
Gαi1
Gαi2
GαoA
GαoB
Gαq
Gα11
Gα14
Gα15
βarr2
βarr2+GRK2
βarr1+GRK2
Response
(% of vehicle)
Log [Compound], M
Double normalized Emax

GPCR: PAR1
Ligand: TFLLR-NH_2

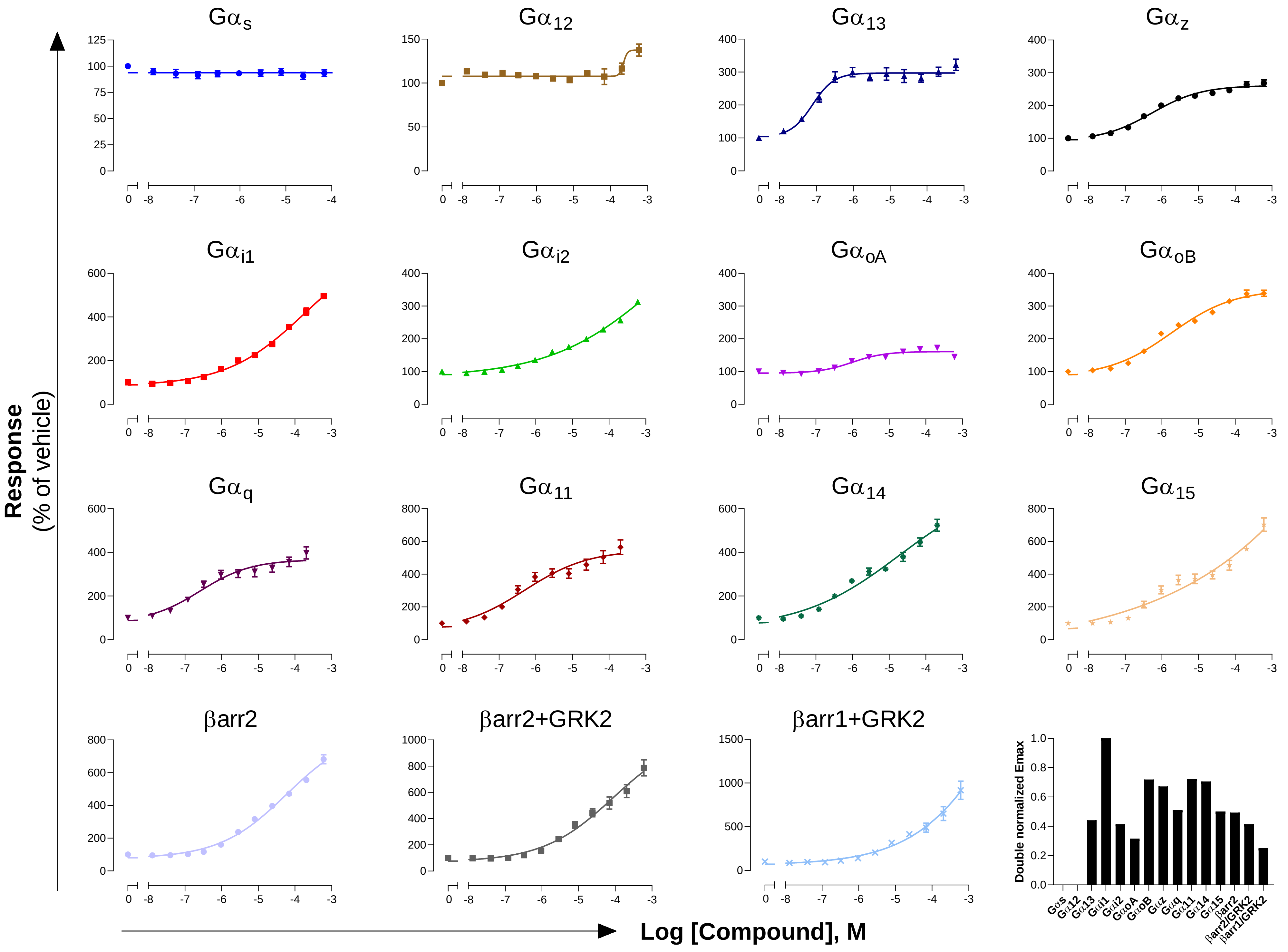

GPCR: PAR2
Ligand: SLIGKV-NH_2

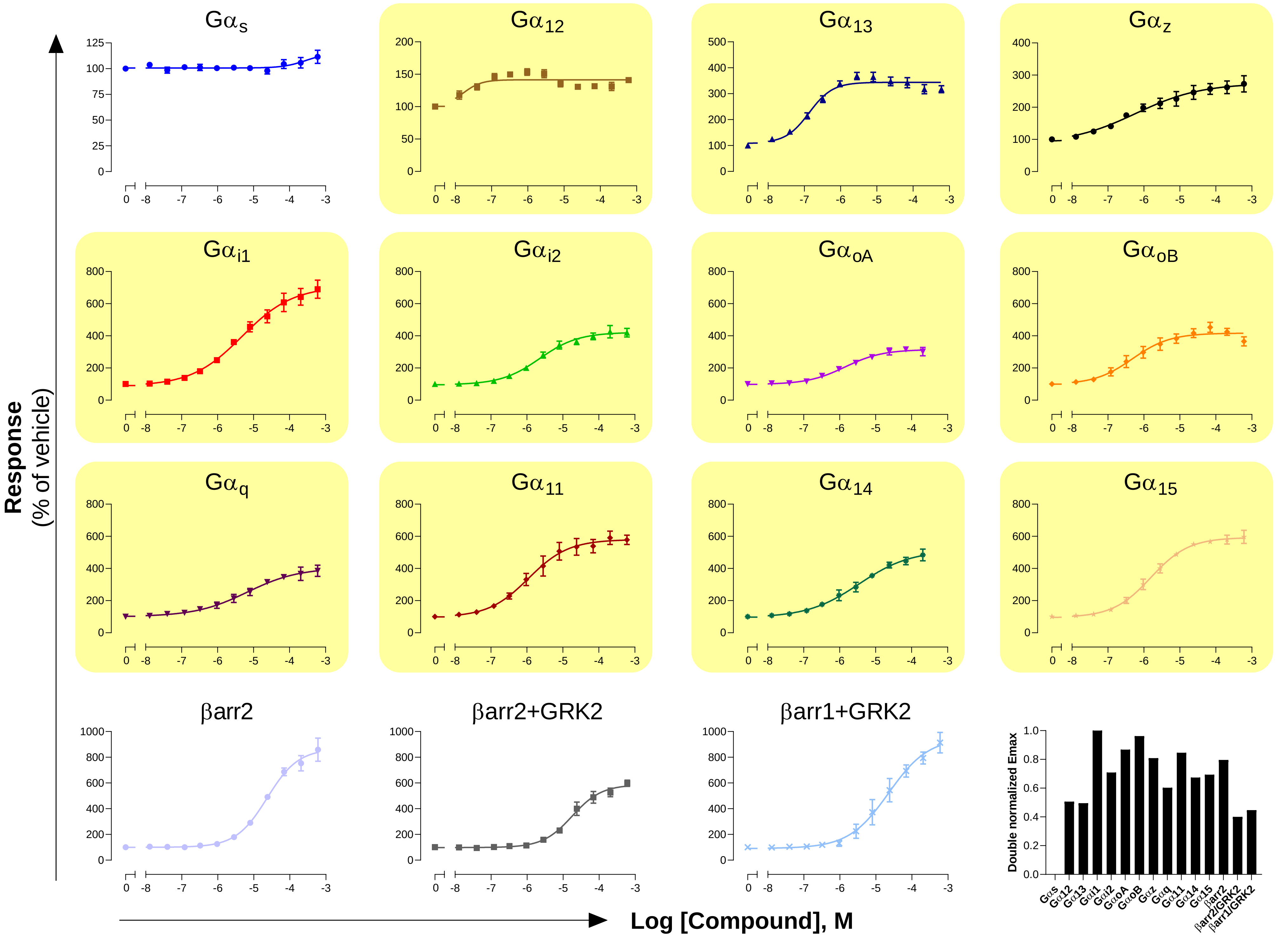

GPCR: PTH1
Ligand: PTH (1-34)

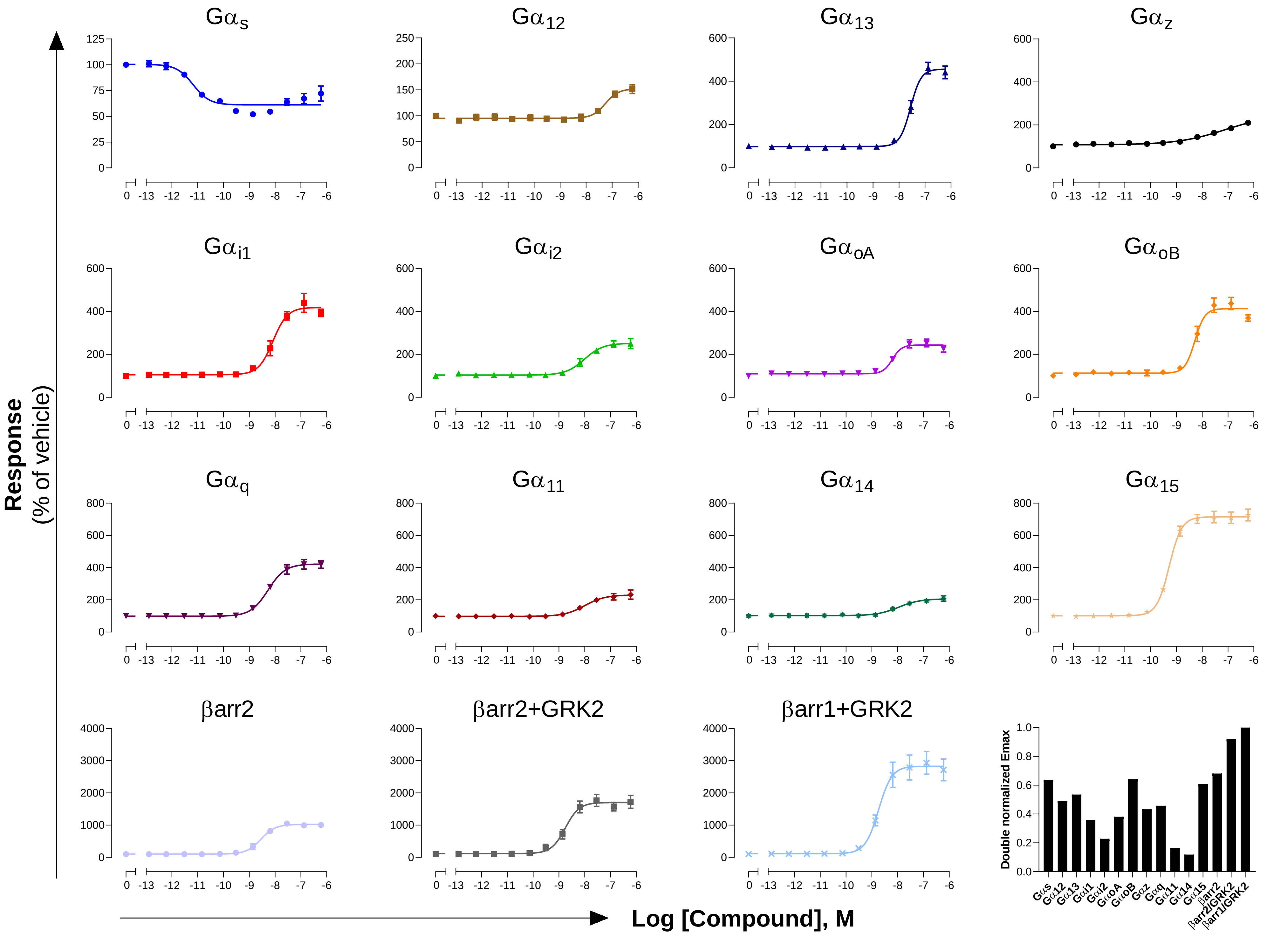

GPCR: S1P$_1$

Ligand: Sphingosine 1-phosphate

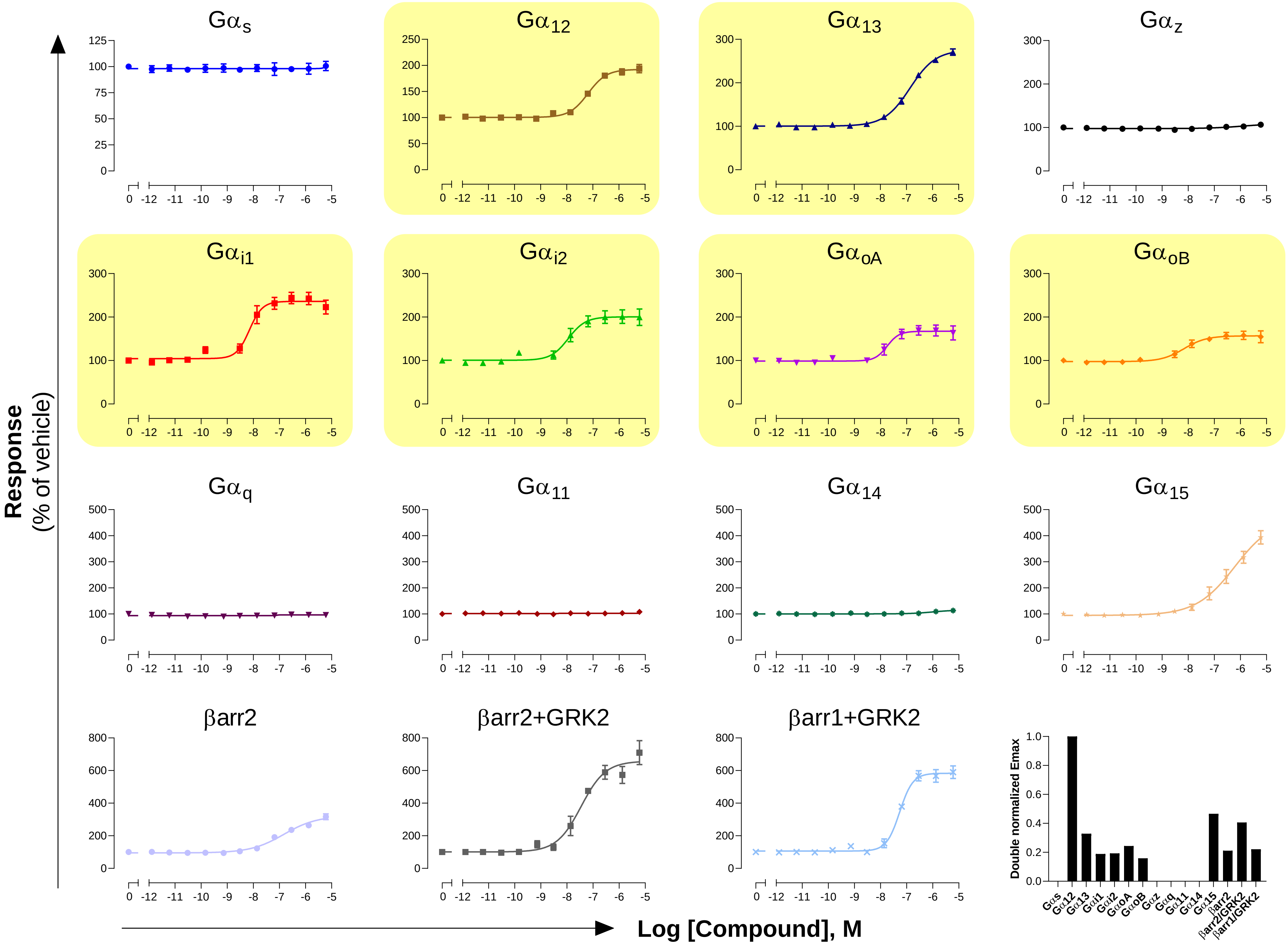

GPCR: SST_{2A}
Ligand: Somatostatin-14

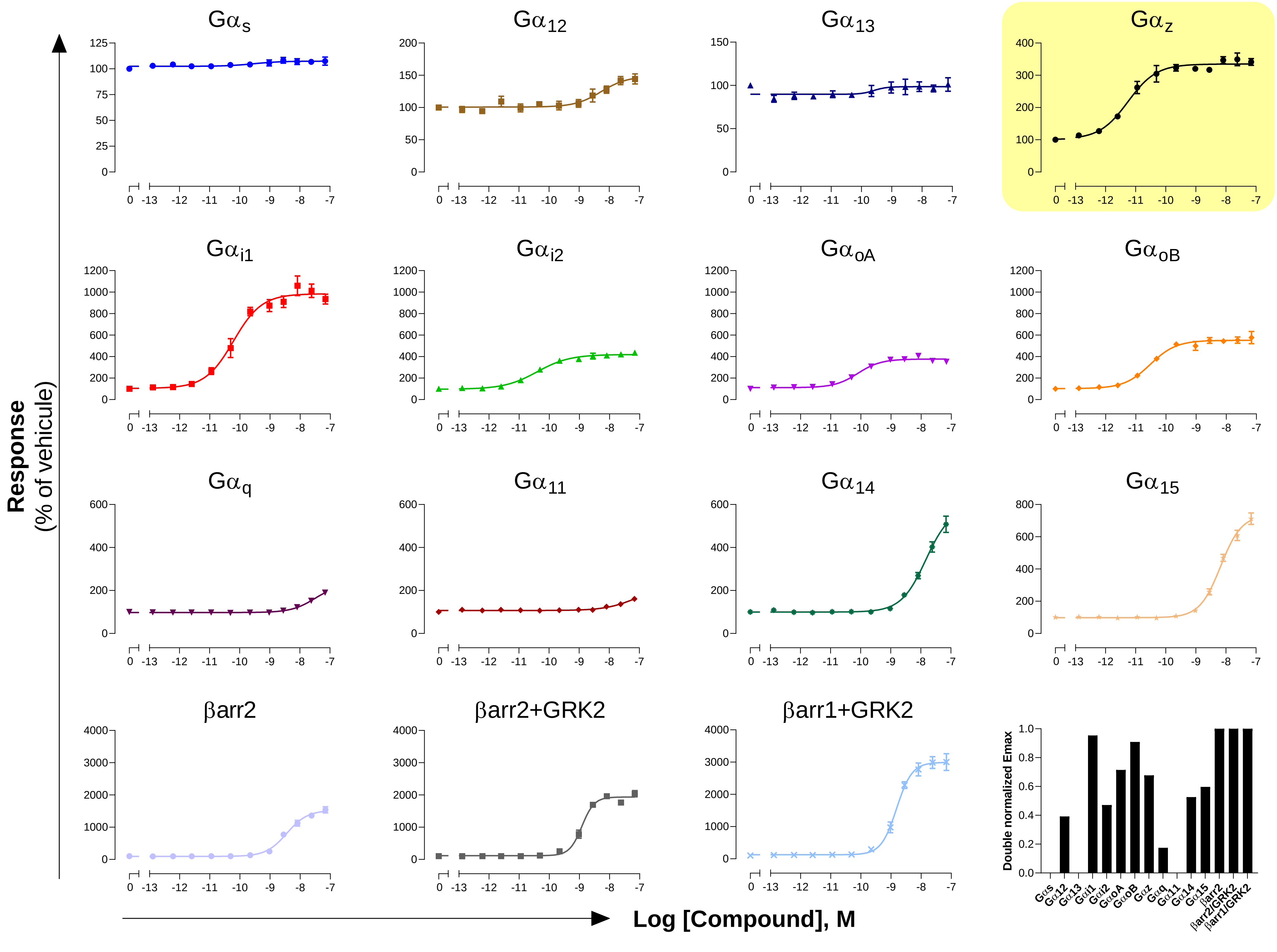

GPCR: V_{1A}
Ligand: AVP

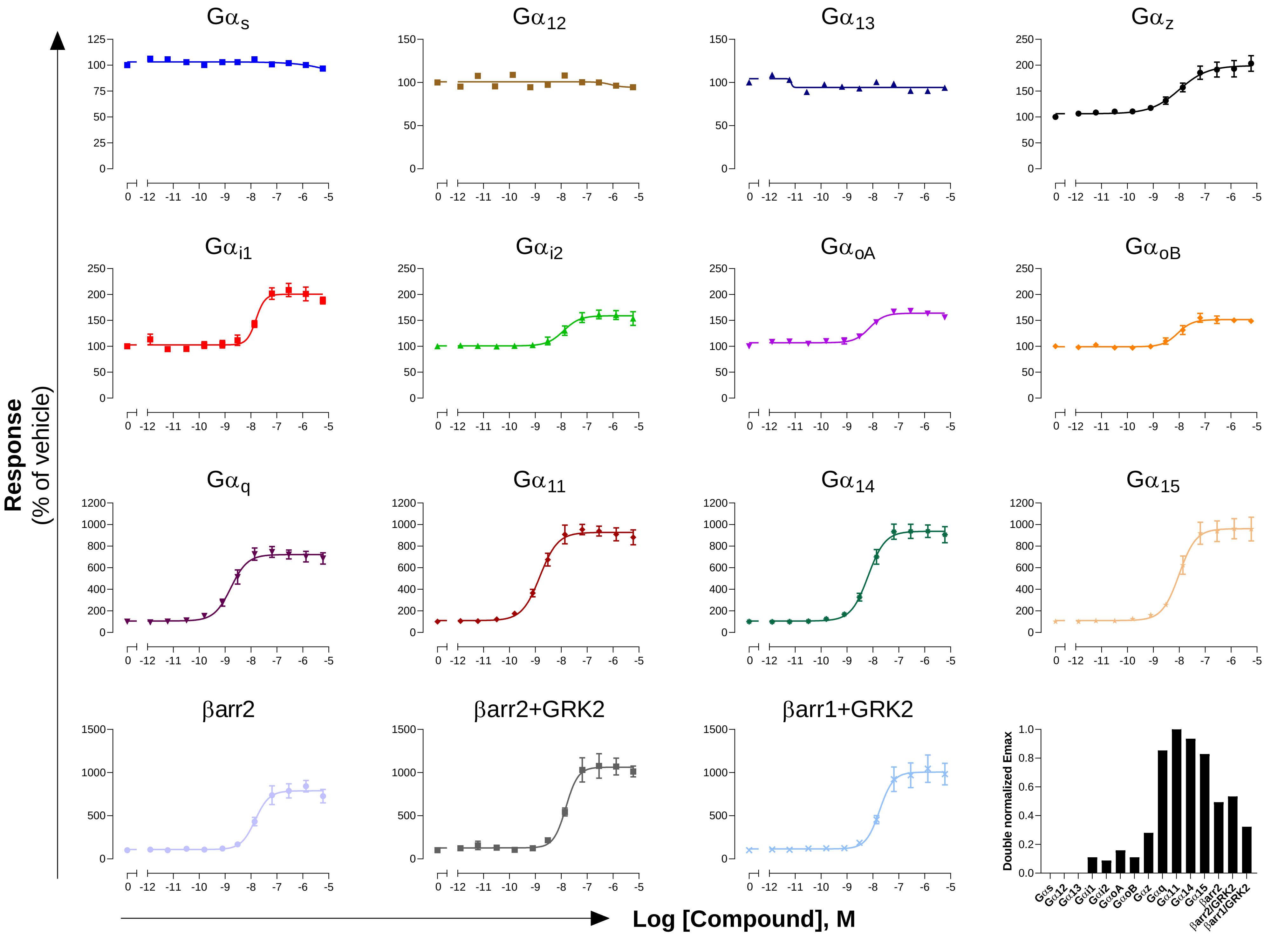

GPCR: V_2
Ligand: AVP

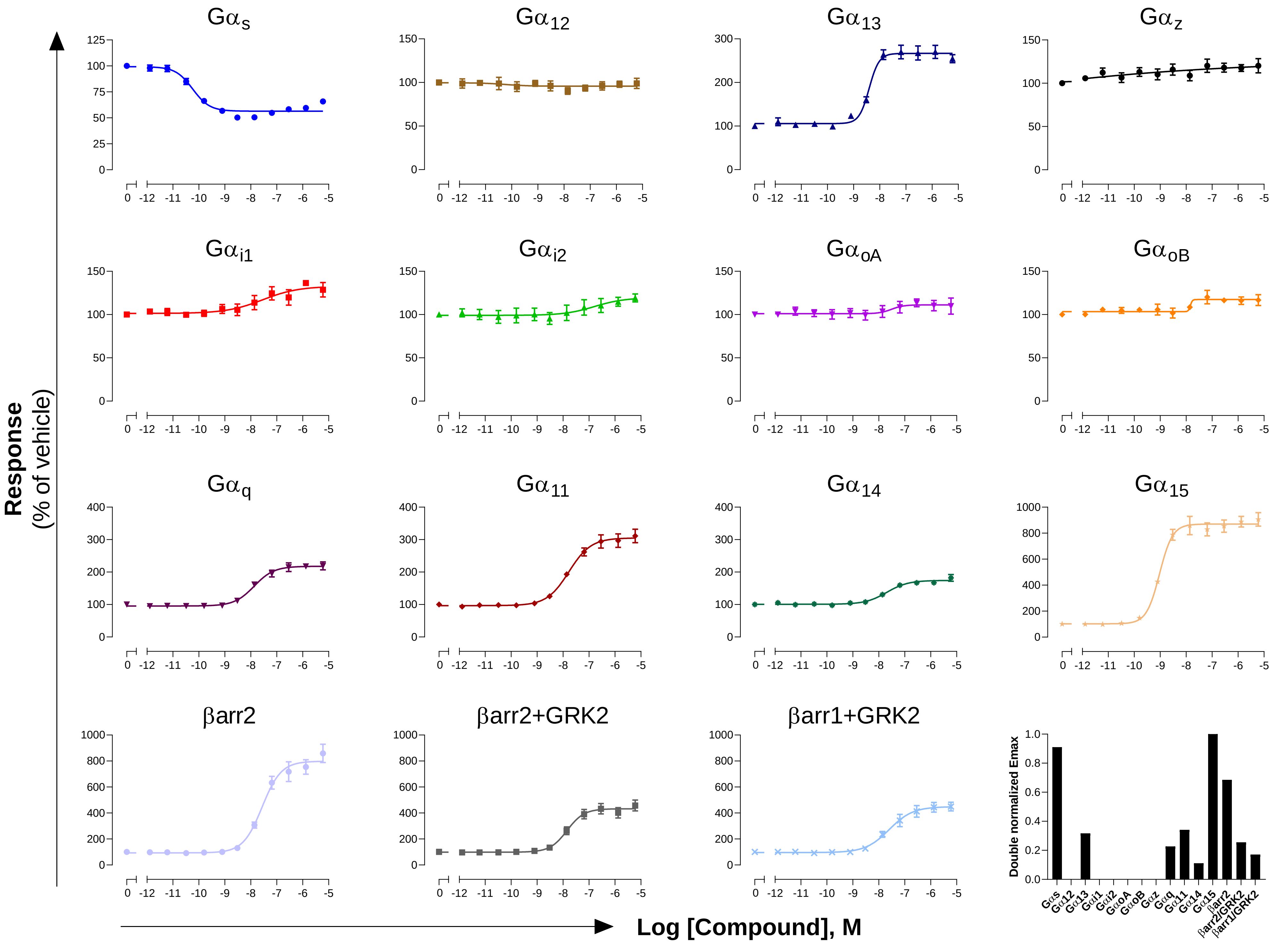

GPCR: VPAC$_1$
Ligand: VIP

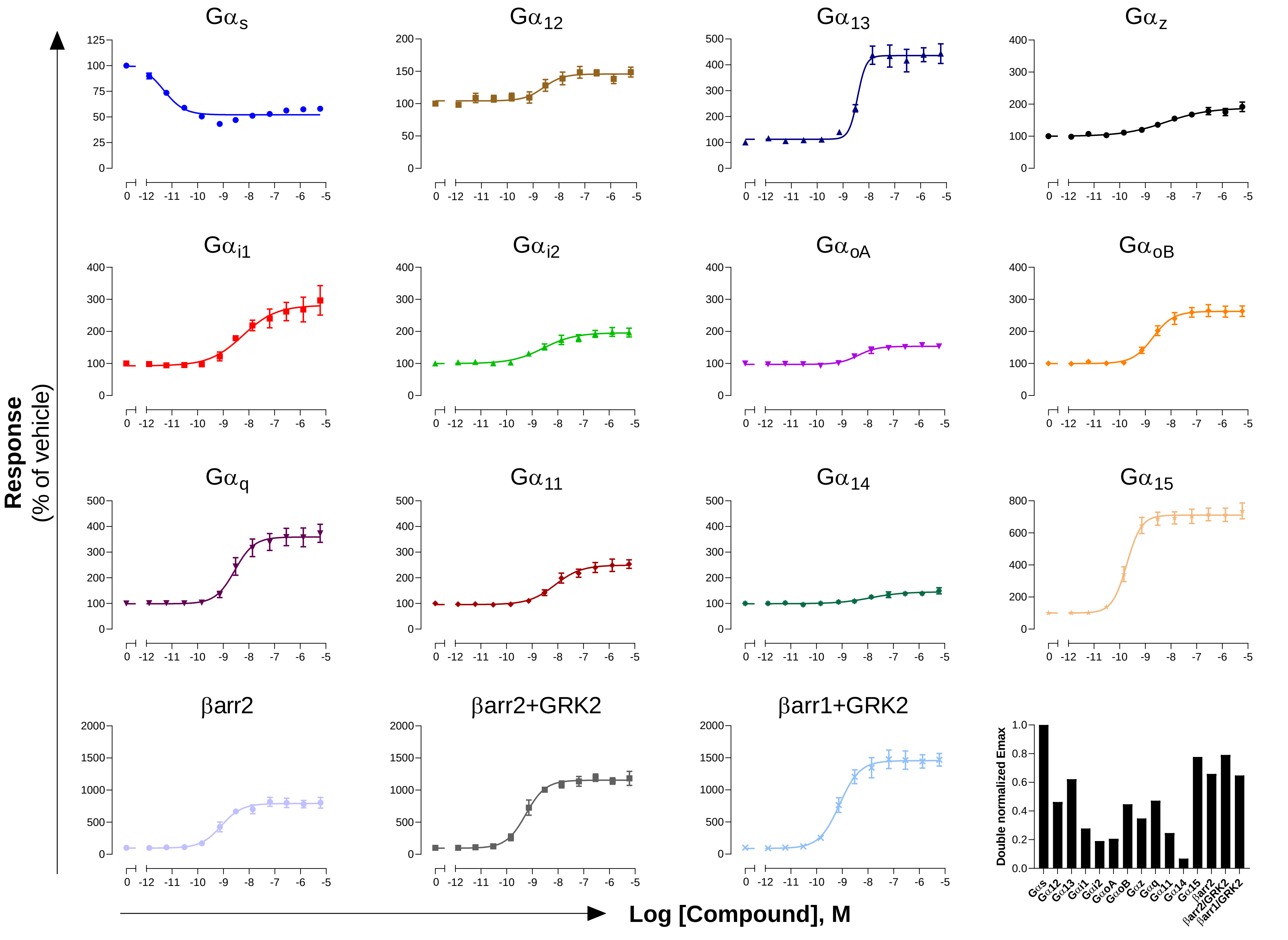

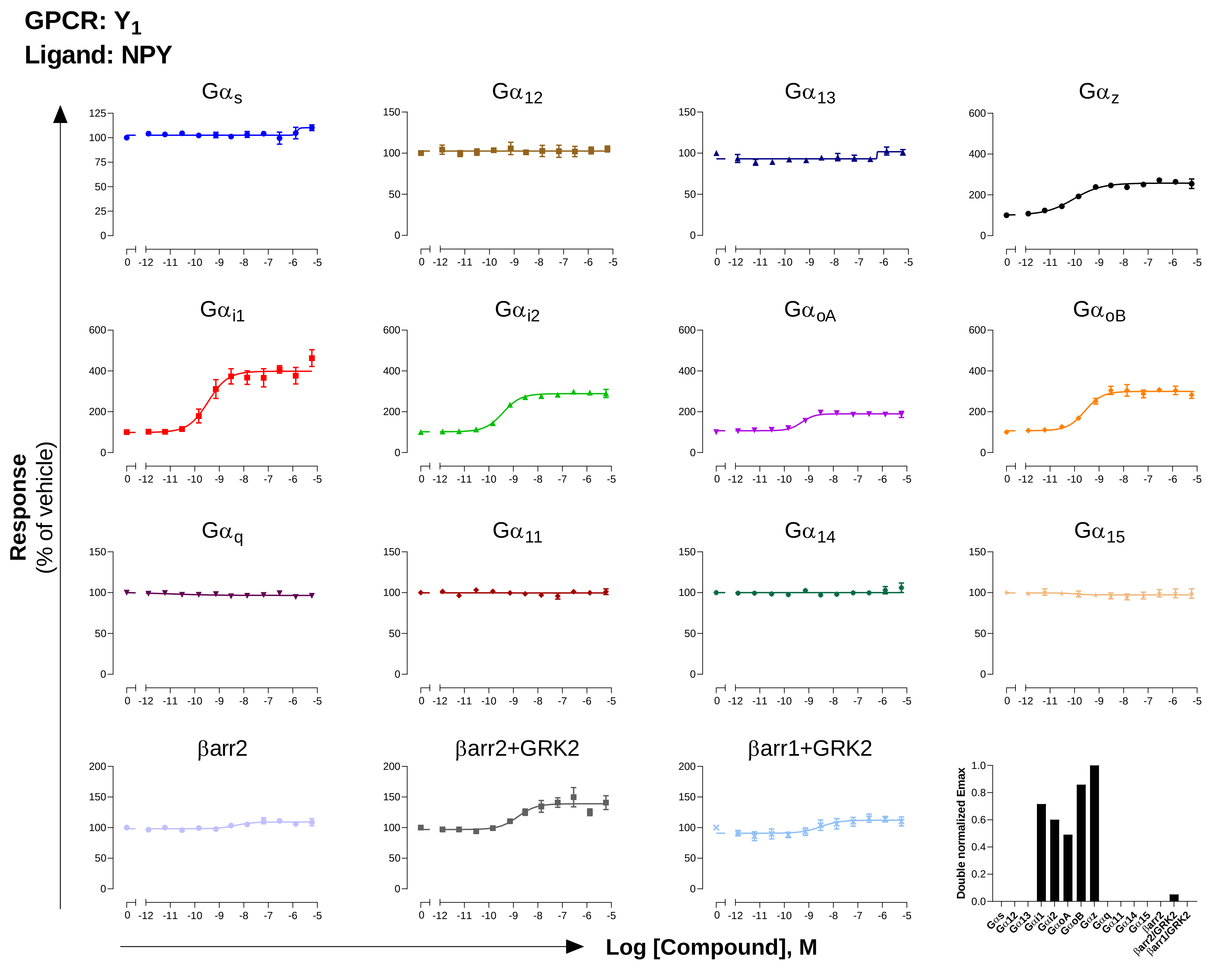

GPCR: Y1
Ligand: NPY
Gαs
Gα12
Gα13
Gαz
Gαi1
Gαi2
GαoA
GαoB
Gαq
Gα11
Gα14
Gα15
βarr2
βarr2+GRK2
βarr1+GRK2
Response
(% of vehicle)
Log [Compound], M
Double normalized Emax
Gαs
Gα12
Gα13
Gαi1
Gαi2
GαoA
GαoB
Gαz
Gαq
Gα11
Gα14
Gα15
βarr2
βarr2/GRK2
βarr1/GRK2

GPCR: Y_5
Ligand: NPY

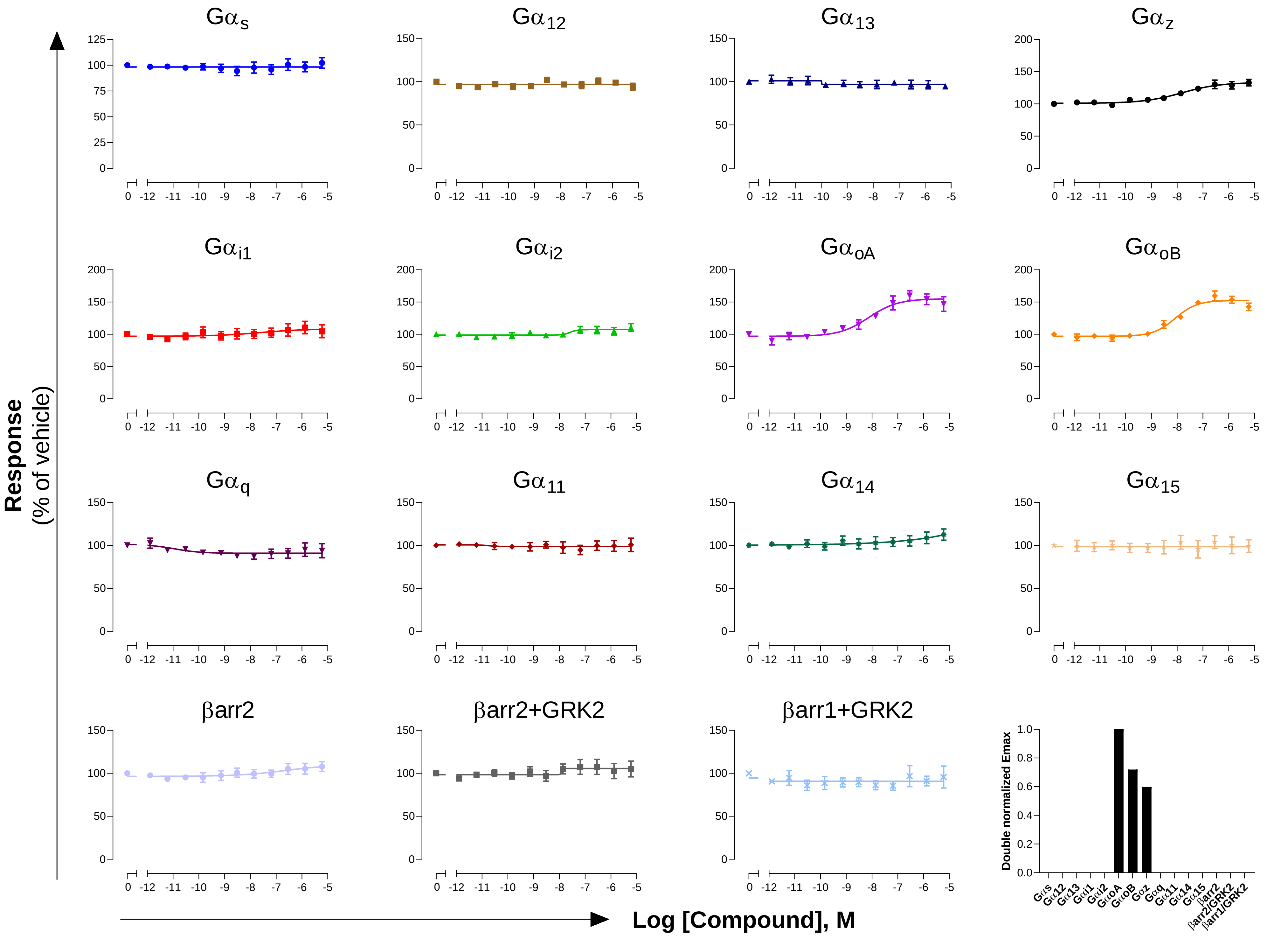

Supplement: Supplementary file 3. — Concentration-response curves in HEK293 cells expressing the indicated biosensor after stimulation of heterologously expressed receptor with the indicated ligand. Data are the mean ± SEM from at least 3 independent experiments and expressed in % of the response obtained for cells treated with vehicle. For ligands that elicited endogenous receptor-mediated responses (curves with light gray and yellow background for responses similar to and better responses than those obtained with the endogenous receptors, respectively), curves from cells expressing endogenous or heterologously expressed receptors are shown in Figure 3—figure supplement 2. [file elife-74101-supp3.pdf]
