# Supplementary material for: Effector membrane translocation biosensors reveal G protein and βarrestin coupling profiles of 100 therapeutically relevant GPCRs
Source: eLife. 2022 Mar 18;11:e74101. doi: 10.7554/eLife.74101 (PMC9005190; doi:10.7554/eLife.74101)
Supplement: Supplementary file 5. [file elife-74101-supp5.docx]

**Supplementary File 2:** **EMTA biosensor amino acid sequences**

Black: rGFP or Gα protein or G protein-effectors

Red: Linker

Purple: CAAX motif

Blue: RlucII

**rGFP-CAAX** MDLAKLGLKEVMPTKINLEGLVGDHAFSMEGVGEGNILEGTQEVKISVTKGAPLPFAFDIVSVAFSYGNRAYTGYPEEISDYFLQSFPEGFTYERNIRYQDGGTAIVKSDISLEDGKFIVNVDFKAKDLRRMGPVMQQDIVGMQPSYESMYTNVTSVIGECIIAFKLQTGKHFTYHMRTVYKSKKPVETMPLYHFIQHRLVKTNVDTASGYVVQHETAIAAHSTIKKIEGSLPGSAGTMASNNTASGGKKKKKKSKTKCVIM

**Gα_s_-67-RlucII**

MGCLGNSKTEDQRNEEKAQREANKKIEKQLQKDKQVYRATHRLLLLGAGESGKSTIVKQMRILHVNGSGGGGSMTSKVYDPEQRKRMITGPQWWARCKQMNVLDSFINYYDSEKHAENAVIFLHGNATSSYLWRHVVPHIEPVARCIIPDLIGMGKSGKSGNGSYRLLDHYKYLTAWFELLNLPKKIIFVGHDWGAALAFHYSYEHQDKIKAIVHAESVVDVIESWDEWPDIEEDIALIKSEEGEKMVLENNFFVETVLPSKIMRKLEPEEFAAYLEPFKEKGEVRRPTLSWPREIPLVKGGKPDVVQIVRNYNAYLRASDDLPKMFIESDPGFFSNAIVEGAKKFPNTEFVKVKGLHFSQEDAPDEMGKYIKSFVERVLKNEQSGGGGSFNGEGGEEDPQAARSNSDGEKATKVQDIKNNLKEAIETIVAAMSNLVPPVELANPENQFRVDYILSVMNVPDFDFPPEFYEHAKALWEDEGVRACYERSNEYQLIDCAQYFLDKIDVIKQADYVPSDQDLLRCRVLTSGIFETKFQVDKVNFHMFDVGGQRDERRKWIQCFNDVTAIIFVVASSSYNMVIREDNQTNRLQEALNLFKSIWNNRWLRTISVILFLNKQDLLAEKVLAGKSKIEDYFPEFARYTTPEDATPEPGEDPRVTRAKYFIRDEFLRISTASGDGRHYCYPHFTCAVDTENIRRVFNDCRDIIQRMHLRQYELL

**Rap1GAP (S437A/S439A/S441A)-RlucII**

MIEKMQGSRMDEQRCSFPPPLKTEEDYIPYPSVHEVLGREGPFPLILLPQFGGYWIEGTNHEITSIPETEPLQSPTTKVKLECNPTARIYRKHFLGKEHFNYYSLDAALGHLVFSLKYDVIGDQEHLRLLLRTKCRTYHDVIPISCLTEFPNVVQMAKLVCEDVNVDRFYPVLYPKASRLIVTFDEHVISNNFKFGVIYQKLGQTSEEELFSTNEESPAFVEFLEFLGQKVKLQDFKGFRGGLDVTHGQTGTESVYCNFRNKEIMFHVSTKLPYTEGDAQQLQRKRHIGAAIVAVVFQDENTPFVPDMIASNFLHAYVVVQAEGGGPDGPLYKVSVTARDDVPFFGPPLPDPAVFRKGPEFQEFLLTKLINAEYACYKAEKFAKLEERTRAALLETLYEELHIHSQSMMGLGGDEDKMENGSGGGGFFESFKRVIR**A**R**A**Q**A**MGSAGTGGRAIDIKLPATMTSKVYDPEQRKRMITGPQWWARCKQMNVLDSFINYYDSEKHAENAVIFLHGNATSSYLWRHVVPHIEPVARCIIPDLIGMGKSGKSGNGSYRLLDHYKYLTAWFELLNLPKKIIFVGHDWGAALAFHYSYEHQDKIKAIVHAESVVDVIESWDEWPDIEEDIALIKSEEGEKMVLENNFFVETVLPSKIMRKLEPEEFAAYLEPFKEKGEVRRPTLSWPREIPLVKGGKPDVVQIVRNYNAYLRASDDLPKMFIESDPGFFSNAIVEGAKKFPNTEFVKVKGLHFSQEDAPDEMGKYIKSFVERVLKNEQ

**p63-RhoGEF-RlucII**

MIMKYQLLLKDFLKYYNRAGMDTADLEQAVEVMCFVPKRCNDMMTLGRLRGFEGKLTAQGKLLGQDTFWVTEPEAGGLLSSRGRERRVFLFEQIIIFSEALGGGVRGGTQPGYVYKNSIKVSCLGLEGNLQGDPCRFALTSRGPEGGIQRYVLQAADPAISQAWIKHVAQILESQRDFLNALQSPIEYQRRESQTNSLGRPRGPGVGSPASGSAGTGGRAIDIKLPATMTSKVYDPEQRKRMITGPQWWARCKQMNVLDSFINYYDSEKHAENAVIFLHGNATSSYLWRHVVPHIEPVARCIIPDLIGMGKSGKSGNGSYRLLDHYKYLTAWFELLNLPKKIIFVGHDWGAALAFHYSYEHQDKIKAIVHAESVVDVIESWDEWPDIEEDIALIKSEEGEKMVLENNFFVETVLPSKIMRKLEPEEFAAYLEPFKEKGEVRRPTLSWPREIPLVKGGKPDVVQIVRNYNAYLRASDDLPKMFIESDPGFFSNAIVEGAKKFPNTEFVKVKGLHFSQEDAPDEMGKYIKSFVERVLKNEQ

**PDZ-RhoGEF-RlucII**

MQGVDQSPKPLIIGPEEDYDPGYFNNESDIIFQDLEKLKSRPAHLGVFLRYIFSQADPSPLLFYLCAEVYQQASPKDSRSLGKDIWNIFLEKNAPLRVKIPEMLQAEIDSRLRNSEDARGVLCEAQEAAMPEIQEQIHDYRTKRTLGLGSLYGENDLLDLDGDPLRERQVAEKQLAALGDILSKYEEDRSAPMDFALNTYMSHAGIRLREALKLPATMTSKVYDPEQRKRMITGPQWWARCKQMNVLDSFINYYDSEKHAENAVIFLHGNATSSYLWRHVVPHIEPVARCIIPDLIGMGKSGKSGNGSYRLLDHYKYLTAWFELLNLPKKIIFVGHDWGAALAFHYSYEHQDKIKAIVHAESVVDVIESWDEWPDIEEDIALIKSEEGEKMVLENNFFVETVLPSKIMRKLEPEEFAAYLEPFKEKGEVRRPTLSWPREIPLVKGGKPDVVQIVRNYNAYLRASDDLPKMFIESDPGFFSNAIVEGAKKFPNTEFVKVKGLHFSQEDAPDEMGKYIKSFVERVLKNEQ
